# Porphyrin‐Geländer–Helical Conjugated Banister Type Porphyrin Dyads

**DOI:** 10.1002/chem.202503049

**Published:** 2026-02-06

**Authors:** Joël F. Keller, Adriano D'Addio, Marcel Mayor

**Affiliations:** ^1^ Department of Chemistry University of Basel Basel Switzerland; ^2^ Institute For Nanotechnology (INT) and Karlsruhe Nano Micro Facility (KNMFi) Karlsruhe Institute of Technology (KIT) P. O. Box 3640 Karlsruhe Germany; ^3^ Lehn Institute of Functional Materials(LIFM) School of Chemistry Sun Yat‐Sen University (SYSU) Guangzhou P.R. of China

**Keywords:** exciton chirality method, geländer molecules, porphyrin dyads, optical spectroscopy

## Abstract

A new “Porphyrin‐Geländer” (**PoGe**) macrocycle *rac*‐**PoGe[Zn, Zn]** embodying a twisted butadiyne‐linked porphyrin dyad as the banister was designed and synthesized according to a previously published successful design principle for Geländer molecules. The C_2_‐symmetric properties of *rac‐* **PoGe[Zn, Zn]** were revealed by detailed 2D NMR analysis. Postsynthesis modification consisting of central metal ion exchange in the porphyrin moieties led to **PoGe** macrocycles *rac‐* **PoGe[2H, 2H]** and *rac‐* **PoGe[Cu, Cu]** and was demonstrated to lead to finely tunable absorption and emission spectra, reaching in the near‐IR region. CSP‐HPLC resolved all *rac‐* **PoGe[M, M]** into their (*P*) and (*M*) enantiomers, which were analyzed by UV‐Vis, fluorescence and ECD spectroscopy. The absolute configuration was assigned by the exciton chirality method (ECM) and corroborated by comparing experimental and simulated ECD spectra.

## Graphical abstract

Porphyrin arrays go chiral–A porphyrin Geländer macrocycle represents the intriguing prototype architecture of helically chiral butadiyne‐linked porphyrin arrays. This study combines synthetic, spectroscopic, and computational methods to shed light on its structure, chiroptical properties, and tunability, highlighting its promise for advancing functional optoelectronic materials.

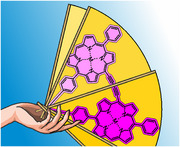

## 1 Introduction

**FIGURE 1:**
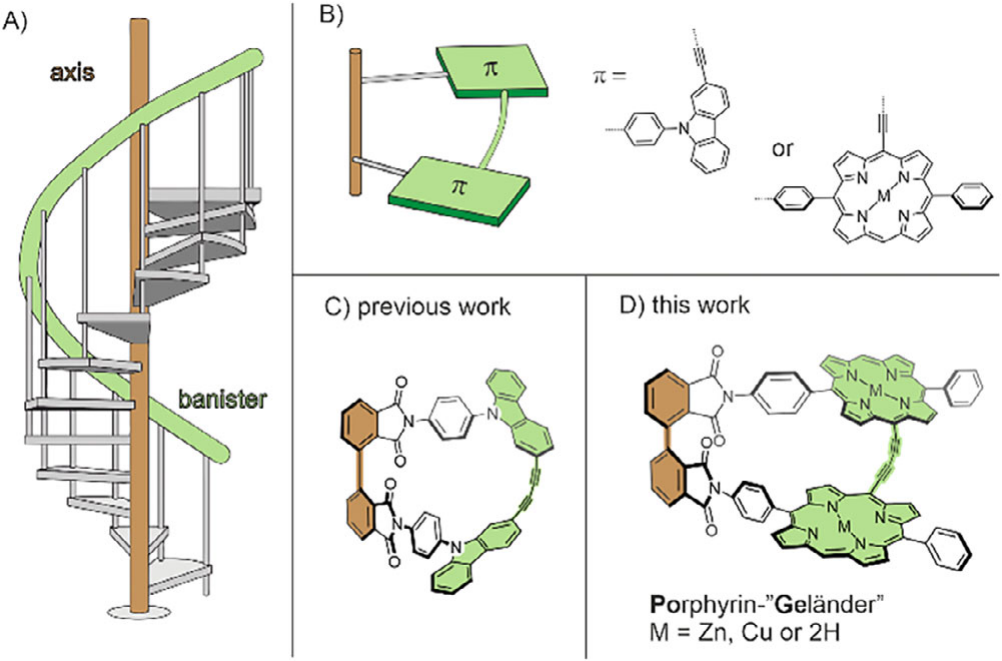
(A) Representation of the eponymous spiral staircase. The axis is depicted in brown and in green the banister. (B) Cartoon representation of two repeating units in a symmetric “Geländer” oligomer with orthogonal joints. The 2,9‐substitution ensured the 90° angle in our previously reported dimer (C), and orthogonality is also ensured by the substitution pattern of 5,10‐porphyrin, leading to **PoGe[M, M]** (D).

Porphyrins are well‐studied chromophores prevalent in nature and an appealing compound class in the design of novel functional materials due to their distinct, well‐studied optical features. Chirality is an emerging design factor for future materials and technologies, such as spintronics, displays, sensing, and pharmaceuticals [1, 2, 3]. Even though the parent porphine scaffold is achiral, there is ongoing research to harvest its spectral features for chiroptically active materials as its optical features may be minutely controlled. Different strategies have been employed in recent years towards helical porphyrins and porphyrinoids. Rather unique are helical “twin” porphyrins where the pyrazole‐expanded porphyrin takes a helical conformation [4, 5] and peripheral π‐extension of the scaffold leading to incorporation into helicenes [6, 7, 8] or propeller‐like structures [9]. In another approach the head and tail of helicenes are decorated with porphyrins [10]. Further helical structures can also be obtained by introducing strain by length mismatch of *meso* and β‐tethers in porphyrin dyads [11, 12, 13]. All these approaches either distort the parent scaffold or move the porphyrin's helical geometry in the periphery. *Vögtle* and coworkers pioneered an approach towards helicity in the 90s. The structures were reminiscent of the banister (or “Geländer” in German) of a spiral staircase (Figure 1A) as elongated linkers (banister) are forced to wrap helically around an oligophenylene rod (axis) [14, 15]. In recent years, we have developed the synthesis of “Geländer” structures by size mismatch of two stringers in ladder‐type molecules, resulting in dynamic, helically pure molecules [16, 17, 18, 19, 20]. Our most recent advancement is the design of symmetric “Geländer” oligomers (Figure 1C) where 90° angles are central motifs (Figure 1B) of the design and were the first optically resolved helimers which showed to be stable towards racemization even at elevated temperatures and had optically efficient conjugated banisters [21, 22], Other approaches to rigid “Geländer” structures are the mechanical locking of the banister or using a rigid stringer as axis [23, 24, 25]. The square‐like shape of porphyrins and the relative ease of selectively addressing the *meso* positions make them ideal chromophores to be incorporated in a symmetric banister dimer (Figure 1B,1D). Therefore, we designed a new helical porphyrin dyad system with our established geometric framework [21], with butadiyne‐linked porphyrins as the banister, which we call porphyrin “Geländer” (**PoGe[M, M]**). Optical properties can be readily tuned by exchanging the metals coordinated to the porphyrin moieties, resulting in a series of chromophores with UV‐Vis absorption properties broadly covering the far red region. Due to the structural properties of porphyrins mentioned above, the well‐studied optical features and their efficient conjugation [10, 26, 27, 28, 29, 30, 31, 32, 33, 34], the designed dimers should give insights and a deeper understanding of the properties of “Geländer” structures.

## 2 Results and Discussion

### 2.1 Retrosynthesis

**SCHEME 1:**
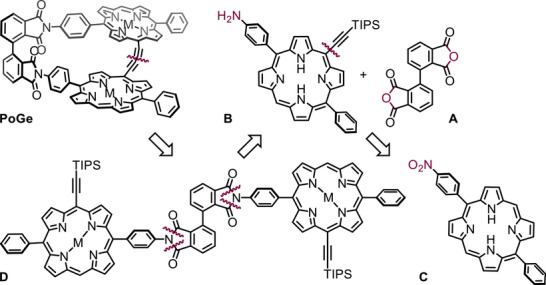
Retrosynthetic analysis of **PoGe**: disconnections and transformations of synthons are indicated with violet color. **PoGe** is cyclized from **D** by alkyne homocoupling following TIPS deprotection. Bis‐Imide **D** is condensed from aniline **B** and anhydride **A**. Aniline **B** is synthesized from porphyrin **C** in multiple steps involving *meso*‐bromination, Zn(II) metalation, *Sonogashira‐Hagihara* coupling and nitro reduction.

**SCHEME 2:**
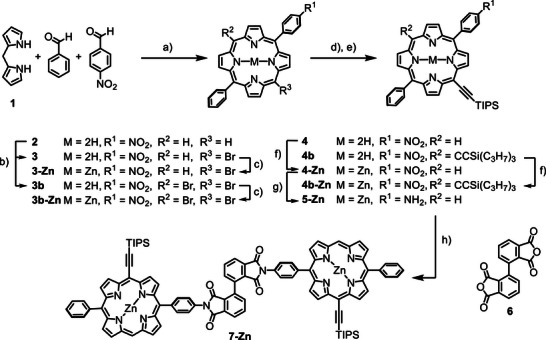
Synthesis of **7‐Zn**. Reagents and conditions: (a) 1) benzaldehyde, 4‐nitrobenzaldehyde, TFA, CH_2_Cl_2_, r.t., 16 h 2) DDQ, r.t., 1 h, 17% (b) NBS, pyridine, CHCl_3_, r.t., 2 h, **3**: 34%, **3b**: 18% (c) Zn(OAc)_2_, CH_2_Cl_2_/MeOH (9:1), 40 °C, 4 h, **3‐Zn**: 98%, **3b‐Zn**: 97% (d) 1) Pd(PPh_3_)_2_Cl_2_, CuI, TIPSA, NEt_3_/THF (3:1), 50 °C, 14 h (e) TFA, CH_2_Cl_2_, r.t., 10 min, **4**: 31% over 4 steps, **4b**: 11% over 4 steps (f) Zn(OAc)_2_, CH_2_Cl_2_/MeOH (9:1), 40 °C, 4–12 h, **4‐Zn**: 98%, **4b‐Zn**: 97% (g) hydrazine monohydrate, Pd/C, THF, 80 °C, 7 h, 78% h) NMP, 2 h, mw, 180 °C, 91%.

The retrosynthetic analysis (Scheme 1) of **PoGe[M, M]** reveals an oxidative acetylene homocoupling for the *geländerfication*–forming the banister. In recent years, we have successfully used either *Eglinton* conditions [21] or *Eglinton‐Breslow* conditions [35, 36, 37, 38, 39, 40], for similar structures. Complementing our previously published strategy, where axis A is formed after incorporating the chromophore, we found that this order can be reversed. This stratetgy potentially enables the rapid assembly of dimeric “Geländer” structures embedding other chromophores. In contrast to our previous strategy, axis **A** can be assembled in literature‐known protocols before the imide condensation with ABCD‐type porphyrin **B** (Scheme 2). This generally allows the exchange of the chosen chromophore to be embedded within the “Geländer” scaffold. The ABCD‐type porphyrin **B** was identified as the crucial building block for the synthetic plan as this ensures the 90° angle in the junction connecting the banister to the axis. **B** can be synthesized from the trans AB_2_C‐porphyrin **C** in a sequence of bromination, Zn(II) metalation, *Sonagashira‐Hagihara* coupling, and nitro to aniline reduction to provide all necessary functional groups. Blocking of the *meso* position *trans* to the rung is essential to ensure the 90° angle. Disconnection of **C** leads to the statistical porphyrin condensation of 4‐nitrobenzaldehyde, benzaldehyde, and dipyrromethane to ensure the *trans* substitution pattern.

### 2.2 Synthesis of Zinc Porphyrin‐Dimer 7‐Zn

Zinc 5‐(4‐nitrophenyl)‐15‐phenyl porphyrin (**2**) was synthesized by a modified literature procedure [41] from 4‐nitrobenzaldehyde, benzaldehyde, and dipyrromethane **1**. The ratio between electron‐poor and electron‐rich aldehyde was kept as suggested by *Banfi* et al., however, the yield of the unsymmetrically substituted porphyrin **2** could be increased from 5.5% to 17% by basifying the reaction mixture with NEt_3_ before purification on silica gel. The nitrophenyl group of **2** was readily reduced to the aniline with SnCl_2_ in HCl/EtOH. Unfortunately, subsequent attempts to brominate in *meso*‐position failed due to the high susceptibility of the electron‐rich aniline to bromination in *ortho*‐position to the amine. Highly dilute statistical *meso*‐bromination of the poorly soluble free‐base porphyrin **2** with *N*‐bromosuccinimide in CHCl_3_/pyridine resulted in a mixture of the desired monobrominated compound **3** and the dibrominated compound **3b**. Attempts at the statistical bromination of the better soluble zinc‐complex of **2** (or **2‐Zn**) under identical conditions resulted in the recovery of only starting material and dibrominated product. Mixtures of **3** and **3b** can be separated by tedious column chromatography, but for preparative purposes, separating the mixture at a later step proved to be more efficient. Metalation of the bromoporphyrin mixture with Zn(OAc)_2_ and *Sonogashira* coupling with TIPS‐acetylene gave a mixture of **4‐Zn** and **4b‐Zn**, which both showed good solubility in cyclohexane/CH_2_Cl_2_. Demetalation of the mixture with TFA/CH_2_Cl_2_ finally enabled simple and economic isolation of **4** by column chromatography in a yield of 31% over four steps. Reduction of the nitro group of **4** using SnCl_2_/HCl or SnCl_2_/formic acid in EtOH resulted in substrate degradation, and the desired aniline could be isolated only in trace amounts. Heating **4** with Zn dust/AcOH in EtOH only resulted in sluggish metalation of **4** to **4‐Zn**, but no reduction to the aniline. With Zn dust/NH_4_Cl in THF/MeOH, the desired aniline was isolated in 30–50% yield, after treatment of the crude product with TFA/CH_2_Cl_2_ to reverse the partial metalation that occurred during the reduction process, along with the symmetrical diazene in 20–30% yield. As product yields were not consistently reproducible and the diazene side product stemming from the nitrosyl intermediate could not be suppressed, we next investigated acid‐free methods to reduce the nitro group. To prevent the complexation of metals used in the reduction process under basic conditions, **4** was remetalated to **4‐Zn** with Zn(OAc)_2_ in 98% yield. A combination of CuSO_4_ and NaBH_4_ in MeOH at 60°C led to complete reduction of the nitro group within several minutes, but also to partial reduction of the triple bond to the alkene, which could not be separated from the desired product. Finally, a Pd‐catalyzed reduction with hydrazine in THF [42] provided **5‐Zn** in a satisfying yield of 78% while keeping the acetylene intact. Next, we turned our attention to forming the bis‐imide **7‐Zn** from two equivalents of aniline **5‐Zn** and one equivalent of *ortho*‐bis‐phthalic anhydride **6**, which was synthesized from 3‐chloro phthalic anhydride in three steps according to literature known protocols [43]. Refluxing the components in AcOH resulted in acetylation of the aniline rather than imide formation. It was possible to perform the twofold nucleophilic anhydride ring opening to the bis‐amide precursor at 60°C in AcOH/1,2‐dichloroethane, followed by further heating of the mixture to 110°C which initiated imidization accompanied by partial degradation of the mixture. Acid‐free microwave‐assisted heating of the components at 180°C dissolved in minimal amounts of *N*‐methyl‐2‐pyrrolidone, keeping the reactant/water ratio as high as possible to shift the equilibrium to the product side, resulted in clean formation of the desired bis‐imide **7‐Zn** in 91% yield.

### 2.3 Geländerfication of 7‐Zn to *rac*‐PoGe[Zn, Zn]

**SCHEME 3:**
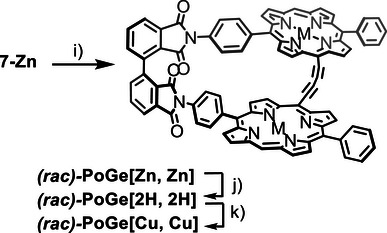
Synthesis of **PoGe‐[M, M]** series. Reagents and conditions: i) 1) TBAF, pyridine/CH_2_Cl_2_ (1:1), r.t., 2 h 2) Cu(OAc)_2_, CuCl, pyridine/CH_2_Cl_2_ (51:1), 50 °C, 18 h, 83% over both steps j) 1) TFA, 3 min., r.t., 61%, k) Cu(OAc)_2_, CH_2_Cl_2_/THF/NEt_3_, r.t., 2 d.

With the key intermediate **7**‐**Zn** in hand, we moved to the *geländerfication* step (Scheme 3). Using CuF_2_ under aerobic conditions to liberate the alkynes and oxidatively couple them in one pot allowed to observe the formation of ***rac*‐PoGe[Zn, Zn]** by MALDI‐ToF MS but it was not possible to isolate the target structure. To exclude air from the reaction we employed *Eglinton*‐*Breslow* conditions which due to an excess of Cu(I) and Cu(II) performs well under inert atmosphere [35, 38]. The alkynes were first liberated from the silyl protecting group in the presence of TBAF in pyridine/CH_2_Cl_2_ (1:1). After full conversion was observed by TLC and MALDI‐ToF MS the mixture was diluted to 10^−4^ M with more pyridine and extensively degassed with argon. Macrocyclization was then effected with excess Cu(OAc)_2_ and CuCl to provide ***rac*‐PoGe[Zn, Zn**], which was isolated by normal phase (np) HPLC (1% ethanol in CH_2_Cl_2_) as dark moss green powder in 83% yield.

### 2.4 Demetalation and Remetalation of *rac‐*PoGe[Zn, Zn]

With the final “Geländer” architecture in hand, we investigated removal/reinsertion of porphyrin metal centers. Using a four‐ to 20 fold excess of TFA in CH_2_Cl_2_ at room temperature followed by basic aqueous extraction resulted in incomplete removal of zinc(II) from ***rac‐*PoGe[Zn, Zn]**. Dissolving ***rac‐*PoGe[Zn, Zn]** in pure TFA used in large excess, followed by evaporation of TFA, washing with aq. NaHCO_3_ and GPC purification provided the freebase ***rac‐*PoGe[2H, 2H]** in 61% yield. Insertion of copper(II) ions was effected using Cu(OAc)_2_ in a THF/CH_2_Cl_2_/NEt_3_ ternary mixture at room temperature. In this manner, analytical quantities of ***rac‐*PoGe[Cu, Cu]** were isolated from excess Cu(OAc)_2_ by np HPLC (1% ethanol in CH_2_Cl_2_).

## 3 Structural Analysis

### 3.1 NMR Analysis and Assignment

**FIGURE 2:**
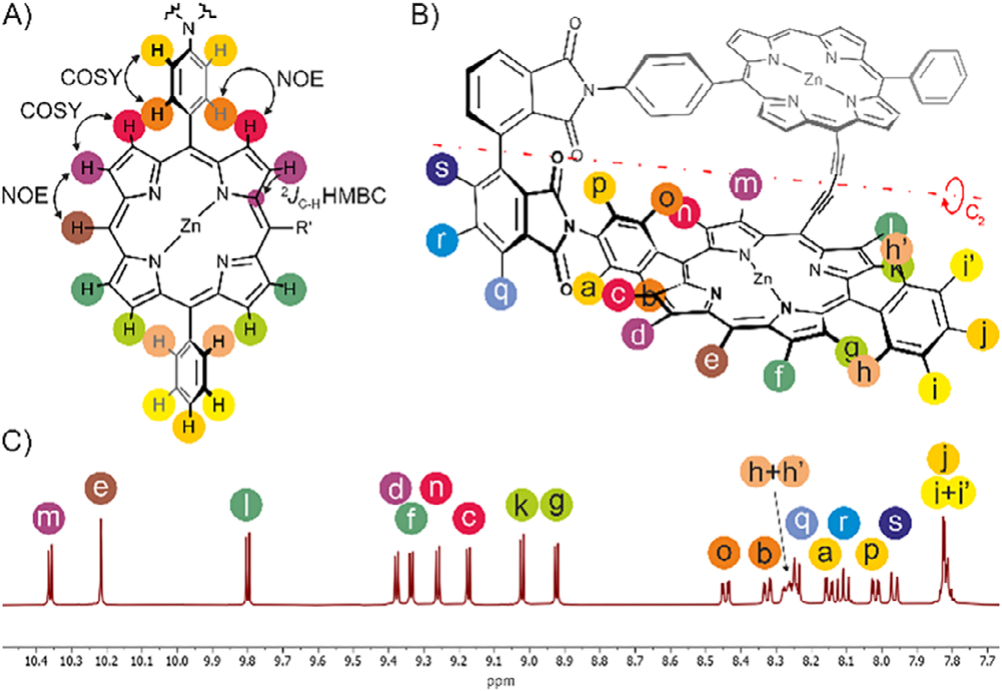
(A) Generalized porphyrin connectivity for compounds used in this study. The key couplings/NOE used for NMR assignment are highlighted between atoms with arrows. Porphyrin to phenyl NOE can be observed between orange and red protons, as well as between light brown and light green protons, while purple and dark green protons can be recognized by the lack of NOE couplings to phenyl protons. NOE coupling between purple/dark green protons (one of each) and the meso‐proton (dark brown) enables the distinction between protons of each color pair. (B) Perspective view on the C_2_‐symmetric chemical structure of **PoGe‐[Zn, Zn]**, with labeled hydrogen atoms for the assignment of (C) the ^1^H NMR spectrum of **PoGe‐[Zn, Zn]**. Distinct NOE pairs are protons o and c as well as b and n.

We observed the broadening of α‐ and β‐carbon signals in all freebase porphyrins that were soluble enough for ^13^C NMR spectroscopy of **4**, **4b**, **5,** and ***rac‐*PoGe[2H, 2H]**, which can be attributed to exchange effects stemming from the tautomerism of the pyrrolic N‐H protons [44]. In order to obtain unambiguous and clear NMR data for the assignment of the compounds, all freebase porphyrins in the pathway were converted to their zinc(II)‐porphyrinato complexes, if they were not already at hand. In addition to suppressing tautomerism for **4, 4b, 5,** and ***rac‐*PoGe[2H, 2H]**, leading to well‐resolved α‐ and β‐carbon resonances, zinc(II) introduction increased the solubility of **2**, **3,** and **3b** in pyridine‐d5 for ^13^C NMR analysis. In pure pyridine‐d5, however, the NMR spectra of the metalated porphyrins showed mostly broad features at concentrations suitable for ^13^C NMR analysis. We found that mild evaporation of these pyridine‐d5 solutions (interrupted when solidification was observed and without applying high‐vacuum) to form the pyridine‐d5 complexes, followed by re‐dissolution of thereby obtained residues in CDCl_3_ or THF‐d8 enabled the recording of clean ^1^H and ^13^C NMR spectra for full assignment. The porphyrin “Geländer” dimers exhibit C_2_ symmetry, every ^1^H NMR signal corresponds to at least one pair of homotopic protons in the molecules. The advantageous atom connectivity and throughspace arrangement in the 5,15‐diphenylporphyrin moiety simplifies NMR assignment considerably. This ultimately enabled the full assignment of all compounds in the pathway up to ***rac‐*PoGe[Zn, Zn]** (*See* Figure 2B,2C). In the 5,15‐diphenylporphyrin scaffold, the *para*‐substituted and the unsubstituted phenyl moiety can be easily differentiated by COSY and combined with the strong NOE between phenyl protons and neighboring β‐protons, this enabled to identify a set of two pairs of β‐protons adjacent to the phenyl moieties (*See* Figure 2A *, light green/pink proton set*). Their COSY correlation to neighboring β‐protons revealed the complementary set of two pairs of β‐protons distant from the rings (*See* Figure 2A *, dark green/purple proton set*), resulting in local C_2v_‐symmetric positional information about the porphyrin moiety. NOE contacts between the free *meso*‐proton and its neighboring β‐protons or HMBC correlations to carbons that are bound to these positions then break the formal C_2v_ symmetry and enable to differentiate along the *meso*‐H to acetylene axis. In this fashion, the ^1^H spectra of all compounds in the synthetic pathway could be assigned. Based on these assignments and by standard 2D‐NMR techniques, the ^13^C spectra could be assigned as well, leaving only the α‐carbons and the acetylene carbons of some compounds ambiguous. The ambiguity between the pairs of α‐carbons within a given pyrrole moiety could be resolved by recording HMBC spectra optimized for *J =* 5 Hz, which effectively suppressed ^3^ *J*(^1^H‐^13^C) couplings in the pyrrole elements and revealed the ^2^J(^1^H_β_‐^13^C_α_) couplings. Rotation about the phenyl rung axis is blocked, leading to four distinct and sharp proton signals for the rung protons. This is in contrast to a previously published design of our group that was characterized by free rotation of the rung when carbazoles were used as the banister chromophore [21]. The fact that for a given phenyl proton directed towards the porphyrin ring (Figure 2A *, protons shown in light/dark brown)*, there is only one NOE contact to a single porphyrin β‐proton observable furthermore indicates that the rungs lie in a fixed, C_2_‐symmetric orientation confined between the phthalimide and the porphyrin plane, which is in line with our calculations.

### 3.2 Geometry Optimization

**FIGURE 3:**
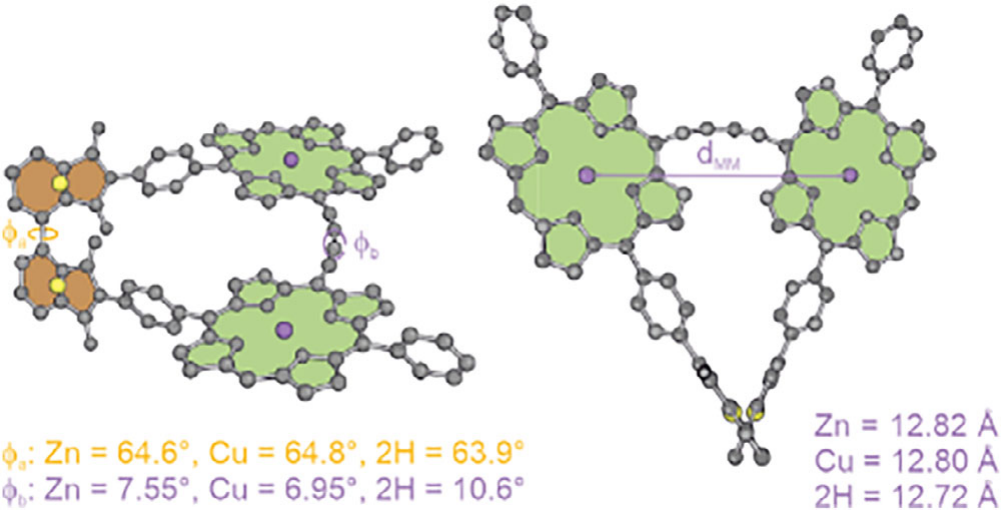
Optimized geometry of *(P)‐* **PoGe[Zn, Zn]** at the B3LYP/def2svp level of theory, on left a side view of the model is visualized, on the right a top view along the axis is visualized. In yellow the added centroids in the planes of the phthalimides (red) are shown. In green the centroids within the porphyrin plane (blue) are shown. Torsional angles ϕ_a_ around the axis and ϕ_b_ around the banister as well as the interporphyrin distance d_MM_ are indicated.

Despite our efforts, we could not yet grow a single crystal of either ***rac‐*PoGe[Zn, Zn]**, ***rac‐*PoGe[Cu, Cu],** or ***rac‐*PoGe[2H, 2H]** suitable for X‐ray spectroscopy. Thus, we turned to DFT calculation to gain further insights into the banister structures' spatial arrangement (see Supporting Information for graphical representations of our models). For that, we optimized the geometries at the B3LYP/def2svp level of theory. To describe the key geometric features, we defined 4 centroids–one in each phthalimide subunit and one in each porphyrin subunit (*Figure* 3), which was congruent with the Zn atoms in ***(P)‐*PoGe[Zn, Zn]** and the Cu atoms in ***(P)‐*PoGe[Cu, Cu]**. The interporphyrin distance d_MM_ does almost not change upon transmetalation and only a minute difference was found in the torsional angles between the phthalimide subunits and from the phthalimide to the porphyrin subunits. The torsion in the axis found is 63.9° for ***(P)‐*PoGe[2H, 2H]** and 64.6° and 64.8° for ***(P)‐*PoGe[Zn, Zn]** and ***(P)‐*PoGe[Cu, Cu]**, respectively. Another interesting torsion is observed along the banister defined by the planes spanned (Zn = 7.6°, Cu = 7.0°, and 2H = 10.6°) by the porphyrin subunits making the inner C‐C bond of the butadiyne linker a second stereogenic axis. The torsion around the banister is of particular interest as a study of electronic coupling and conjugation of similar dyads has shown the dependence to the torsional angle between the porphyrins [26]. The minute changes in the spatial arrangement upon metalation irrespective of the metal did not cause significant changes in our optical analysis and thus showed similar efficient electronic coupling between the porphyrin subunits.

### 3.3 Chiral Resolution

A particular structural feature of banister structures is their helicity, and all **PoGe** derivatives were synthesized as racemic mixtures. We submitted all banister structures to the chiral stationary phase (CSP) HPLC (*See* *Supporting Information* *for chromatograms*). With an analytical *IG Chiralpak* column, we were able to separate analytical amounts for ECD spectroscopy of all isomers in either 50% ethyl acetate in heptane (free base and M = Zn) or 70% ethyl acetate in heptane (M = Cu).

### 3.4 Optical Properties

#### 3.4.1 Absorption and Emission Spectra

**FIGURE 4:**
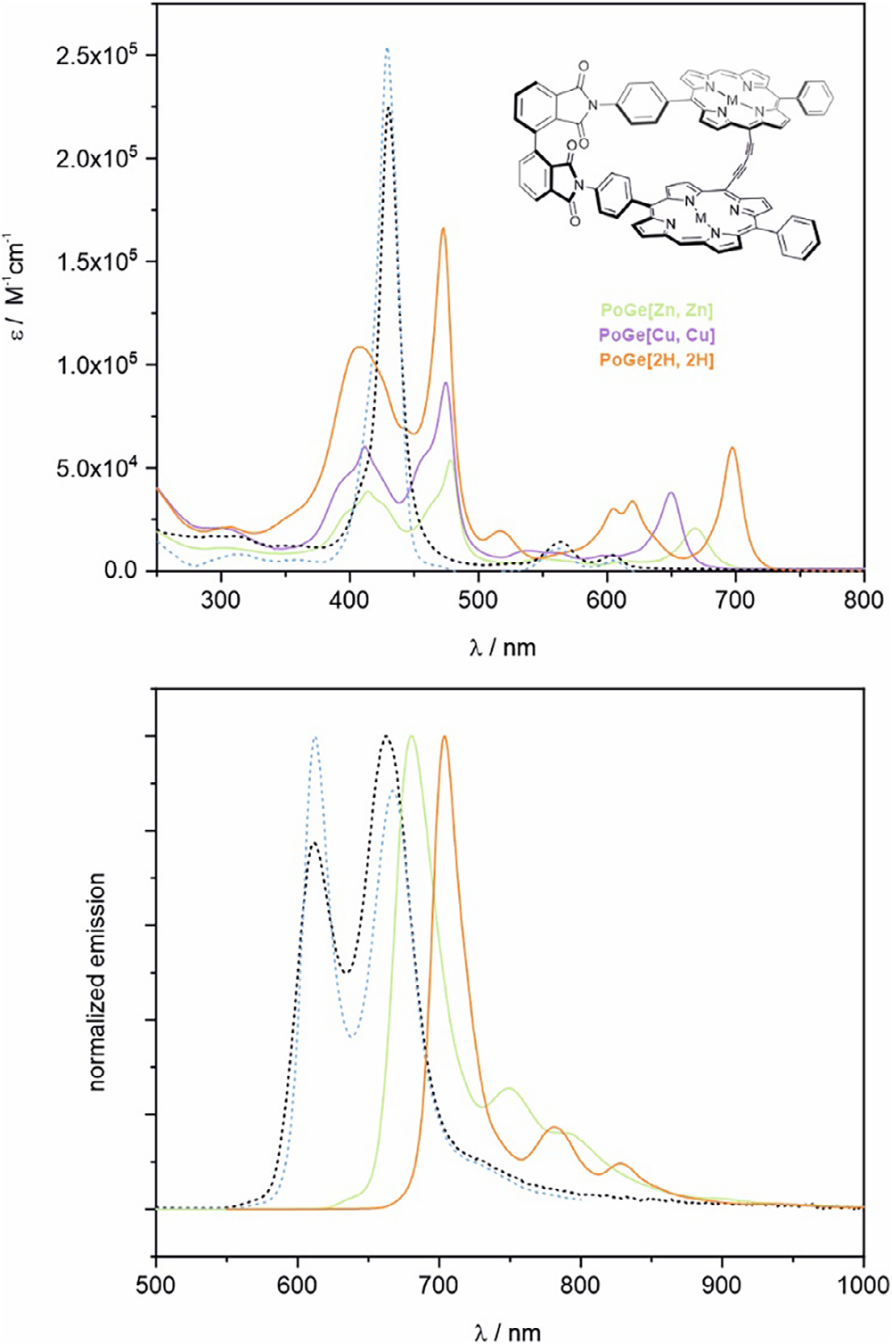
Abosrption (top) and emission (bottom) spectra of *rac‐* **P** **o** **Ge[Zn, Zn]** (green, solid line), *rac‐* **PoGe[Cu, Cu]** (purple, solid line, no emission), *rac‐* **PoGe[2H, 2H]** (orange, solid line), **7‐Zn** (blue, dotted line), and **4‐Zn** (black, dotted line); c = 10^−6^; chloroform.

**FIGURE 5:**
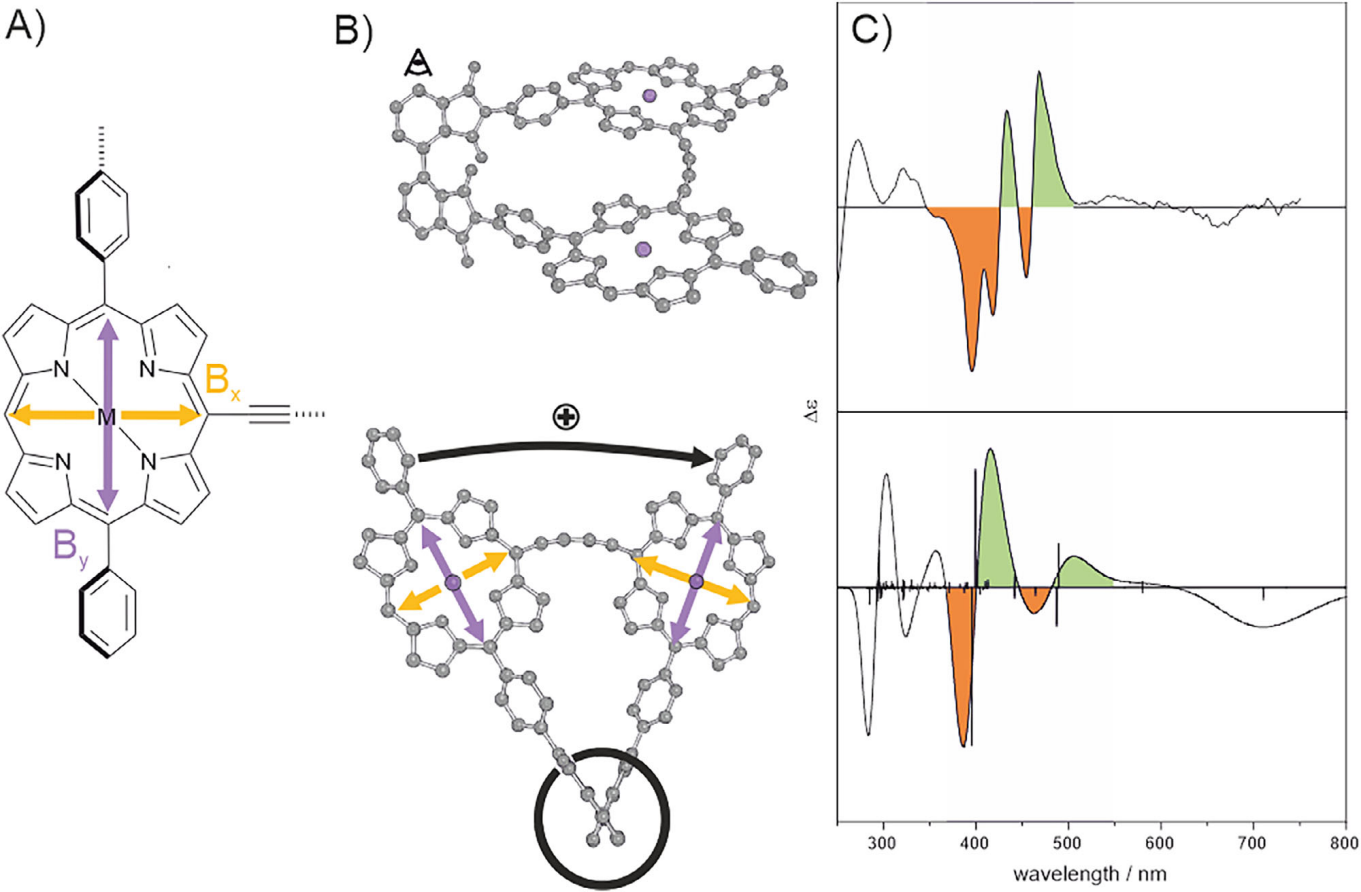
(A) The porphyrin subunit with indicated TDM as usually described in literature as B_x_ (yellow arrow) and B_y_ (purple arrow). (B) The geometry of *(P)‐* **PoGe[Zn, Zn]** in “staircase” representation a stylized eye indicates the axis for the *Newman‐*type projection. TDMs and rotation direction are indicated for the exciton coupling arising due to B_y_‐B_y_ interaction (purple). (C) The experimental (top) and simulated (bottom) ECD spectra of *(* *P* *)*‐**PoGe[Zn, Zn]**. Two bisignate signals are colored and indicate two positive couplets – from longer to shorter wavelengths, both signals are first positive (indicated by the green area) then negative (indicated by orange area). 200 vertical transitions were calculated at the TD‐LC‐PBE0/def2SV(p) level of theory and indicated as black sticks.

Absorption spectroscopy (*Figure* 4) of conjugated porphyrin systems demonstrates certain structural and electronic features directly by shift and shape of the B‐ (or *Soret*, roughly 400 to 500 nm) and Q‐bands (above 600 nm in the conjugated dyads) [27, 31, 32, 45]. For both investigated parent (nonlinked) compounds **4‐Zn** and **7‐Zn** we observed, as to be expected, an intensive sharp B‐band with little fine‐structure and weak signals for the Q‐bands [46], which is indicative that there is no apparent perturbation arising from the bis‐phthalimide axis present in **7‐Zn**. Upon *geländerfication* to ***rac‐*PoGe[Zn, Zn]**, the B‐ band split into a broader, weaker blue‐shifted signal and a relatively sharper, more intense red‐shifted signal. Electrochemical analysis by cyclic voltammetry revealed a reversible first oxidation and reduction process for **4‐Zn** ($\Delta \;E_{HOMO-LUMO}^{ec}=2.10\;eV)$, and a reversible first oxidation process for **7‐Zn (** $\Delta \;E_{\mathrm{HOMO}-\mathrm{LUMO}}^{\mathrm{ec}}=2.63\;\mathrm{eV})$. However, the reduction wave for **7‐Zn** was ill‐defined and the strained ***(rac)*‐PoGe[Zn, Zn]** $\left(\Delta \;E_{HOMO-LUMO}^{ec}=2.17\;eV\right)$ showed neither reversible oxidation nor reduction, likely due to increased structural reorganization and follow‐up chemical processes upon electron transfer. The metalated Geländer structures show similar B‐band fine‐structures, while the free base is less resolved, probably owing to the mobility of the NH protons and flexibility of the pyrrole moieties within the porphyrin framework. On the other hand, the Q‐region increased in intensity, and appears successively red‐shifted from M = Cu over M = Zn to M = 2H. The metalated structures show a single intensive peak in the Q‐region while in the freebase form, three intensive signals can be observed, of which the lowest energy band is most intensive. Similar spectral features have been previously seen in other conjugated porphyrin dyads and oligomers with coplanar orientation [26, 27, 31, 32, 34, 45, 47] Our scaffold (the axis and rung) forces the porphyrin subunits in an arrangement with 11 ° or less torsion along the butadiyne linker according to our optimized geometries discussed above. In multiple studies*, Anderson* and coworkers have demonstrated that the observed B‐band splitting behavior is owed to the torsion between two butadiyne‐linked porphyrins, where they ensured the conformation by variation of temperature, aggregation, or addition of ligands [26, 27, 28, 29]. Therefore, planarity and efficient conjugation of such a butadiyne‐linked porphyrin dyad leads to increased separation of the B‐band into a blue‐ and a red‐shifted region. The transition dipole moments (TDM) contributing to the B‐band of **PoGe** can be deconstructed as two TDMs along the banister (B_x_) and two along the rungs (B_y_) within the porphyrin moieties (see Figure 5A) [10, 27, 30, 48, 49] Describing the relative TDM orientation with *Kasha's* model for molecular excitons, B_x_ to B_x_ and B_y_ to B_y_ are arranged obliquely in **PoGe**s, leading to so‐called band splitting (into higher and lower energy signals compared to the isolated transition). Vector analysis [50] of the calculated TDM for transitions calculated for ***(P)‐*PoGe[Zn, Zn]** at the TD‐B3LYP/def2SV(p) level of theory also indicates that behavior for **PoGe** (See Supporting Information, Figures S91,S92). This exciton coupling induced band splitting is apparent in the UV‐Vis spectra of metalated **PoGe**s for both, the blue‐ and red region of their B‐bands (*Figure* 4), but stronger evidence for this effect is discussed in the following subchapter (ECD). The banister structures are significantly red‐shifted in their emission spectra compared to **4‐Zn** and **7‐Zn** (*Figure* 4). Upon de‐metalation of ***rac‐*PoGe[Zn, Zn]** (*λ*_em,max_ = 680 nm and Φ_F_ = 12%, τ = 1.2 ns (See Supporting Information, Figures S97–S100)) to ***rac‐*PoGe[2H, 2H]** (*λ*_em,max_ = 704 nm and Φ_F_ = 21%, τ = 2.8 ns (See Supporting Information, Figures S101–S104)), we observed a red shift, a significantly more intense signal and enhanced fluorescence lifetime. These results indicate that the enhanced quantum yield of ***rac‐*PoGe[2H, 2H]** results from slower nonradiative decay with respect to fluorescence (k_nr,[Zn, Zn]_/k_r,[Zn, Zn]_ > k_nr,[2H, 2H]_/k_r,[2H, 2H]_). ***rac‐*PoGe[2H, 2H]** also exhibits a smaller Stokes’ shift for the E_0‐0_‐transition than ***rac‐*PoGe[Zn, Zn]** (Δλ_[2H,2H]_ = 7 nm, Δλ_[Zn,Zn]_ = 31 nm), complementing the optical data and indicating smaller geometric and electronic relaxation of the excited state prior to emission. The incorporation of Cu(II) led to the quenching of the emission, so we could not observe any signal. These results show that we can precisely tune not only the far‐red absorption maxima of **PoGe**s but also their emission features by variation of the coordinated metal, entering the near‐IR region in the case of ***rac‐*PoGe[2H, 2H]** with emission bands up to 835 nm.

#### 3.4.2 Electronic Circular Dichroism

**FIGURE 6:**
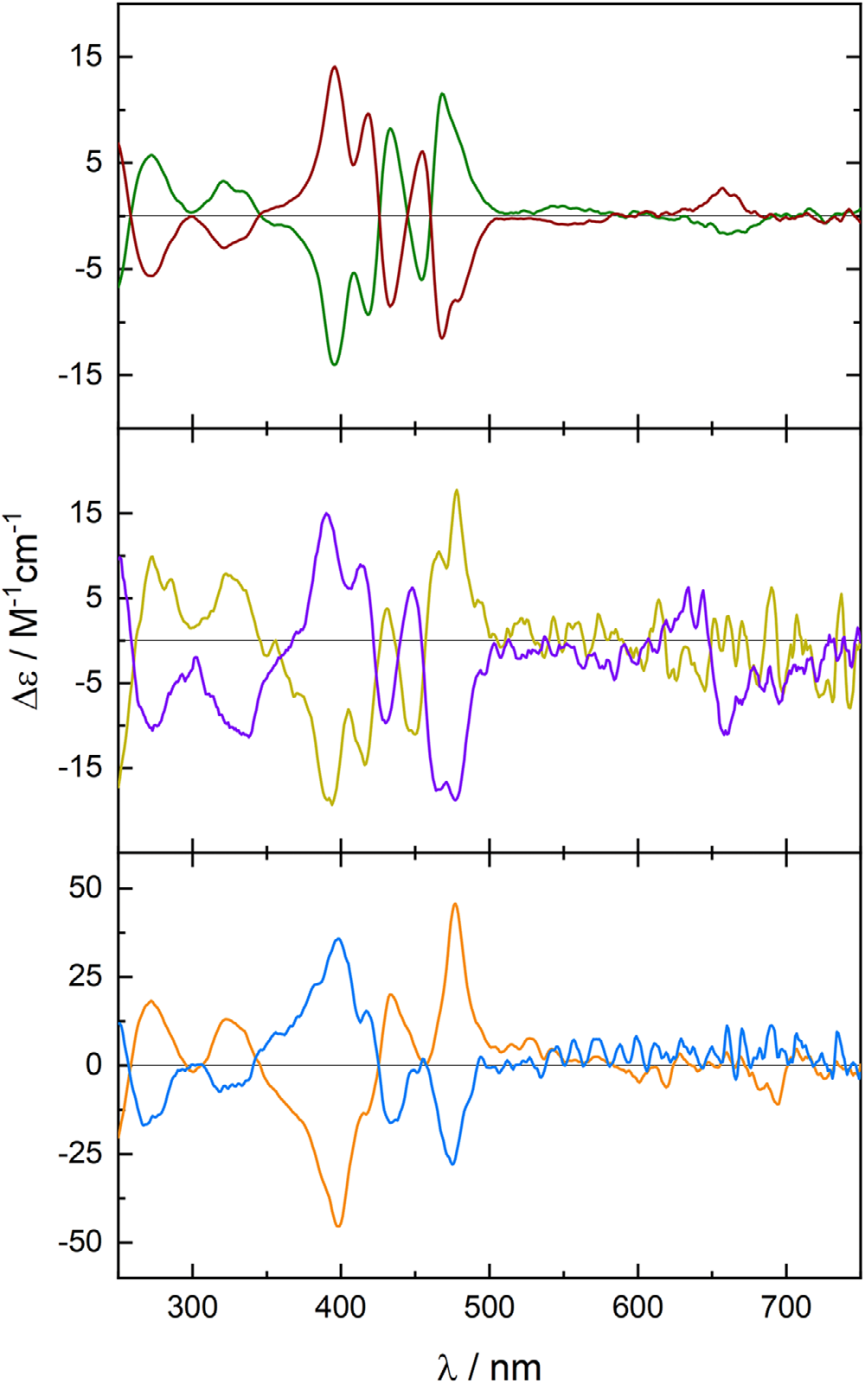
ECD spectra of *(P)‐* **P** **o** **Ge[Zn, Zn]** (green), *(M)‐* **PoGe[Zn, Zn]** (brown), *(P)‐* **PoGe[Cu, Cu]** (yellow), *(M)‐* **PoGe[Cu, Cu]** (purple), *(P)‐* **PoGe[2H, 2H]** (orange), and *(M)‐* **PoGe[2H, 2H]** (blue). The positive couplet between 400 and 450 nm is conserved in all structures. **PoGe[Zn, Zn]** c = 10^−5^, **PoGe[Cu, Cu]** and **PoGe[2H, 2H]** c = 10^−6^; chloroform.

We measured ECD (electronic circular dichroism) spectra of all *(P)* and *(M)* isomers **PoGe[2H, 2H]**, **PoGe[Zn, Zn]**, and **PoGe[Cu, Cu]** (*Figure* 6). We observed signs of opposition for the cotton bands in the ECD spectra in all cases. The most intense molar ellipticity was observed for **PoGe[2H, 2H]** within the B‐band. ECD signal for the Q‐band could only be observed for the *(P)* and *(M)* isomer of **PoGe[Zn, Zn]**. This can be easily rationalized from the higher molar absorption of ***rac*‐PoGe[2H, 2H]** compared to its metalated counterparts, and as the dissymmetry factor is in the same magnitude for all structures (g_abs_ ∼10^−4^), the increased molar ellipticity is owed to the higher molar absorption of **PoGe[2H, 2H]** than of **PoGe[Zn, Zn],** and **PoGe[Cu, Cu]**. Compared to porphyrin dyads in a helicene scaffold, this is a 10 fold lower [10]. An elegant method to assign the helicity of the enantiomers is the exciton chirality method (ECM), where bisignate ECD signals are analyzed, which arise from excitonic coupling [51, 52, 53, 54]. The signal is referred as a positive couplet when the red component has a positive ECD signal and negative for its blue part. *Vice versa*, a negative couplet has a negative ECD signal in its red component and a positive signal in its blue component. In the work of *Crassous* and coworkers, mentioned in the introduction, they studied the excitonic coupling of conjugated helicenes decorated with porphyrins, where ECM correctly predicts the stereo configuration [10, 55, 56]. First, we need to know the orientation of the TDM within the chromophores. We elaborated above that the B‐band is characterized by two TDM B_x_ and B_y_ (Figure 5A). To assign the configuration by ECM, the molecule needs to be projected appropriately. In C_2_‐symmetric helical molecules, this is simply along the correct stereogenic axis, which is (for B_y_ to B_y_) the bis‐phthalimide axis along z, also representing the crossing point for both B_y_ TDMs in the x/y plane. This leads to a visualization reminiscent of a *Newman* projection for banister structures (Figure 5B).^1^ A positive couplet is observed if the TDM rotate from the front to back clockwise, which can be observed for B_y_ in the molecular structure of ***(P)*‐PoGe[Zn, Zn]** (Figure 5B,5C). If they rotate anticlockwise, a negative couplet is observed. To fulfill the handedness of helical structures, the positive couplet must arise from a *(P)* helices and the negative from a *(M)* helices. The first eluting enantiomer shows for all **PoGe[M, M]** one to two positive couplets, which can be attributed to the discussed TDM (B_x_ and B_y_), thus, ECM predicts right‐handedness determining *(P)* configuration. The second eluted enantiomer, *vice versa*, showed negative couplets predicting *(M)* configuration. We observed other banister structures before which under the same resolution conditions (same CSP and eluent), with only minute differences resolving in the same order [18, 21]. To corroborate the assignment by ECM, we turned to TD‐DFT calculation at the LC‐PBE0/def2SV(p) [10, 57] and the B3LYP/def2SV(p) level of theory with PCM model for chloroform. We calculated for all here in presented ***(P)‐*PoGe[M, M]** a negative signal for the lowest energy transition. We could only detect this unambiguously for ***(P)‐*PoGe[Zn, Zn]** in the experimental spectrum. In both ***(P)‐*PoGe[Zn, Zn]** and ***(P)‐*PoGe[Cu, Cu]** the simulated spectrum shows two positive couplets in the B‐band while in ***(P)‐*PoGe[2H, 2H]** only one can be clearly identified, matching the experimental spectra in all cases. The intensity of the simulated spectrum seems to underestimate the intensity of the lower energy part in the B‐band (450‐500 nm) relative to the lowest energy transition and the rest of the B‐band. The experimental and simulated ECD and absorption spectra fit well for all three ***(P)*‐PoGe[M, M**] and confirmed the prediction by ECM. In the TD‐B3LYP/def2SV(p) of ***(P)‐*PoGe[Zn, Zn]**, four computed transitions were identified as being responsible for the bisignate signals observed in the experimental spectrum. Vector analysis of the corresponding TDMs revealed polarization on the porphyrin subunits along B_y_ (Figure S92) supporting the assignment by ECM.

## 4 Conclusions

New “Geländer” macrocycles **PoGe** were designed and synthesized according to a design principle following orthogonal joints between the axis, rung, and banister. CSP‐HPLC resolved all ***rac‐*PoGe[M, M]** into their *(P)* and *(M)* isomers. The structural and optical properties of **PoGe**s were studied by NMR, UV‐Vis, fluorescence, and ECD spectroscopy, and the obtained results align well with our computational studies. The rational design of a 90° ABCD‐type aminophenyl‐alkynylporphyrin and a preformed stereogenic phthalimide axis enabled the efficient condensation of the open‐chained precursor **7‐Zn**. Thus, synthon **6** allows the rapid introduction of a multitude of orthogonal chromophores into Geländer structures. Macrocyclization was achieved by oxidative alkyne coupling and post‐synthesis modification and fine‐tuning of the absorption and emission spectra was demonstrated by removal and reintroduction of the central metal of ***rac‐*PoGe[Zn, Zn]** to ***rac‐*PoGe[Cu, Cu]** *via* ***rac‐*PoGe[2H, 2H]**. Computational modeling and optical analysis revealed a low torsional rotation angle along the banister, which led to unique spectral features matching well with related literature systems. The well‐established knowledge of the spectral qualities of porphyrins allowed the assignment of the absolute configuration of the **PoGe** structures by ECM, which was corroborated by simulation of the ECD spectra. The reported structures herein show that a Geländer scaffold renders the features of a conjugated porphyrin dyad chiral and represents a further example of *ortho*‐bisphthalimide derived Geländer structures, further hinting to the universal applicability of this design approach and contributing to forming the identity of this unique class of helically chiral structures. Furthermore, the minutely tunable absorption and emission spectra of **PoGe**s reach the near‐IR region and show bright fluorescence emission due to inherently effective internal conversion in porphyrins. Thus, the herein presented molecules could be readily expanded in the future to move their optical spectra further to the red, while keeping their tunability by variation of the metal centers. This idea may be explored by simple extension of the porphyrin pi‐systems or by synthesizing tri‐ and higher oligomers, introducing the already at hand **4b‐Zn** as a central banister‐elongating motif. **PoGe** are presented as model systems adding chirality as a further intriguing characteristic for modern optical applications to porphyrin dyads, which are already known to combine near‐IR absorption/emission, strong non‐linear optics (NLO) properties and easy processability due to their all‐organic nature, making these materials interesting candidates for the development of functional materials. Efforts to further tune and investigate the optical features of **PoGe** are underway in our laboratories.

## Author Contributions

D'A, J. F. K., and M.M conceived the project idea and developed the molecular design in a joint effort. J. F. K. synthesized all compounds except **6** and ***rac‐*PoGe[Zn, Zn]**. A. D'A synthesized **6** and ***rac‐*PoGe[Zn, Zn]**. J. F. K. characterized all compounds and did all work related to NMR spectroscopy. A. D'A did all work related to HPLC chromatography, optical spectroscopy, and DFT calculations. A. D'A and J. F. K. analyzed and discussed all data. M. M. supervised the project. All authors contributed in writing this manuscript.

## Conflicts of Interest

The authors declare no conflict of interest.

## Supporting information



The authors have cited additional references within the Supporting Information [43, 50, 55, 56, 57, 58, 59, 60, 61, 62, 63, 64].

## Data Availability

The data that support the findings of this study are available in the supplementary material of this article.

## Endnote

1 As the (phthalimide) axis and banister are entangled stereogenic axes, for B_x_ to B_x_ the projection needs to be changed so that the inner C‐C bond of the butadiyne is the rotation axis in the Newman‐type projection. In that case, clockwise rotation is still observed for the *(P)* isomers.
