# Supplementary material for: Porphyrin‐Geländer–Helical Conjugated Banister Type Porphyrin Dyads
Source: Chemistry. 2026 Feb 6;32(15):e03049. doi: 10.1002/chem.202503049 (PMC13107505; doi:10.1002/chem.202503049)
Supplement: Supplementary file 1 — The authors have cited additional references within the Supporting Information [43, 50, 55, 56, 57, 58, 59, 60, 61, 62, 63, 64]. [file CHEM-32-e03049-s001.pdf]

Supporting Information; Porphyrin-Geländer - Helical Conjugated Banister Type Porphyrin Dyads

Joël F. Keller,‡[a] Adriano D'Addio,‡[a] and Marcel Mayor *[abc]

‡ These author contributed equally to this work.

[a] A. D'Addio, J. F. Keller, Prof. Dr. M. Mayor
Department of Chemistry
University of Basel
St. Johanns-Ring 19, 4056 Basel (Switzerland)
E-mail: marcel.mayor@unibas.ch

[b] Prof. Dr. M. Mayor
Institute for Nanotechnology (INT)
Karlsruhe Institute of Technology (KIT)
P. O. Box 3640, 76021Karlsruhe (Germany)

[c] Prof. Dr. M. Mayor
Lehn Institute of Functional Materials(LIFM)
School of Chemistry
Sun Yat-Sen University (SYSU)
Guangzhou 510275 (P.R. of China)

Contents

General Remarks 2

Synthetic Procedures and Characterization Data 3

NMR Spectra 15

Fluorescence Lifetime Measurements 108

Cyclic Voltammetry 112

HPLC Chromatography 96

Np-HPLC purification 96

Chiral Resolution 97

ECD Spectroscopy 100

Dissymmetry Plot 100

Computational Details and Geometric Analysis 100

Geometry Optimization 100

TD-DFT 102

TDM Vector Analysis 103

Determination of Torsions 106

Cartesians 114

References 119

General Remarks

Deuterated solvents were obtained from Cambridge Isotope Laboratories, Inc. (Andover, MA, USA). All commercially available compounds were purchased from Sigma-Aldrich (Switzerland), Acros, Apollo Scientific, Alfa Aesar, and Fluorochem (United Kingdom). Column chromatography was performed on silica gel P60 (40-63 µm) from Silicycle^{TM}, and the solvents were HPLC grade. TLC was performed with silica gel 60 F254 aluminum plates purchased from Merck. Analytics and instruments: NMR experiments were performed on Bruker Avance III NMR spectrometers operating at 400 or 500 MHz proton frequencies. The instruments were equipped with a direct-observe 5 mm BBFO smart probe (400 MHz) or an indirect-detection 5 mm BBI probe (500 MHz). All probes were equipped with actively shielded z-gradients (10 A). The chemical shifts are reported in ppm relative to tetramethylsilane or referenced to the residual solvent peak, and the J values are given in Hz (±0.1 Hz). Standard Bruker pulse sequences were used, and the data was processed on Topspin 3.2 (Bruker) using twofold zero-filling in the indirect dimension. UV-Vis absorption spectra were recorded at 20 °C on a Jasco V-770 Spectrophotometer. Fluorescence spectra measurements were performed on a Jasco FP-8600. CD measurements were performed on a JASCO J-1500 CD Spectrophotometer. Quantum yields were measured with Hamamatsu Quantaurus-QY, all compounds were measured three times, and the average was reported. The UV/Vis, CD, and fluorescence spectra were measured in 1 cm quartz glass cuvettes. High-pressure liquid chromatography (HPLC) and gel permeation chromatography (GPC) were performed on a Shimadzu Prominence System. For high-resolution mass spectrometry (HRMS), an HR-ESI-ToF-MS measurement was performed on a maXisTM 4G instrument from Bruker. Zn dust was activated prior to use by stirring in 0.1 M aq. HCl for several minutes, followed by washing the powder in this order with distilled water, ethanol, and diethyl ether and drying it in vacuo. The used column for analytical separation on chiral stationary phase was a Chiralpak IG, 5 µm, 4.6×250 mm, normal phase HPLC was performed on either analytical (4.6×250 mm) or semi-preparative (20×250 mm) reprosil columns by Dr. Maisch. GPC) was performed with a Shimadzu Prominence System equipped with SDV preparative columns from Polymer Standards Service (two Shodex columns in series, 20×600 mm each, exclusion limit: 30 000 g mol−1) with chloroform as solvent. Description of J = 5 Hz experiments.

Synthetic Procedures and Characterization Data

Compound **1** and **6** were prepared according to literature procedures.[1,2]

5-(4-nitrophenyl)-15-phenylporphyrin **(2)**

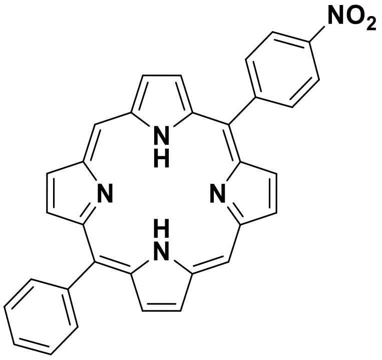

Under an argon atmosphere, a solution of di(1H-pyrrol-2-yl)methane (**1**) (2.860 g, 19.56 mmol, 2.00 eq.), benzaldehyde (749 mg, 7.06 mmol, 0.72 eq.) and 4-nitrobenzaldehyde (1.906 g, 12.61 mmol, 1.29 eq.) in CH_2Cl_2 (2.5 L) was degassed with a continuous, vigorous stream of argon for 2 hours, and TFA (1.43 mL, 19.05 mmol, 1.95 eq.) was added under the absence of light. The solution was stirred in the dark for 18 hours at room temperature. DDQ (6.674 g, 29.4 mmol, 3.00 eq.) was added in one portion, and stirring continued for 1 h at room temperature, and the solvent volume was reduced to 1.5 L. The mixture was basified with NEt_3 (3.21 mL, 22.8 mmol, 2.33 eq.) and filtered carefully through a pad of SiO_2 (350 g), eluting with CH_2Cl_2. Due to the low solubility of the compound, the volume of the solution was reduced to 80 mL by repeated evaporation of portions in a 500 mL round-bottom-flask, to minimize the formation of an insoluble porphyrin film on the glass surface, and transferred to a 1 L Erlenmeyer flask. MeOH (720 mL) was added, and the suspension was left to stand at room temperature for 30 minutes to complete precipitation. The suspension was filtered with a moderate pace by suction to maintain the suspension temperature close to room temperature. The filtrate was evaporated to yield 5,15-diphenylporphyrin **(9)** (135 mg, 0.29 mmol, 3%). The collected solids were washed with several portions of MeOH, and dried *in vacuo* to give the crude product as a mixture of 5-(4-nitrophenyl)-15-phenylporphyrin (**2**) and 5,15-bis(4-nitrophenyl)porphyrin (**10**), containing small amounts of 5,15-diphenylporphyrin (**9**). The crude product was redissolved in $CHCl_3$, mixed with SiO_2 (35 mL), and evaporated to dryness.

Flash column chromatography with SiO_2 (250 mL) and cyclohexane/DCM 1:1 to 0:1 eluted first diphenylporphyrin (**9**, purple band, 16 mg, 34.6 micromol, 0.4%), followed by 5-(4-nitrophenyl)-15-phenylporphyrin (**2,** red band, 830 mg, 17%) and 5,15-bis(4-nitrophenyl)porphyrin (**10,** red band, not isolated).

Compound **9**: The analytical data matched with the literature.[3] Compound **2:** ^{1}**H NMR** (400 MHz, $CDCl_3$) δ = 10.37 (s, 2H), 9.45 (d, J = 4.6 Hz, 2H), 9.42 (d, J = 4.6 Hz, 2H), 9.11 (d, J = 4.5 Hz, 2H), 9.00 (d, J = 4.6 Hz, 2H), 8.70 (d, J = 7.8 Hz, 2H), 8.47 (d, J = 7.9 Hz, 2H), 8.28 (m, 2H), 7.83 (m, 3H), -3.12 (s, 2H) ppm. ^{13}**C NMR** The solubility of this compound was too low for ^{13}C NMR. **HR-MS** (ESI-ToF, +): m/z = [*M*+H]$^+$ Calcd. For $C_{32}H_{22}N_5O_2$: 508.1768; Found 508.1775.

[5-(4-nitrophenyl)-15-phenyl-porphyrinato]zinc(II)(pyridine) **(2-Zn-pyr)**

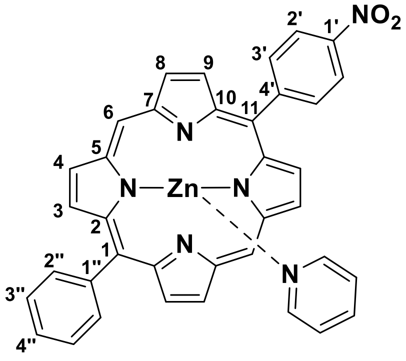

To increase the solubility for analytical purposes, **2** (21.0 mg, 41.4 micromol, 1.00 eq.) was dissolved in $CHCl_3$/MeOH (9:1, 5 mL) and $Zn(OAc)_2$ (380 mg, 2.07 mmol, 50 eq.) was added. The suspension was refluxed at 65 °C for 1 h 30 minutes upon complete dissolution of the material and filtered through a small pad of silica eluting with a mixture of $CHCl_3$/pyridine (10:1). The filtrate was washed with H_2O (5x), and the aqueous layer re-extracted with $CHCl_3$ (3x), the combined organic layers dried over Na_2SO_4, filtered, and the solvent evaporated to yield a red solid (29 mg), which was subjected to gel permeation chromatography in $CHCl_3$ and **2-Zn-pyr** was isolated as a red solid (24 mg, 41.4 µmol, 89%).

^{1}H NMR (400 MHz, $CDCl_3$) δ = 10.26 (s, 2H, C6-**H**), 9.42 (d, *J* = 4.5 Hz, 2H, C8-**H**), 9.39 (d, *J* = 4.4 Hz, 2H, C4-**H**), 9.09 (d, *J* = 4.4 Hz, 2H, C3-**H**), 8.98 (d, *J* = 4.4 Hz, 2H, C9-**H**), 8.68 – 8.63 (m, 2H, C2'-**H**), 8.47 – 8.42 (m, 2H, C3'-**H**), 8.28 – 8.23 (m, 2H, C2''-**H**), 7.81 – 7.75 (m, 3H, C3''-**H**, C4''-**H**), 7.10 – 6.88 (m, pyr, 1H), 6.70 – 6.31 (m, pyr, 2H) ppm. (Only two of three expected pyridine signals are visible (broad, and shifted upfield). The third signal is likely located upfield of 6 ppm and too broad to be visible, most likely due to the systems specific exchange kinetics.) **^{13}C NMR** (126 MHz, $CDCl_3$) δ = 150.83 (**C4'**), 150.33 (**C2**), 149.85 (**C5**), 149.55 (**C7**), 149.08 (**C10**), 147.44 (**C1'**), 143.22 (**C1''**), 135.57 (**C3'**), 134.94 (**C2''**), 132.68 (**C3**), 132.25 (**C8**), 131.81 (**C4**), 131.33 (**C9**), 127.43 (**C4''**), 126.59 (**C3''**), 121.65 (**C2'**), 120.52 (**C1**), 116.14 (**C11**), 106.34 (**C6**), ppm. **HR-MS** (for **2-Zn**) (ESI-ToF, +): m/z = $[M+H]^+$ Calcd. For $C_{32}H_{20}N_5O_2Zn$: 569.0825; Found 569.0815.

Bromination of **2**: 10-bromo-15-(4-nitrophenyl)-5-phenylporphyrin (**3**), 10,20-dibromo-15-(4-nitrophenyl)-5-phenylporphyrin (**3b**)

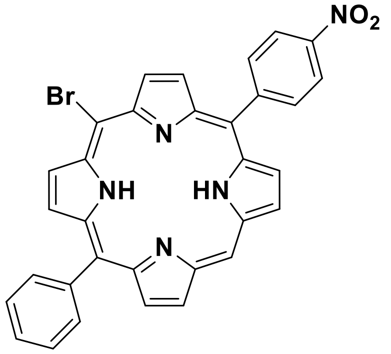

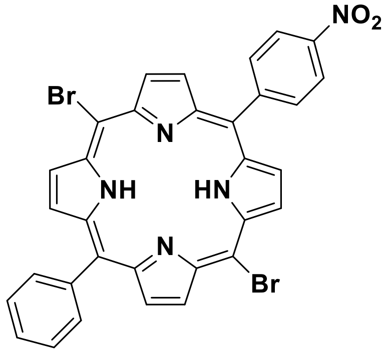

2 (100 mg, 197 micromol, 1.00 equiv.) was suspended in $CHCl_3$ (18 mL, stab. EtOH) and refluxed until complete dissolution of the material. The solution was allowed to cool to toom temperature and pyridine (0.45 mL, 5.59 mmol, 28.3 eq.) was added. The solution was sparged with argon gas for 10 minutes, and a solution of NBS (46.4 mg, 258 micromol, 1.31 eq.) in $CHCl_3$ (0.5

mL) was added dropwise at room temperature. The reaction mixture was stirred for 2 h at room temperature, and acetone (4 mL) was added. The solvent was evaporated *in vacuo* and the residue was subjected to flash column chromatography (SiO_2) with cyclohexane/CH_2Cl_2 (4:6 → 7:4) first eluting 10,20-dibromo-15-(4-nitrophenyl)-5-phenylporphyrin (**3b**, red band, 23.0 mg, 34.6 micromol, 18%) , followed by 10-bromo-15-(4-nitrophenyl)-5-phenylporphyrin (**3**, red band, 39.1 mg, 66.7 micromol, 34%).

Compound 3: ^{1}**H NMR** (400 MHz, $CDCl_3$) δ = 10.22 (s, 1H), 9.79 (d, *J* = 4.9 Hz, 1H), 9.76 (d, *J* = 4.8 Hz, 1H), 9.34 (d, *J* = 4.7 Hz, 1H), 9.31 (d, *J* = 4.7 Hz, 1H), 8.98 (dd, *J* = 6.4, 4.7 Hz, 2H), 8.86 (d, *J* = 4.7 Hz, 1H), 8.85 (d, *J* = 4.9 Hz, 1H), 8.70 – 8.65 (m, 2H), 8.42 – 8.38 (m, 2H), 8.25 – 8.20 (m, 2H), 7.87 – 7.75 (m, 3H), -3.01 (s, 2H) ppm. ^{13}**C NMR** The solubility of this compound was too low for ^{13}C NMR. **HR-MS** (ESI-ToF, +): m/z = [*M*+H]$^+$ Calcd. For $C_{32}H_{21}BrN_5O_2$: 586.0873; Found 586.0865.
Compound 3b: ^{1}**H NMR** (400 MHz, $CDCl_3$) δ = 9.59 (d, *J* = 4.9 Hz, 2H), 9.56 (d, *J* = 4.9 Hz, 2H), 8.79 (d, *J* = 4.8 Hz, 2H), 8.66 (d, *J* = 4.7 Hz, 2H), 8.63 – 8.56 (m, 2H), 8.32 – 8.24 (m, 2H), 8.12 – 8.06 (m, 2H), 7.80 – 7.67 (m, 3H), -2.80 (s, 2H) ppm. ^{13}**C NMR** The solubility of this compound was too low for ^{13}C NMR. **HRMS** (ESI-ToF, +): m/z = [*M*+H]$^+$ Calcd. For $C_{32}H_{20}Br_2N_5O_2$: 663.9978; Found 663.9966.

[5-(4-nitrophenyl)-15-phenyl-10-bromoporphyrinato]zinc(II)(pyridine-d5) **(3-Zn-pyr-d5)**

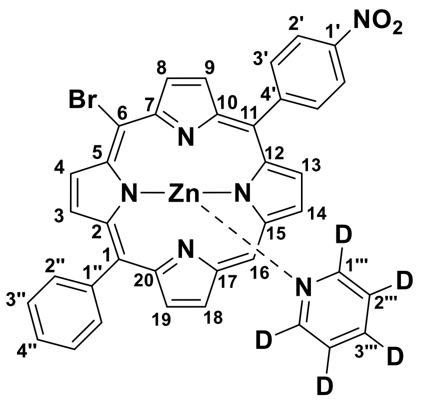

3 (22.5 mg, 38.4 micromol, 1.00 eq.) was dissolved in CH_2Cl_2/MeOH 10:1 (6 mL) and $Zn(OAc)_2$ (11.3 mg, 61.4 micromol, 1.6 eq.) was added. The reaction mixture was heated to 40 °C and stirred for 4 h. The reaction mixture was diluted with CH_2Cl_2 (10 mL) and washed with H_2O (3 x 2 mL), the aqueous layer re-extracted with CH_2Cl_2 (1 x 3 mL). The combined organic layers were dried over Na_2SO_4, filtered, and the solvent was evaporated to give a red solid.

Flash column chromatography (SiO_2) with cyclohexane/CH_2Cl_2 (1:1) provided **3-Zn** as a red solid (24.4 mg, 38.4 micromol, 98%).

To increase the solubility for full NMR characterization, **3-Zn** was dissolved in pyridine-d5 (0.5 mL), and evaporated at 40 °C/20 mbar for 15 minutes to give **3-Zn-pyrd5**, which was then characterized in $CDCl_3$.

Compound **3-Zn-pyrd5**: ^{1}**H NMR** (400 MHz, $CDCl_3$) δ = 10.17 (s, C16-**H**, 1H), 9.80 (d, J = 4.6 Hz, C8-**H**, 1H), 9.77 (d, J = 4.7 Hz, C4-**H**, 1H), 9.35 (d, J = 4.5 Hz, C14-**H**, 1H), 9.32 (d, J = 4.5 Hz, C18-**H**, 1H), 9.01 (d, J = 4.4 Hz, C19-**H**, 1H), 8.99 (d, J = 4.6 Hz, C3-**H**, 1H), 8.89 (d, J = 4.5 Hz, C13-**H**, 1H), 8.76 (d, J = 4.5 Hz, C9-**H**, 1H), 8.68 – 8.62 (m, C2'-**H**, 2H), 8.41 – 8.37 (m, C3'-**H**, 2H), 8.23 – 8.18 (m, C2''-**H**, 2H), 7.82 – 7.73 (m, C3''-**H**, C4''-**H**, 3H) ppm. ^{13}**C NMR** (126 MHz, $CDCl_3$) δ = 150.78 (**C_q**), 150.69 (**C_q**), 150.66 (**C_q**), 150.40 (C4'), 150.37 (**C_q**), 149.75 (**C_q**), 149.47 (**C_q**), 149.44 (**C_q**), 149.42 (**C_q**), 147.53 (**C1'**), 146.75 (**C1'''**), 146.53 (**C1'''**), 146.31 (**C1'''**), 142.90 (**C1''**), 135.75 (**C3'''**), 135.52 (**C3'''**), 135.41, 135.34 (**C3'''**), 134.81 (**C2''**), 133.31

(**C8**), 133.30 (**C3**), 133.14 (**C19**), 132.89 (**C4**), 132.64 (**C14**), 132.18 (**C18**), 131.94 (**C9**), 131.78 (**C13**), 127.59 (**C4''**), 126.59 (**C3''**), 122.86 (**C2'''**), 122.66 (**C2'''**), 122.46 (**C2'''**), 121.68 (**C1''**), 121.65 (**C2'**), 117.25 (**C11**), 106.73 (**C16**), 105.00 (**C6**) ppm. **HR-MS** (ESI-ToF, +): m/z = [*M*+H]$^+$ Calcd. For $C_{37}H_{19}BrD_5N_6O_2Zn$: 732.0744; Found 732.0742.

[5-(4-nitrophenyl)-15-phenyl-10,20-dibromoporphyrinato]zinc(II)(pyridine) **(3b-Zn-pyr)**

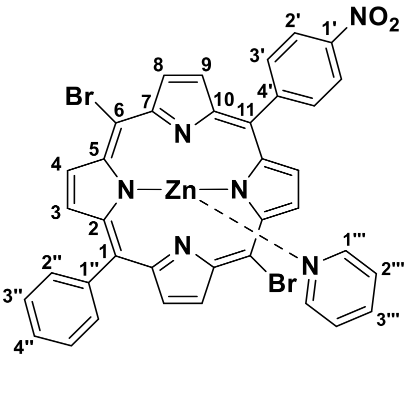

3b (25.0 mg, 37.6 micromol, 1.00 eq.) was dissolved in CH_2Cl_2/MeOH/pyridine (8:1:1, 10 mL) and $Zn(OAc)_2$ (13 mg, 70.9 micromol, 1.89 eq.) was added. The reaction mixture was heated to 30 °C and stirred for 24 h. The reaction mixture was diluted with CH_2Cl_2 (5mL) and washed with H_2O (3 x 2 mL) and the aqueous layer re-extracted with CH_2Cl_2 (2 x 2 mL). The combined organic layers were dried over Na_2SO_4, and filtered. To the filtrate, SiO_2 (500 mg) was added and the suspension was carefully evaporated to dryness.

Flash column chromatography (SiO_2) with cyclohexane/CH_2Cl_2 (2:8 → 1:1 → 8:2) provided **3b-Zn** as a red solid (26.5 mg, 36.4 micromol, 97%).

To increase the solubility for full NMR characterization, **3b-Zn** was dissolved in pyridine (0.5 mL), and evaporated at 40 °C/20 mbar for 15 minutes to give **3b-Zn-pyr**, which was then characterized in $CDCl_3$.

^{1}H NMR (500 MHz, $CDCl_3$) δ = 9.69 (d, J = 4.6 Hz, C8-**H**, 2H), 9.66 (d, J = 4.7 Hz, C4-**H**, 2H), 8.87 (d, J = 4.6 Hz, C3-**H**, 2H), 8.75 (d, J = 4.6 Hz, C9-**H**, 2H), 8.63 – 8.60 (m, C2'-**H**, 2H), 8.34 – 8.30 (m, C3'-**H**, 2H), 8.15 – 8.11 (m, C2''-**H**, 2H), 7.81 – 7.70 (m, C3''-**H**, C4''-**H**, 3H), 7.30 (tt, J = 7.6, 1.8 Hz, C3'''-**H**, 1H), 6.90 – 6.81 (m, C1'''-**H**, 2H), 6.78 (ddd, J = 7.7, 4.5, 1.4 Hz, C2'''-**H**, 2H) ppm. **^{13}C NMR** (126 MHz, $CDCl_3$) δ = 151.10 (**C2**), 150.61 (**C5**), 150.30 (**C7**), 150.02 (**C4'**), 149.81 (**C10**), 148.10 (**C1'''**), 147.67 (**C1'**), 142.60 (**C1''**), 136.15 (**C3'''**), 135.30 (**C3'**), 134.71 (**C2''**), 133.76 (**C3**), 133.70 (**C8**), 133.25 (**C4**), 132.41 (**C9**), 127.78 (**C4''**), 126.62 (**C3''**), 123.54 (**C2'''**), 122.89 (**C1**), 121.70 (**C2'**), 118.40 (**C11**), 105.37 (**C6**) ppm. **HR-MS** (for **3b-Zn**) (ESI-ToF, +): m/z = [*M*+H]$^+$ Calcd. For $C_{32}H_{18}Br_2N_5O_2Zn$: 725.9113; Found 725.9100.

Direct synthesis of 15-(4-nitrophenyl)-5-phenyl-10-((triisopropylsilyl)ethynyl)porphyrin (**4**) and 5-(4-nitrophenyl)-15-phenyl-10,20-bis((triisopropylsilyl)ethynyl)porphyrin (**4b**) from 5-(4-nitrophenyl)-15-phenylporphyrin (**2**).

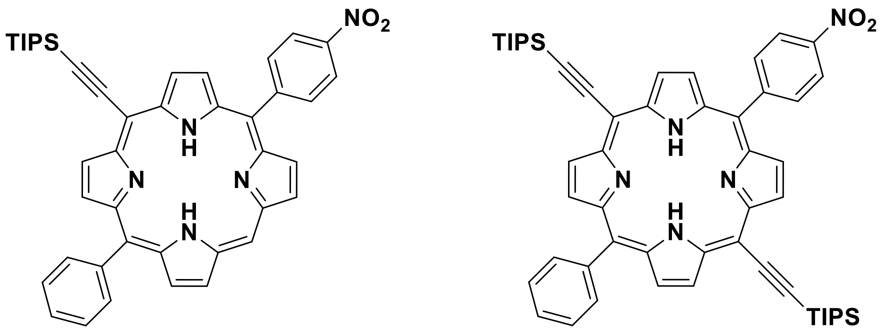

2 (512 mg, 1.01 mmol, 1.00 eq.) was dissolved in $CHCl_3$ (1.2 L) and degassed for one hour with a vigorous stream of argon gas. A solution of *N*-bromosuccinimide (180 mg, 1.00 mmol, 0.99 eq.) in $CHCl_3$ (150 mL) was degassed with argon and added dropwise over the course of 40 minutes, and stirring was continued at room temperature for 40 minutes. The reaction was quenched by the addition of acetone (25 mL), the solvent was evaporated and the residual pyridine removed under high-vacuum. The residue was redissolved in CH_2Cl_2 (150 mL) and MeOH (15 mL) and $Zn(OAc)_2$ (752 mg, 4.10 mmol, 4.06 eq.) were added and stirred at room temperature for 2 hours. The organic layer was washed with H_2O (3x), the organic layer extracted with CH_2Cl_2 (3x), dried over Na_2SO_4, filtered and evaporated to give the *meso*-bromoporphyrin mixture as a purple solid.

The *meso*-bromoporphyrin mixture was dissolved in THF (50 mL), transferred to a 100 mL Schlenk-tube and NEt_3 (15 mL) and TIPS-acetylene (1.667 g, 9.14 mmol, 9.05 eq.) were added. The solution was degassed, and $Pd(PPh_3)_2Cl_2$ (71.6 mg, 101 micromol, 10 mol%) and CuI (38.0 mg, 199 micromol, 19.7 mol%) were added. The Schlenk-tube was sealed and stirred for 14 hours at 50 °C. The solvent was evaporated, and the residue redissolved in CH_2Cl_2, washed with H_2O (3x), and the aqueous layer extracted with CH_2Cl_2 (3x). The combined organic layers were dried over Na_2SO_4, filtered, and evaporated. The residue was dissolved in CH_2Cl_2 (50 mL), and TFA (0.5 mL) was added. The resulting solution was stirred at room temperature for 12 hours. The organic layer was washed with aq. sat. $NaHCO_3$ (1x), H_2O (1x) and brine (1x), dried over Na_2SO_4, filtered and the solvent was evaporated.

Flash column chromatography (SiO_2) with cyclohexane:CH_2Cl_2 (2:1 to 1:1) eluted first 5-(4-nitrophenyl)-15-phenyl-10,20-bis((triisopropylsilyl)ethynyl)porphyrin (**4b,** green band, 97 mg, 112 micromol, 11%), followed by 15-(4-nitrophenyl)-5-phenyl-10-((triisopropylsilyl)ethynyl)porphyrin (**4**, red band, 214 mg, 311 micromol, 31%).

Compound **4b**: ^{1}**H NMR** (400 MHz, $CDCl_3$) δ = 9.69 (d, J = 4.8 Hz, 2H), 9.66 (d, J = 4.7 Hz, 2H), 8.85 (d, J = 4.8 Hz, 2H), 8.73 (d, J = 4.8 Hz, 2H), 8.69 – 8.62 (m, 2H), 8.40 – 8.34 (m, 2H), 8.19 – 8.14 (m, 2H), 7.82 – 7.73 (m, 3H), 1.52 – 1.37 (m, 42H), -2.16 (s, 2H) ppm.^{13}**C NMR** (126 MHz, $CDCl_3$) δ = 148.54, 147.95, 141.27, 135.16, 134.54, 128.21, 127.04, 122.66, 122.11, 118.33, 108.46, 101.86, 100.34, 19.22, 11.99 ppm. *α*-Pyrrole carbons cannot be observed (appearing well-resolved between 149 – 153 ppm in **4b-Zn**), *β*-pyrrole carbons are visible as one broad feature between 130 – 134 ppm (appearing well-resolved between 131 –134 ppm in **4b-Zn**) **HR-MS** (ESI-ToF, +): m/z = $[M+H]^+$ Calcd. For $C_{54}H_{62}N_5O_2Si_2$: 868.4437; Found 868.4438.
Compound **4**: ^{1}**H NMR** (400 MHz, $CDCl_3$) δ = 10.21 (s, 1H), 9.83 (d, *J* = 4.7 Hz, 1H), 9.80 (d, *J* = 4.7 Hz, 1H), 9.33 (d, J = 4.6 Hz, 1H), 9.30 (d, J = 4.6 Hz, 1H), 8.99 (d, J = 4.8 Hz, 1H), 8.97 (d, J = 4.6 Hz, 1H), 8.87 (d, J = 4.8 Hz, 1H), 8.84 (d, J = 4.6 Hz, 1H), 8.71 – 8.64 (m, 2H), 8.44 – 8.38 (m, 2H), 8.25 – 8.18 (m, 2H), 7.84 – 7.75 (m, 3H), 1.44 (d, J = 5.9 Hz, 21H), -2.67 (s, 2H) ppm. ^{13}**C NMR** (126 MHz, $CDCl_3$) δ = 150–145 (br., **C**-NH-**C**, **C**-N-**C**, 8$C_α$), 148.50, 147.86, 141.23, 135.29, 134.73, 132.19

(2C), 131.84, 131.69, 131.63, 131.57, 131.16, 130.75, 130.28, 128.12, 127.10, 122.12, 121.45, 117.02, 108.94, 106.95, 100.72, 99.51, 19.25, 12.03 ppm. **HR-MS** (ESI-ToF, +): m/z = [*M*+H]$^+$ Calcd. For $C_{43}H_{42}N_5O_2Si$: 688.3102; Found 688.3108.z

[15-(4-nitrophenyl)-5-phenyl-10-((triisopropylsilyl)ethynyl)-porphyrinato]zinc(II) (**4-Zn**)

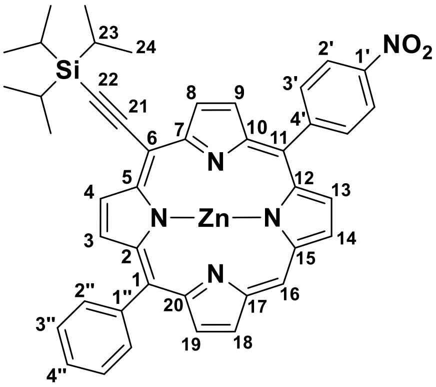

4 (22.5 mg, 38.4 micromol, 1.00 eq.) was dissolved in CH_2Cl_2/MeOH (10:1, 6 mL) and $Zn(OAc)_2$ (11.3 mg, 61.4 micromol, 1.6 eq.) was added. The resulting solution was refluxed at 40 °C for 4 h. The reaction mixture was diluted with CH_2Cl_2 (6 mL) and washed with H_2O (3 x 3 mL). The aqueous layer was reextracted with CH_2Cl_2 (2 x 2 mL), and the combined organic layers were dried over Na_2SO_4, filtered and the solvent was evaporated to give **4-Zn** (24.4 mg, 38.4 micromol, 98%).

^{1}H NMR (500 MHz, $CDCl_3$) δ = 9.88 (s, C16-**H**, 1H), 9.86 (d, J = 1.8 Hz, C4-**H**, 1H), 9.85 (d, J = 1.8 Hz, C8-**H**, 1H), 9.16 (d, J = 4.5 Hz, C18-**H**, 1H), 9.10 (d, J = 4.5 Hz, C14-**H**, 1H), 9.04 (d, J = 4.6 Hz, C3-**H**, 1H), 8.92 (d, J = 4.4 Hz, C19-**H**, 1H), 8.85 (d, J = 4.6 Hz, C9-**H**, 1H), 8.69 (d, J = 4.4 Hz, C13-**H**, 1H), 8.66 – 8.61 (m, C2'-**H**, 2H), 8.28 – 8.24 (m, C3'-**H**, 2H), 8.18 – 8.14 (m, C2''-**H**, 2H), 7.84 – 7.76 (m, C3''-**H**, C4''-**H**, 3H), 1.55 – 1.45 (m, C23-**H**, C24-**H_3**, 21H).**^{13}C NMR** (126 MHz, $CDCl_3$) δ = 152.67 (**C5**), 152.36 (**C7**), 150.63 (**C20**), 150.17 (**C2**), 149.68 (**C17**), 149.49 (**C4'**), 149.33 (**C15**), 149.15 (**C12**), 148.75 (**C10**), 147.72 (**C1'**), 142.17 (**C1''**), 135.17 (**C3'**), 134.59 (**C2''**), 133.37 (**C3**), 132.85 (**C19**), 132.41 (**C14**), 132.18 (**C18**), 131.91 (**C9**), 131.84 (**C8**), 131.49 (**C4**), 131.34 (**C13**), 127.89 (**C4''**), 126.86 (**C3''**), 122.19 (**C1**), 121.87 (**C2'**), 117.89 (**C11**), 109.25 (**C6**), 107.62 (**C16**), 101.31 (**C21**), 98.82 (**C22**), 19.28 (**C24**), 12.07 (**C23**) ppm. **HR-MS** (ESI-ToF, +): m/z = [*M*+H]$^+$ Calcd. For $C_{43}H_{40}N_5O_2SiZn$: 750.2237; Found 750.2224.

[15-(4-nitrophenyl)-5-phenyl-10,20-bis((triisopropylsilyl)ethynyl)-porphyrinato]zinc(II) (**4b-Zn**)

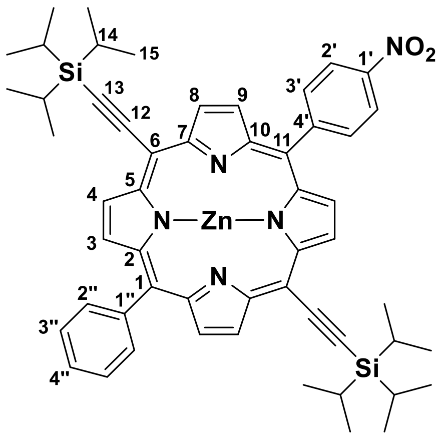

4b (25 mg, 28.8 micromol, 1.00 eq.) was dissolved in CH_2Cl_2/MeOH (9:1, 10 mL) and $Zn(OAc)_2$ (11.0 mg, 60.0 micromol, 2.08 eq.) was added. The reaction mixture was refluxed at 40 °C for 12 hours, washed with H_2O (3 x 2 mL) and the aqueous layers were re-extracted with CH_2Cl_2 (2 x 2 mL). The combined organic layers were dried over Na_2SO_4, filtered, and dried *in vacuo* to give the crude product as a green solid.

Flash column chromatography (SiO_2) with cyclohexane/CH_2Cl_2 (1:1) provided **4b-Zn** as a green solid (26 mg, 27.9 mmol, 97%).

^{1}H NMR (500 MHz, $CDCl_3$) δ = 9.78 (dd, J = 4.6, 3.3 Hz, C4-**H**, C8-**H**, 4H), 8.96 (d, J = 4.6 Hz, C3-**H**, 2H), 8.81 (d, J = 4.6 Hz, C9-**H**, 2H), 8.65 – 8.61 (m, C2'-**H**, 2H), 8.37 – 8.33 (m, C3'-**H**, 2H), 8.20 – 8.17 (m, C2''-**H**, 2H), 7.83 – 7.75 (m, C3''-**H**, C4''-**H**, 3H), 1.49 – 1.41 (m, C14-**H**, C15-**H_3**, 42H) ppm. **^{13}C NMR** (126 MHz, $CDCl_3$) δ = 152.81 (**C5**), 152.55 (**C7**), 150.54 (**C2**), 149.38 (**C4'**), 149.11 (**C10**), 147.78 (**C1'**), 142.03 (**C1''**), 135.07 (**C3'**), 134.45 (**C2''**), 133.38 (**C3**), 132.03 (**C8**), 131.97 (**C9**), 131.69 (**C4**), 127.98 (**C4''**), 126.86 (**C3''**), 123.48 (**C1**), 121.91 (**C2'**), 119.27 (**C11**), 108.97 (**C6**), 102.47 (**C12**), 99.55 (**C13**), 19.24 (**C15**), 12.02 (**C14**) ppm. **HR-MS** (ESI-ToF, +): m/z = $[M+H]^+$ Calcd. For $C_{54}H_{60}N_5O_2Si_2Zn$: 930.3572; Found 930.3556.

15-(4-aminophenyl)-5-phenyl-10-((triisopropylsilyl)ethynyl)porphyrin (**5**)

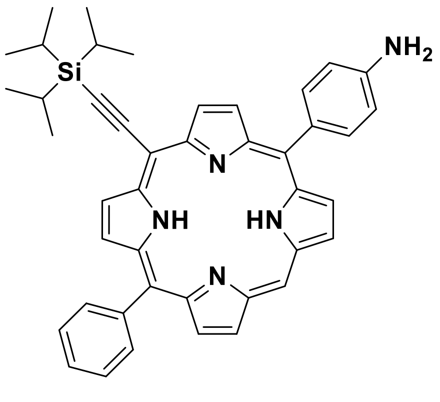

Under argon atmosphere, **4** (214 mg, 0.311 mmol, 1.00 equiv.) was dissolved in THF/MeOH (2:1, 105 mL), and degassed for 30 minutes with argon. Freshly activated zinc dust (140 mg, 2.14 mmol, 6.88 eq.) and NH_4Cl (60 mg, 1.12 mmol, 3.44 eq.) were added in portions over the course of 30 minutes, and stirring was continued at room temperature for 1 h, until MALDI analysis indicated absence of the nitro substrate and the nitrosyl intermediate. The reaction mixture was poured on aq. sat. $NaHCO_3$

(350 mL), and diluted with CH_2Cl_2 (400 mL). The layers were separated, and the aqueous layer washed with H_2O (2x), and brine (1x), dried over Na_2SO_4, filtered and the solvent was evaporated, and the residue dried *in vacuo* overnight, containing a mixture of **5** and **5-Zn** (monitored by ^{1}H NMR and MALDI).

The dried residue was dissolved in CH_2Cl_2 (60 mL), and degassed with a stream of argon. TFA (6 mL) was added, and the solution was stirred at room temperature for 1 hour. It was poured on aq. sat. $NaHCO_3$ (20 mL), and the layers were separated. The organic layer was washed with aq. sat. $NaHCO_3$ (1 x 20 mL), H_2O (1 x 20 mL), and brine (1 x 20 mL), dried over Na_2SO_4, filtered, and the solvent was evaporated.

Flash column chromatography (SiO_2) with cyclohexane: CH_2Cl_2 (1:1) (eluting the diazene side product) to 3:7 (eluting **5**) gave **5** (106 mg, 0.161 mmol, 52%) as a purple crystalline solid.

^{1}H NMR (400 MHz, $CDCl_3$) δ = 10.15 (s, 1H), 9.78 (d, *J* = 4.8 Hz, 2H), 9.27 (dd, *J* = 4.6, 1.3 Hz, 2H), 9.05 (dd, *J* = 8.6, 4.7 Hz, 2H), 8.94 (dd, *J* = 8.4, 4.7 Hz, 2H), 8.26 – 8.18 (m, 2H), 8.04 – 7.96 (m, 2H), 7.82 – 7.74 (m, 3H), 7.14 – 7.06 (m, 2H), 4.06 (s, 2H), 1.52 – 1.37 (m, 21H), -2.61 (s, 2H). **^{13}C NMR** (101 MHz, $CDCl_3$) δ = 146.33, 141.55, 135.93, 134.75, 131.98 (brs, **$C_β$**), 131.67 (brs, **$C_β$**) 131.63, 131.58 (brs, **$C_β$**), 131.48 (brs, **$C_β$**), 131.45 (brs, **$C_β$**), 131.15 (brs, **$C_β$**), 130.90 (brs, **$C_β$**), 130.66 (brs, **$C_β$**), 127.91, 127.03, 121.36, 120.23, 113.80, 109.42, 106.55, 99.89, 98.61, 19.26, 12.05 ppm. *α*-Pyrrole carbons can be observed as one broad feature between 145 – 150 ppm (appearing well-resolved between 149 – 153 ppm in **5-Zn**) **HR-MS** (ESI-ToF, +): m/z = [*M*+H]$^+$ Calcd. For $C_{43}H_{44}N_5Si$: 658.3360; Found 658.3358.

[15-(4-aminophenyl)-5-phenyl-10-((triisopropylsilyl)ethynyl)-porphyrinato]zinc(II) (**5-Zn**)

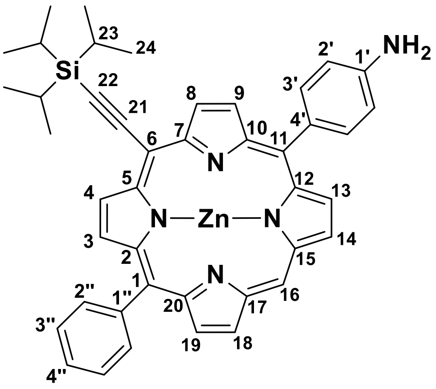

4-Zn (523 mg, 0.696 mmol, 1.00 equiv.) was dissolved in anhydrous THF (62 mL) in a 100 mL pressure flask and Pd/C (10 wt% Pd, 284 mg, 0.176 mmol, 57.5 mol%) was added. The reaction mixture was heated to 80 °C and hydrazine monohydrate (65 wt%, 11.6 mL, 155 mmol, 222.7 eq.) was added in three equimolar portions over the course of 24 h until TLC indicated full conversion of the starting material. The reaction mixture was allowed to come to room temperature, was washed with aq. sat. NH_4Cl (1x) and H_2O (2x), dried over Na_2SO_4, filtered and the solvent was evaporated to give a purple solid with green luster.

Flash column chromatography (SiO_2) with CH_2Cl_2/cyclohexane (6:4) provided **5-Zn** as a purple solid (380 mg, 0.527 mmol, 76%).

^{1}H NMR (400 MHz, $CDCl_3$) δ = 10.20 (s, C16-**H**, 1H), 9.88 (d, *J* = 4.6 Hz, C8-**H**, 1H), 9.86 (d, *J* = 4.6 Hz, C4-**H**, 1H), 9.49 (d, *J* = 4.5 Hz, C14-**H**, 1H), 9.35 (d, *J* = 4.5 Hz, C18-**H**, 1H), 9.06 (d, *J* = 4.6 Hz, C3-**H**, 1H), 9.04 (d, *J* = 4.5 Hz, C19-**H**, 1H), 8.97 (d, *J* = 4.4 Hz, C13-**H**, 1H), 8.87 (d, *J* = 4.6 Hz, C9-**H**, 1H), 8.31 – 8.24 (m, C2''-**H**, 2H), 7.80 – 7.72 (m, C3''-**H**, C4''-**H**, 3H), 7.43 (d, *J* = 8.0 Hz, C3'-**H**, 2H), 5.08 (d, *J* = 7.6 Hz, C2'-**H**, 2H), 1.45 – 1.34 (m, C23-**H**, C24-**H₃**, 21H) ppm. **^{13}C NMR** (126 MHz,

$CDCl_3$) δ = 152.47 (**C5**), 152.25 (**C7**), 150.85 (**C10**), 150.45 (**C20**), 149.98 (**C12**), 149.78 (**C2**), 149.74 (**C17**), 149.66 (**C15**), 142.93 (**C1''**), 137.81 (**C1'**), 135.31 (**C4'**), 134.85 (**C2''**), 133.25 (**C3'**), 132.97 (**C9**), 132.94 (**C13**), 132.87 (**C3**), 132.40 (**C19**), 131.97 (**C18**), 131.81 (**C14**), 131.06 (**C4**), 130.81 (**C8**), 127.55 (**C4''**), 126.72 (**C3''**), 121.09 (**C1**), 120.66 (**C11**), 113.04 (**C2'**), 110.29 (**C6**), 107.61 (**C16**), 99.89 (**C**$_{\mathbf{acetylene}}$), 97.40 (**C**$_{\mathbf{acetylene}}$), 19.22 (**C24**), 12.03 (**C23**) ppm. **HR-MS** (ESI-ToF, +): m/z = $[M+H]^+$ Calcd. For $C_{43}H_{42}N_5SiZn$: 720.2495; Found 720.2488.

7-Zn

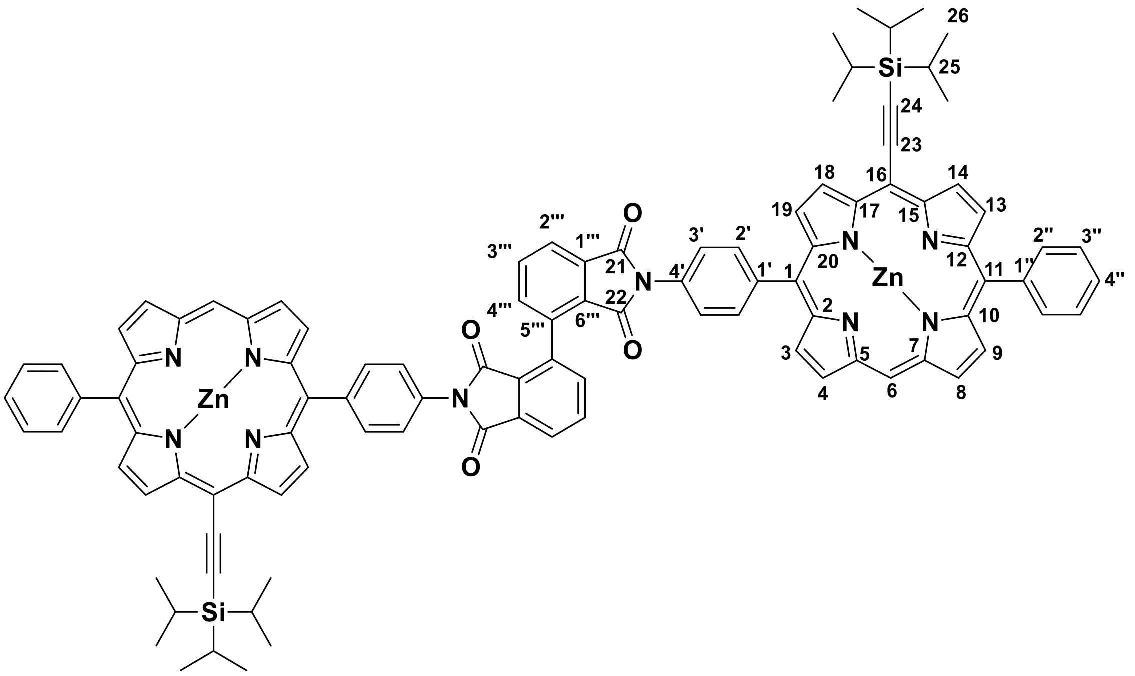

6 (16.4 mg, 55.7 micromol, 1.00 eq.) and **5-Zn** (82.0 mg, 114 micromol, 2.04 eq.) were dissolved in NMP (0.2 mL) and degassed for 5 minutes with argon. The deep blue solution was heated to 180 °C in the microwave for 1h and 45 minutes. The NMP was removed at 80 °C by N_2 flow evaporation. The resulting purple solid was subjected to GPC purification in $CHCl_3$, which provided **7-Zn**as a purple solid with green luster (86 mg, 50.6 micromol, 91%).

^{1}**H NMR** (500 MHz, THF-d8) δ = 10.07 (s, C6-**H**, 2H), 9.79 (d, J = 4.6 Hz, C18-**H**, 2H), 9.76 (d, J = 4.5 Hz, C14-**H**, 2H), 9.27 (d, J = 4.5 Hz, C8-**H**, 2H), 9.23 (d, J = 4.4 Hz, C4-**H**, 2H), 9.02 (d, J = 4.6 Hz, C19-**H**, 2H), 9.00 (d, J = 4.4 Hz, C3-**H**, 2H), 8.90 (d, J = 4.5 Hz, C9-**H**, 2H), 8.89 (d, J = 4.5 Hz, C13-**H**, 2H), 8.37 – 8.33 (m, C2'-**H**, 4H), 8.26 (dd, J = 7.3, 1.1 Hz, C2'''-**H**, 2H), 8.20 – 8.16 (m, C2''-**H**, 4H), 8.11 – 8.07 (m, C3'''-**H**, 2H), 8.04 (dd, J = 7.8, 1.1 Hz, C4'''-**H**, 2H), 8.01 – 7.97 (m, C3'-**H**, 4H), 7.80 – 7.73 (m, C3''-**H,** C4''-**H**, 6H), 1.47 – 1.40 (m, C25-**H,** C26-**H**$_3$, 42H) ppm. ^{13}**C NMR** (126 MHz, THF-d8) δ = 167.99 (**C22**), 167.59 (**C21**), 153.32 (**C**$_α$), 153.26 (**C**$_α$), 151.52 (**C**$_α$), 151.45 (**C**$_α$), 150.77 (**C**$_α$, 3C), 150.72 (**C**$_α$), 144.15 (**C1''**), 143.39 (**C1'**), 137.18 (**C4'''**), 136.05 (**C5'''**), 135.78 (**C2'**), 135.60 (**C2''**), 135.12 (**C3'''**), 133.68 (**C1'''**), 133.35 (**C19**), 133.30 (**C9**), 133.13 (**C4'**), 132.94 (**C4**), 132.84 (**C3**, **C13**, 2C), 132.73 (**C8**), 131.64 (**C18**), 131.39 (**C14**), 130.95 (**C6'''**), 128.37 (**C4''**), 127.44 (**C3''**), 125.65 (**C3'**), 124.54 (**C2'''**), 122.21 (**C11**), 121.15 (**C1**), 111.98 (**C16**), 108.47 (**C6**), 100.14 (**C23**), 97.14 (**C24**), 19.67 (**C26**), 13.04 (**C25**) ppm. **HR-MS** (ESI-ToF, +): m/z = $[M+Na]^+$ Calcd. For $C_{102}H_{84}N_{10}NaO_4Si_2Zn_2$: 1719.4691; Found 1719.4692.

***(rac)*-PoGe-[Zn, Zn]**

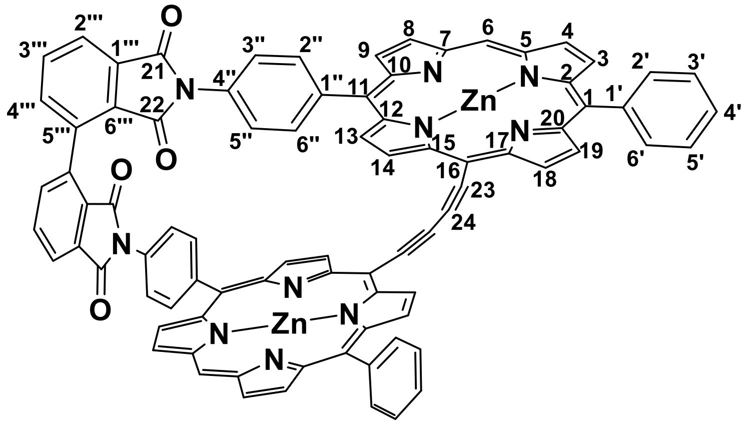

7-Zn (13.0 mg, 6.11 micromol, 1.00 eq.) was dissolved in 2 mL CH_2Cl_2 and 1 mL pyridine and degassed with argon for 5 min. TBAF (1 M in THF, 0.1 mL) was added and the reaction stirred for 2 h. The mixture was diluted with 6.5 mL pyridine and degassed for 40 min. $Cu(OAc)_2$ (23.5 mg, 0.13 mmol, 21.0 eq.) and CuCl (9.34 mg, 91.5 micromol, 15.0 eq.) were added and the mixture was heated to 50 °C and stirred for 18 h. Volatiles were removed, the residue dissolved in EtOH/CH_2Cl_2 (1:99) and filtered through a syringe filter before purification by np-HPLC (1% EtOH in CH_2Cl_2). After volatiles were removed, the obtained solid was dissolved in an aliquot of CH_2Cl_2**,** over layered with pentane and allowed to precipitate in the fridge yielding **(*rac*)-PoGe[Zn, Zn]** as moss green powder (7.00 mg, 6.10 micromol, 83%).

^{1}H NMR (500 MHz, THF-d8) δ = 10.36 (d, J = 4.5 Hz, C14-**H**, 2H), 10.22 (s, C6-**H**, 2H), 9.80 (d, J = 4.4 Hz, C18-**H**, 2H), 9.38 (d, J = 4.4 Hz, C8-**H**, 2H), 9.34 (d, J = 4.3 Hz, C4-**H**, 2H), 9.26 (d, J = 4.5 Hz, C13-**H**, 2H), 9.17 (d, J = 4.3 Hz, C9-**H**, 2H), 9.02 (d, J = 4.5 Hz, C19-**H**, 2H), 8.92 (d, J = 4.3 Hz, C3-**H**, 2H), 8.44 (dd, J = 8.0, 2.2 Hz, C2''-**H**, 2H), 8.33 (dd, J = 8.0, 2.2 Hz, C6''-**H**, 2H), 8.29 – 8.24 (m, C2'-**H**, C6'-**H**, 4H), 8.24 (dd, J = 7.3, 1.0 Hz, C2'''-**H**, 2H), 8.15 (dd, J = 8.0, 2.3 Hz, C5''-**H**, 2H), 8.11 (dd, J = 8.2, 7.3 Hz, C3'''-**H**, 2H), 8.02 (dd, J = 7.9, 2.3 Hz, C3''-**H**, 2H), 7.96 (dd, J = 8.1, 1.1 Hz, C4'''-**H**, 2H), 7.84 – 7.79 (m, C3'-**H**, C4'-**H**, C5'-**H**, 6H) ppm. **^{13}C NMR** (126 MHz, THF-d8) δ = 167.65 (**C21**), 167.44 (**C22**), 155.49 (**C15**), 151.93 (**C2**), 151.72 (**C17**), 151.29 (**C10**), 151.08 (**C20**), 151.04 (**C7**), 150.95 (**C12**), 150.93 (**C5**), 144.13 (**C1'**), 142.87 (**C1''**), 137.30 (**C4'''**), 136.54 (**C6''**), 136.29 (**C5'''**), 135.63 (**C2'/C6'**), 135.61 (**C2'/C6'**), 135.53 (**C3'''**), 135.13 (**C13**), 135.04 (**C2''**), 133.68 (**C19**), 133.30 (**C4''**), 133.17 (**C1'''**), 133.07 (**C8**), 133.00 (**C3**), 132.96 (**C4**), 132.72 (**C9**), 131.68 (**C14**), 131.02 (**C6'''**), 130.45 (**C18**), 128.48 (**C4'**), 127.57 (**C3'**, **C5'**), 125.05 (**C5''**), 124.59 (**C2'''**), 124.30 (**C3''**), 122.86 (**C1**), 122.12 (**C11**), 109.17 (**C6**), 99.18 (**C16**), 95.10 (**$C_{acetylene}$**), 84.59 (**$C_{acetylene}$**) ppm. **HR-MS** (ESI-ToF, +): m/z = $[M+H]^+$ Calcd. For $C_{84}H_{42}N_{10}O_4Zn_2$: 1382.1968; Found 1382.1953.

***(rac)*-PoGe-[2H,2H]**

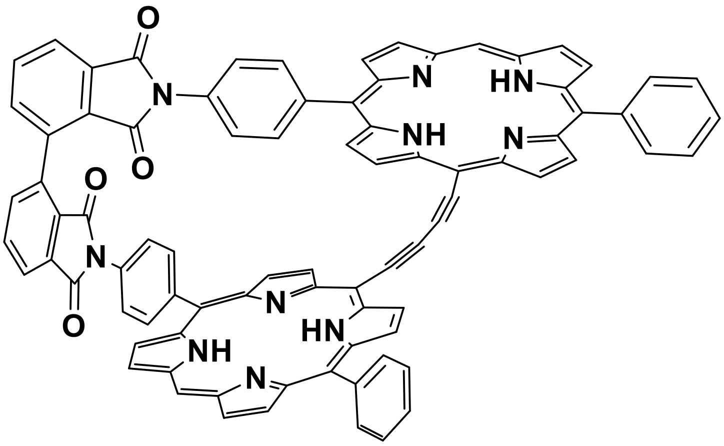

***(rac)*-PoGe-[Zn, Zn]** (5.1 mg, 3.68 micromol, 1.00 eq.) was dissolved in TFA (2 mL) and stirred for 3 minutes. The TFA was evaporated, the residue redissolved in CH_2Cl_2 (10 mL) and washed with $NaHCO_3$ aq. sat. (2 x 20 mL) and H_2O (2 x 20 mL). The organic layer was dried over Na_2SO_4, filtered, and the solvent evaporated. The deep green residue was subjected to GPC purification in $CHCl_3$ to provide ***(rac)*-PoGe-[2H, 2H]**as a green solid (2.75 mg, 2.14 micromol, 61%).

^{1}H NMR (400 MHz, $CDCl_3$) δ = 10.28 (d, J = 4.7 Hz, 2H), 10.19 (s, 2H), 9.81 (d, J = 4.7 Hz, 2H), 9.34 (d, J = 4.6 Hz, 2H), 9.27 (d, J = 4.5 Hz, 2H), 9.17 (d, J = 4.5 Hz, 2H), 9.10 (d, J = 4.7 Hz, 2H), 9.04 (d, J = 4.7 Hz, 2H), 8.95 (dd, J = 43.8, 4.5 Hz, 2H), 8.51 (dd, J = 8.1, 2.1 Hz, 2H), 8.35 (dd, J = 8.2, 2.2 Hz, 2H), 8.26 (m, J = 6.1 Hz, 6H), 8.15 (dd, J = 8.2, 2.3 Hz, 2H), 8.07 (t, J = 7.7 Hz, 2H), 7.97 (dd, J = 8.0, 2.3 Hz, 2H), 7.89 (d, J = 8.0 Hz, 2H), 7.86 – 7.80 (m, 6H), -2.31 (s, 4H) ppm. **^{13}C NMR** (151 MHz, THF-d8) δ = 167.61, 167.41, 142.38, 141.19, 137.31, 136.70, 136.29, 135.70, 135.67, 135.58, 134.90, 133.77, 133.15, 130.99, 129.02, 128.12 (2C), 125.62, 124.82, 124.65, 122.36, 121.76, 108.72, 98.98, 94.19, 84.94 ppm. *α*-Pyrrole carbons cannot be observed (appearing well-resolved between 150 – 155 ppm in ***(rac*)-PoGe-[Zn, Zn]**), *β*-pyrrole carbons are visible as broad features between 130 – 135 ppm (appearing well-resolved between 130 –136 ppm in ***(rac)*-PoGe-[Zn, Zn]**) **HR-MS** (ESI-ToF, +): m/z = [*M*+H]$^+$ Calcd. For $C_{84}H_{47}N_{10}O_4$: 1259.3776; Found 1259.3755.

***(rac)*-PoGe-[Cu, Cu]**

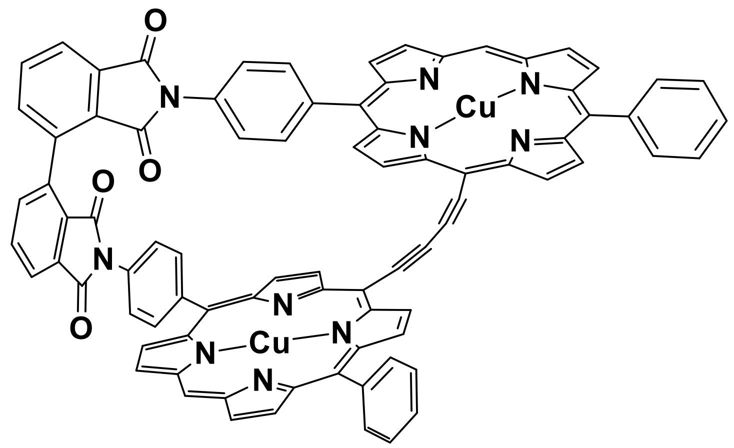

Under argon atmosphere, analytical amounts of ***(rac)*-PoGe-[2H, 2H]**(0.8 mg, 0.635 µmol, 1.00 eq.) were dissolved in THF/CH_2Cl_2/NEt_3 (5:5:2, 1.5 mL) and degassed with a stream of argon. $Cu(OAc)_2$ (monohydrate, 5.1 mg, 25.5 µmol, 40.2 eq.) was added, and the resulting solution was stirred at room temperature for 48 h, until aliquots analyzed by normal-phase HPLC (heptane/EtOAc) showed the presence of a single species. The solvent was evaporated, and the resulting solid resuspended in CH_2Cl_2, filtered and evaporated again. The residue was purified by normal-phase HPLC (1% EtOH in CH_2Cl_2), to provide ***(rac)*-PoGe-[Cu, Cu]** for UV-Vis, fluorescence and CD characterization.

HR-MS (ESI-ToF, +): m/z = $[M+H]^+$ Calcd. For $C_{84}H_{43}Cu_2N_{10}O_4$: 1381.2055; Found 1381.2066.

NMR Spectra

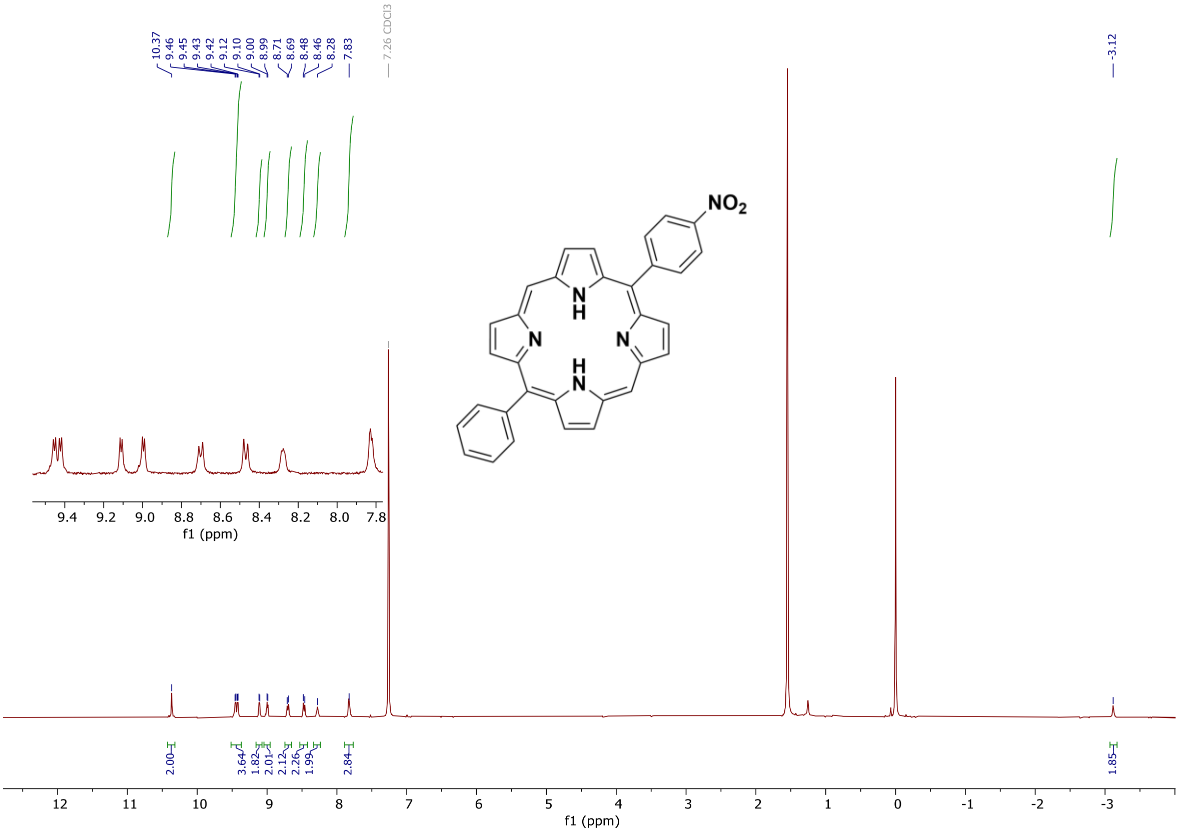

Figure S1: **^{1}H NMR** spectrum of **2** in **$CDCl_3$** (400 MHz)

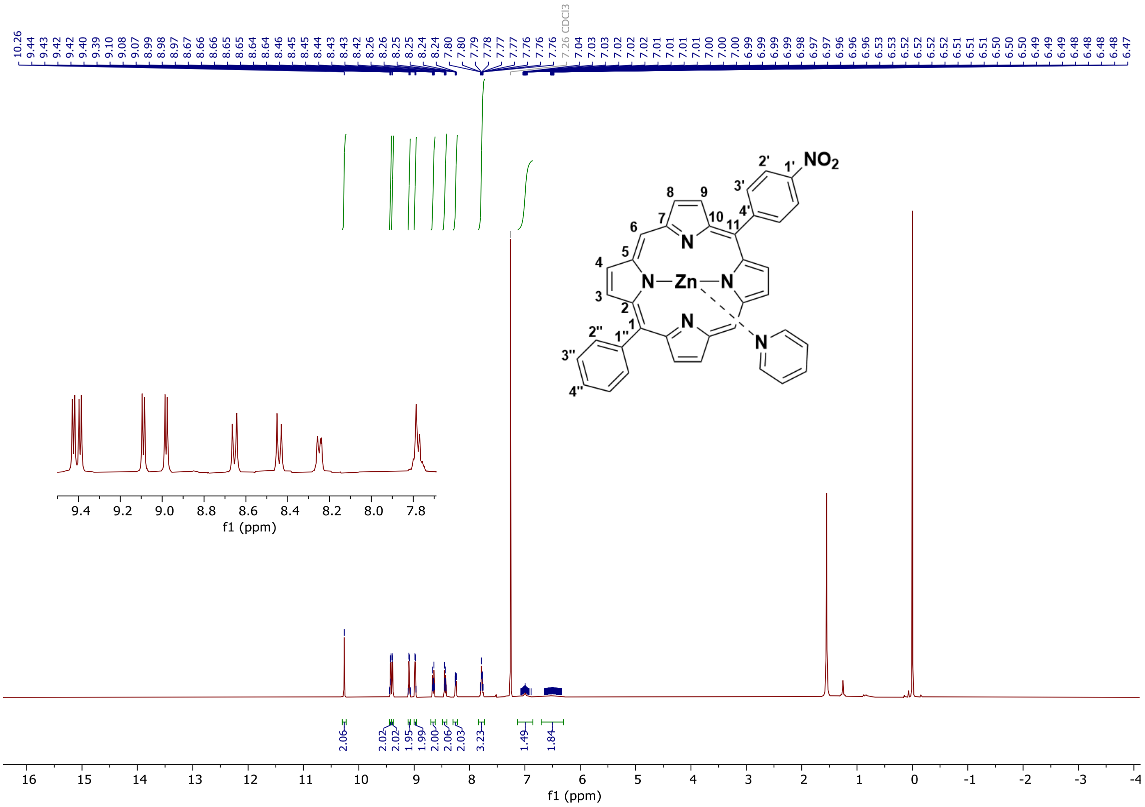

Figure S2: **^{1}H NMR** spectrum of **2-Zn-pyr** in **$CDCl_3$** (400 MHz). Only two of three expected pyridine signals are visible (broad, and shifted upfield). The third signal is likely located upfield of 6 ppm and too broad to be visible, most likely due to the systems specific exchange kinetics.

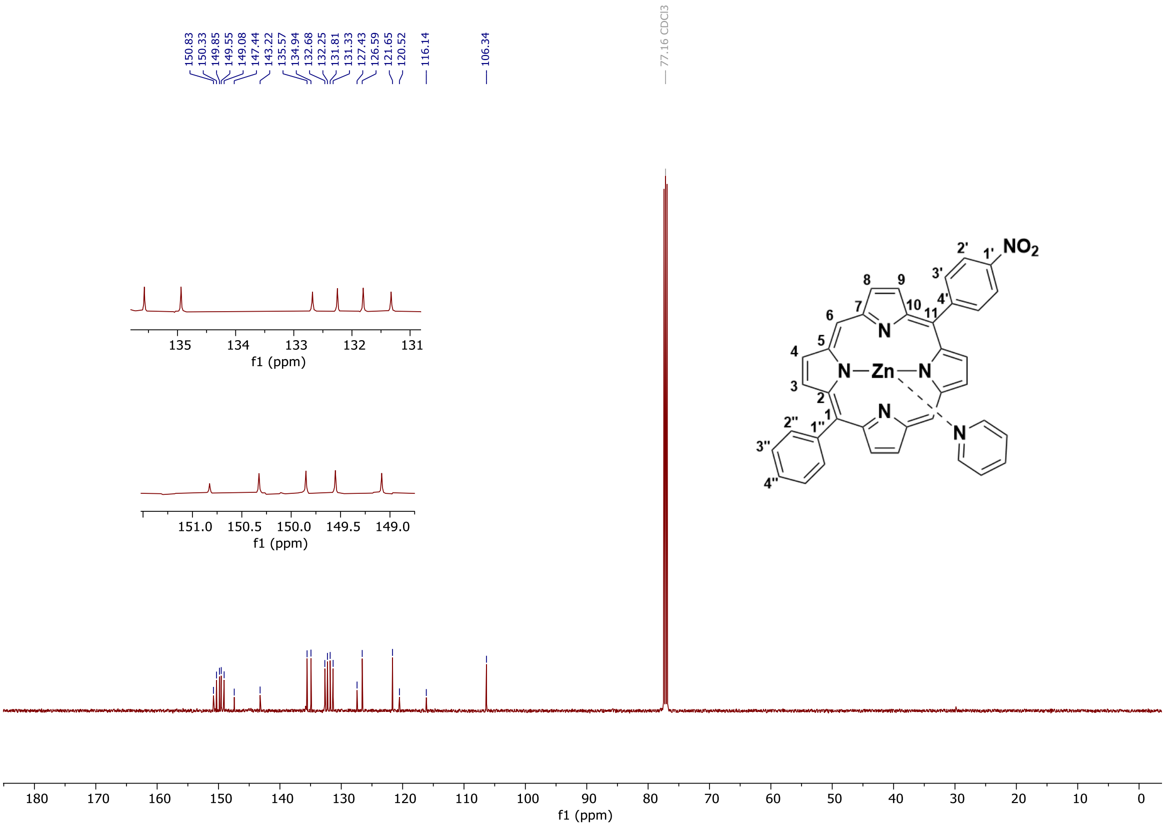

Figure S3: ^{13}**C NMR** spectrum of **2-Zn-pyr** in $CDCl_3$ (126 MHz)

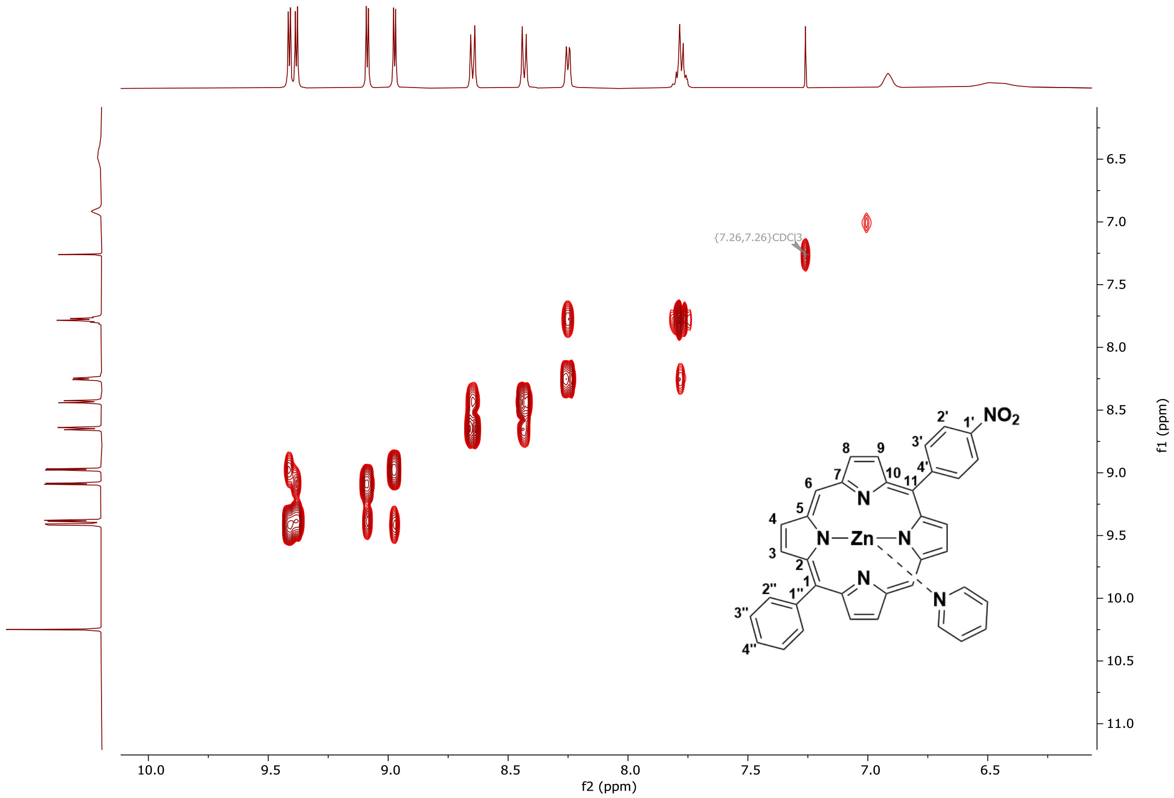

Figure S4: COSY NMR spectrum of **2-Zn-pyr** in $CDCl_3$ (500 MHz)

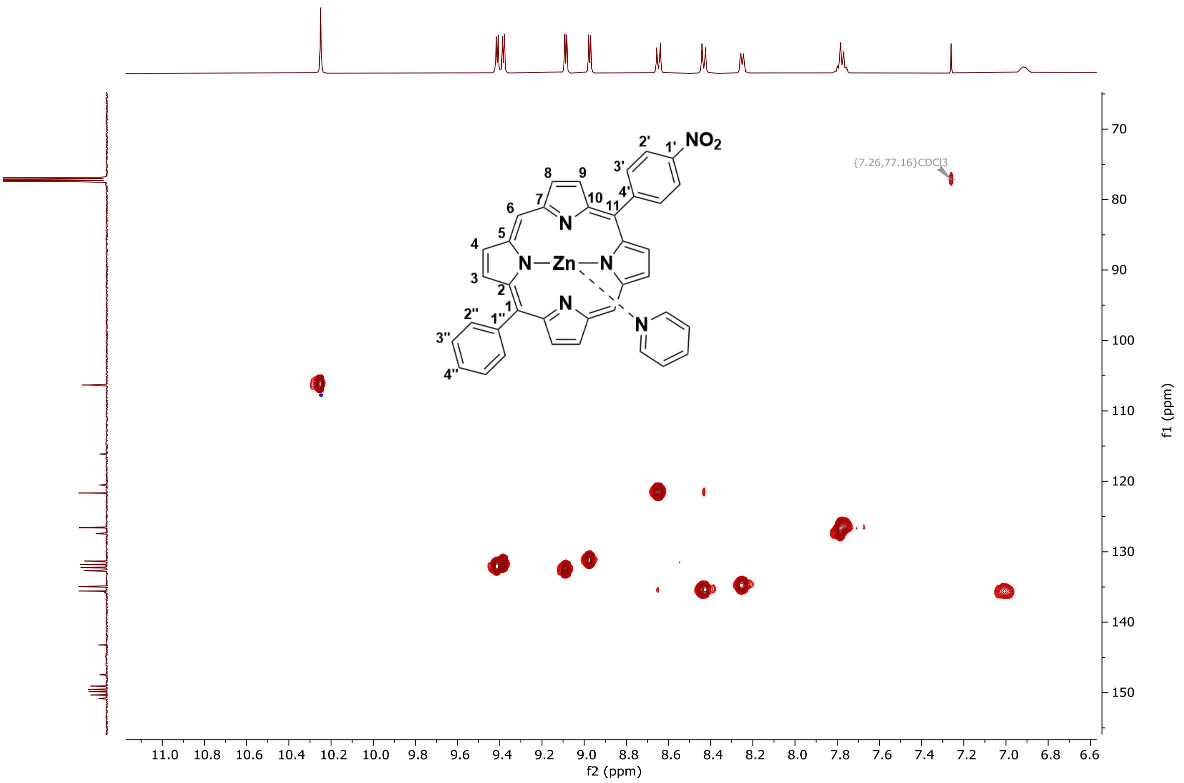

Figure S5: HMQC NMR spectrum of **2-Zn-pyr** in **$CDCl_3$** (500 MHz)

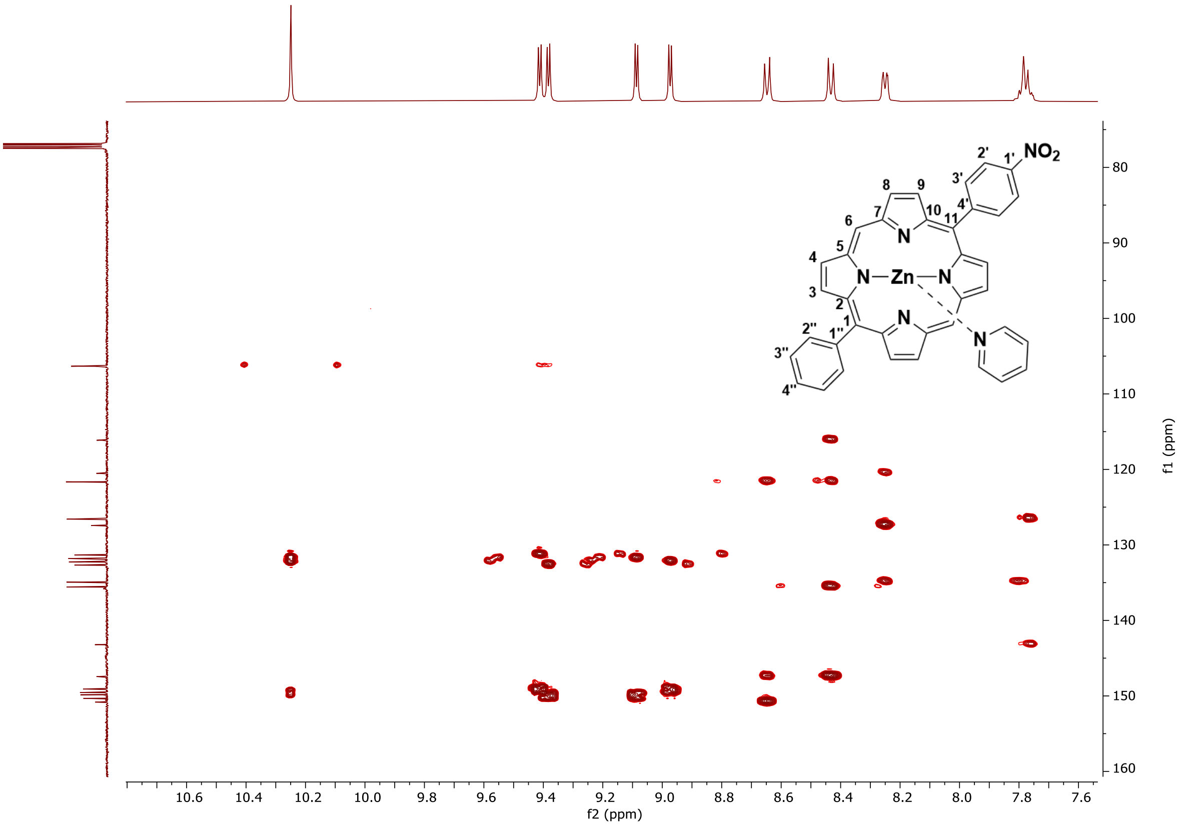

Figure S6: HMBC NMR spectrum of **2-Zn-pyr** in **$CDCl_3$** (500 MHz)

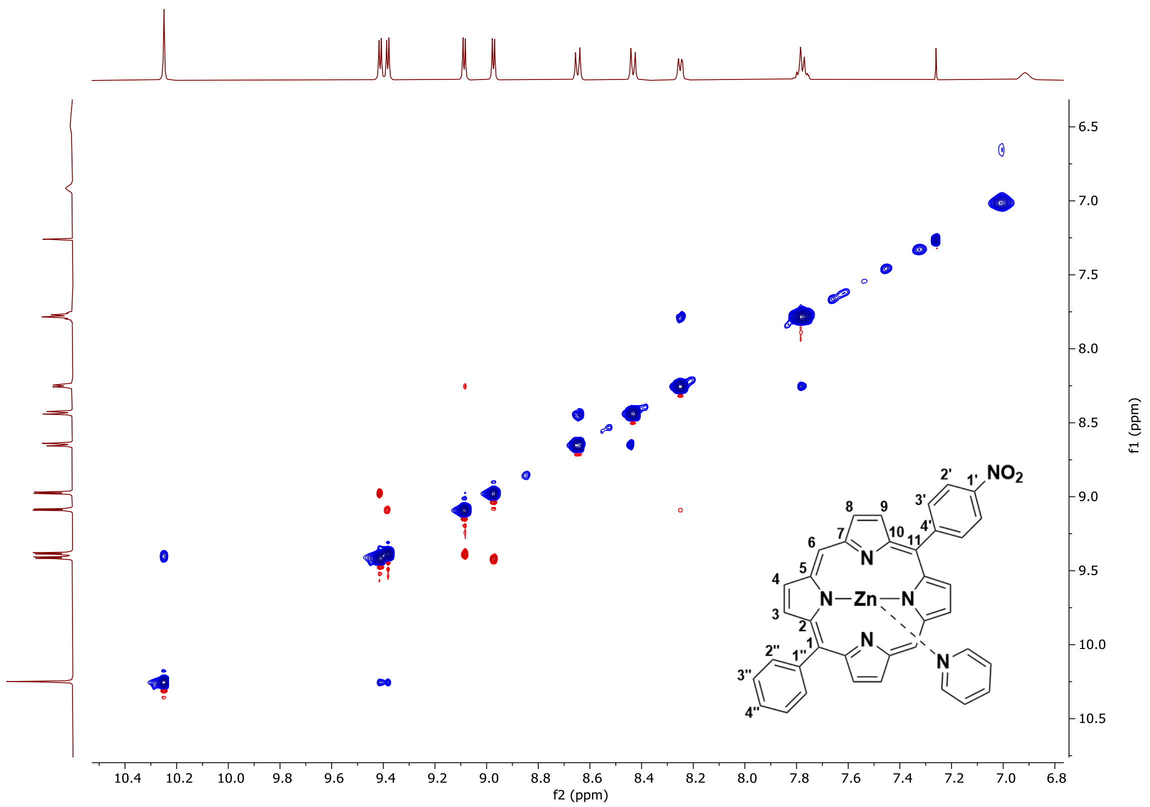

Figure S7: NOESY NMR spectrum of **2-Zn-pyr** in **$CDCl_3$** (500 MHz)

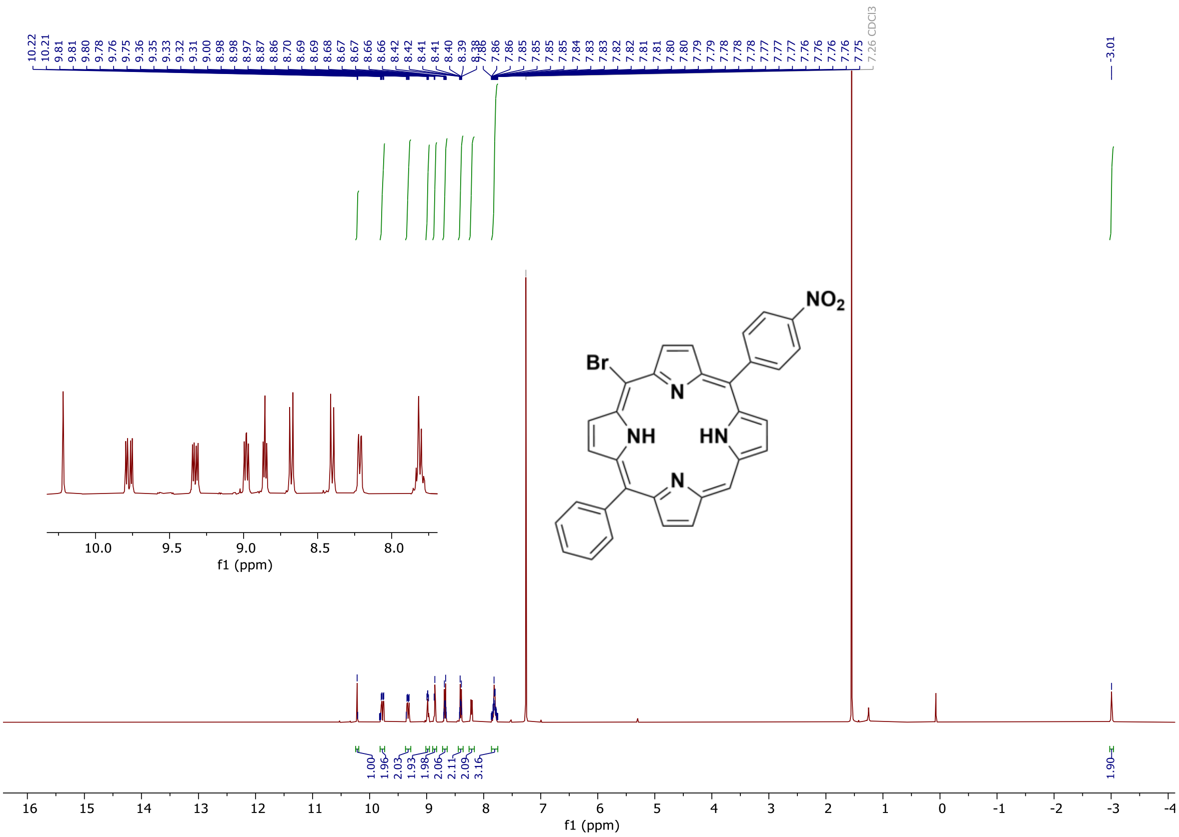

Figure S8: ^{1}H NMR spectrum of **3** in **$CDCl_3$** (400 MHz)

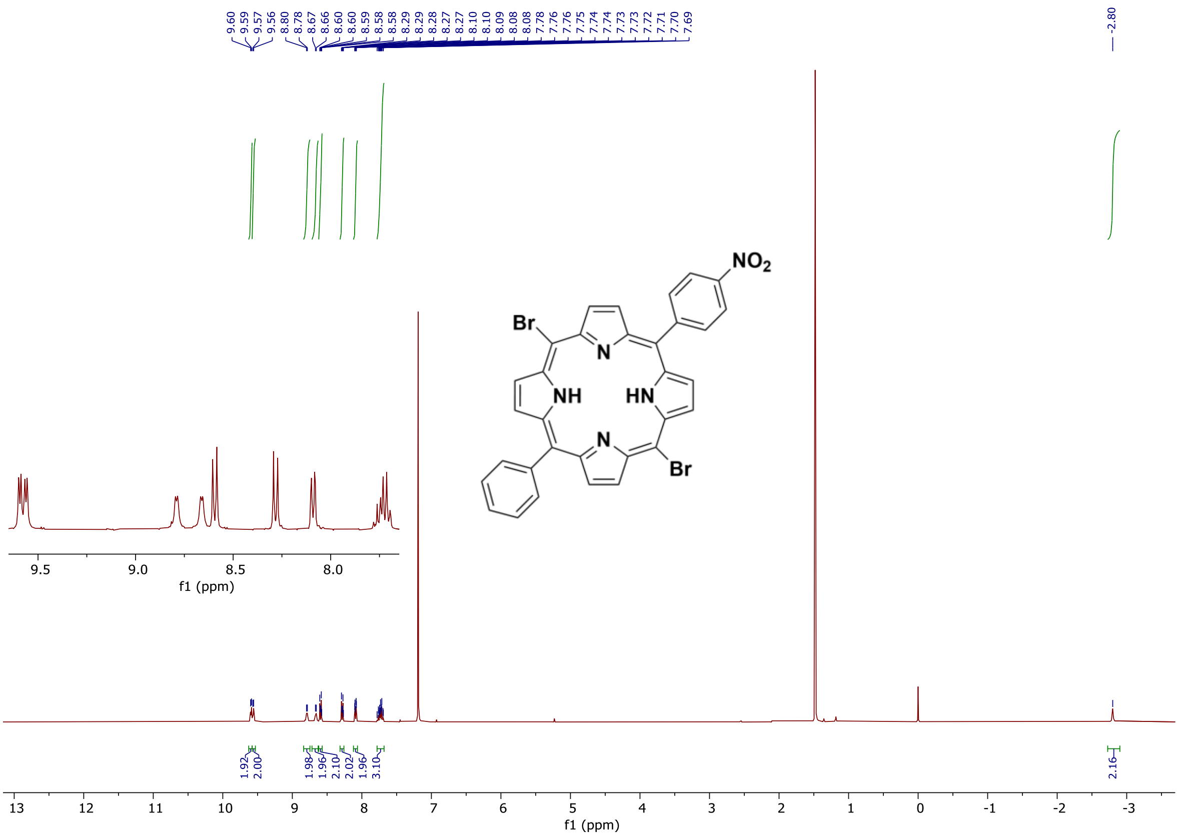

Figure S9: ^{1}H NMR spectrum of **3b** in **$CDCl_3$** (400 MHz)

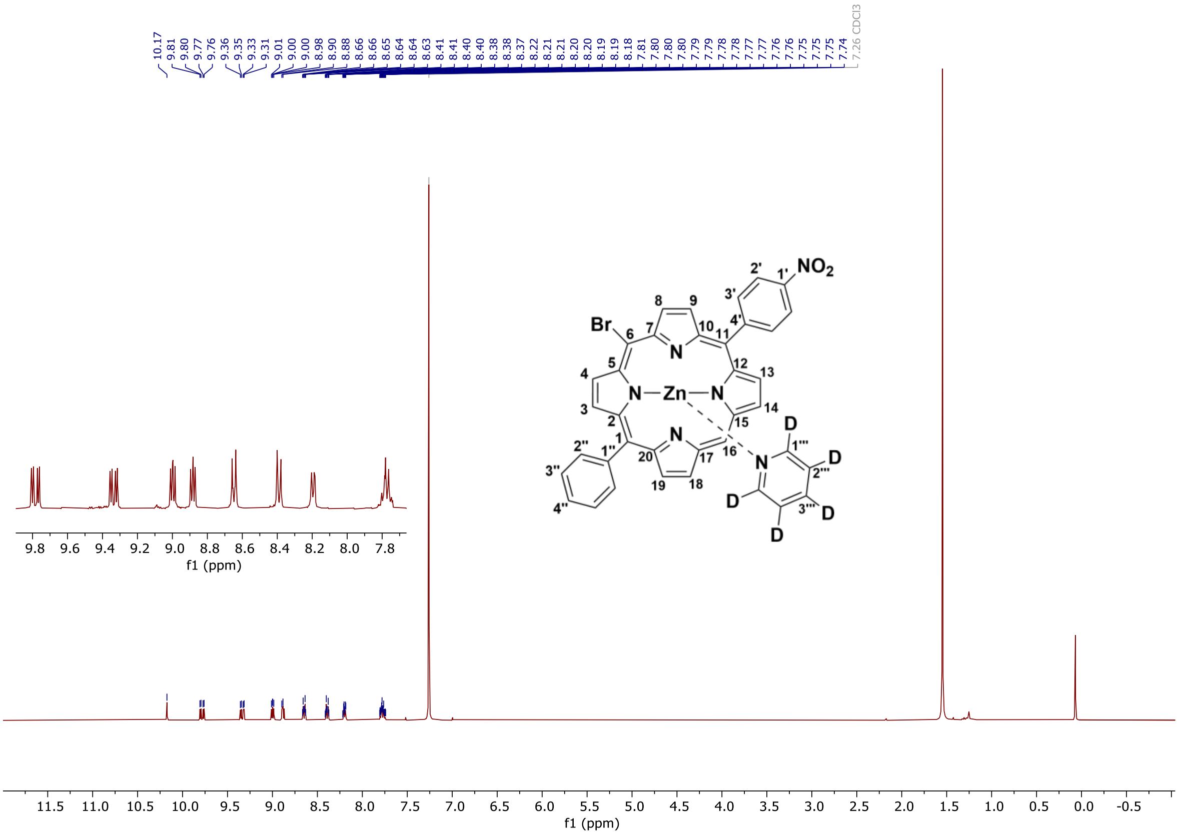

Figure S10: ^{1}H NMR spectrum of **3-Zn-pyrd5** in **$CDCl_3$** (400 MHz)

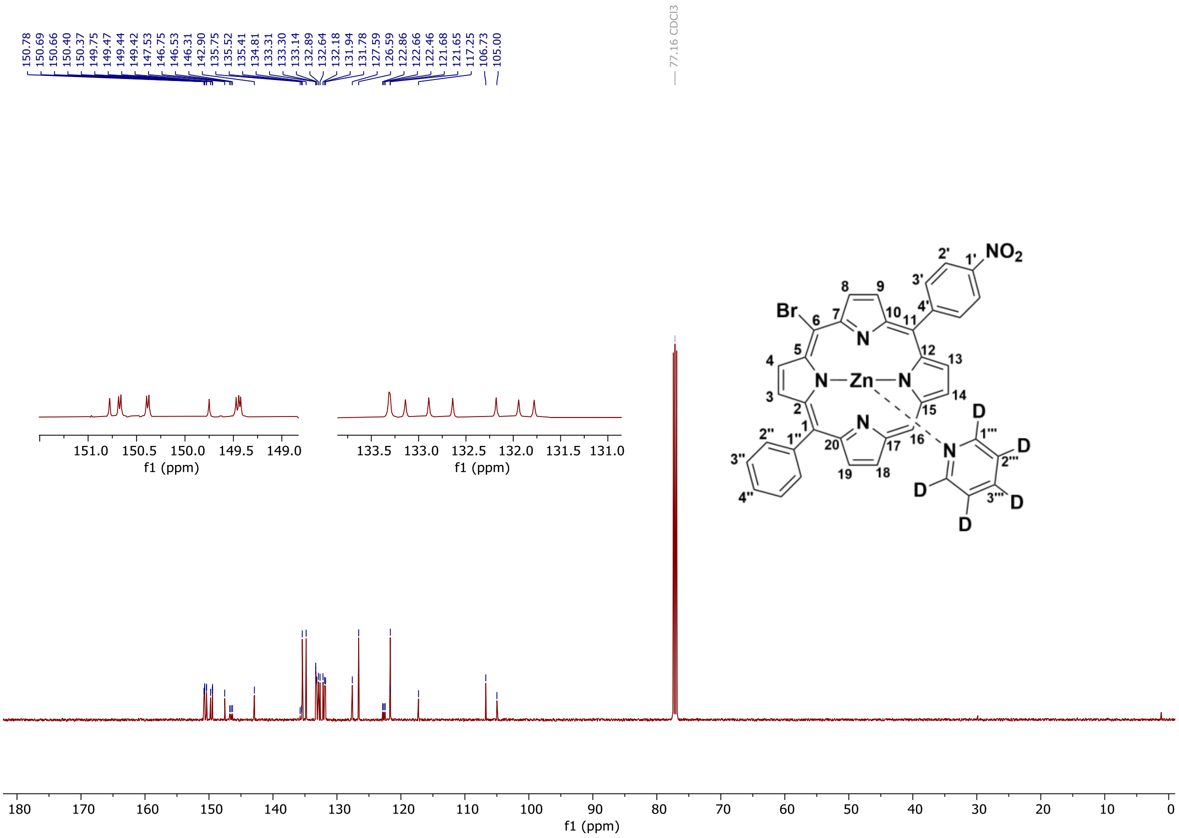

Figure S11: ^{13}C NMR spectrum of **3-Zn-pyrd5** in **$CDCl_3$** (126 MHz)

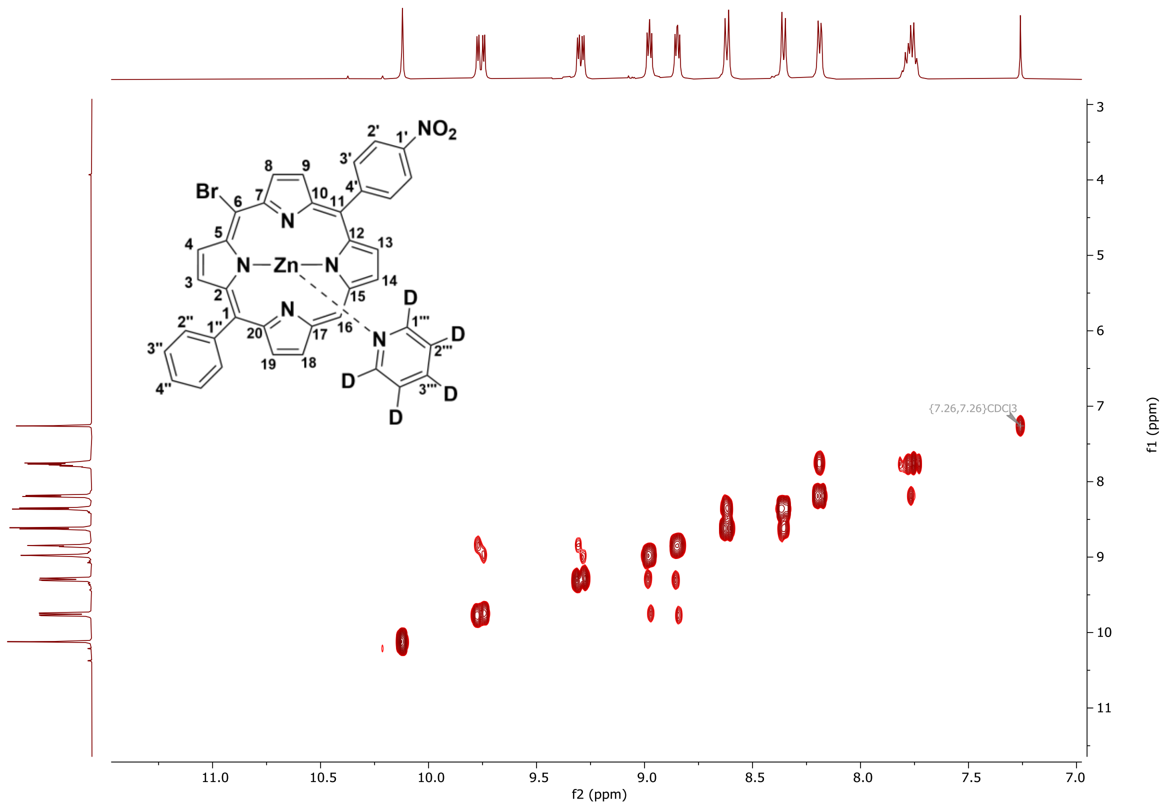

Figure S12: COSY NMR spectrum of **3-Zn-pyrd5** in **$CDCl_3$** (500 MHz)

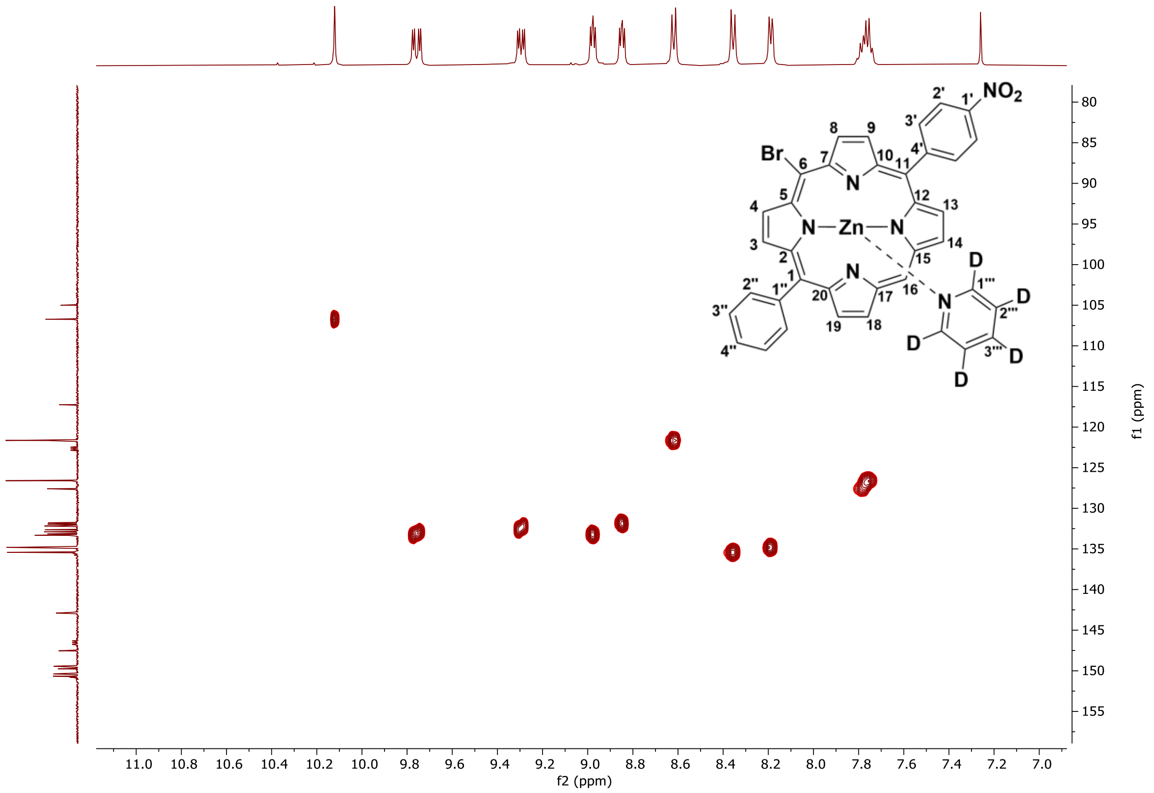

Figure S13: HMQC NMR spectrum of **3-Zn-pyrd5** in **$CDCl_3$** (500 MHz)

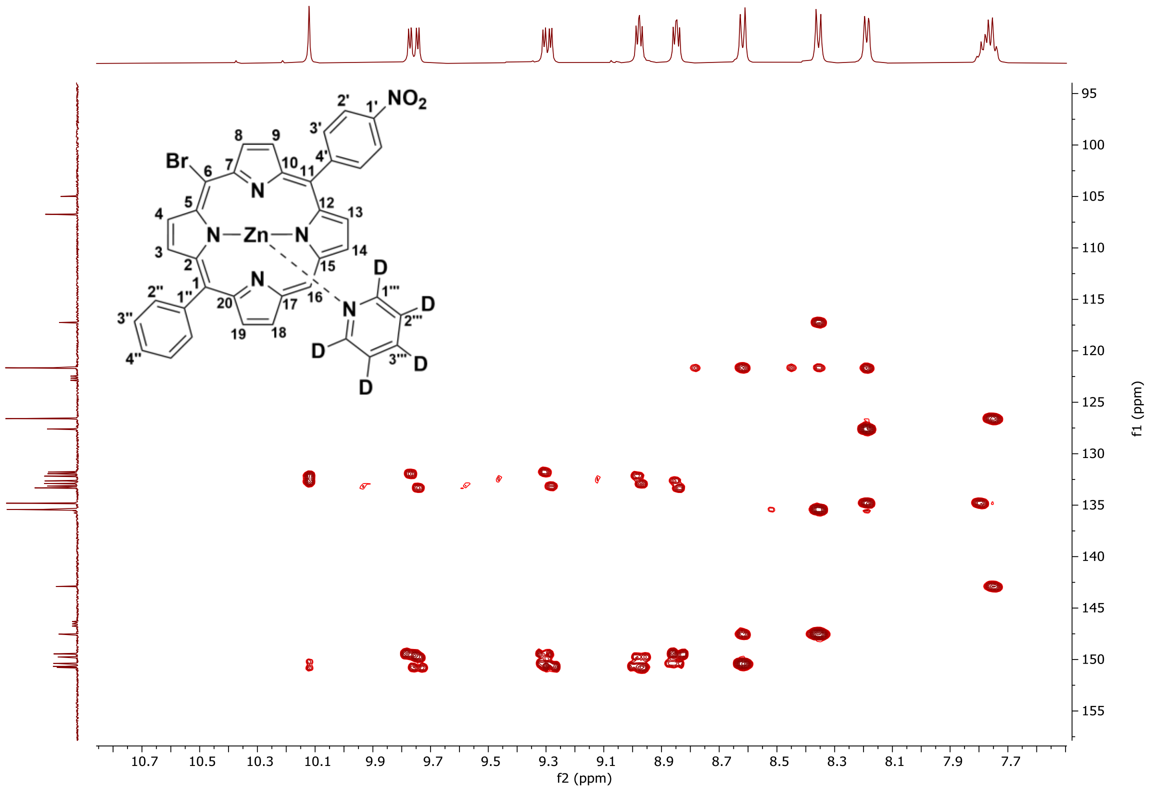

Figure S14: HMBC NMR spectrum of **3-Zn-pyrd5** in **$CDCl_3$** (500 MHz)

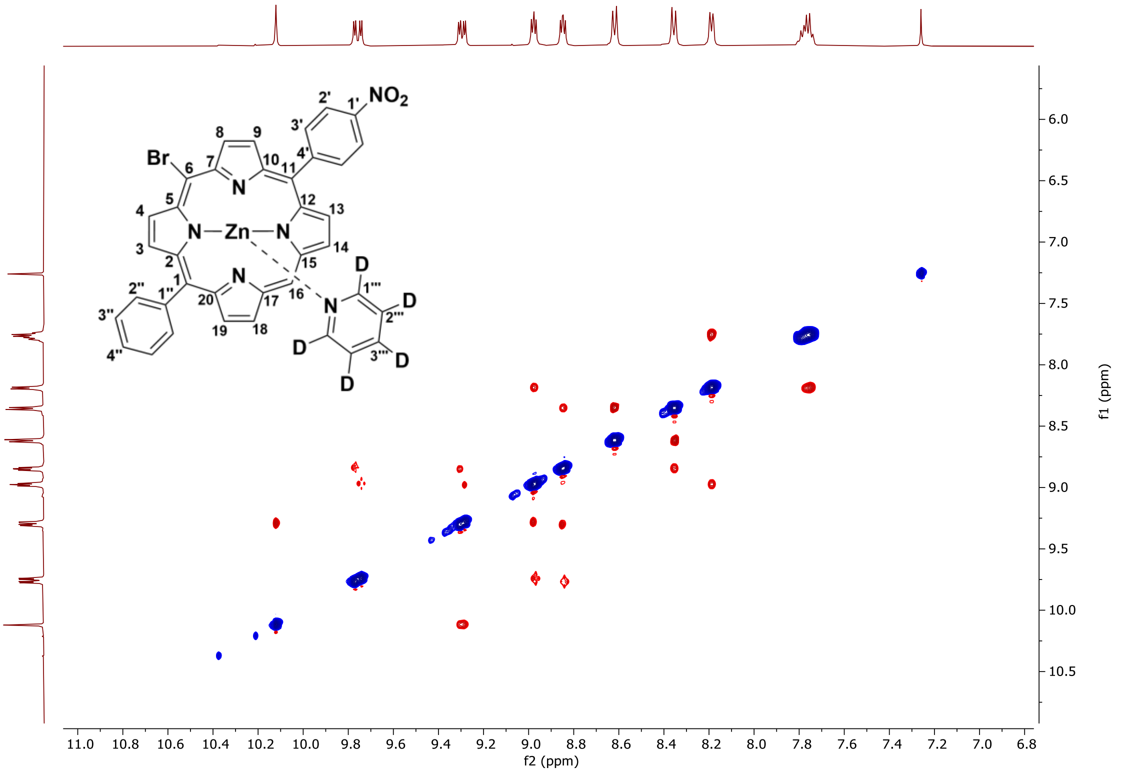

Figure S15: NOESY NMR spectrum of **3-Zn-pyrd5** in $\mathbf{CDCl_3}$ (500 MHz)

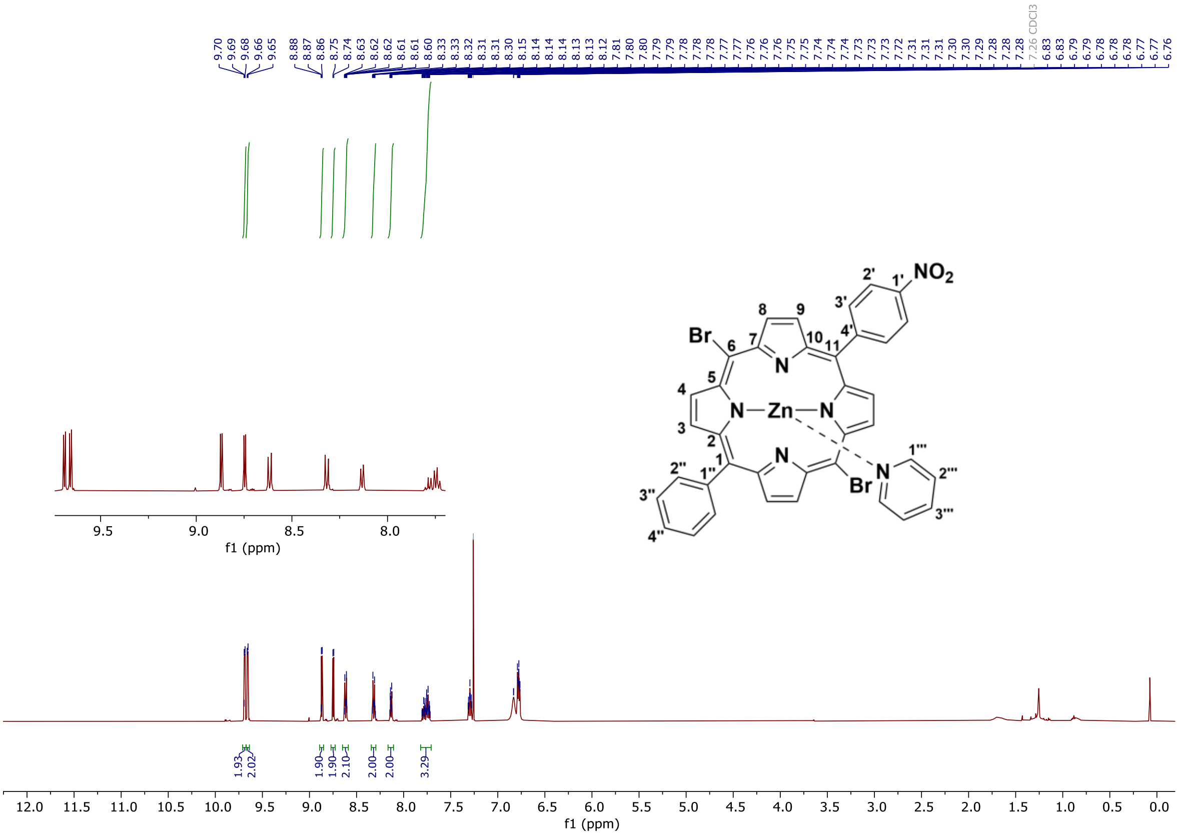

Figure S16: ^{1}H NMR spectrum of **3b-Zn-pyr** in $\mathbf{CDCl_3}$ (400 MHz)

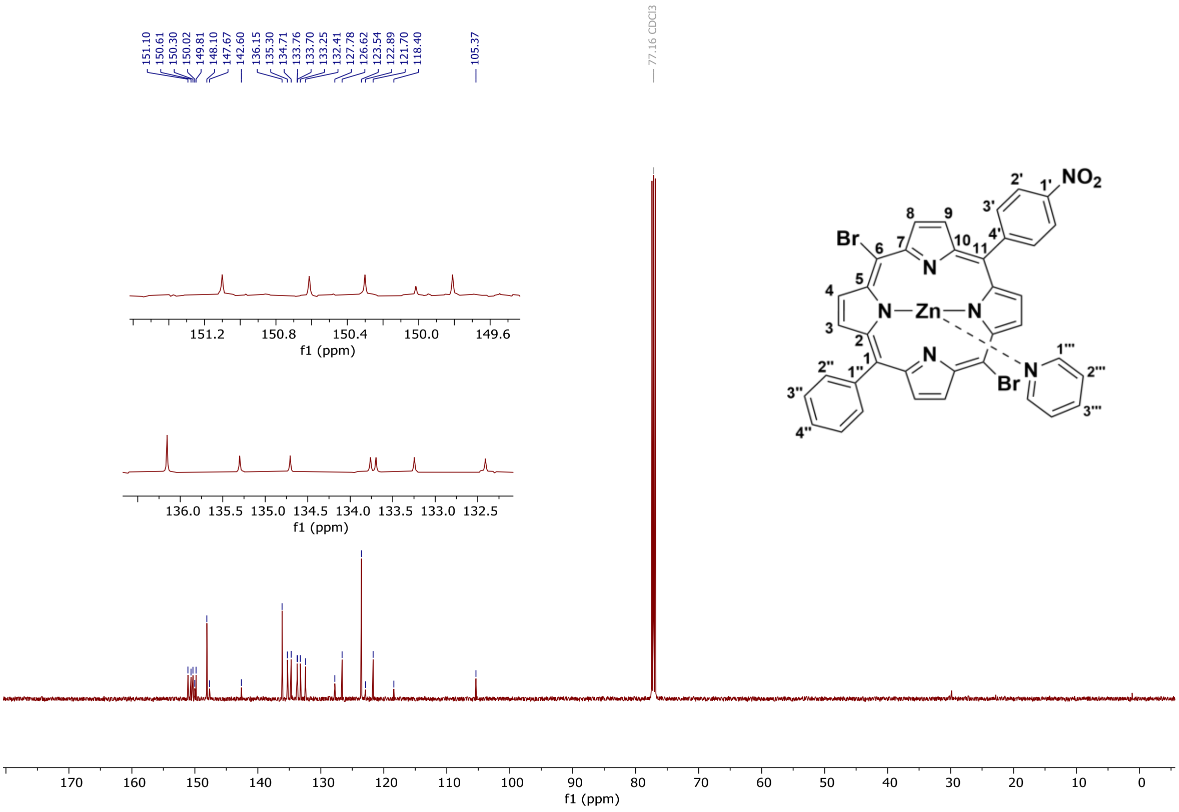

Figure S17: ^{13}C NMR spectrum of **3b-Zn-pyr** in **$CDCl_3$** (126 MHz)

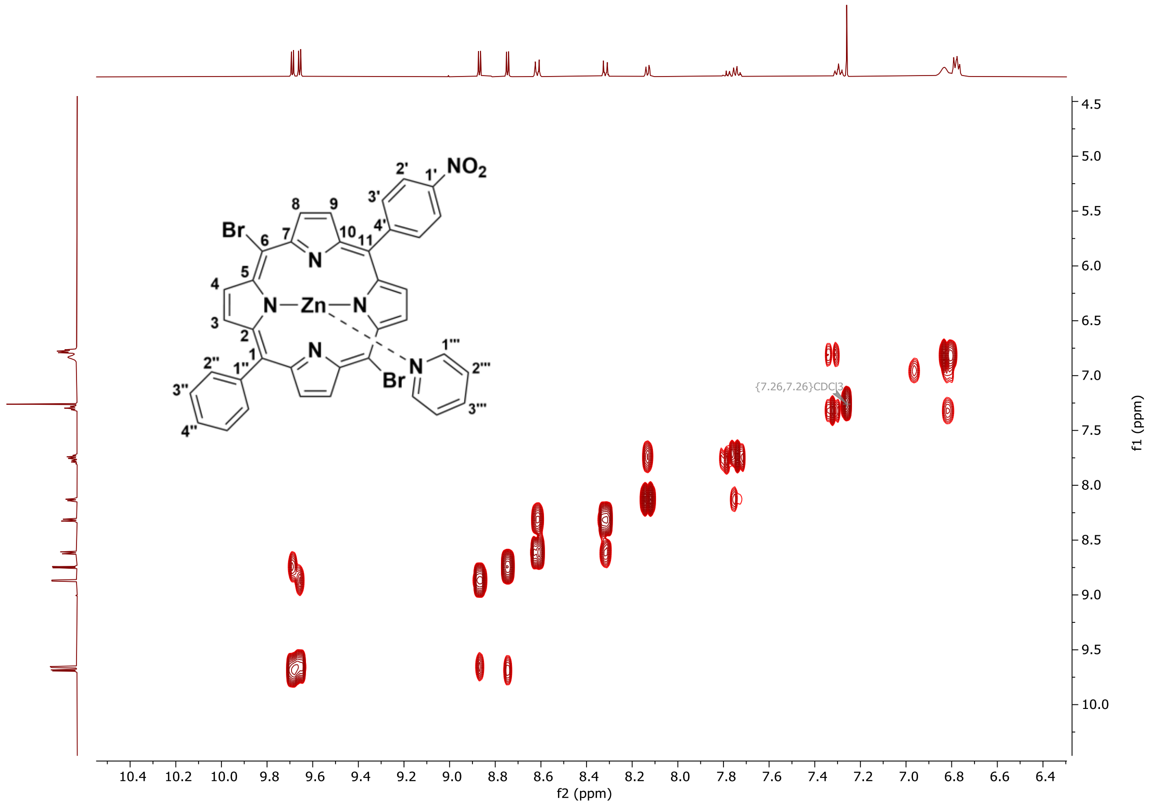

Figure S18: COSY NMR spectrum of **3b-Zn-pyr** in **$CDCl_3$** (500 MHz)

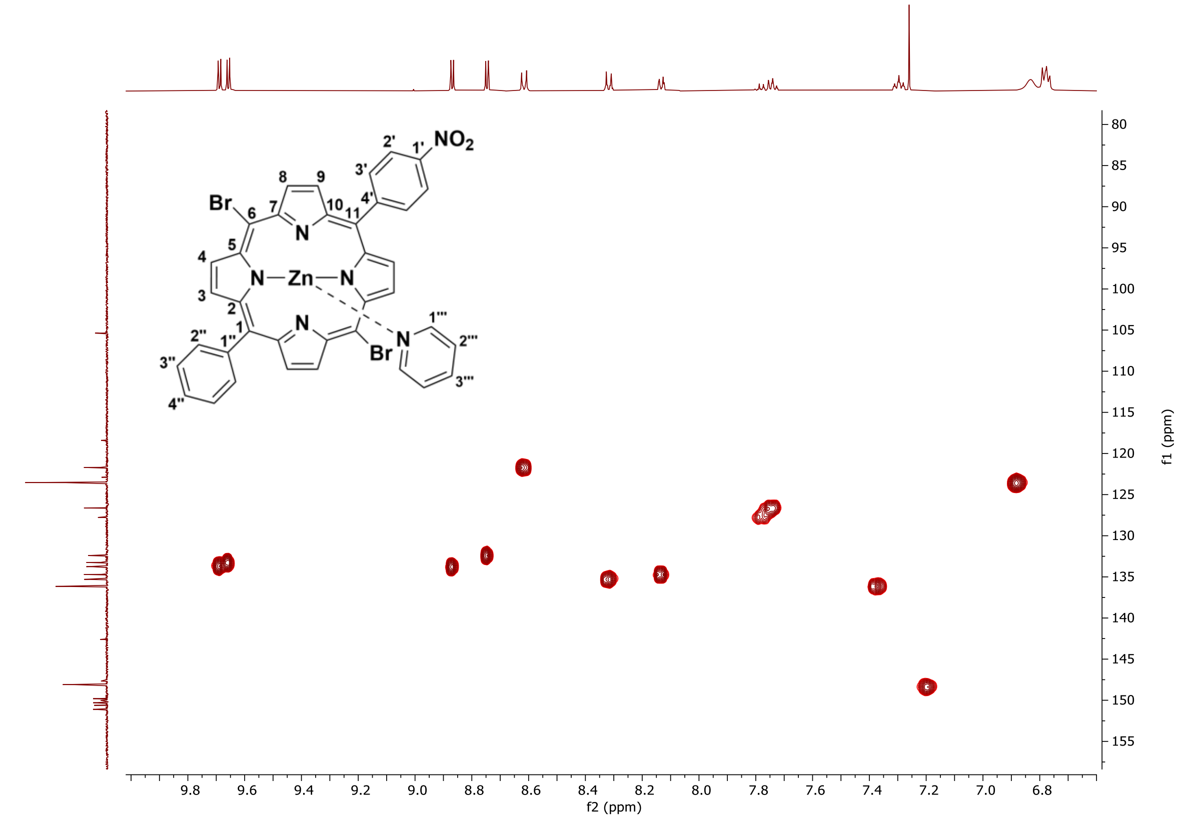

Figure S19: HMQC NMR spectrum of **3b-Zn-pyr** in **$CDCl_3$** (500 MHz)

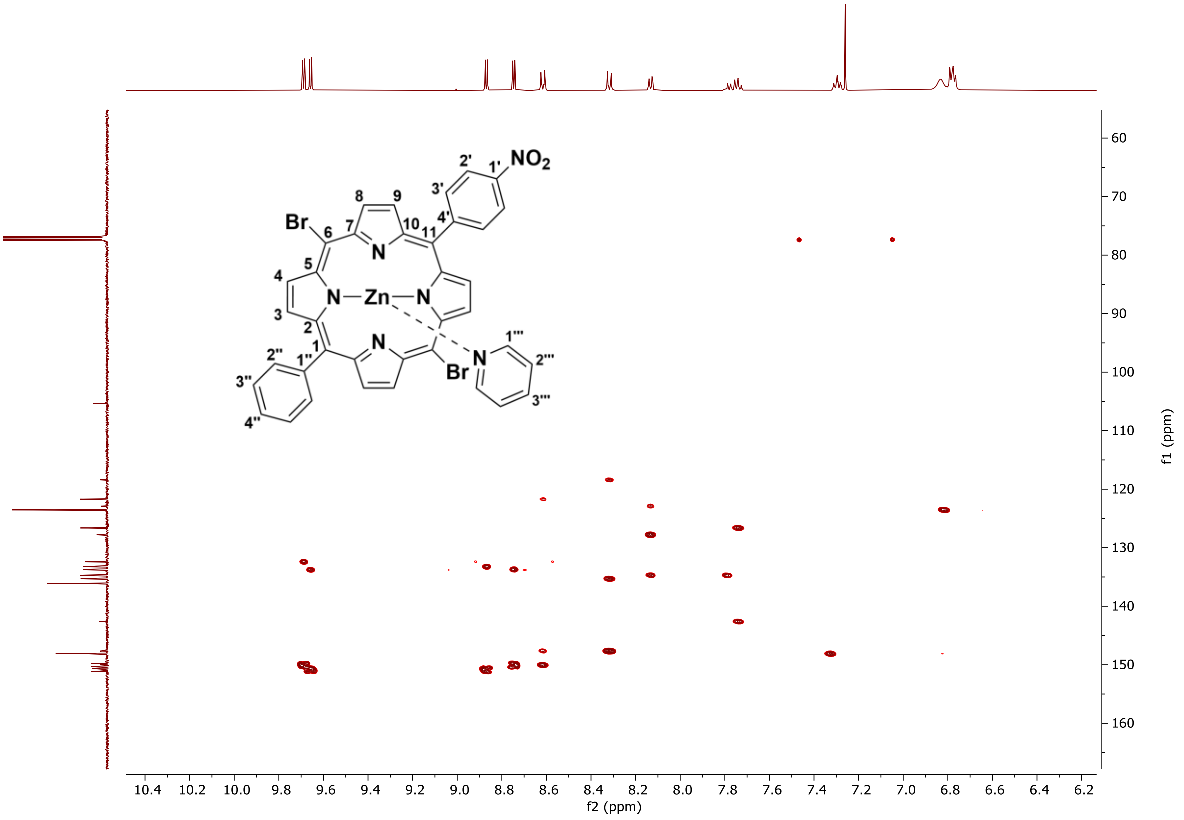

Figure S20: HMBC NMR spectrum of **3b-Zn-pyr** in **$CDCl_3$** (500 MHz), optimized for J = 10 Hz

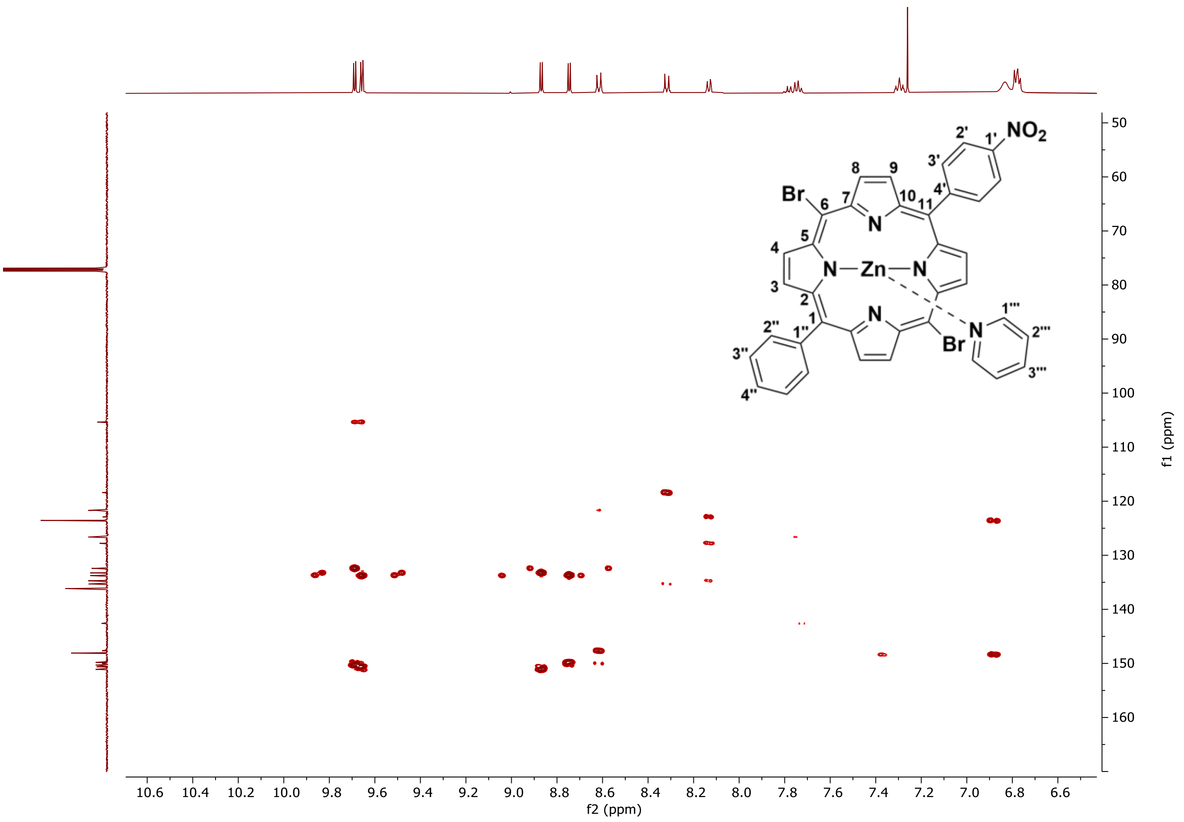

Figure S21: HMBC NMR spectrum of **3b-Zn-pyr** in **CDCl$_3$** (500 MHz), optimized for J = 5 Hz

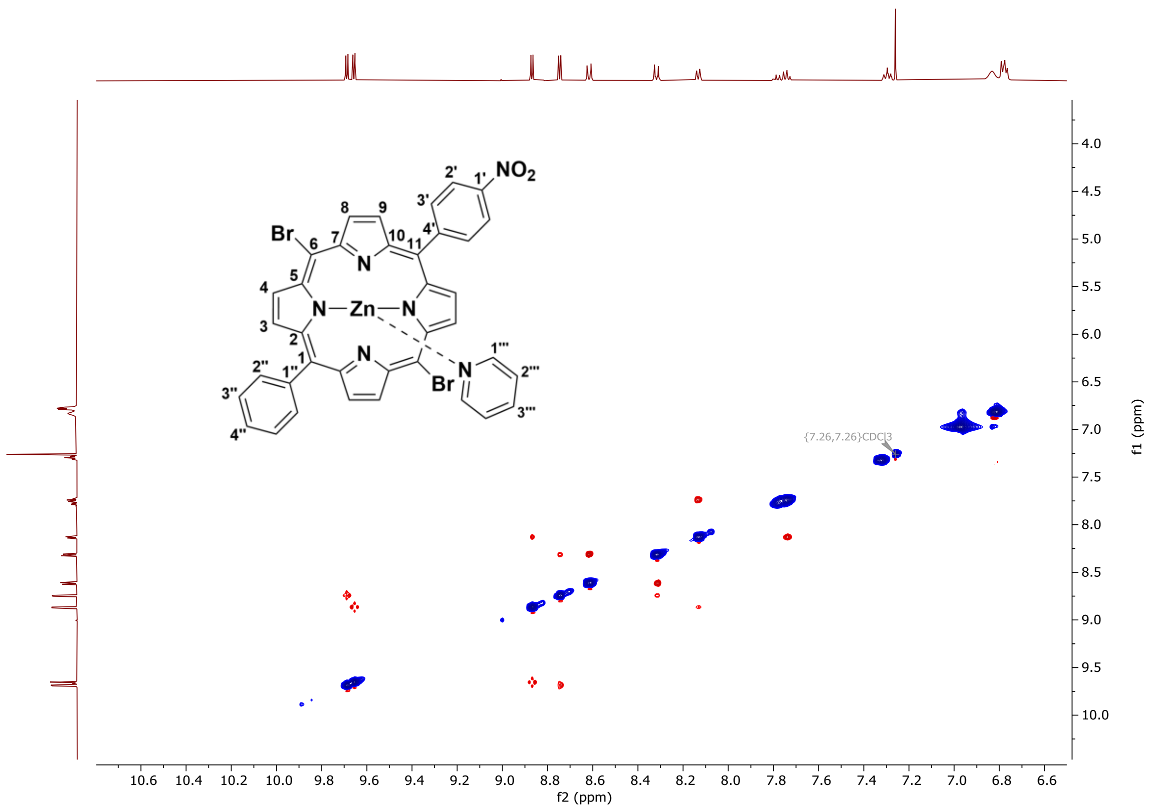

Figure S22: NOESY NMR spectrum of **3b-Zn-pyr** in **CDCl$_3$** (500 MHz)

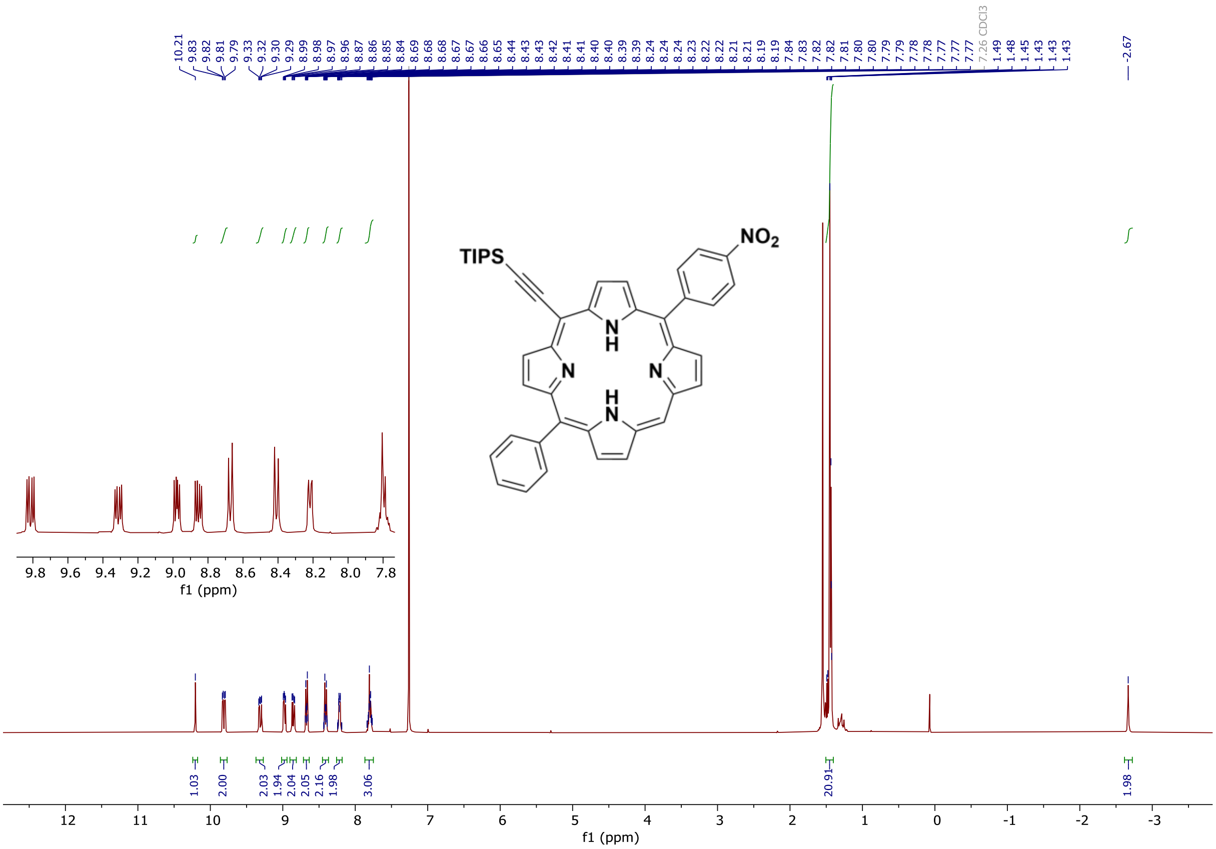

Figure S23: ^{1}H NMR spectrum of **4** in **CDCl$_3$** (400 MHz)

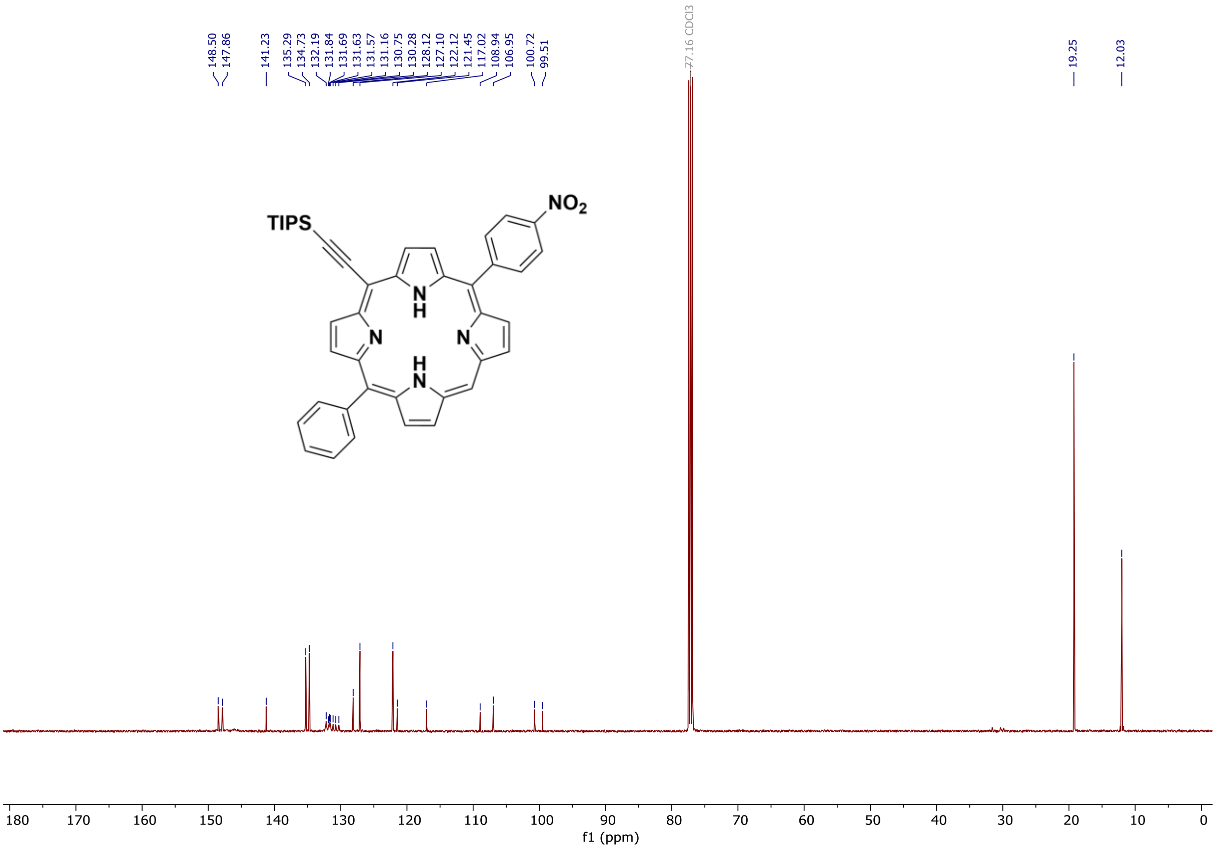

Figure S24: ^{13}C NMR spectrum of **4** in **CDCl$_3$** (126 MHz)

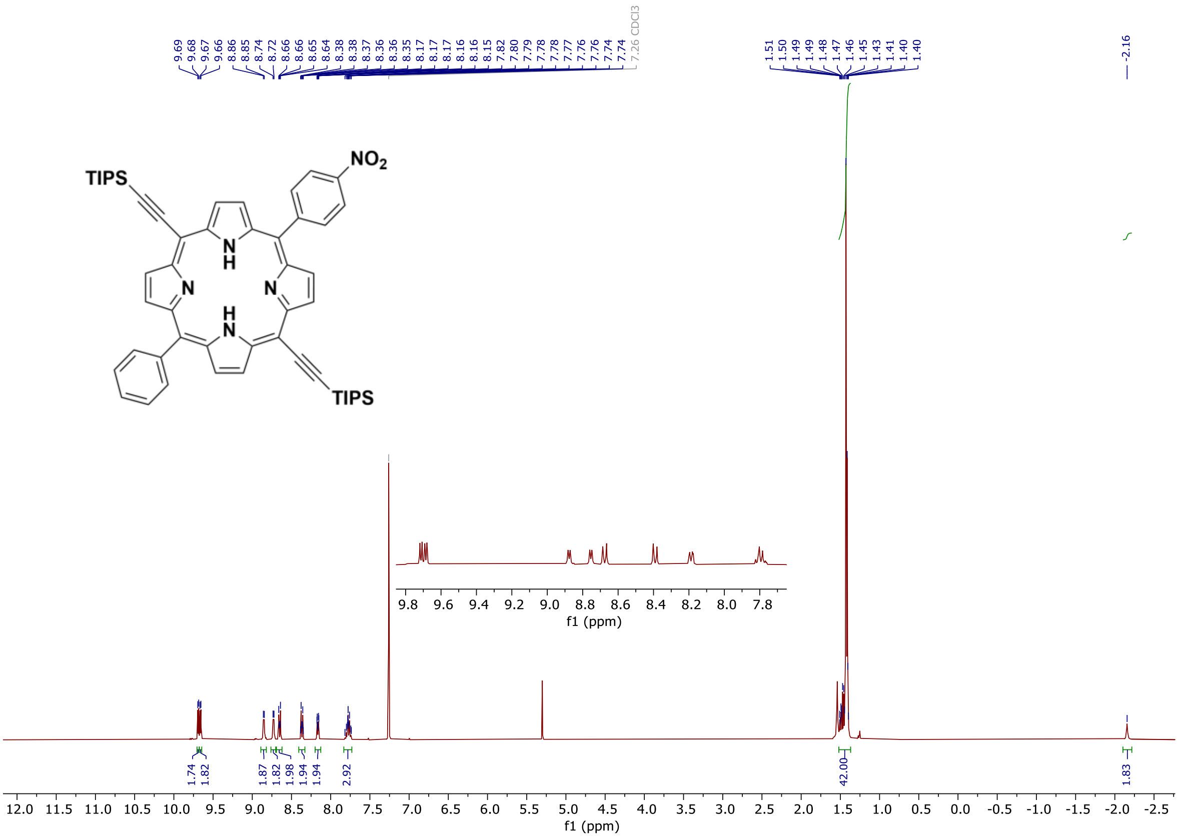

Figure S25: ^{1}H NMR spectrum of **4b** in **$CDCl_3$** (400 MHz)

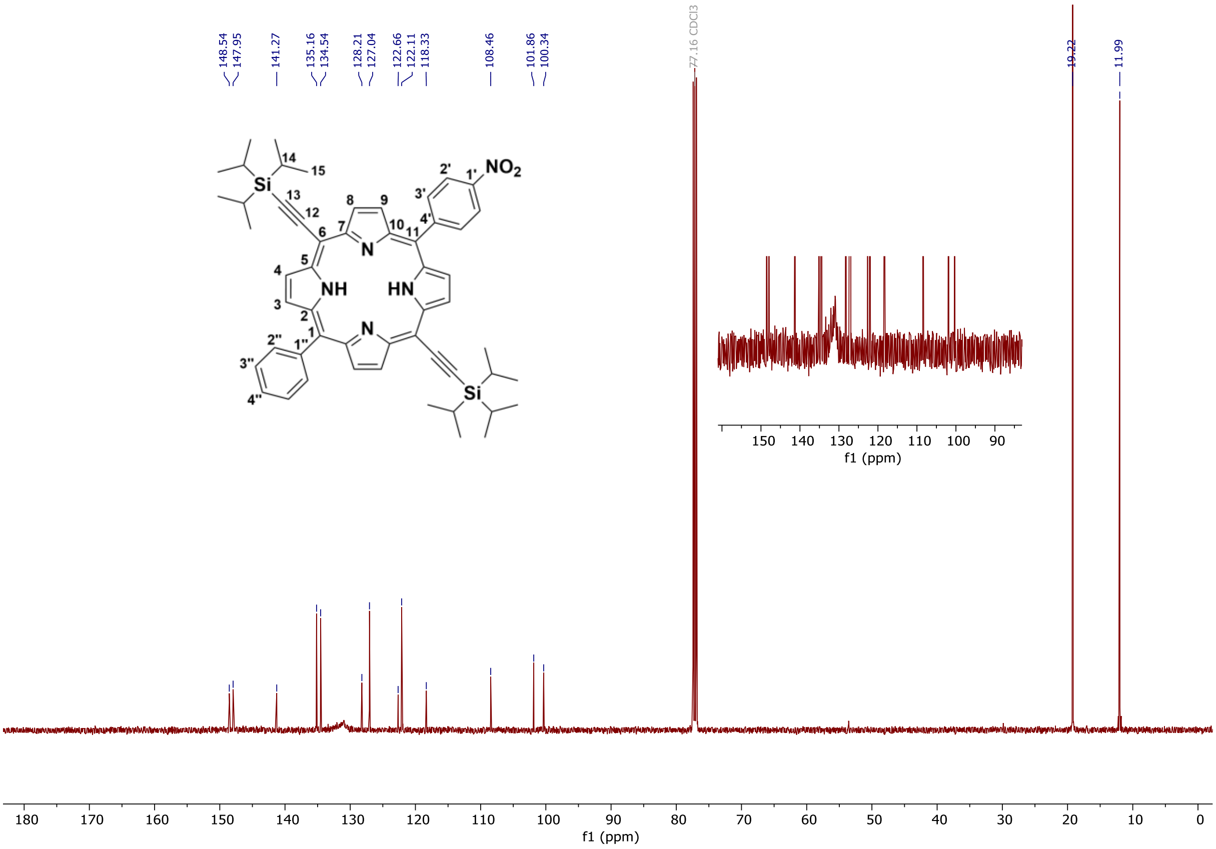

Figure S26: ^{13}C NMR spectrum of **4b** in **$CDCl_3$** (126 MHz). β-pyrrole carbons are visible as a single broad feature between 130 – 133 ppm, α-pyrrole carbons are not visible at all.

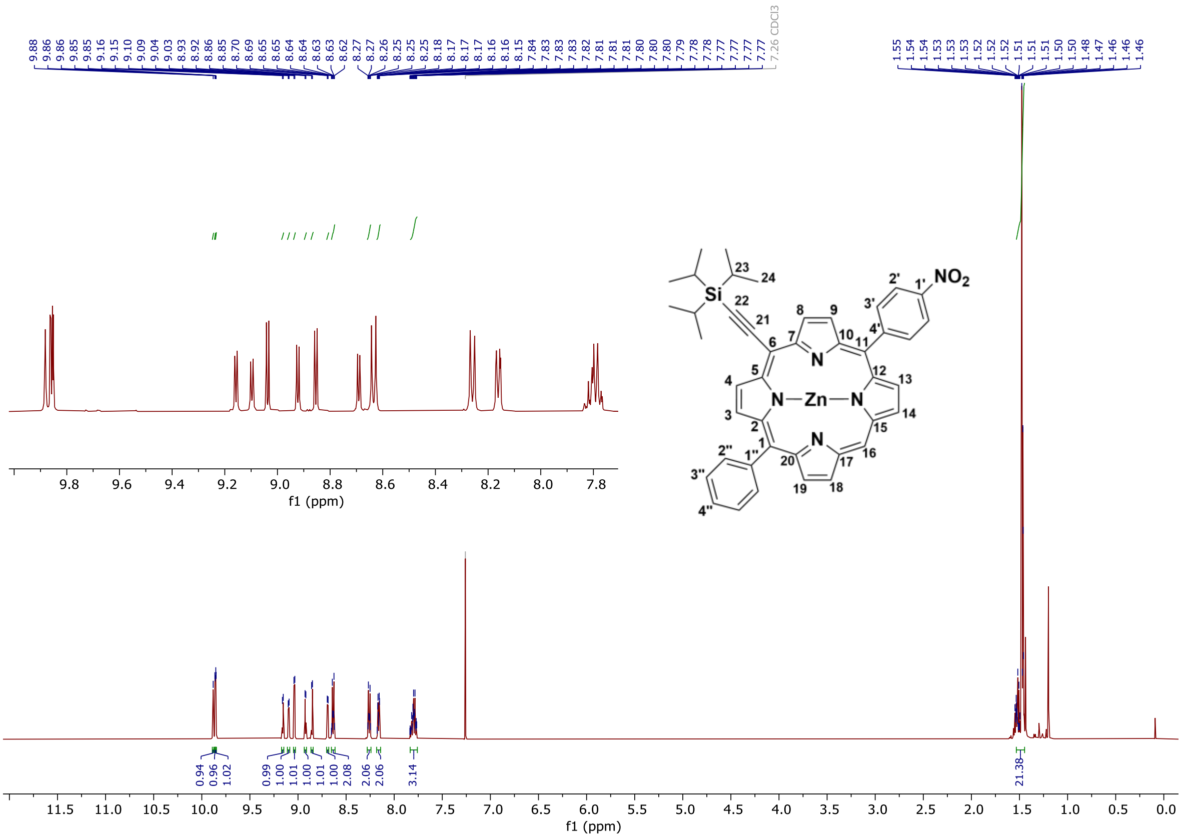

Figure S27: ^{1}H NMR spectrum of **4-Zn** in **$CDCl_3$** (500 MHz)

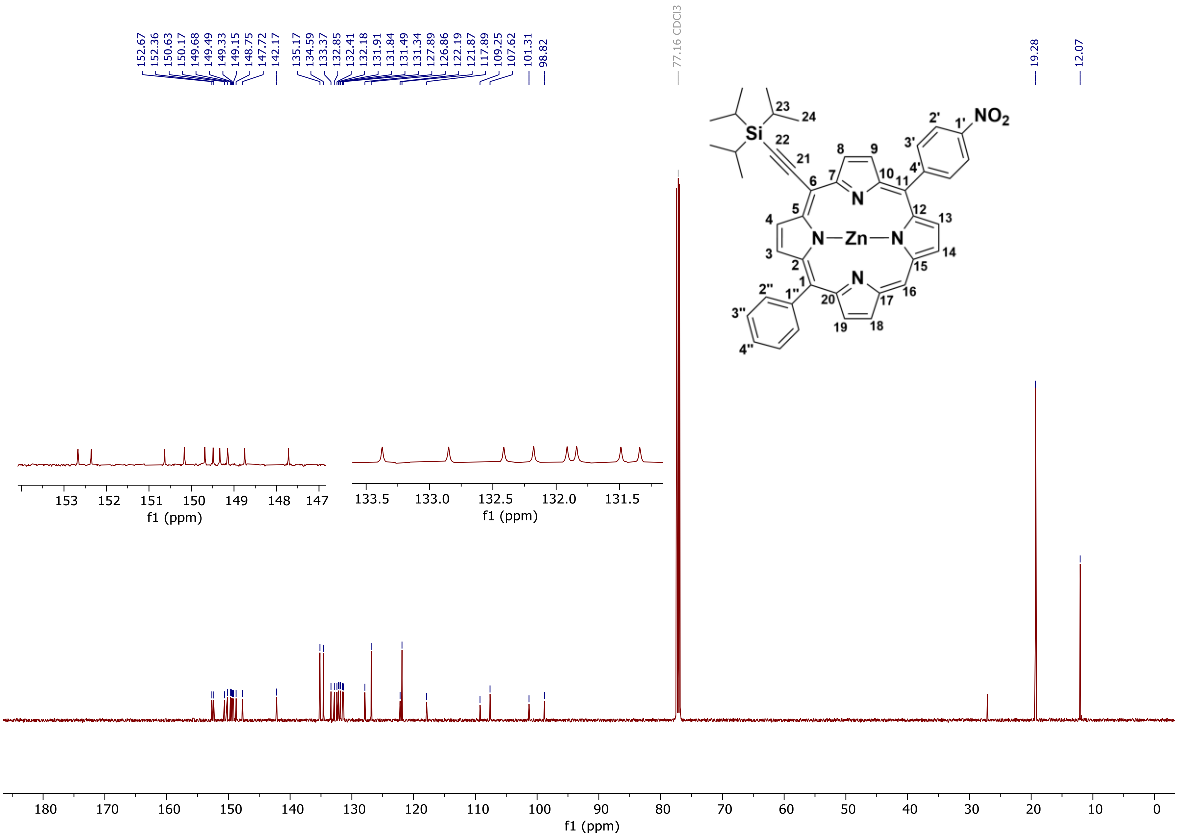

Figure S28: ^{13}C NMR spectrum of **4-Zn** in **$CDCl_3$** (126 MHz)

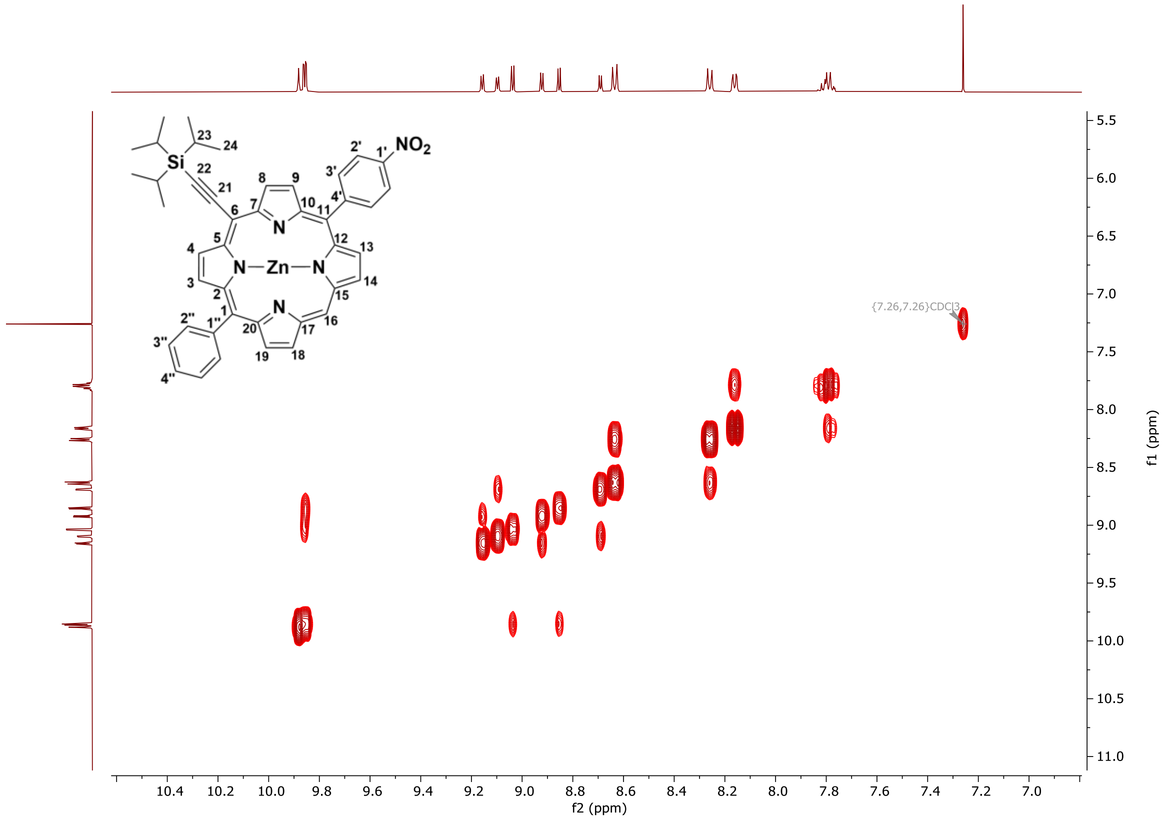

Figure S29: COSY NMR spectrum of **4-Zn** in **CDCl$_3$** (500 MHz)

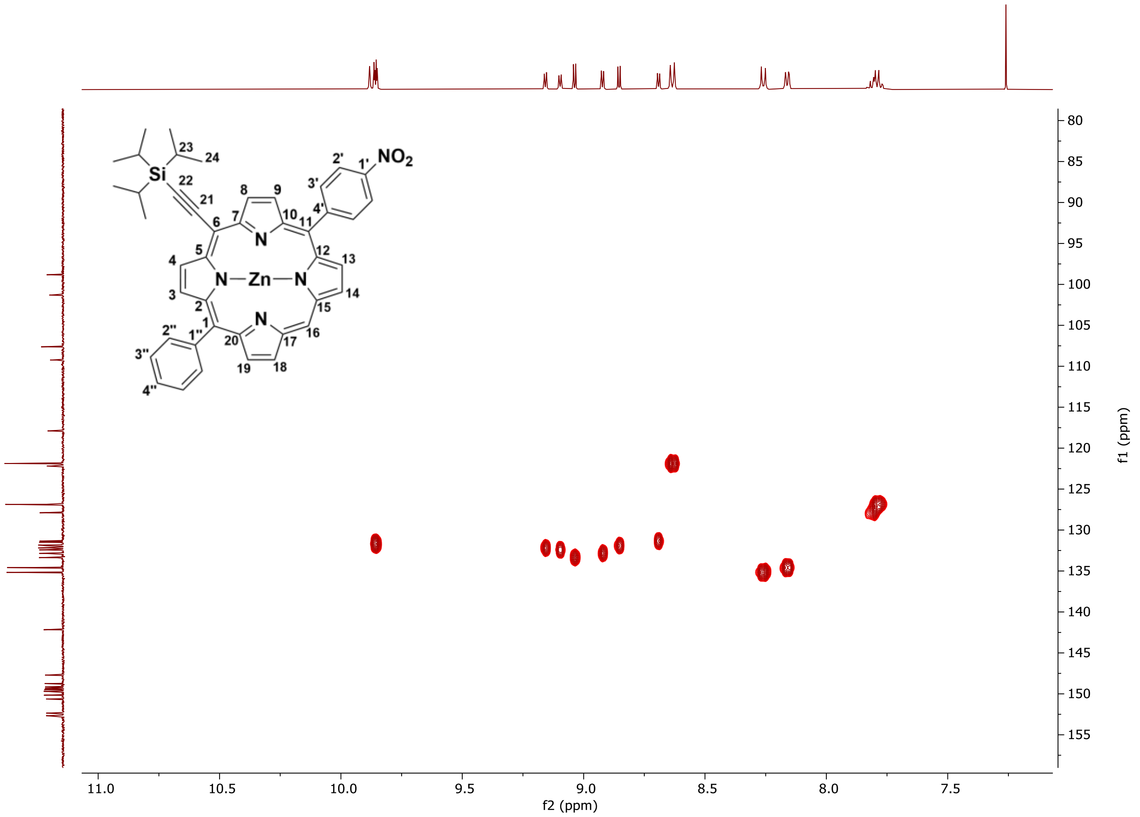

Figure S30: HMQC NMR spectrum of **4-Zn** in **CDCl$_3$** (500 MHz)

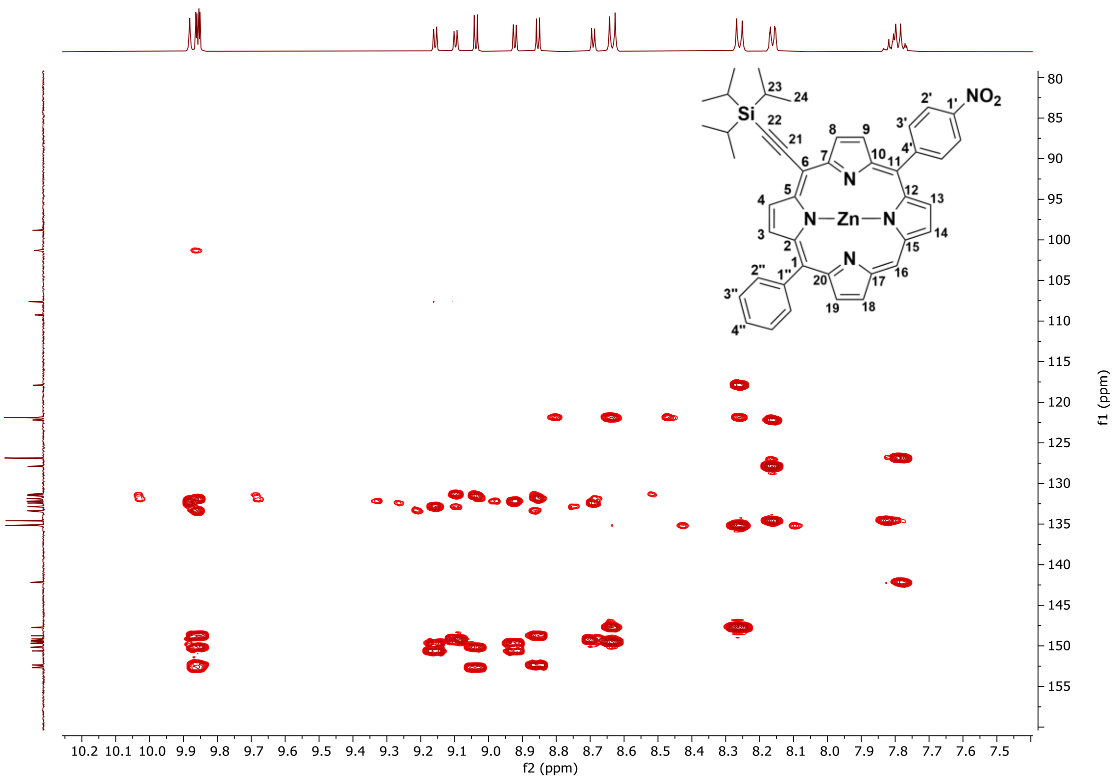

Figure S31: HMBC NMR spectrum of **4-Zn** in **CDCl$_3$** (500 MHz), optimized for J = 10 Hz

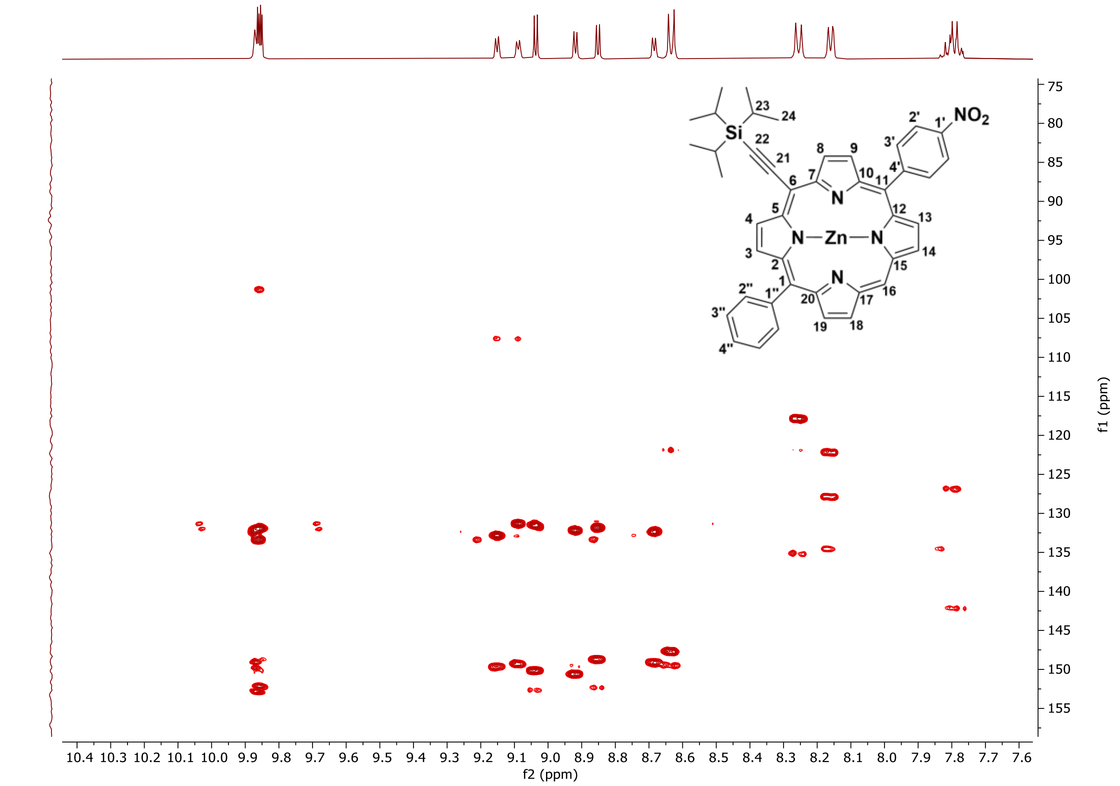

Figure S32: HMBC NMR spectrum of **4-Zn** in **CDCl$_3$** (500 MHz), optimized for J = 5 Hz

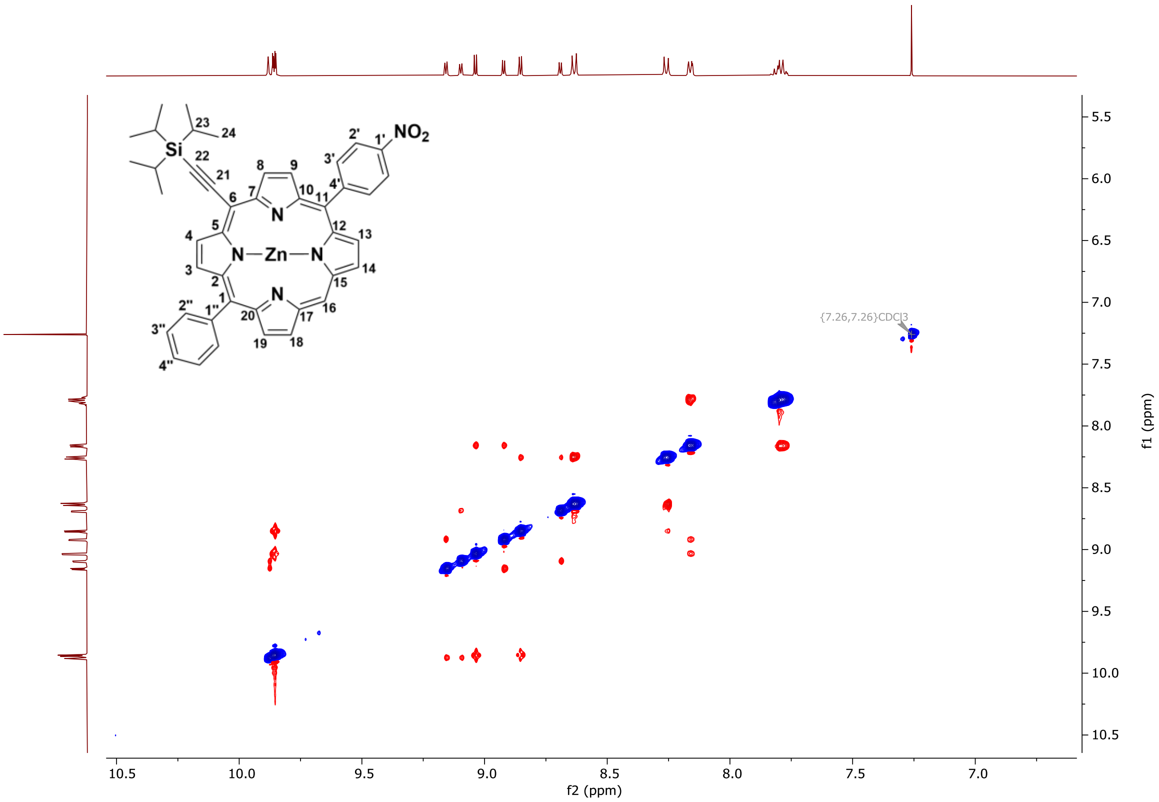

Figure S33: NOESY NMR spectrum of **4-Zn** in **CDCl$_3$** (500 MHz)

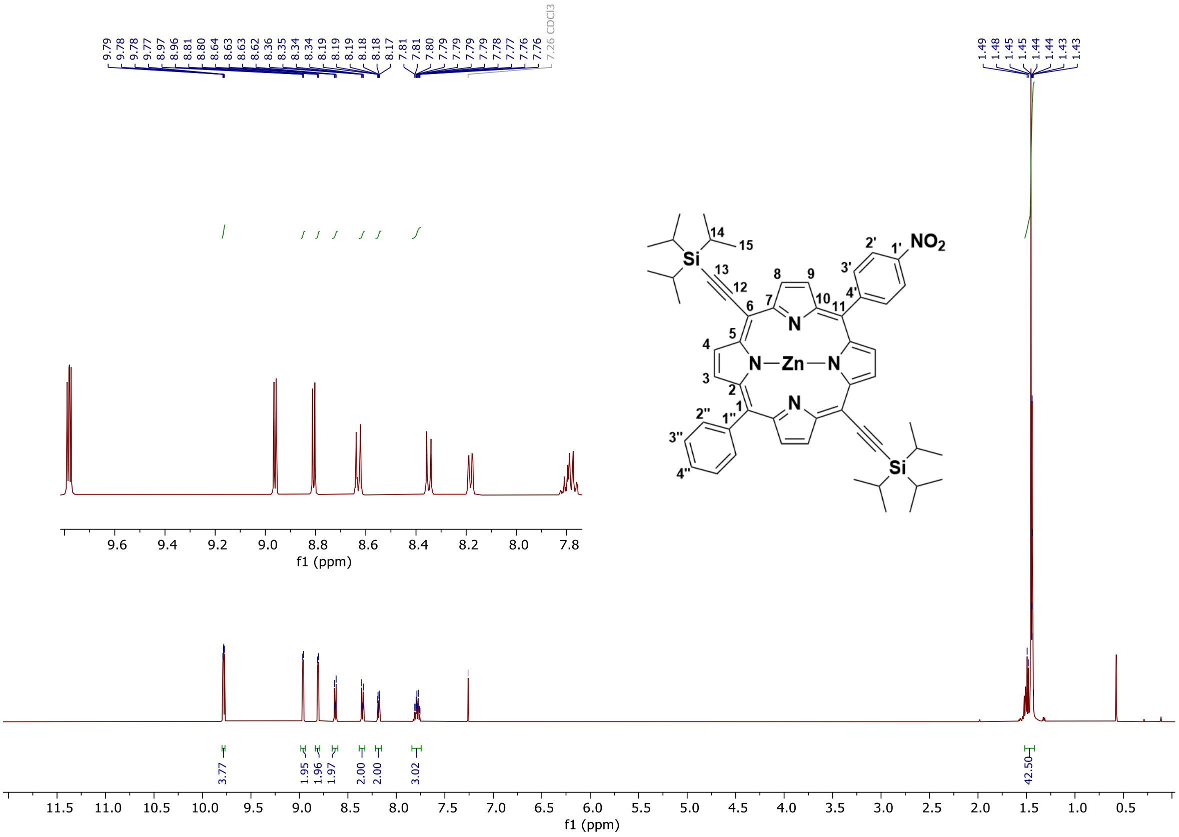

Figure S34: ^{1}H NMR spectrum of **4b-Zn** in **CDCl$_3$** (500 MHz).

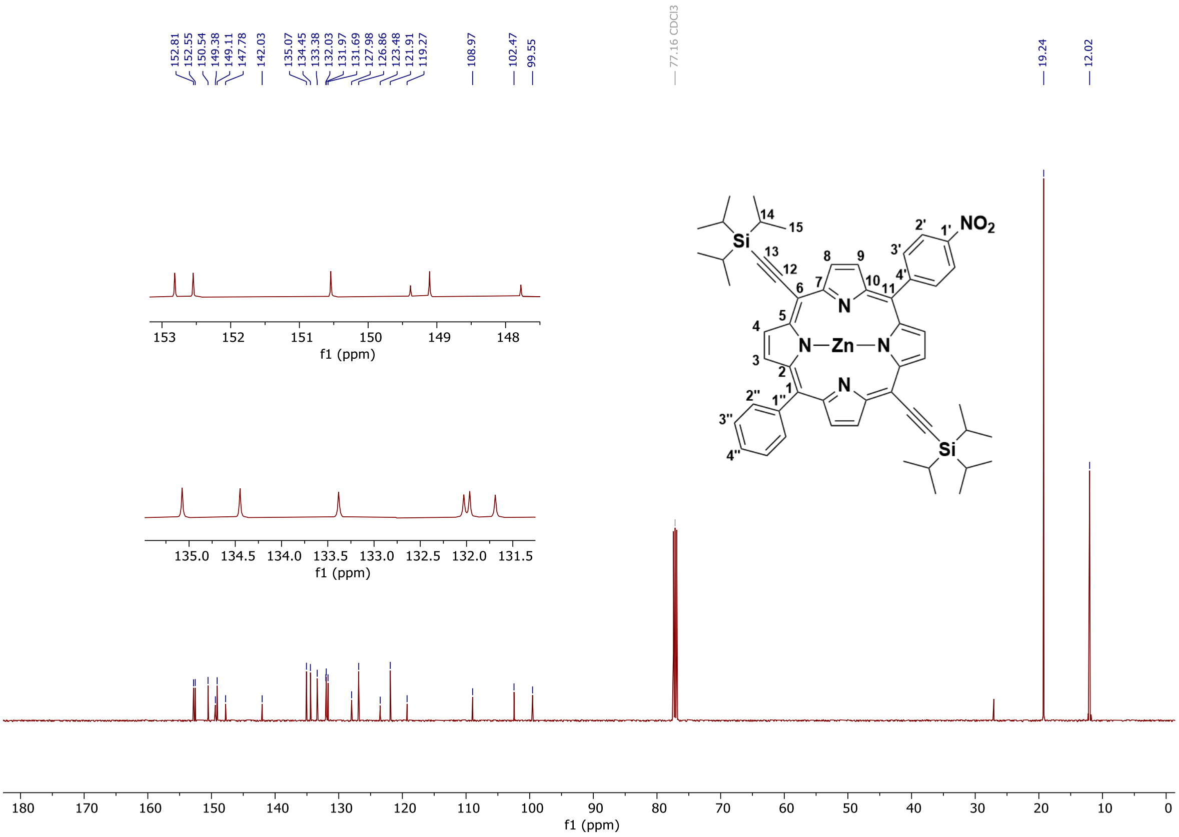

Figure S35: ^{13}**C NMR** spectrum of **4b-Zn** in **$CDCl_3$** (126 MHz)

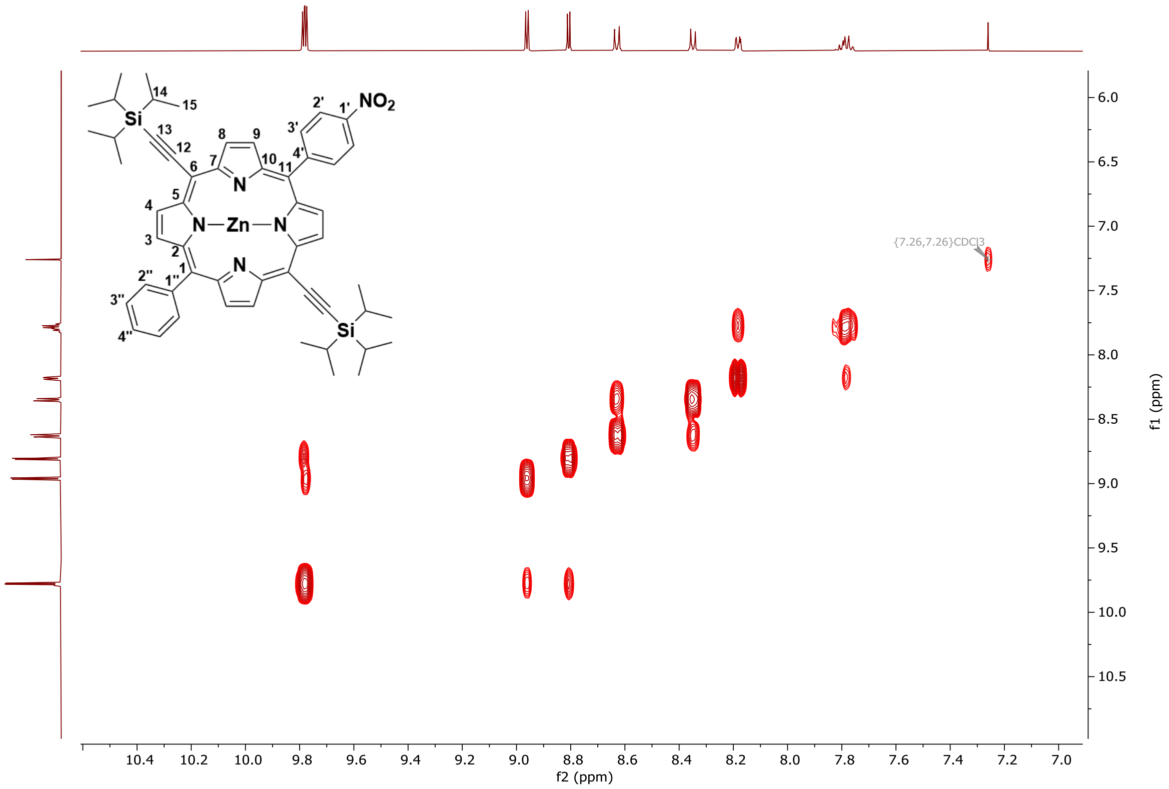

Figure S36: COSY NMR spectrum of **4b-Zn** in **$CDCl_3$** (500 MHz)

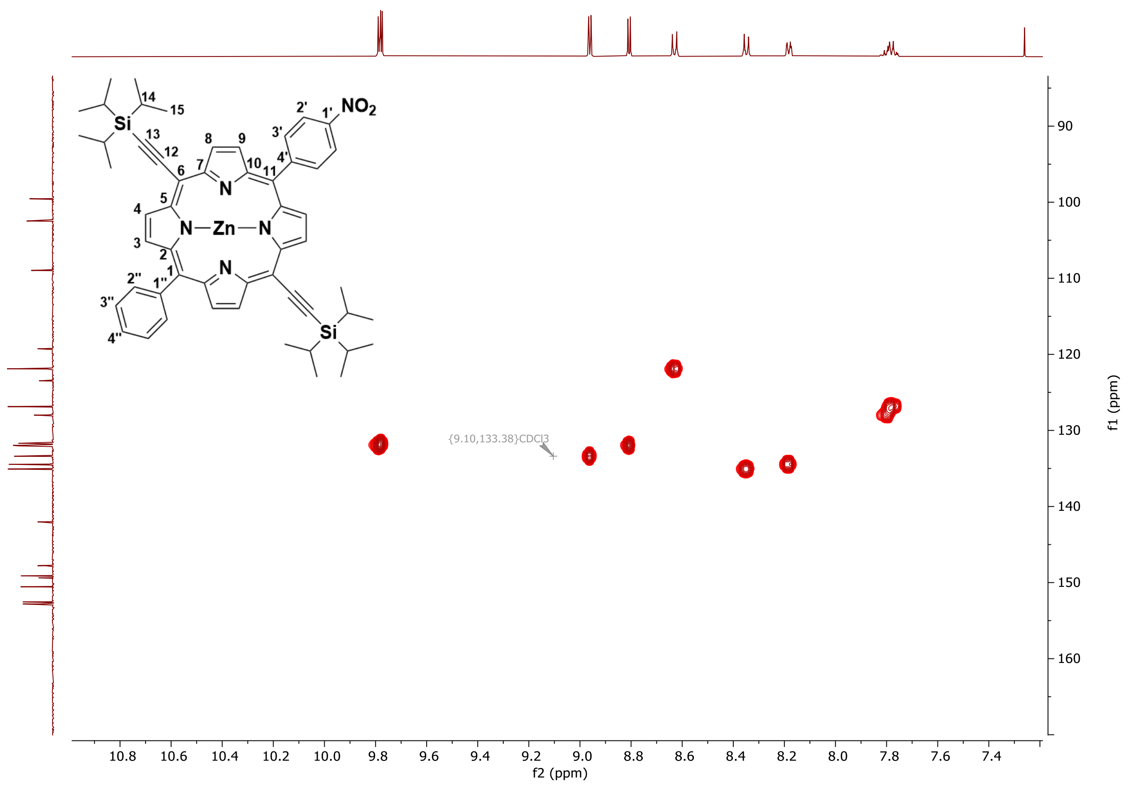

Figure S37: HMQC NMR spectrum of **4b-Zn** in **CDCl$_3$** (500 MHz)

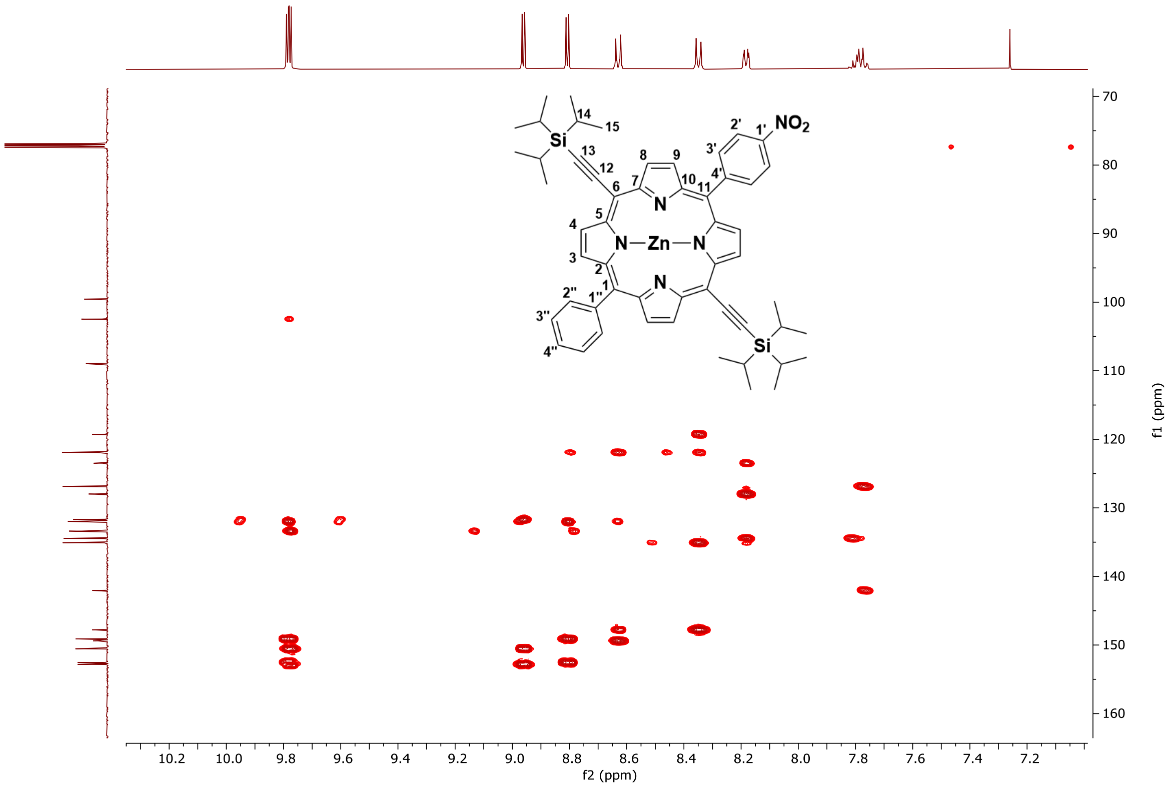

Figure S38: HMBC NMR spectrum of **4b-Zn** in **CDCl$_3$** (500 MHz), optimized for J = 10 Hz

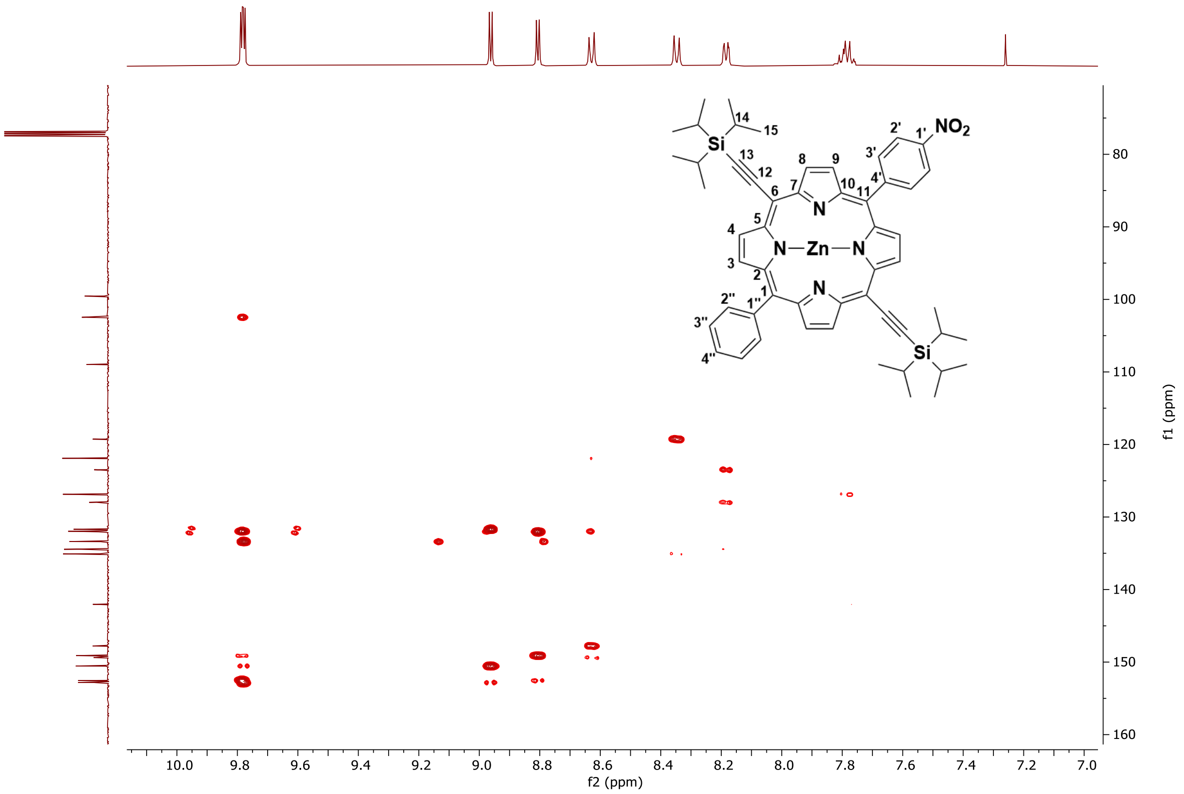

Figure S39: HMBC NMR spectrum of **4b-Zn** in **CDCl$_3$** (500 MHz), optimized for J = 5 Hz

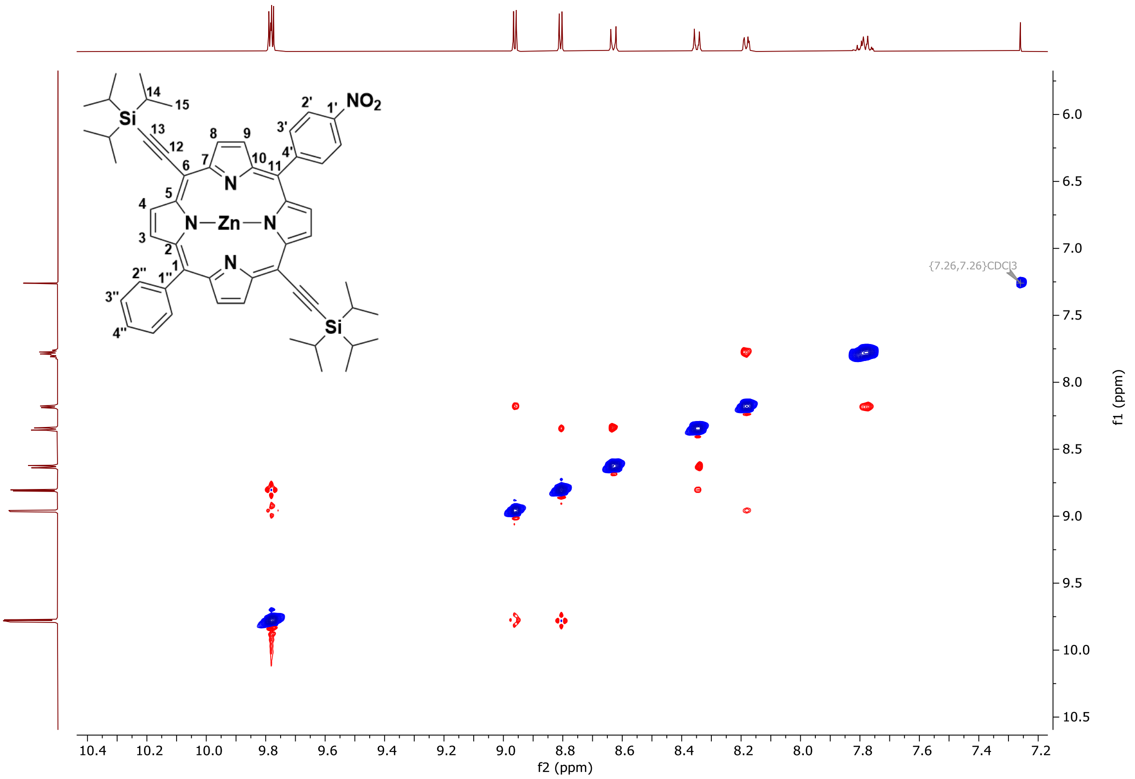

Figure S40: NOESY NMR spectrum of **4b-Zn** in **CDCl$_3$** (500 MHz)

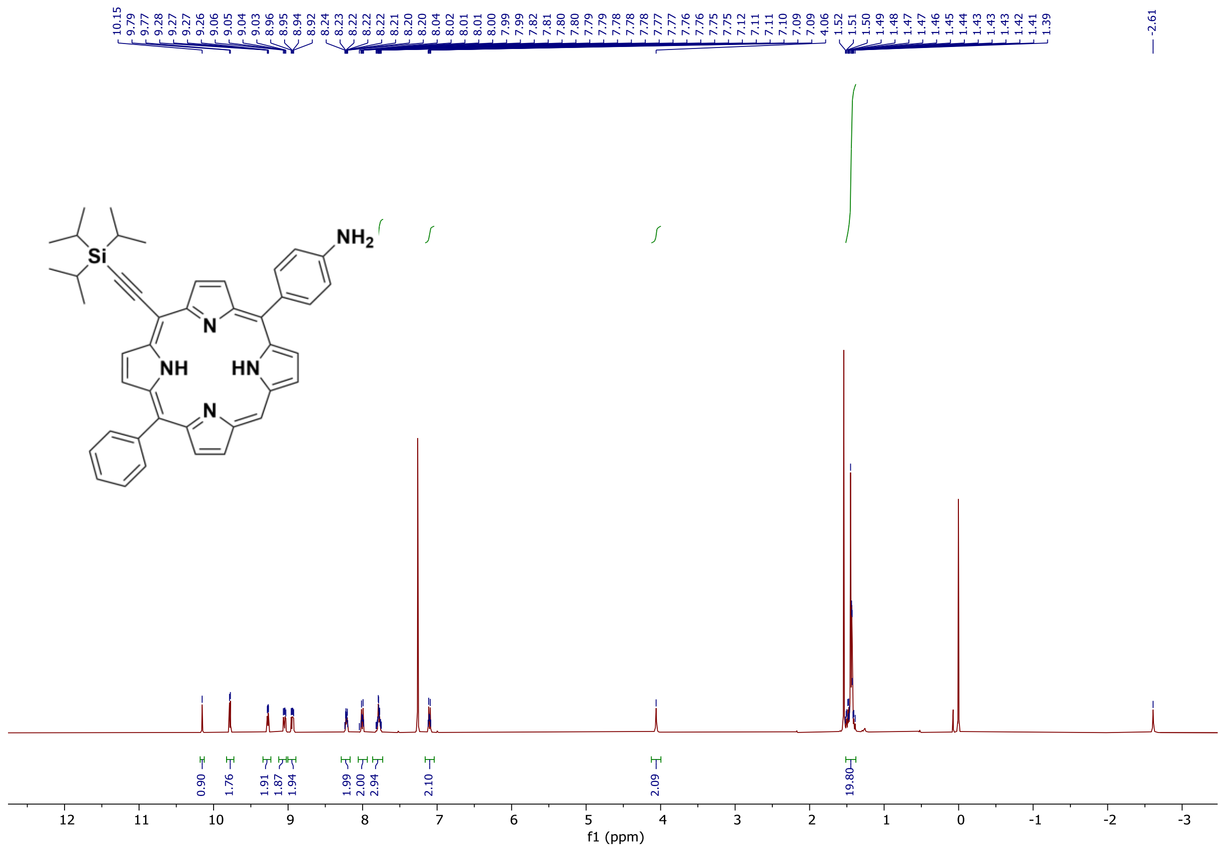

Figure S41: **^{1}H NMR** spectrum of **5** in **$CDCl_3$** (400 MHz)

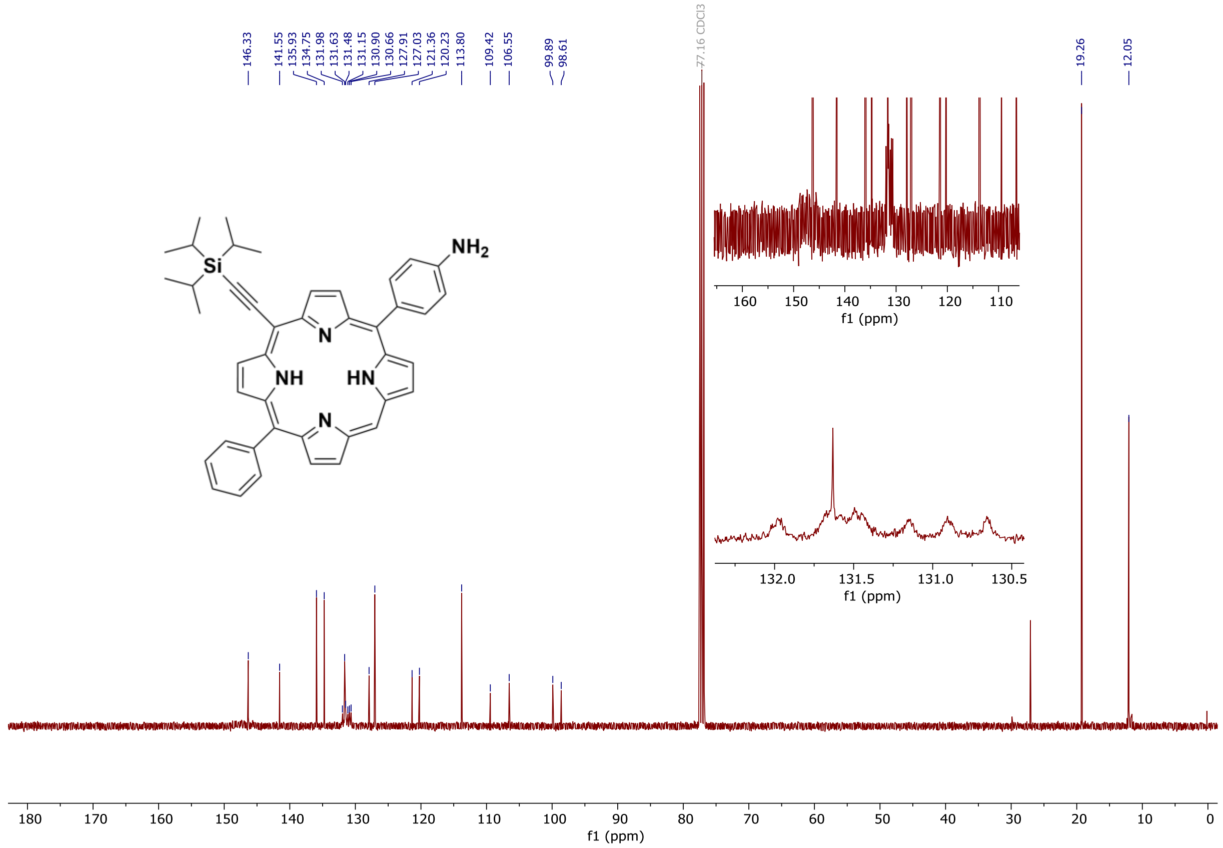

Figure S42: **^{13}C NMR** spectrum of **5** in **$CDCl_3$** (101 MHz). β-pyrrole carbons are broadened but still resolved between 130 – 132 ppm, α-pyrrole carbons are visible as a single broad feature between 145 – 150 ppm.

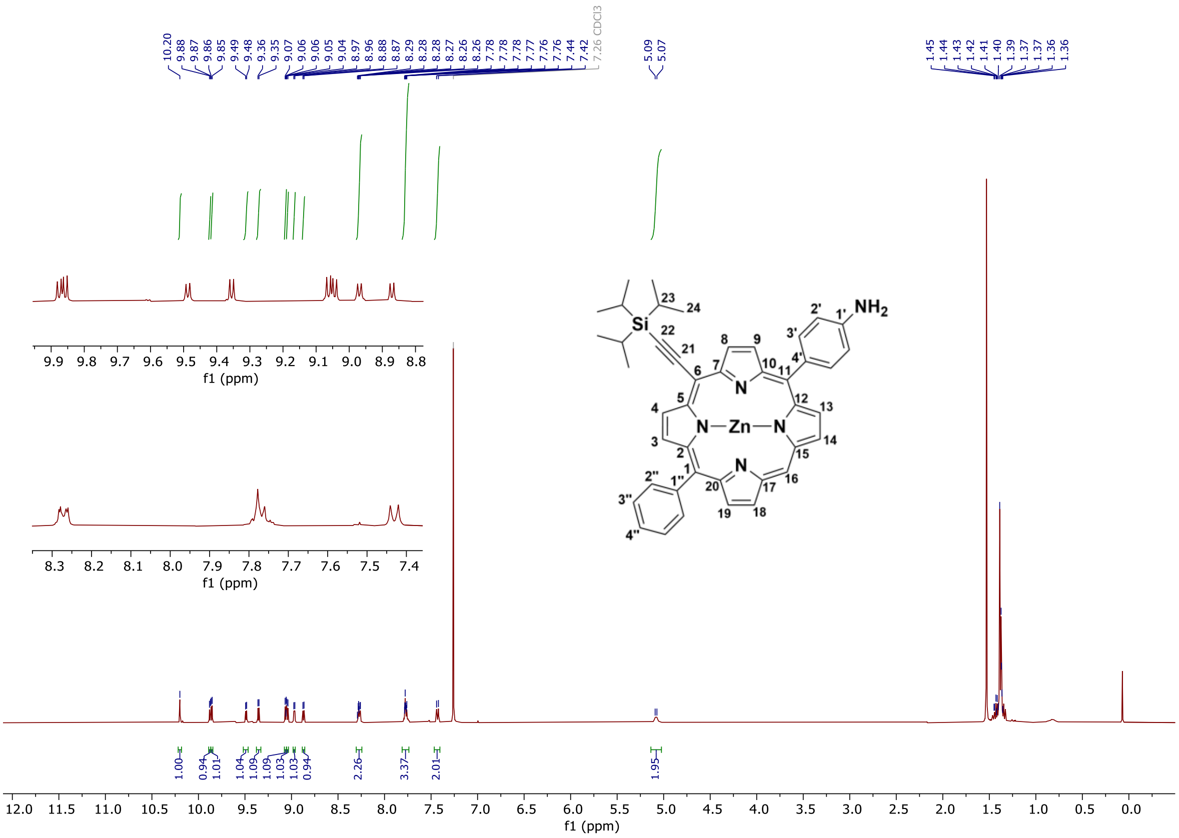

Figure S43: ^{1}**H NMR** spectrum of **5-Zn** in **CDCl$_3$** (400 MHz)

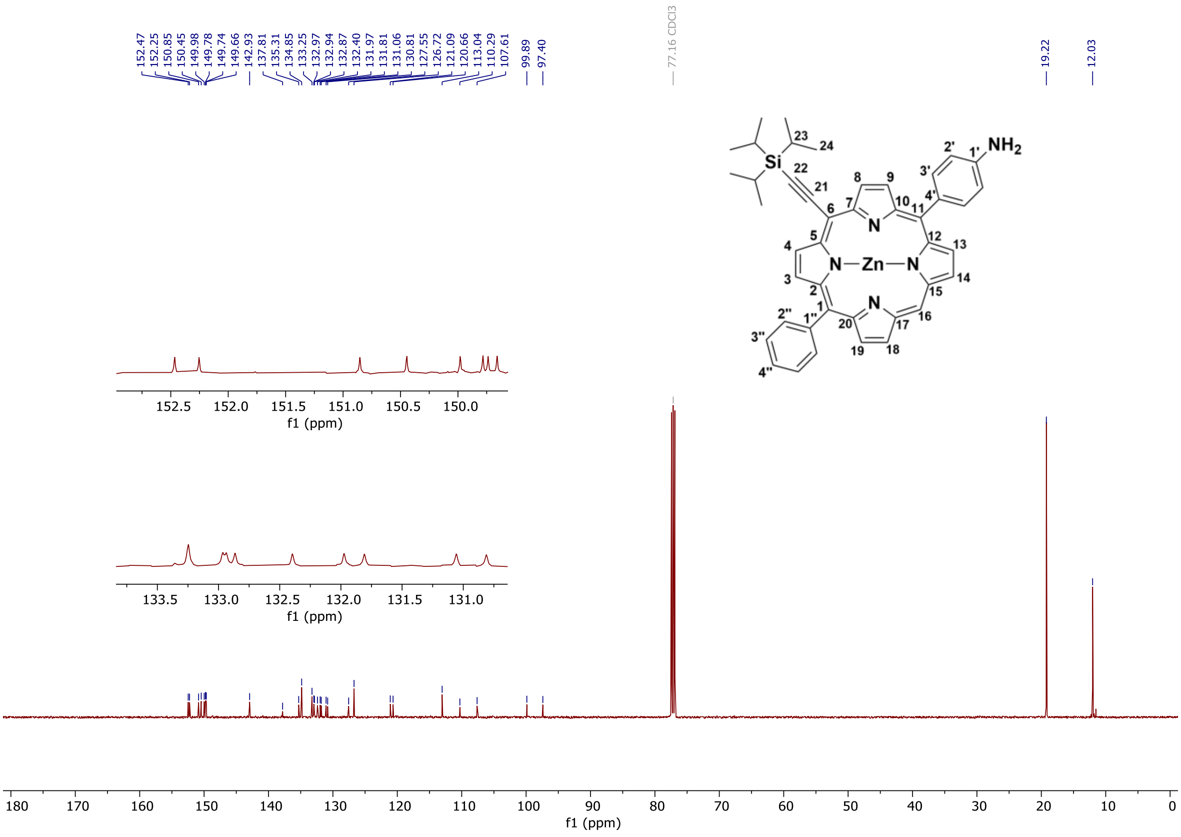

Figure S44: ^{13}**C NMR** spectrum of **5-Zn** in **CDCl$_3$** (126 MHz)

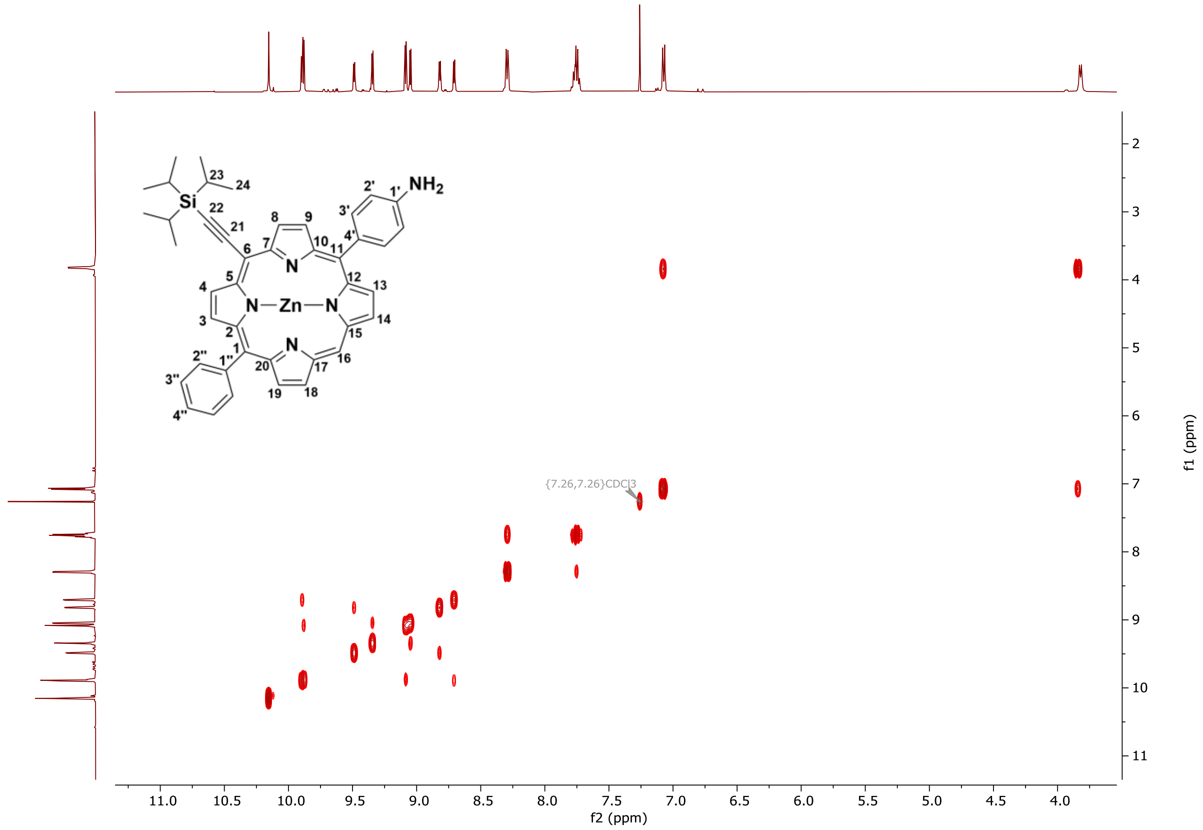

Figure S45: COSY NMR spectrum of **5-Zn** in **CDCl$_3$** (500 MHz)

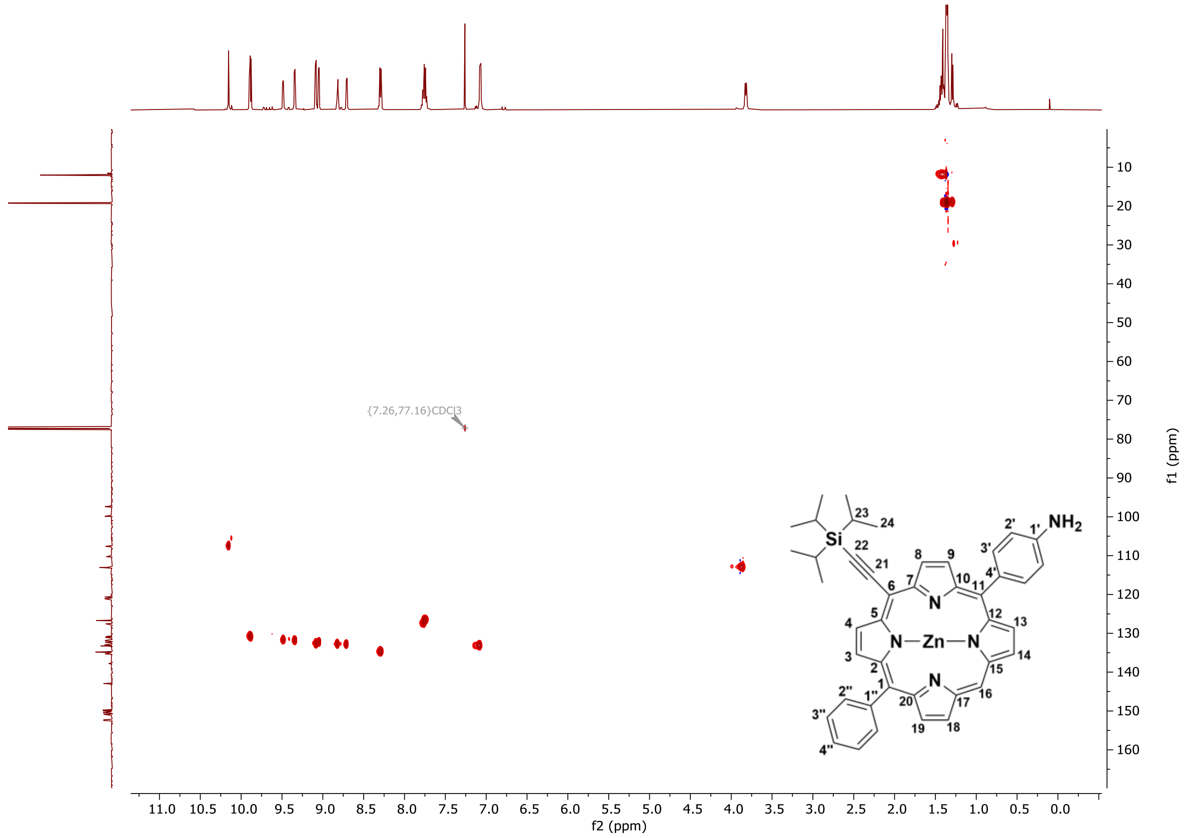

Figure S46: HMQC NMR spectrum of **5-Zn** in **CDCl$_3$** (500 MHz)

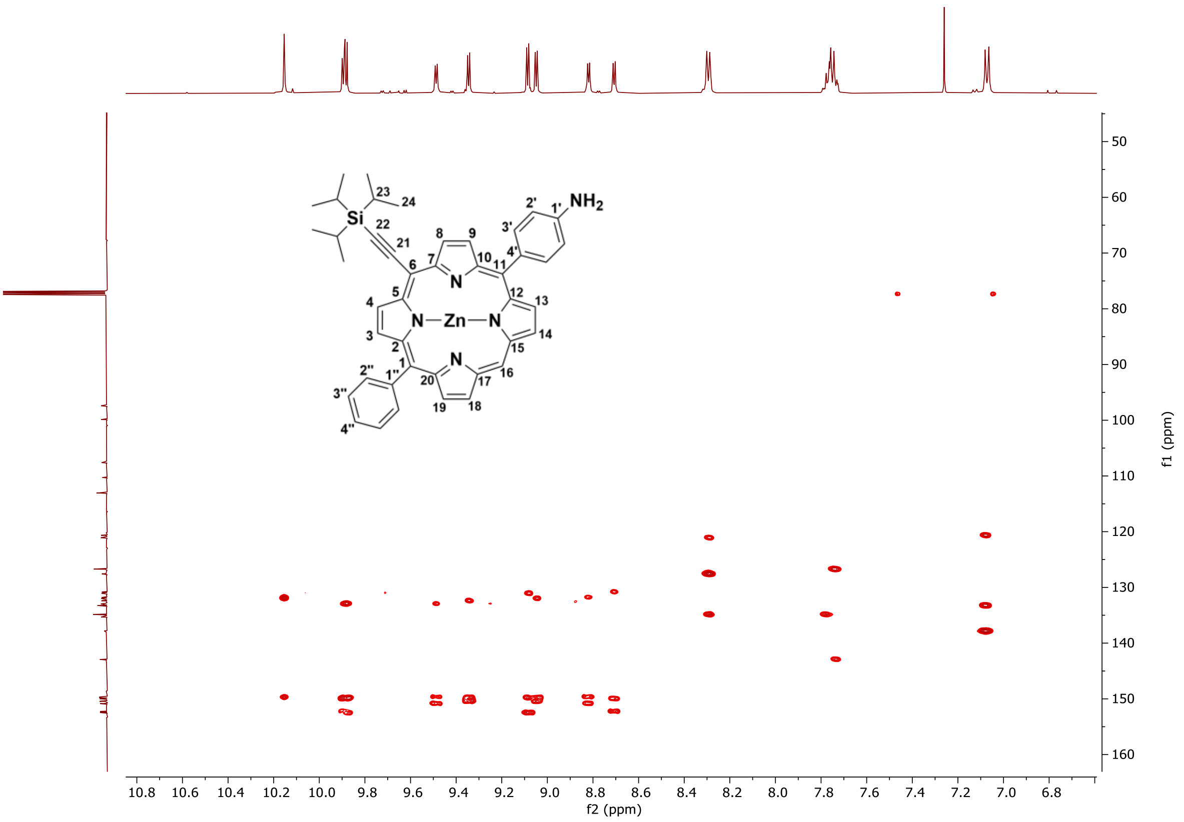

Figure S47: HMBC NMR spectrum of **5-Zn** in **CDCl$_3$** (500 MHz), optimized for J = 10 Hz.

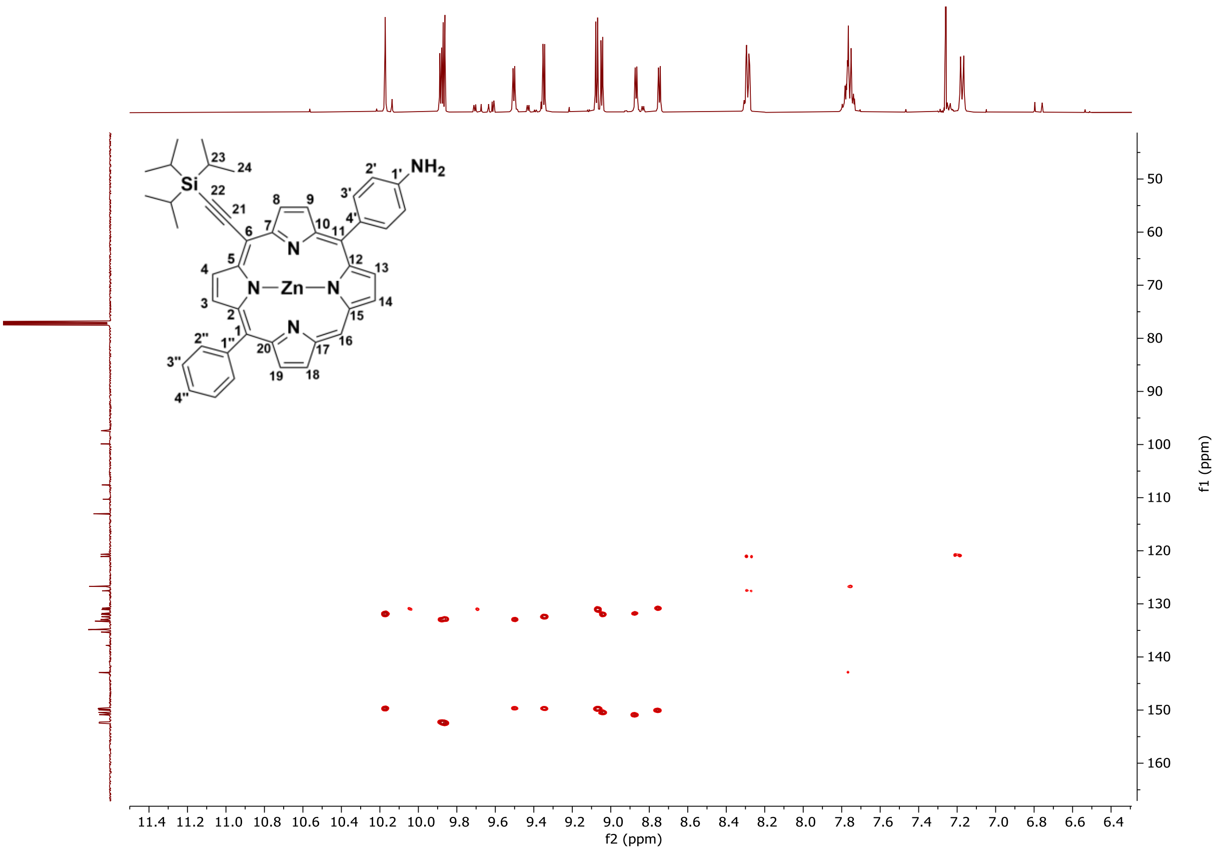

Figure S48: HMBC NMR spectrum of **5-Zn** in **CDCl$_3$** (500 MHz), optimized for J = 5 Hz

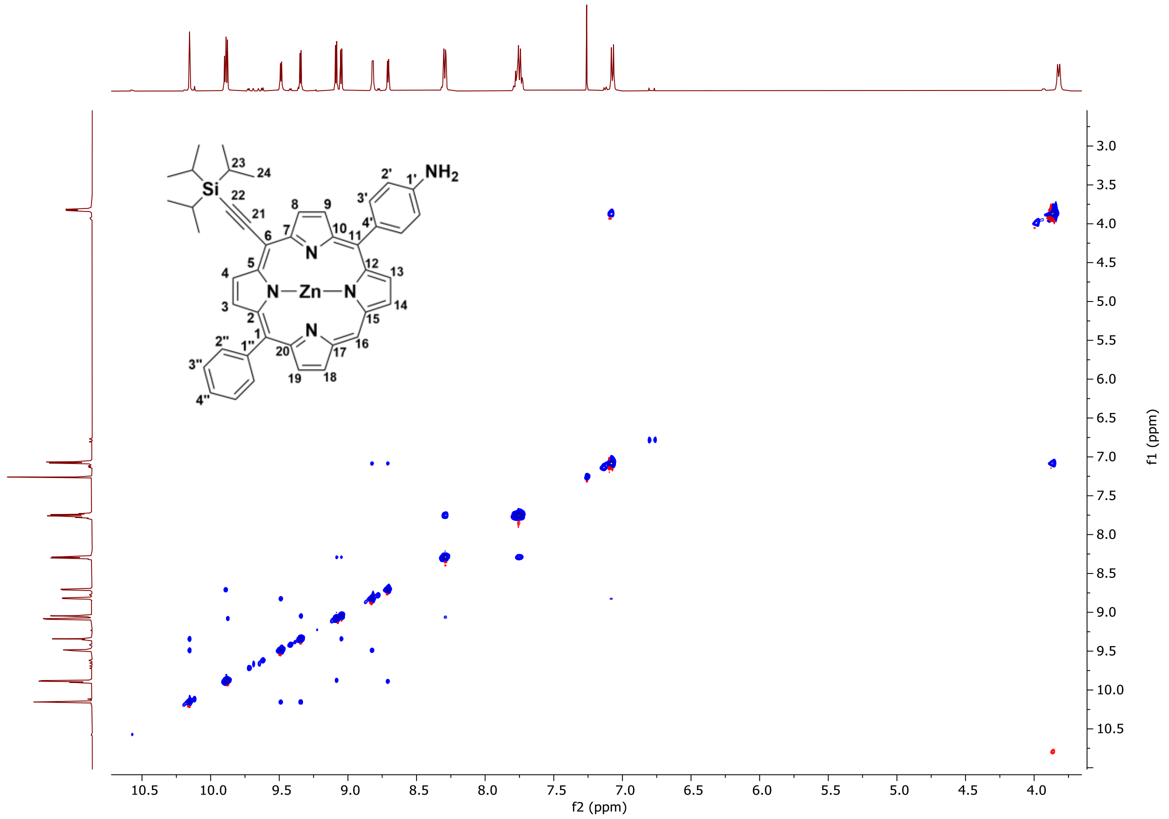

Figure S49: NOESY NMR spectrum of **5-Zn** in **$CDCl_3$** (500 MHz)

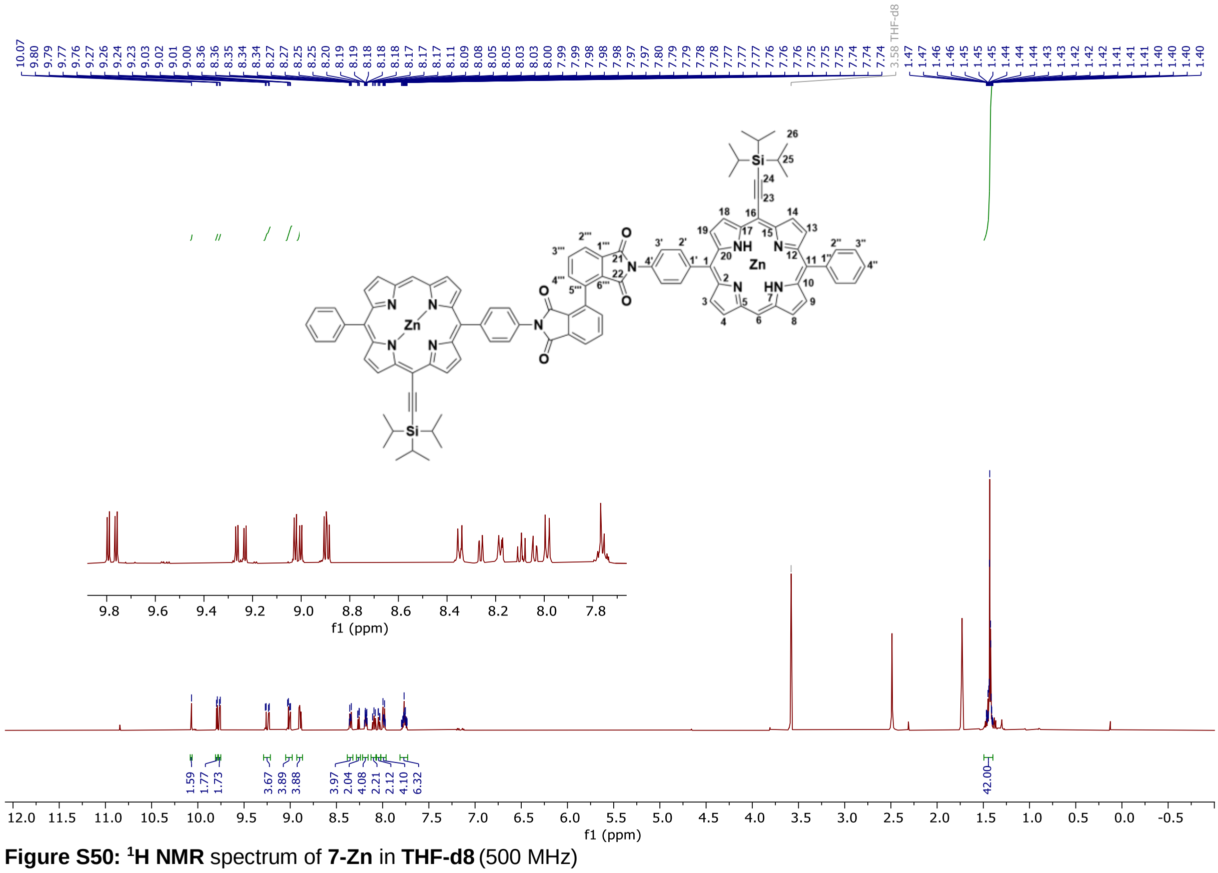

Figure S50: ^{1}H NMR spectrum of **7-Zn** in **THF-d8** (500 MHz)

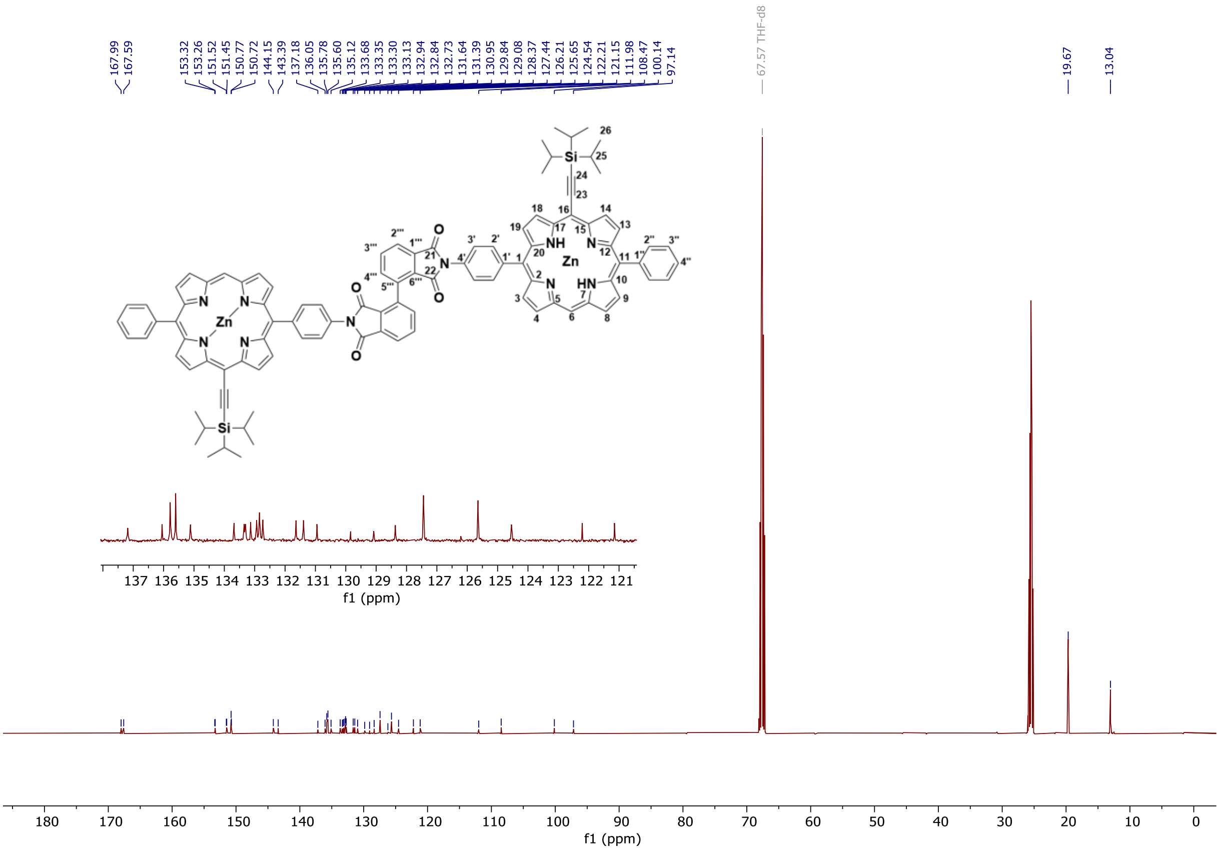

Figure S51: ^{13}C NMR spectrum of **7-Zn** in **THF-d8** (126 MHz)

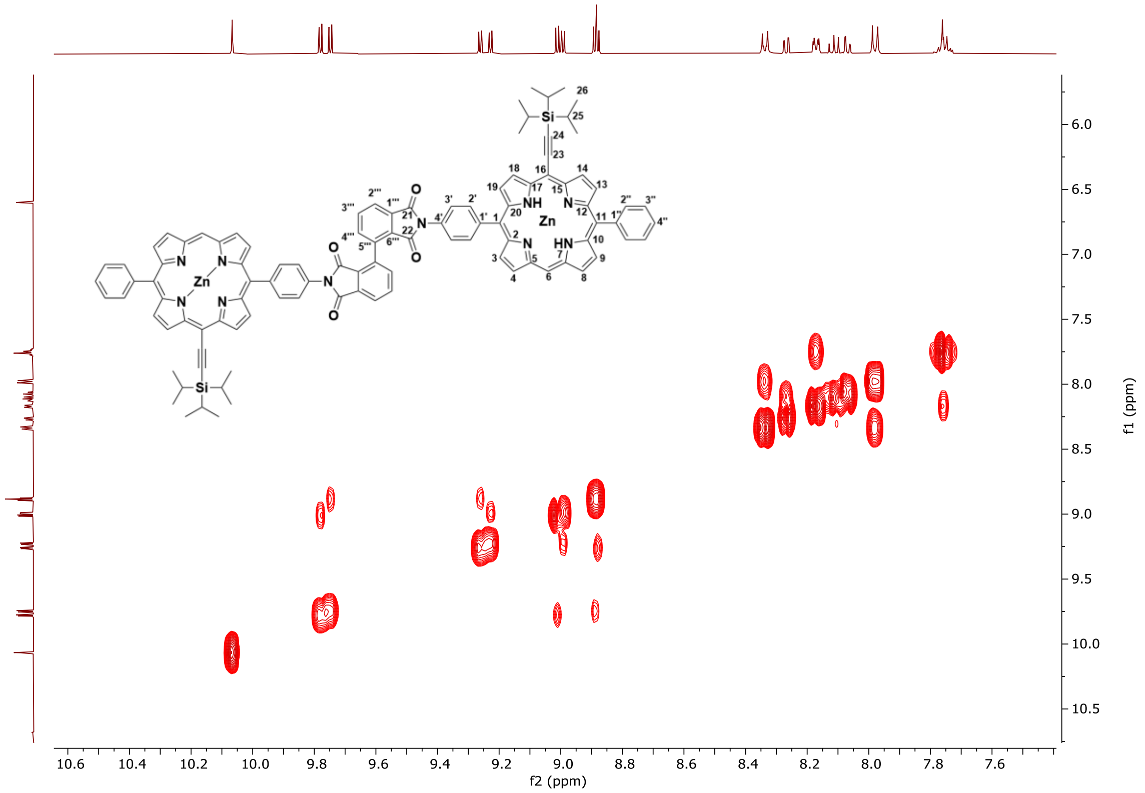

Figure S52: COSY NMR spectrum of **7-Zn** in **THF-d8** (126 MHz)

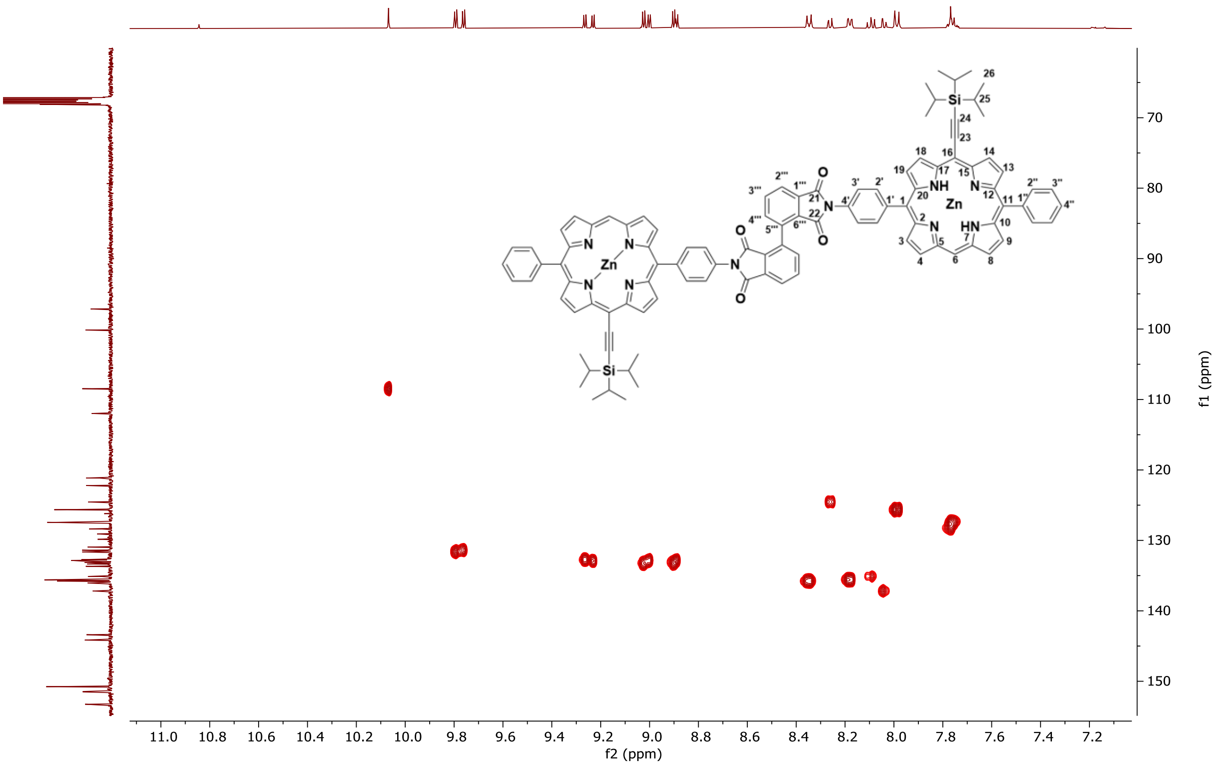

Figure S53: HMQC NMR spectrum of **7-Zn** in **THF-d8** (500 MHz)

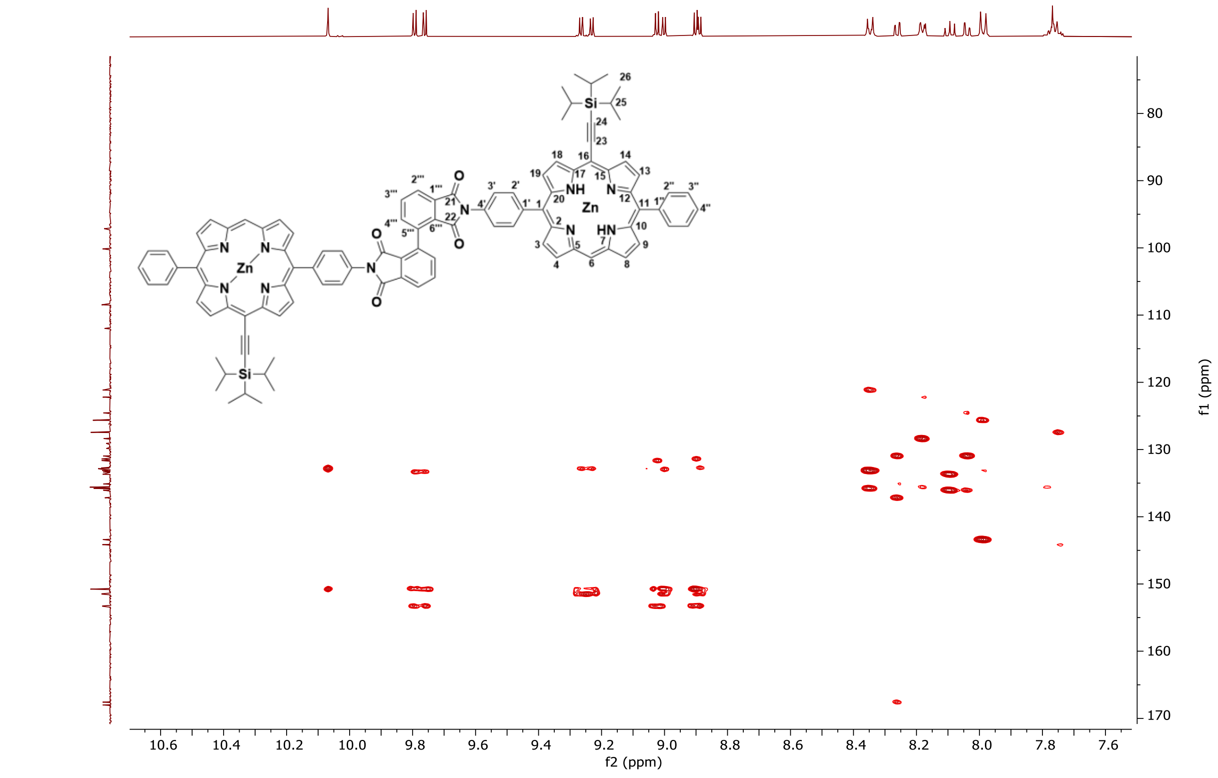

Figure S54: HMBC NMR spectrum of **7-Zn** in **THF-d8** (500 MHz), optimized for J = 10 Hz

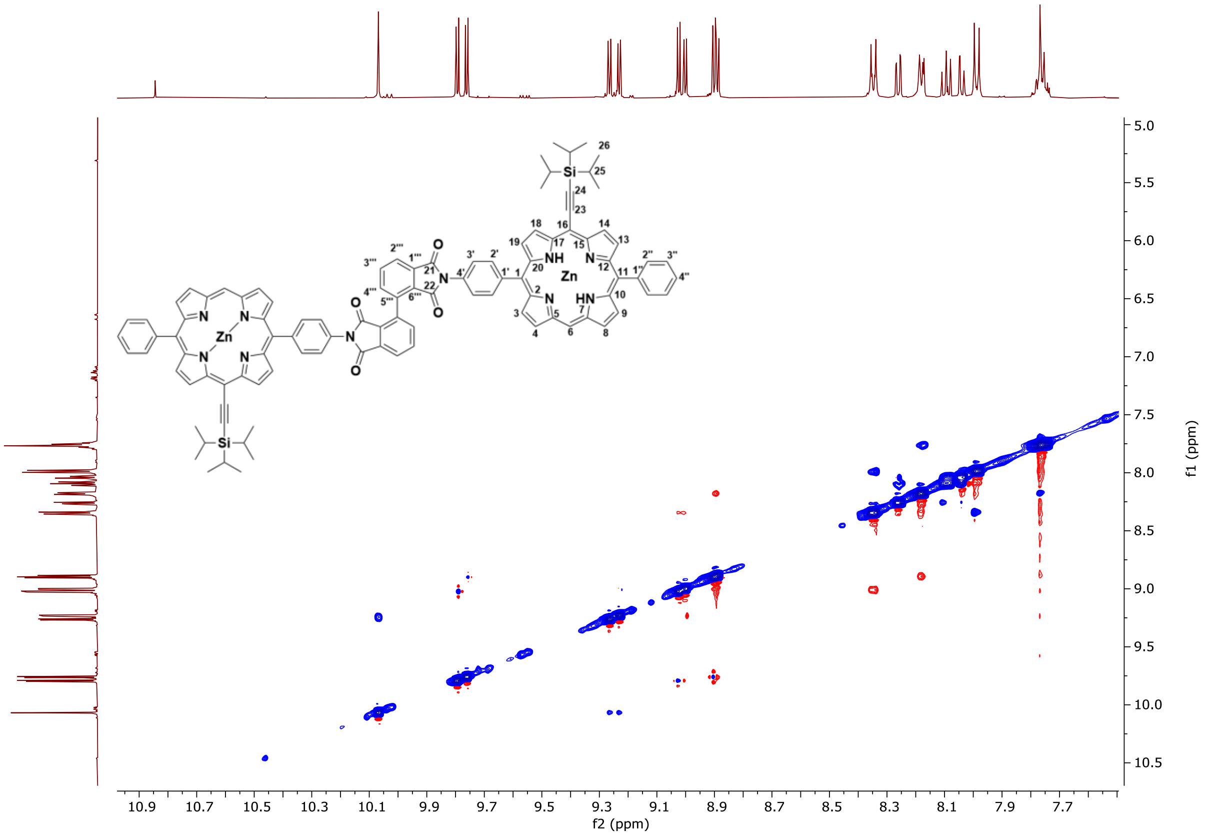

Figure S55: NOESY NMR spectrum of **7-Zn** in **THF-d8** (500 MHz)

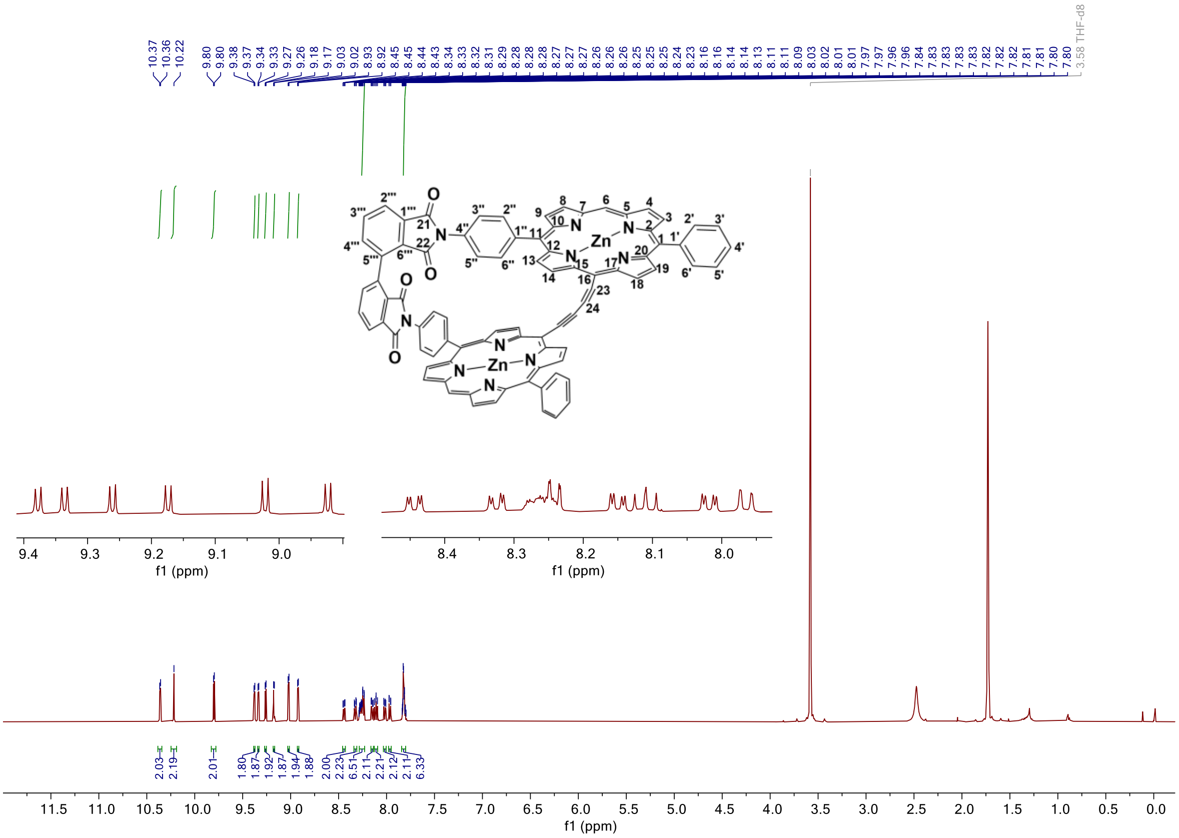

Figure S56: ^{1}H NMR spectrum of ***(rac)*-PoGe-[Zn, Zn]**in **THF-d8** (500 MHz)

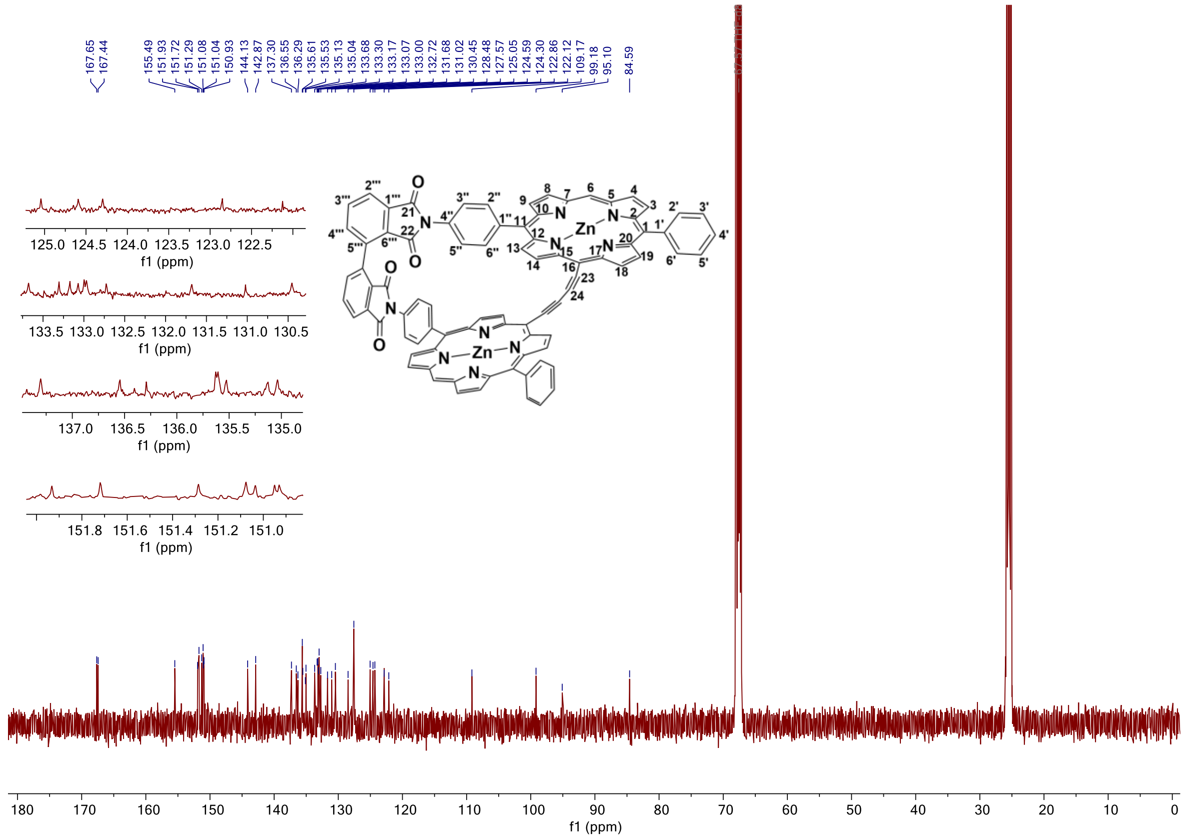

Figure S57: ^{13}C NMR spectrum of **(rac)-PoGe-[Zn, Zn]**in **THF-d8** (126 MHz)

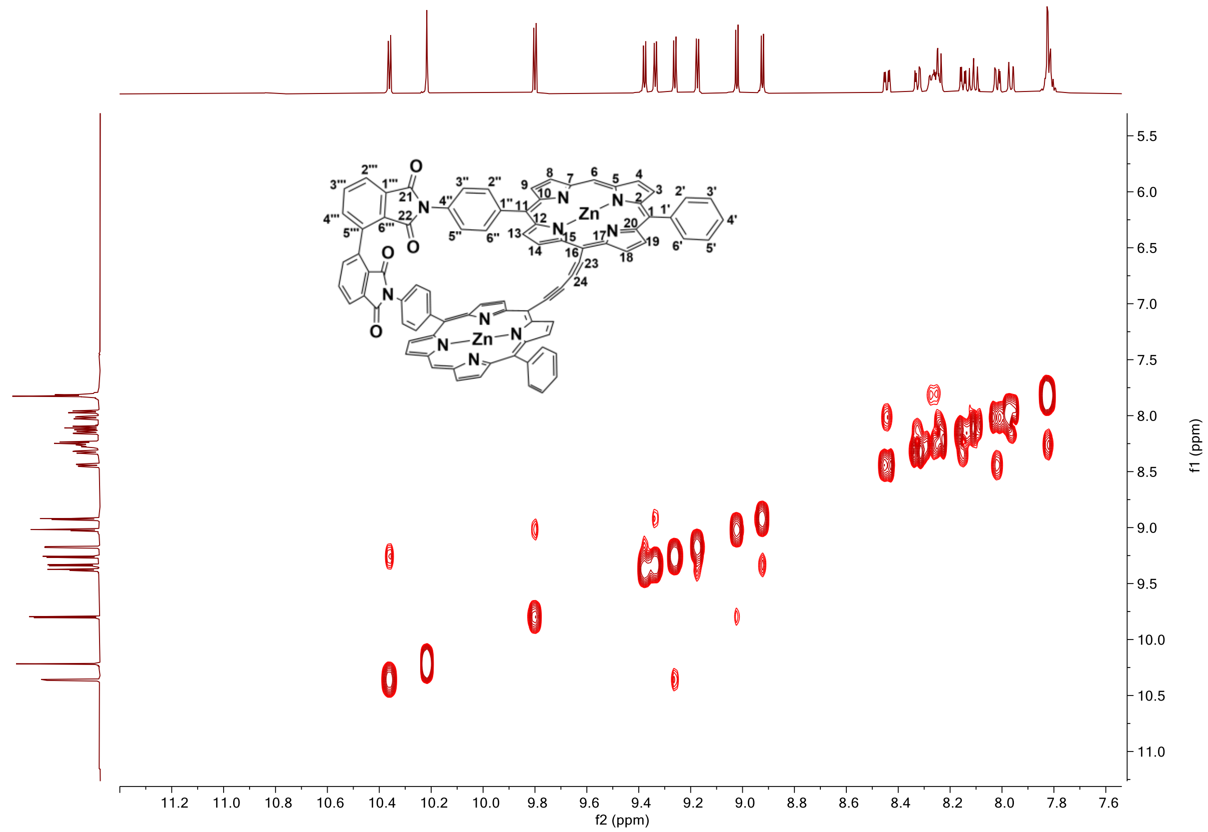

Figure S58: COSY NMR spectrum of **(rac)-PoGe-[Zn, Zn]**in **THF-d8** (500 MHz)

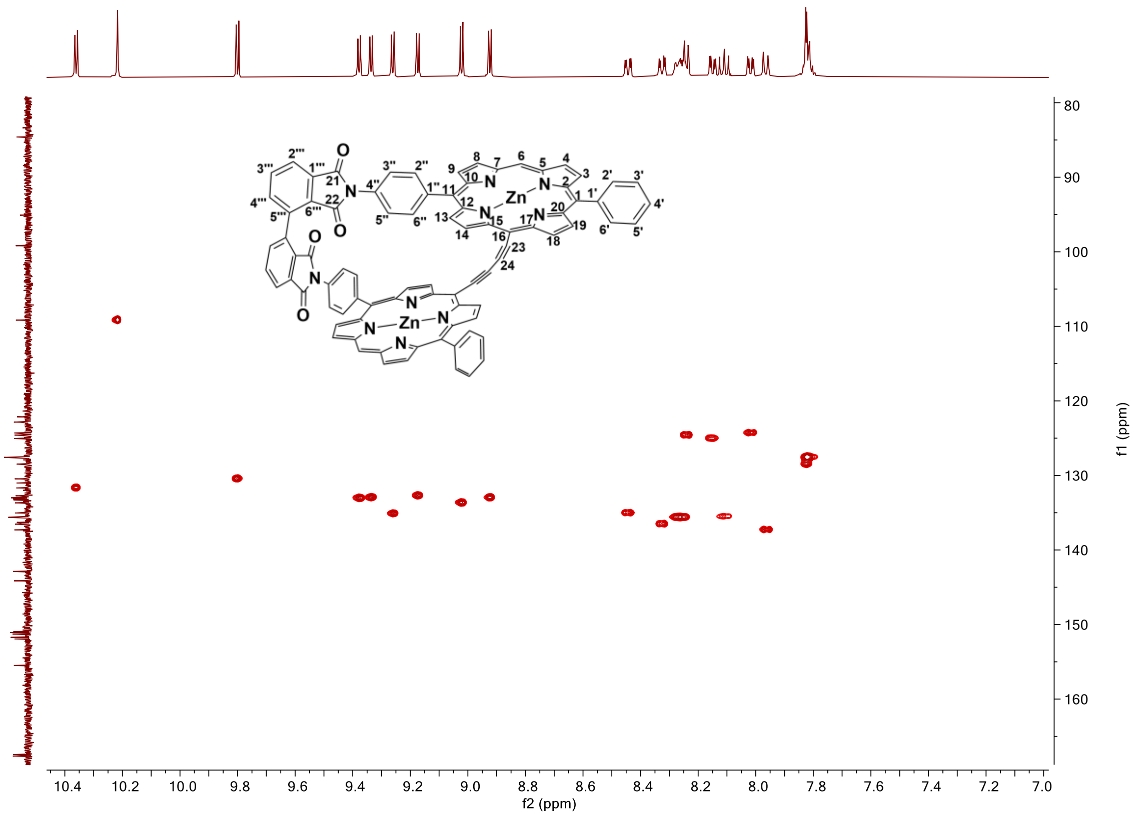

Figure S59: HMQC NMR spectrum of **(rac)-PoGe-[Zn, Zn]** in **THF-d8** (500 MHz)

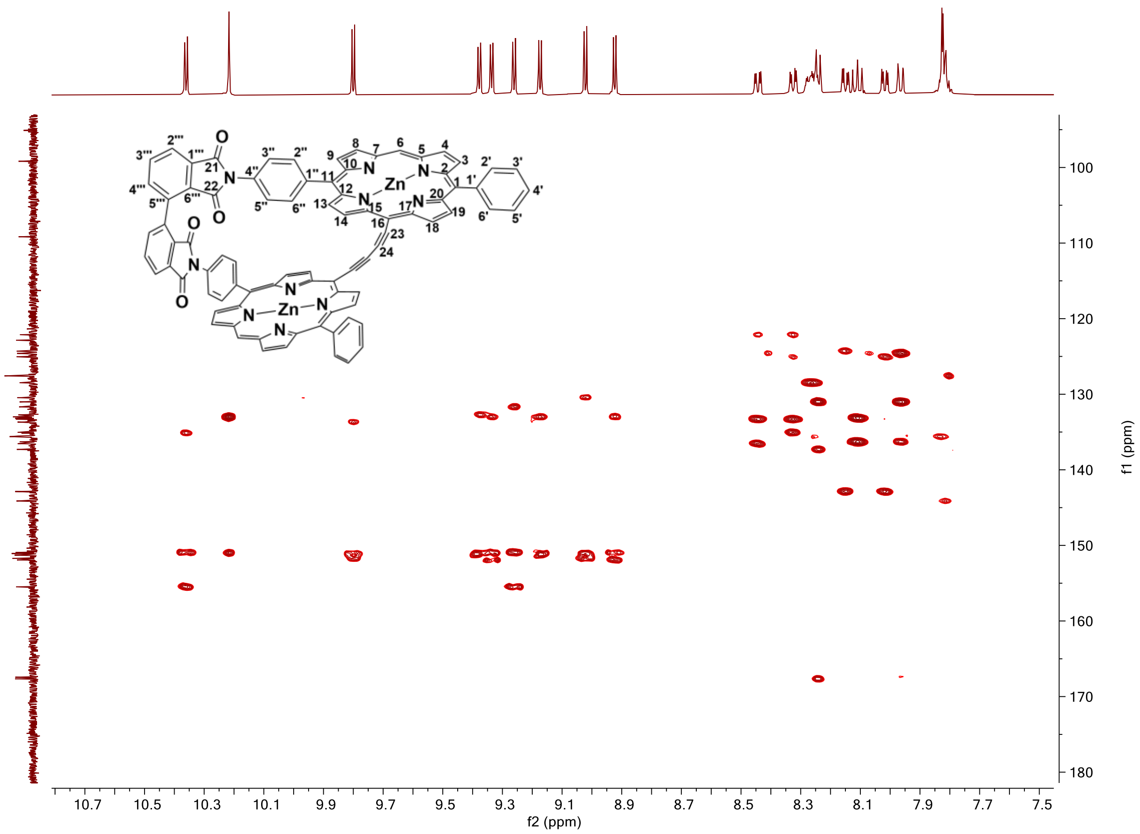

Figure S60: HMBC NMR spectrum of **(rac)-PoGe-[Zn, Zn]** in **THF-d8** (500 MHz), optimized for J = 10 Hz

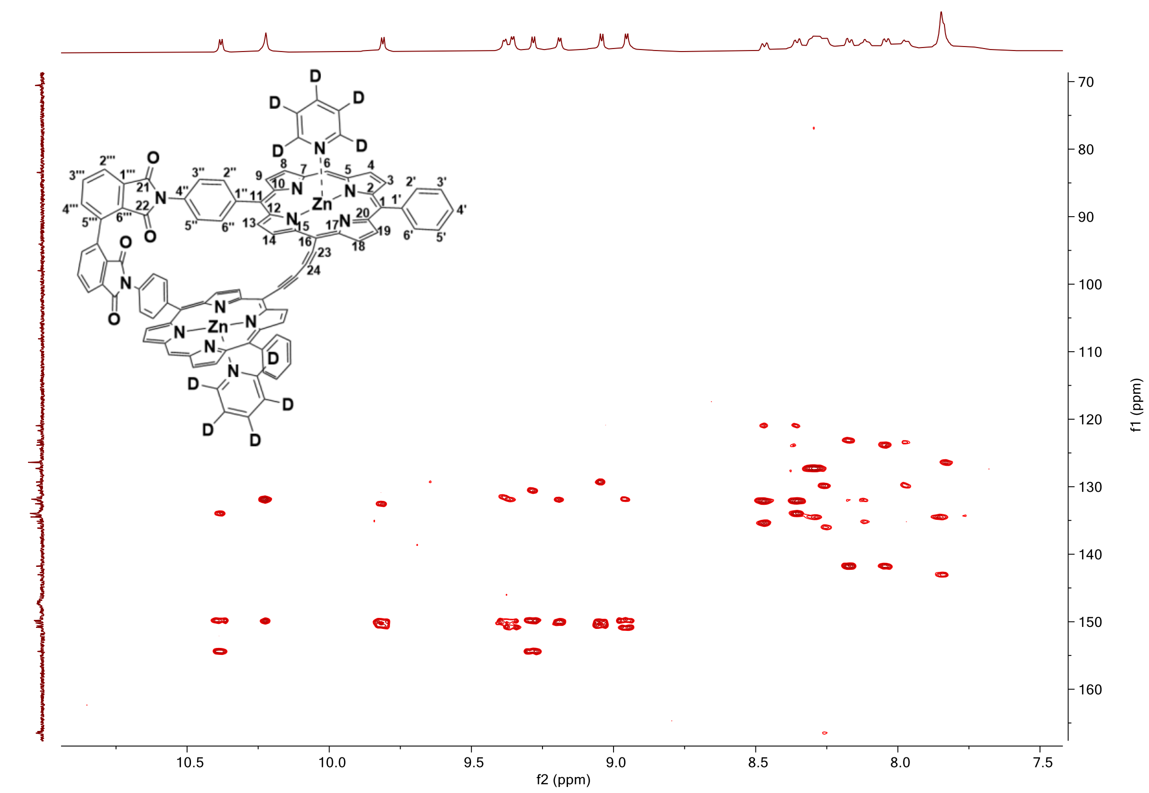

Figure S61: HMBC NMR spectrum of **(rac)-PoGe-[Zn, Zn]-pyrd5** in **THF-d8** (500 MHz), optimized for J = 10 Hz

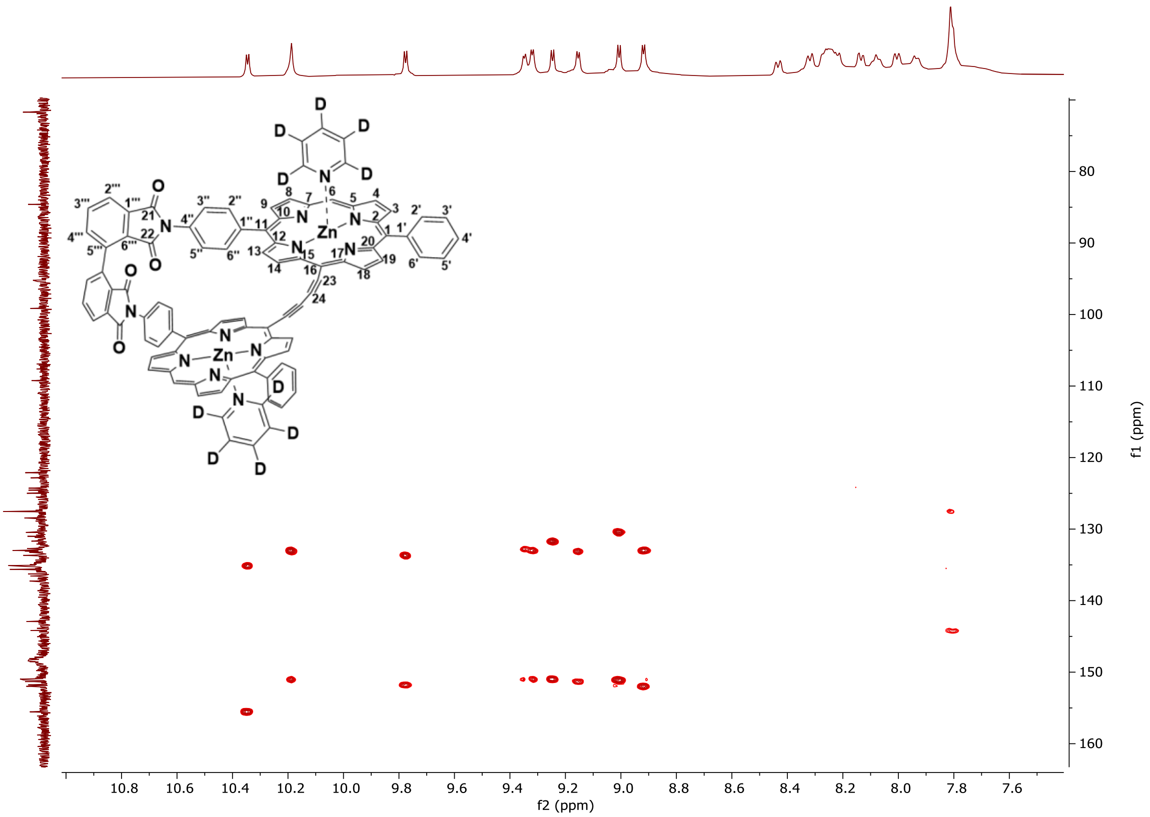

Figure S62: HMBC NMR spectrum of **(rac)-PoGe-[Zn, Zn]-pyrd5** in **THF-d8** (500 MHz), optimized for J = 5 Hz

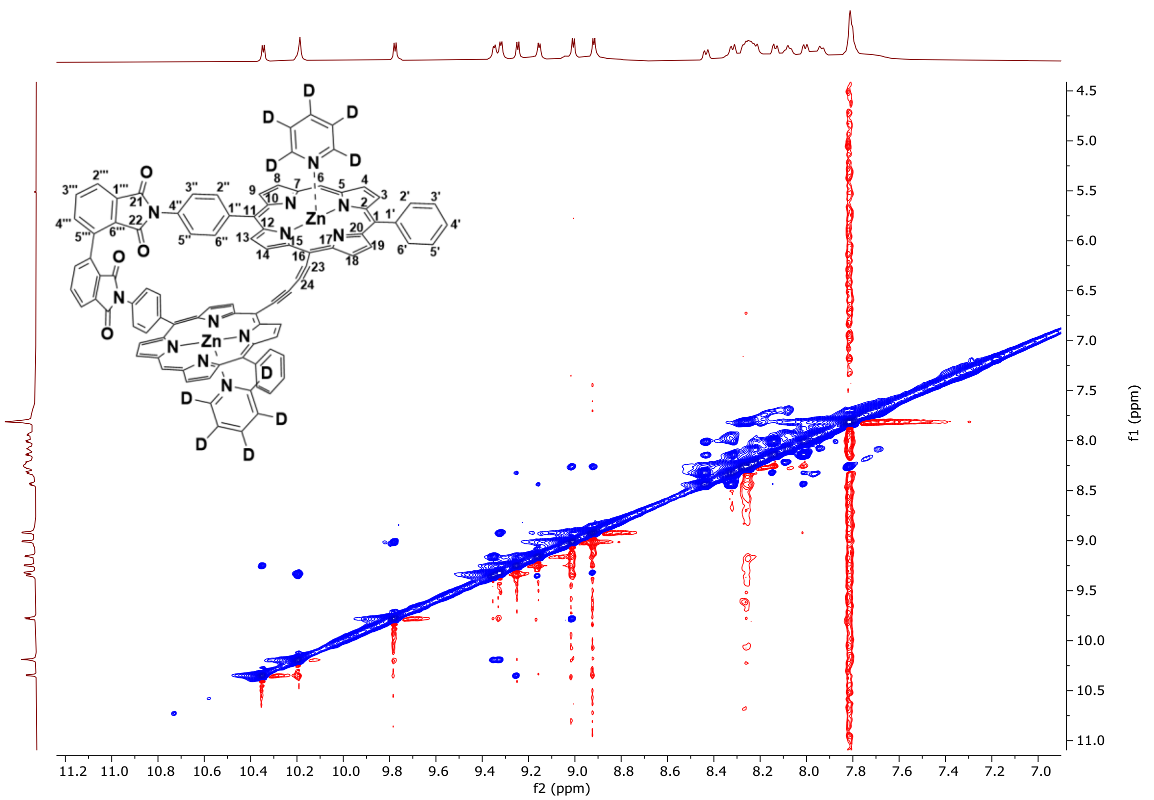

Figure S63: NOESY NMR spectrum of **(rac)-PoGe-[Zn, Zn]-pyrd5** in **THF-d8** (500 MHz)

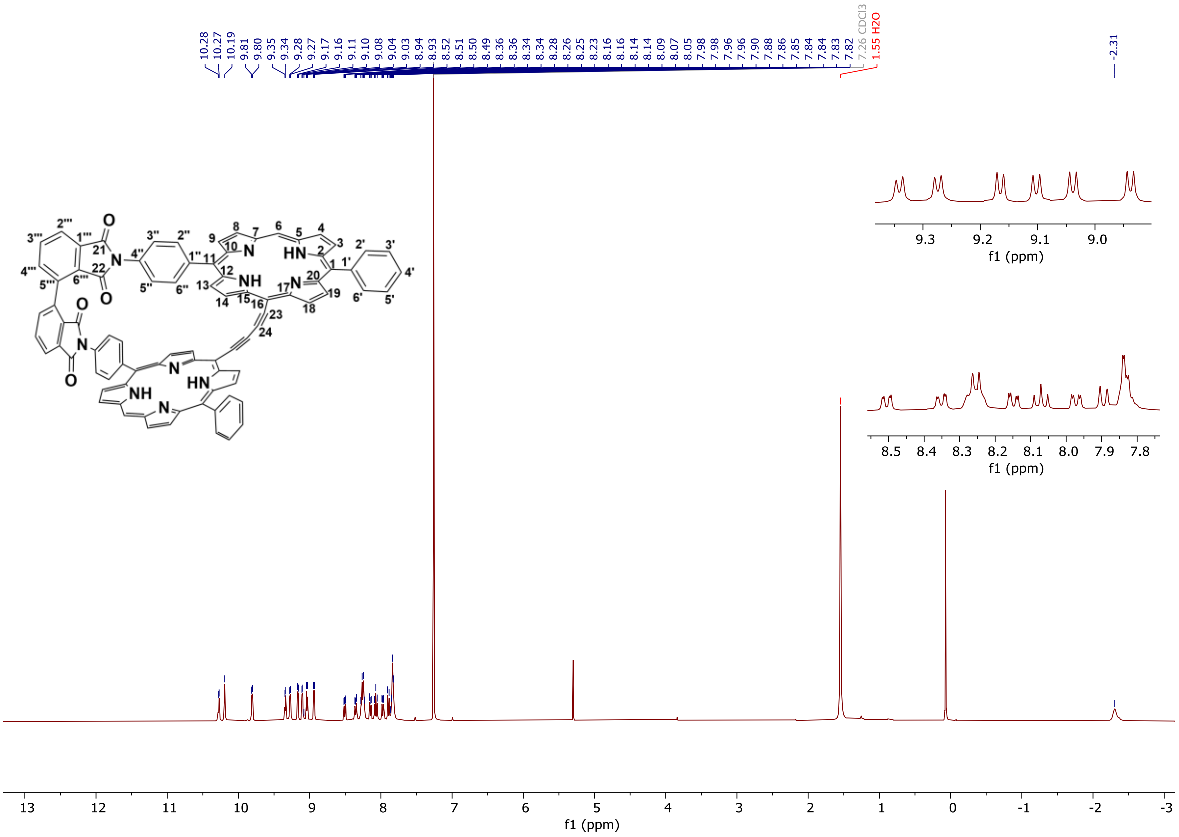

Figure S64: ^{1}H NMR spectrum of **(rac)-PoGe-[2H, 2H]**in **$CDCl_3$** (400 MHz)

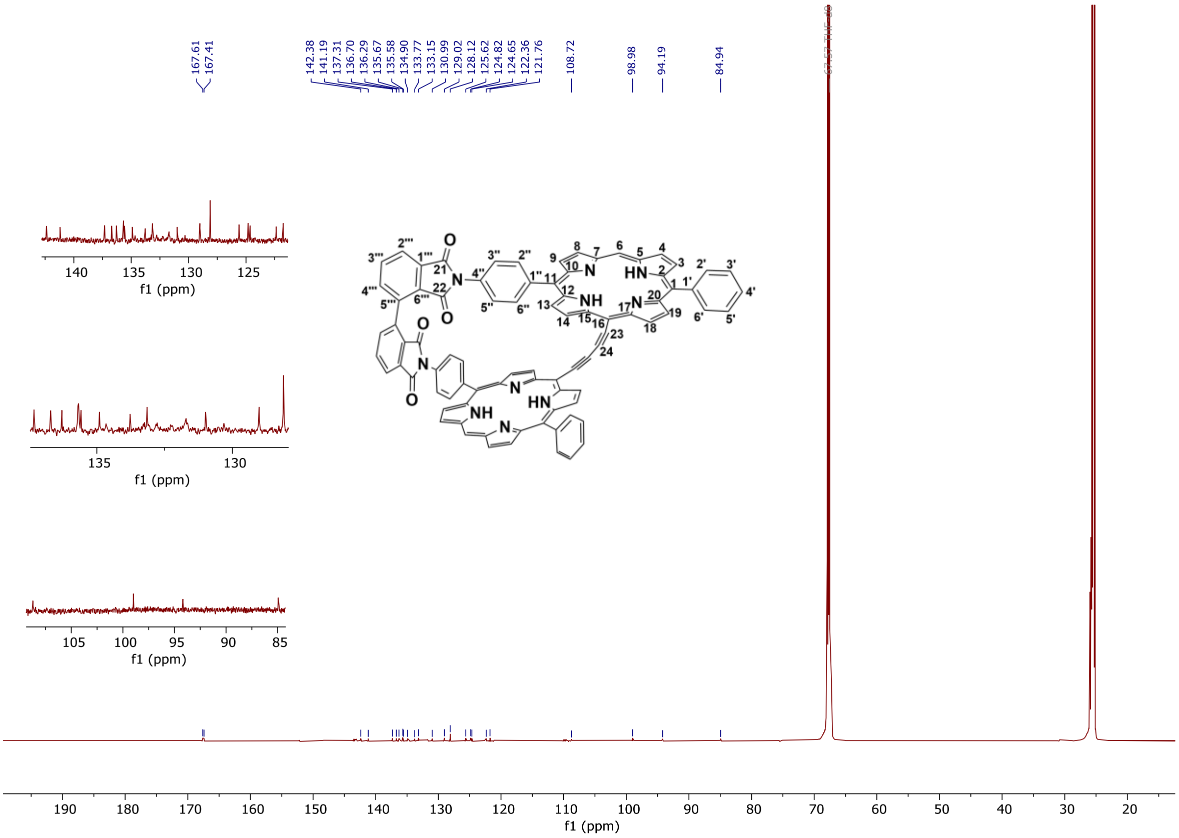

Figure S65: **^{13}C NMR** spectrum of **(rac)-PoGe-[2H, 2H]**in **THF-d8** (155 MHz). β-pyrrole carbons are broadened but can still be observed between 130 – 133 ppm, α-pyrrole carbons cannot be observed at all.

HR-MS Spectra

High Resolution Mass Spectrometry Report

Sample Name **JK-367**
Comment

Instrument maXis 4G
Method ms_nocolumn_mid_pos.m

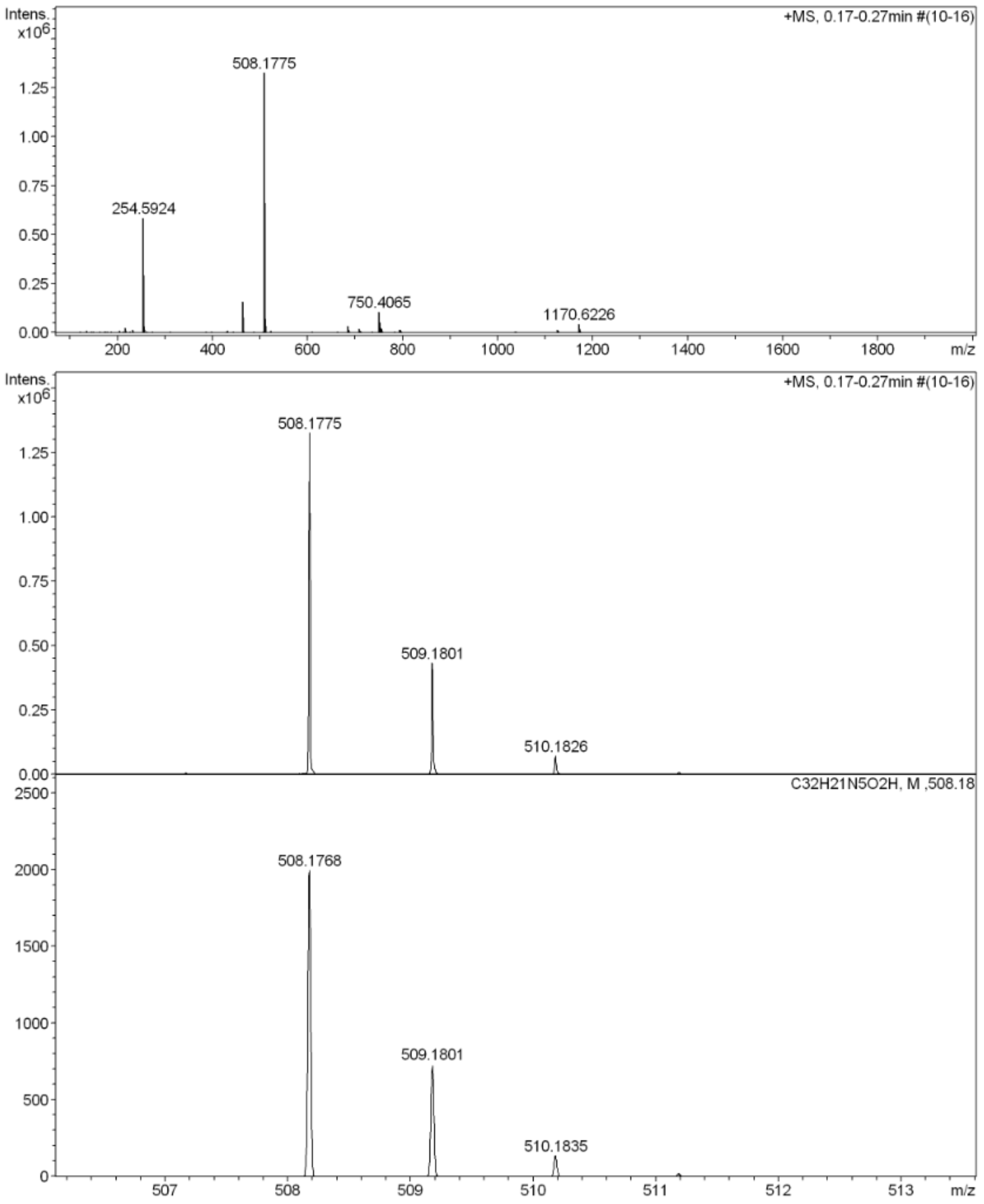

Bruker Compass DataAnalysis 4.0 Acquisition Date 02.11.2022 10:19:29 Page 1 of 3

High Resolution Mass Spectrometry Report

Measured m/z vs. theoretical m/z

Meas. m/z	#	Formula	Score	m/z	err [mDa]	err [ppm]	mSigma	rdb	e⁻ Conf	z
508.1775	1	C 32 H 22 N 5 O 2	100.00	508.1768	-0.7	-1.4	20.2	24.5	even	1+

Mass list

#	m/z	I %	I
1	122.0964	0.6	7992
2	136.1121	0.9	12530
3	144.9824	0.4	4739
4	147.0918	0.3	3580
5	147.9311	0.3	3379
6	149.9311	0.3	3636
7	150.1278	0.7	8677
8	161.1071	0.2	3240
9	164.1434	0.5	6431
10	173.0785	0.4	5233
11	175.1230	0.4	4776
12	178.1589	0.5	6968
13	186.0086	0.5	7107
14	188.0067	0.2	3310
15	198.1277	0.3	3803
16	205.0598	0.9	12031
17	212.1431	0.6	7535
18	215.1252	0.3	4443
19	216.5762	0.2	3293
20	217.1045	1.8	23970
21	226.1586	0.3	4446
22	226.9513	0.5	6399
23	227.0350	0.5	6880
24	229.0330	0.2	3087
25	229.1405	0.2	3062
26	232.0992	1.3	16592
27	232.6007	0.5	6617
28	242.2840	0.3	4105
29	254.5924	44.3	588065
30	255.0937	14.9	197427
31	255.5948	2.7	36256
32	256.0962	0.4	4842
33	261.1303	0.5	6244
34	265.9619	0.3	4270
35	273.1669	0.6	7407
36	301.1401	0.2	3220
37	312.0922	0.5	6300
38	326.3774	0.3	4409
39	355.2810	0.3	4376
40	385.2918	0.3	4185
41	387.1594	0.4	5310
42	399.3073	0.4	5669
43	413.3225	0.3	3800
44	429.3178	0.4	5242
45	432.1448	0.8	10854
46	433.1474	0.3	3503
47	443.3333	0.5	6691
48	457.3489	0.3	3922
49	463.1915	12.0	158770
50	464.1943	4.1	54530
51	465.1972	0.7	9886
52	473.3437	0.3	4385
53	487.3599	0.4	5238
54	507.1680	0.5	6875
55	508.1775	100.0	1327329
56	509.1801	32.7	433406
57	510.1826	5.5	72731
58	511.1856	0.7	9426
59	523.3231	0.8	10524
60	524.3269	0.3	3423
61	531.3861	0.2	3158
62	559.1310	0.3	3456

High Resolution Mass Spectrometry Report

#	m/z	I %	I
63	610.1832	0.4	4744
64	633.1490	0.3	4238
65	663.4527	0.7	8966
66	664.4569	0.3	4386
67	680.4790	0.3	3546
68	685.4345	2.6	34685
69	686.4379	1.2	16250
70	687.4411	0.3	4336
71	707.1678	0.3	3761
72	709.3796	1.8	23560
73	710.3825	0.8	10765
74	711.3792	1.0	12765
75	712.3816	0.4	5656
76	736.5409	0.4	5352
77	750.4065	8.2	109474
78	751.4096	4.0	52613
79	752.4060	4.3	57243
80	753.3546	1.7	22956
81	753.4079	1.8	24408
82	754.3581	0.8	10824
83	754.4105	0.5	7004
84	755.3551	1.8	23420
85	756.3583	0.8	10634
86	784.5417	0.4	4696
87	785.5449	0.2	3075
88	794.3812	1.1	14927
89	795.3848	0.5	7161
90	796.3817	1.1	15192
91	797.3840	0.5	7112
92	1037.3259	0.6	7640
93	1038.3298	0.4	5307
94	1125.6370	1.1	14501
95	1126.6402	0.9	11601
96	1127.6431	0.4	4998
97	1170.6226	3.6	47151
98	1171.6257	2.8	37615
99	1172.6289	1.3	17504
100	1173.6308	0.4	5326

Acquisition Parameter

**General**	Fore Vacuum<br>Scan Begin	3.16e+000 mBar<br>75 m/z	High Vacuum<br>Scan End	1.27e-007 mBar<br>2000 m/z	Source Type<br>Ion Polarity	ESI<br>Positive
**Source**	Set Nebulizer<br>Set Dry Heater	2.0 Bar<br>200 °C	Set Capillary<br>Set End Plate Offset	4500 V<br>-500 V	Set Dry Gas	8.0 l/min
**Quadrupole**	Set Ion Energy (MS only)	4.0 eV				
**Coll. Cell**	Collision Energy	8.0 eV	Set Collision Cell RF	600.0 Vpp	100.0 Vpp	
**Ion Cooler**	Set Ion Cooler Transfer Time	75.0 µs	Set Ion Cooler Pre Pulse Storage Time	10.0 µs		

Figure S66: HR-MS ESI spectrum of **2**

High Resolution Mass Spectrometry Report

Sample Name **JK-580**
Comment

Instrument maXis 4G
Method ms_nocolumn_mid_pos.m

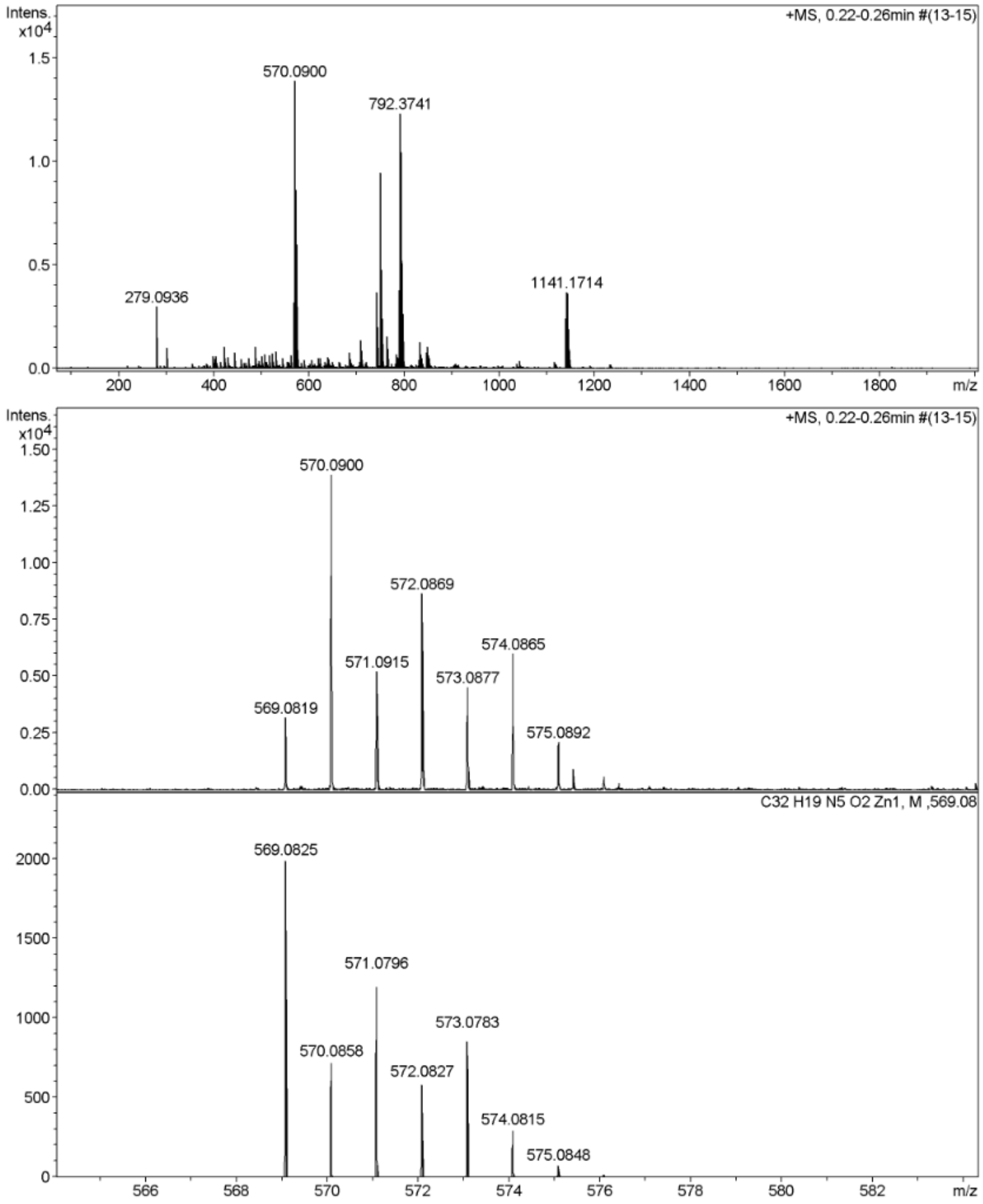

High Resolution Mass Spectrometry Report

Measured m/z vs. theoretical m/z

Meas. m/z	#	Formula	Score	m/z	err [mDa]	err [ppm]	mSigma	rdb	e⁻ Conf	z
570.0900	1	C 32 H 20 N 5 O 2 Zn	100.00	570.0903	0.3	0.6	20.3	25.5	even	1+
1139.1731	1	C 64 H 39 N 10 O 4 Zn 2	100.00	1139.1733	0.2	0.2	90.0	50.5	even	

Mass list

#	m/z	I %	I
1	279.0936	21.5	2987
2	301.0752	7.3	1014
3	422.3779	7.6	1058
4	487.3614	7.7	1065
5	569.0819	22.9	3187
6	570.0900	100.0	13898
7	571.0915	37.4	5203
8	572.0869	62.2	8649
9	573.0877	32.6	4527
10	574.0865	43.2	6000
11	575.0892	15.1	2102
12	709.3807	9.8	1369
13	742.4947	26.5	3677
14	743.4993	14.4	1997
15	750.4063	68.1	9463
16	751.4096	34.2	4759
17	752.4065	33.7	4688
18	753.4077	17.4	2422
19	764.5724	11.2	1559
20	790.3731	27.1	3770
21	791.3742	65.2	9059
22	792.3741	88.7	12334
23	793.3771	38.6	5360
24	794.3739	75.1	10437
25	795.3775	33.7	4680
26	796.3755	36.6	5083
27	797.3783	19.2	2664
28	833.3998	9.1	1271
29	848.1728	7.5	1047
30	1139.1731	17.5	2427
31	1140.1758	15.5	2153
32	1141.1714	26.5	3686
33	1142.1690	20.2	2804
34	1143.1692	26.2	3636
35	1144.1718	16.4	2280
36	1145.1702	13.7	1910
37	1146.1682	8.3	1159

Acquisition Parameter

**General**	Fore Vacuum	3.10e+000 mBar	High Vacuum	9.50e-008 mBar	Source Type	ESI
	Scan Begin	75 m/z	Scan End	2000 m/z	Ion Polarity	Positive
**Source**	Set Nebulizer	2.0 Bar	Set Capillary	3500 V	Set Dry Gas	8.0 l/min
	Set Dry Heater	200 °C	Set End Plate Offset	-500 V		
**Quadrupole**	Set Ion Energy (MS only)	4.0 eV				
**Coll. Cell**	Collision Energy	8.0 eV	Set Collision Cell RF	500.0 Vpp	100.0 Vpp	
**Ion Cooler**	Set Ion Cooler Transfer Time	75.0 µs	Set Ion Cooler Pre Pulse Storage Time	10.0 µs		

Bruker Compass DataAnalysis 4.0 Acquisition Date 08.12.2023 09:24:03 Page 2 of 2

Figure S67: HR-MS ESI spectrum of **2-Zn**

High Resolution Mass Spectrometry Report

Sample Name **JK-381-G**
Comment

Instrument maXis 4G
Method ms_nocolumn_mid_pos.m

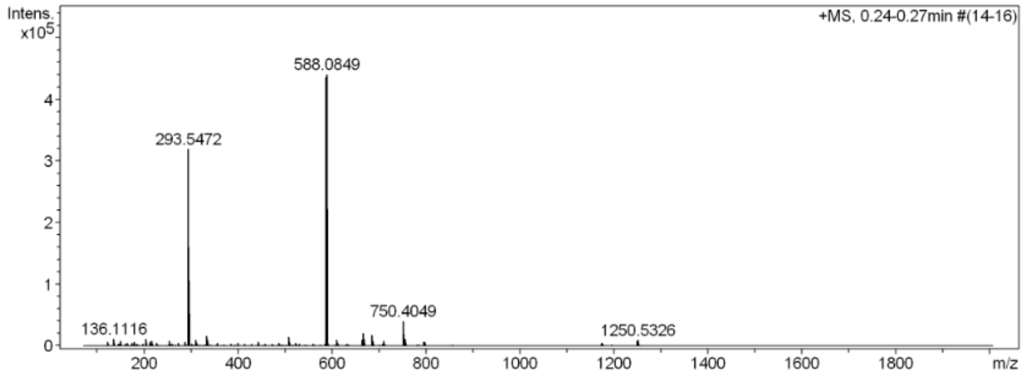

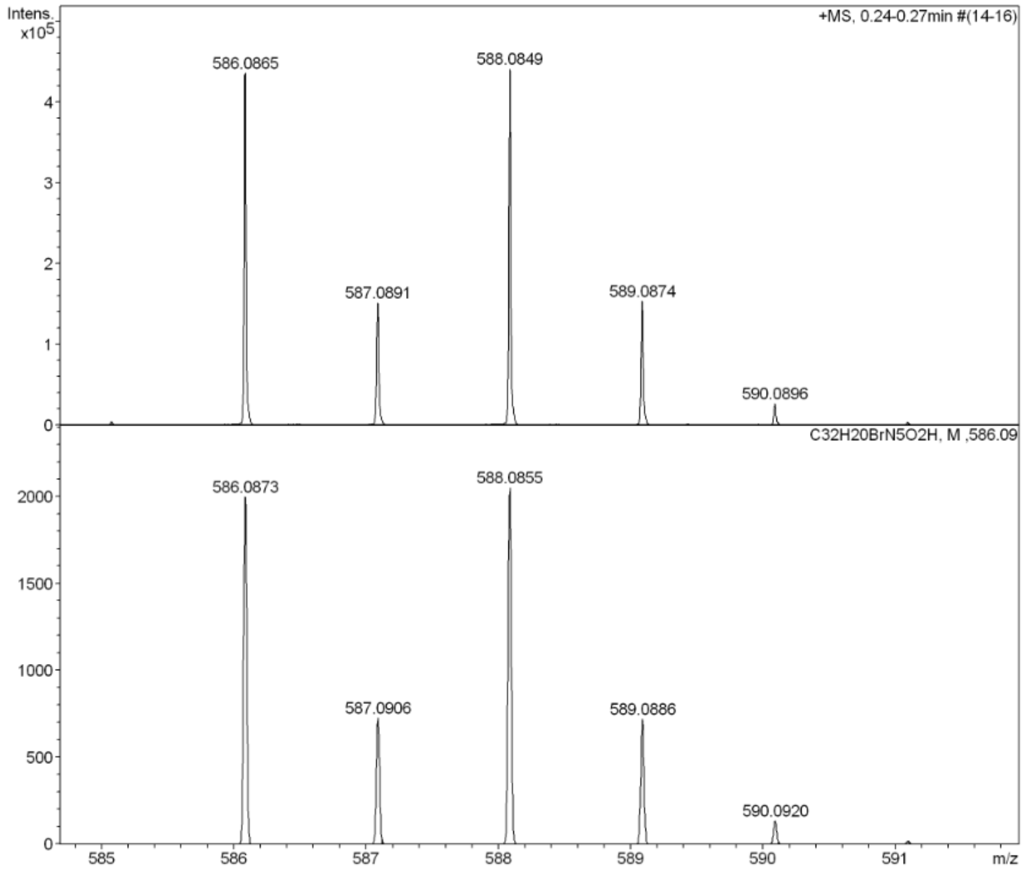

High Resolution Mass Spectrometry Report

Measured m/z vs. theoretical m/z

Meas. m/z	#	Formula	Score	m/z	err [mDa]	err [ppm]	mSigma	rdb	e⁻ Conf	z
586.0865	1	C 32 H 21 Br N 5 O 2	100.00	586.0873	0.8	1.4	11.8	24.5	even	1+
1171.1668	1	C 64 H 41 Br 2 N 10 O 4	100.00	1171.1674	0.6	0.5	46.5	48.5	even	

Mass list

#	m/z	I %	I
1	122.0960	1.6	7269
2	136.1116	2.8	12289
3	144.9821	0.9	3800
4	147.0912	0.8	3352
5	147.9309	0.7	3145
6	150.1277	2.0	8685
7	161.1070	0.8	3515
8	164.1430	1.3	5520
9	175.1227	1.1	4803
10	178.1586	1.5	6456
11	186.0084	1.0	4521
12	198.1270	0.8	3543
13	205.0595	2.5	11205
14	212.1430	1.5	6821
15	215.1250	0.7	3196
16	217.1041	1.9	8583
17	226.1583	1.0	4256
18	226.9513	1.0	4287
19	227.0348	1.1	5065
20	254.5916	2.0	8812
21	255.0930	0.8	3470
22	261.1302	0.9	3801
23	273.1666	1.3	5588
24	288.2526	1.5	6505
25	293.5472	72.6	319967
26	294.0485	25.3	111743
27	294.5464	69.2	305315
28	295.0475	24.4	107824
29	295.5487	4.4	19208
30	296.0502	0.7	3026
31	301.1400	0.8	3394
32	310.2345	2.2	9914
33	312.0922	1.3	5927
34	332.5019	1.9	8187
35	333.0036	0.7	3029
36	333.5010	3.8	16745
37	334.0026	1.5	6572
38	334.5003	2.2	9585
39	335.0016	0.8	3628
40	355.2809	1.1	4895
41	385.2912	0.8	3684
42	399.3066	1.4	6217
43	413.3228	0.9	3840
44	429.3173	1.0	4202
45	443.3331	1.5	6458
46	457.3480	0.8	3686
47	473.3429	0.9	3798
48	487.3588	1.1	5013
49	508.1750	3.4	15036
50	509.1785	1.1	5009
51	512.0523	0.7	3120
52	523.3218	1.2	5218
53	531.3846	0.9	4110
54	559.1295	0.9	3821
55	585.0773	1.1	4711
56	586.0865	98.8	435875
57	587.0891	34.4	151740
58	588.0849	100.0	441011
59	589.0874	34.9	153841
60	590.0896	6.2	27235
61	591.0917	0.9	4085

High Resolution Mass Spectrometry Report

#	m/z	I %	I
62	610.1826	2.3	10029
63	611.1827	1.4	6200
64	612.1804	0.9	4118
65	633.1480	0.9	3985
66	663.4528	1.3	5544
67	663.9954	2.3	9996
68	664.9982	0.9	4185
69	665.9939	4.8	21383
70	666.9975	1.7	7526
71	667.9925	2.5	11158
72	668.9951	0.9	3874
73	684.2001	0.9	4137
74	685.4335	4.2	18472
75	686.4365	2.0	8950
76	707.1664	0.9	3944
77	709.3785	2.1	9072
78	710.3824	1.0	4214
79	711.3782	1.0	4367
80	750.4049	9.3	40983
81	751.4082	4.4	19315
82	752.4049	4.9	21625
83	753.3533	2.5	11133
84	753.4067	2.4	10561
85	754.3569	1.3	5514
86	754.4097	0.7	3233
87	755.3540	2.7	12009
88	756.3575	1.2	5324
89	794.3804	1.6	6860
90	795.3836	0.8	3513
91	796.3813	1.7	7543
92	797.3829	0.9	4163
93	1173.1638	1.3	5706
94	1174.1682	0.9	4052
95	1175.1650	0.9	3879
96	1248.5328	1.8	8021
97	1249.5367	1.5	6727
98	1250.5326	2.2	9855
99	1251.5352	1.5	6681
100	1252.5382	0.7	3002

Acquisition Parameter

**General**	Fore Vacuum<br>Scan Begin	3.21e+000 mBar<br>75 m/z	High Vacuum<br>Scan End	1.27e-007 mBar<br>2000 m/z	Source Type<br>Ion Polarity	ESI<br>Positive
**Source**	Set Nebulizer<br>Set Dry Heater	2.0 Bar<br>200 °C	Set Capillary<br>Set End Plate Offset	4500 V<br>-500 V	Set Dry Gas	8.0 l/min
**Quadrupole**	Set Ion Energy (MS only)	4.0 eV				
**Coll. Cell**	Collision Energy	8.0 eV	Set Collision Cell RF	600.0 Vpp	100.0 Vpp	
**Ion Cooler**	Set Ion Cooler Transfer Time	75.0 µs	Set Ion Cooler Pre Pulse Storage Time	10.0 µs		

Figure S68: HR-MS ESI spectrum of **3**

High Resolution Mass Spectrometry Report

Sample Name	**JK-381-dibrom**	Instrument	maXis 4G
Comment		Method	ms_nocolumn_mid_pos.m

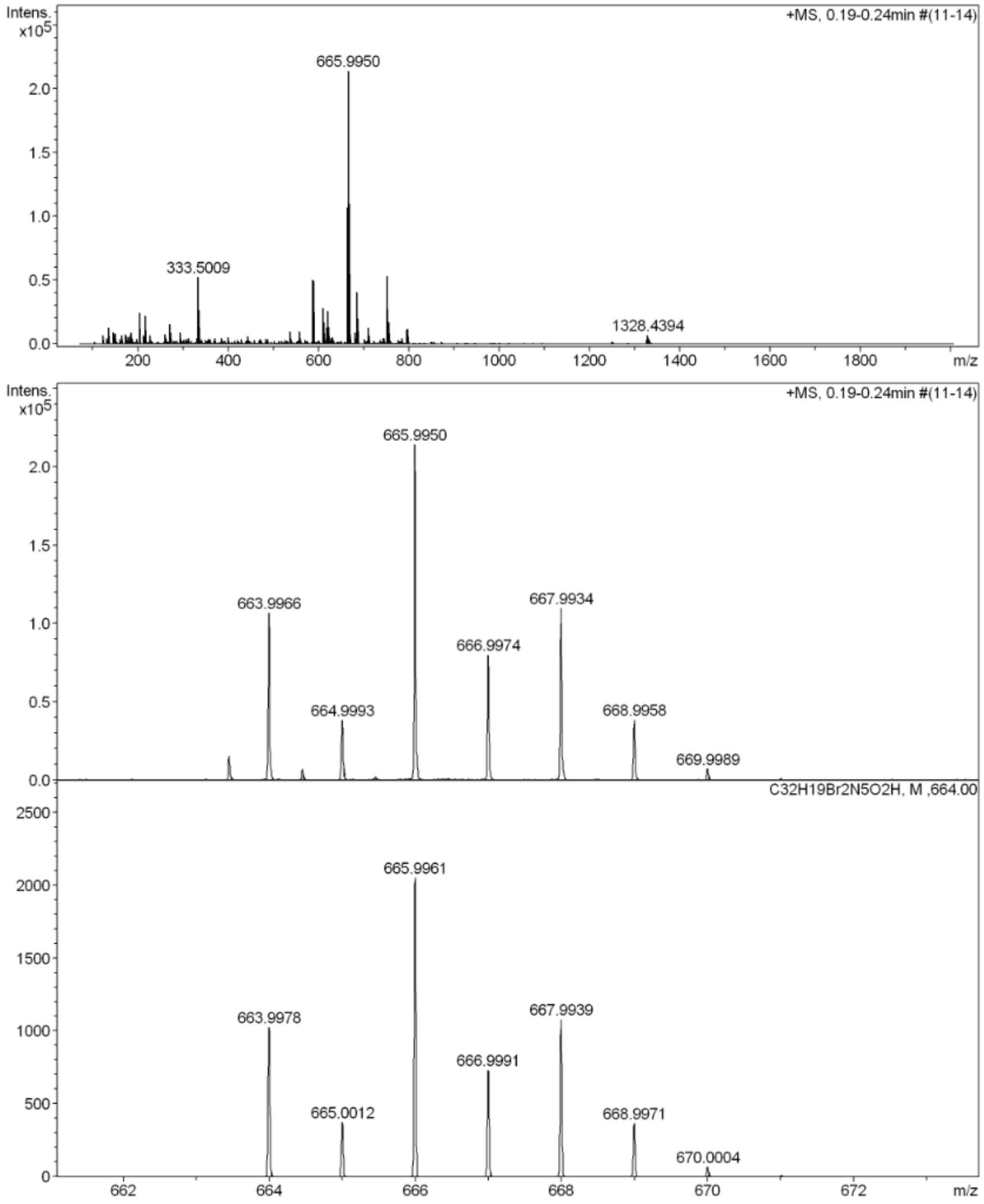

High Resolution Mass Spectrometry Report

Measured m/z vs. theoretical m/z

Meas. m/z	#	Formula	Score	m/z	err [mDa]	err [ppm]	mSigma	rdb	e⁻ Conf	z
663.9966	1	C 32 H 20 Br 2 N 5 O 2	100.00	663.9978	1.2	1.8	11.3	24.5	even	1+

Mass list

#	m/z	I %	I
1	122.0963	3.2	6941
2	131.9621	2.0	4376
3	136.1120	6.0	12885
4	144.9821	4.3	9272
5	146.9801	2.4	5131
6	147.0916	2.4	5075
7	147.9310	3.7	7898
8	149.9307	3.7	7974
9	150.1276	3.9	8308
10	164.1431	3.1	6681
11	173.0781	3.4	7384
12	175.1225	2.8	5966
13	178.1587	2.6	5550
14	183.0775	3.2	6913
15	186.0084	4.2	8912
16	188.0068	2.1	4492
17	205.0594	11.3	24238
18	212.1428	3.1	6554
19	215.1245	2.5	5307
20	217.1041	10.4	22201
21	226.1583	2.0	4355
22	227.0346	3.2	6760
23	261.1299	3.5	7418
24	262.1793	2.1	4541
25	270.2420	7.3	15553
26	273.1662	4.1	8834
27	293.5463	4.2	8936
28	294.5453	3.7	7855
29	312.0918	2.1	4494
30	331.2078	2.2	4705
31	332.5016	13.2	28331
32	333.0029	4.5	9629
33	333.5009	24.5	52460
34	334.0020	8.8	18923
35	334.4999	12.5	26848
36	335.0011	4.5	9728
37	336.2130	2.2	4640
38	355.2806	2.0	4256
39	371.1000	2.2	4693
40	385.2912	2.1	4410
41	399.3066	2.4	5222
42	429.3175	2.0	4253
43	443.3327	2.7	5779
44	536.1635	4.7	9998
45	537.1640	2.4	5248
46	557.0933	4.6	9823
47	558.0941	2.6	5492
48	586.0857	23.4	50197
49	587.0888	8.8	18871
50	588.0840	23.1	49591
51	589.0869	8.4	18034
52	610.1823	13.0	27883
53	611.1830	8.0	17123
54	612.1812	6.0	12916
55	613.1807	2.2	4695
56	619.0108	6.0	12758
57	620.0143	2.6	5499
58	621.0092	12.2	26172
59	622.0126	4.4	9474
60	623.0078	6.4	13749
61	624.0101	2.1	4484
62	628.1931	2.1	4541

High Resolution Mass Spectrometry Report

#	m/z	I %	I
63	631.1115	2.6	5655
64	663.4520	7.3	15673
65	663.9966	49.8	106745
66	664.4553	3.4	7187
67	664.9993	18.0	38540
68	665.9950	100.0	214237
69	666.9974	37.4	80138
70	667.9934	51.2	109720
71	668.9958	18.1	38729
72	669.9989	3.4	7273
73	680.4782	4.2	8935
74	681.4812	2.1	4444
75	684.2012	3.4	7209
76	685.2016	2.2	4711
77	685.4337	19.1	40838
78	686.4371	9.3	19876
79	687.4396	2.4	5057
80	709.3780	6.0	12925
81	710.3818	3.0	6362
82	711.3786	3.1	6553
83	743.9042	2.2	4756
84	745.9028	2.3	4880
85	750.4052	24.8	53216
86	751.4082	13.1	27978
87	752.4050	14.8	31611
88	753.3534	8.0	17204
89	753.4071	6.7	14312
90	754.3571	3.7	7997
91	755.3546	8.2	17510
92	756.3570	3.4	7288
93	784.5402	2.1	4601
94	794.3799	5.4	11633
95	795.3833	2.7	5885
96	796.3802	5.7	12232
97	797.3837	2.4	5078
98	1328.4394	3.2	6904
99	1329.4416	2.3	4921
100	1330.4425	2.2	4651

Acquisition Parameter

**General**	Fore Vacuum	3.17e+000 mBar	High Vacuum	1.27e-007 mBar	Source Type	ESI
	Scan Begin	75 m/z	Scan End	2000 m/z	Ion Polarity	Positive
**Source**	Set Nebulizer	2.0 Bar	Set Capillary	4500 V	Set Dry Gas	8.0 l/min
	Set Dry Heater	200 °C	Set End Plate Offset	-500 V		
**Quadrupole**	Set Ion Energy (MS only)	4.0 eV				
**Coll. Cell**	Collision Energy	8.0 eV	Set Collision Cell RF	600.0 Vpp	100.0 Vpp	
**Ion Cooler**	Set Ion Cooler Transfer Time	75.0 µs	Set Ion Cooler Pre Pulse Storage Time	10.0 µs		

Bruker Compass DataAnalysis 4.0 Acquisition Date 02.11.2022 10:09:52 Page 3 of 3

Figure S69: HR-MS ESI spectrum of **3b**

High Resolution Mass Spectrometry Report

Sample Name	**JK monoBr Zn pyr**	Instrument	maXis 4G
Comment		Method	ms_nocolumn_75-1000_pos.m

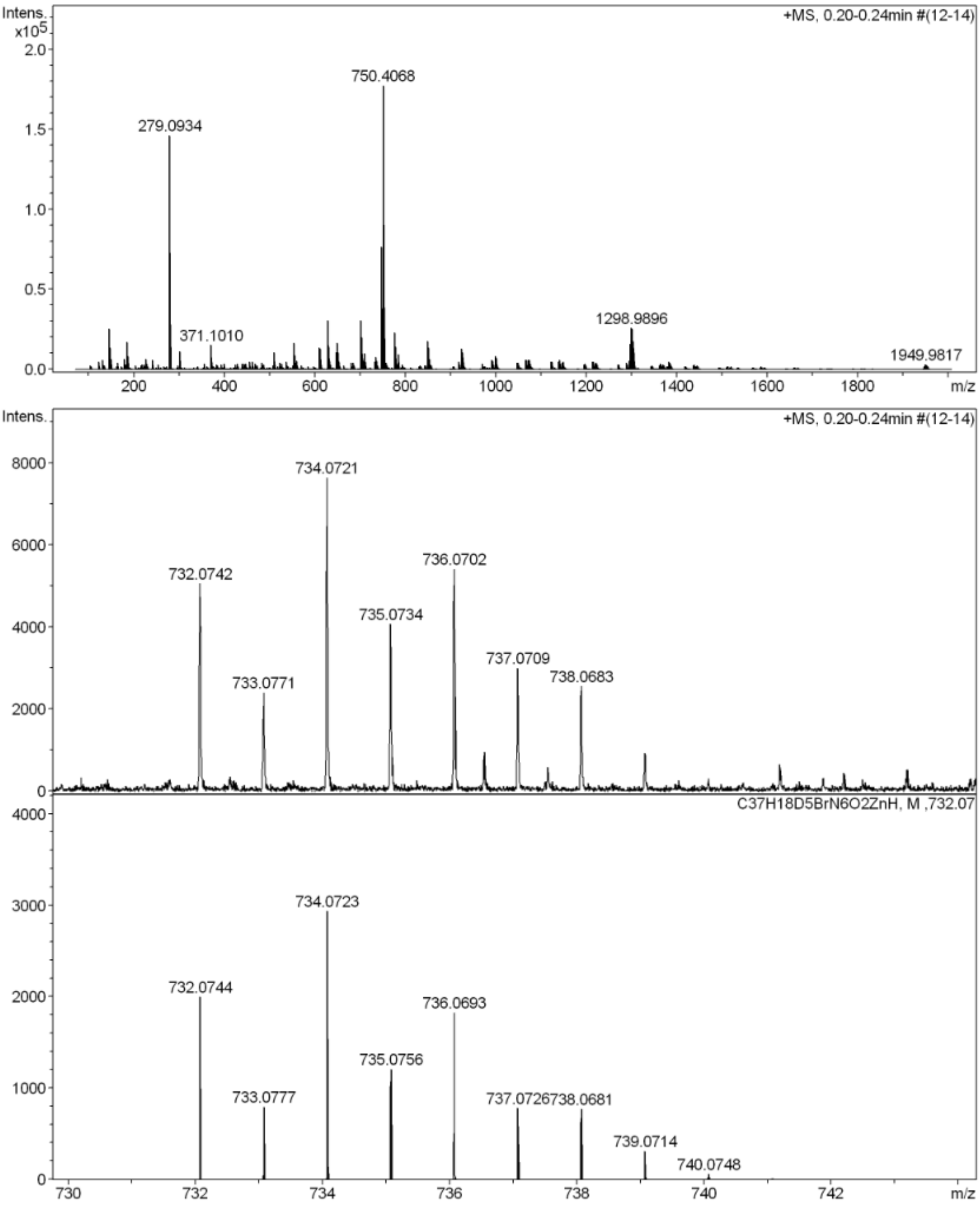

High Resolution Mass Spectrometry Report

Measured m/z vs. theoretical m/z

Meas. m/z	#	Formula	Score	m/z	err [mDa]	err [ppm]	mSigma	rdb	e⁻ Conf	z
732.0742	1	C 37 H 19 Br D 5 N 6 O 2 Zn	100.00	732.0744	0.2	0.2	57.5	28.5	even	1+

Mass list

#	m/z	I %	I
1	122.0965	2.7	4799
2	131.9621	3.2	5736
3	144.9822	14.2	25298
4	146.9803	7.5	13409
5	150.1276	3.7	6576
6	178.1588	3.6	6447
7	186.0087	9.8	17327
8	188.0069	4.7	8429
9	226.9514	3.3	5794
10	227.0353	3.6	6410
11	242.2838	3.5	6172
12	279.0934	82.3	146191
13	280.0964	16.1	28565
14	301.0748	6.3	11251
15	371.1010	8.7	15373
16	372.1014	2.8	5052
17	463.1296	2.9	5082
18	509.4354	6.1	10847
19	536.1645	2.8	4913
20	554.1751	9.5	16870
21	555.1759	5.0	8836
22	556.1737	3.5	6207
23	559.1311	2.9	5192
24	610.1836	7.6	13505
25	611.1867	7.5	13245
26	612.1855	4.3	7708
27	628.1942	17.3	30824
28	629.1949	10.0	17814
29	630.1927	8.1	14469
30	631.1933	3.6	6449
31	633.1488	3.2	5628
32	647.9995	6.0	10579
33	648.9962	3.1	5587
34	649.9976	9.4	16623
35	650.9987	4.5	8028
36	651.9953	6.0	10653
37	652.9974	2.8	5023
38	702.2124	17.4	30993
39	703.2135	11.4	20221
40	704.2115	8.5	15143
41	705.2108	3.9	6935
42	707.1689	3.1	5570
43	709.3794	5.7	10058
44	711.3790	2.8	5005
45	732.0742	2.8	5063
46	734.0721	4.3	7649
47	736.0702	3.1	5424
48	747.5268	43.1	76609
49	748.5297	21.4	38038
50	749.5327	6.1	10788
51	750.4068	100.0	177672
52	751.4098	46.9	83347
53	752.4063	50.4	89557
54	753.4084	21.9	38931
55	754.4108	5.3	9389
56	776.2315	13.0	23071
57	777.2320	10.3	18375
58	778.2295	8.0	14222
59	779.2296	4.3	7557
60	784.5414	5.5	9843
61	785.5448	3.7	6578
62	850.2498	10.0	17727

High Resolution Mass Spectrometry Report

#	m/z	I %	I
63	851.2505	7.7	13621
64	852.2487	6.6	11666
65	853.2479	3.6	6389
66	917.2966	2.8	4936
67	924.2691	7.3	13050
68	925.2690	6.1	10814
69	926.2671	5.4	9613
70	927.2663	3.4	6039
71	991.3151	3.4	5976
72	992.3158	3.0	5405
73	993.3148	2.8	4917
74	998.2875	4.5	7952
75	999.2875	4.4	7852
76	1000.2861	4.2	7490
77	1065.3337	3.2	5750
78	1066.3354	3.5	6169
79	1067.3342	3.3	5778
80	1072.3054	3.5	6146
81	1073.3069	3.3	5783
82	1074.3044	3.3	5886
83	1122.3427	2.9	5104
84	1139.3525	2.9	5098
85	1140.3526	3.2	5774
86	1141.3521	2.9	5222
87	1148.3223	2.7	4812
88	1214.3713	2.8	4951
89	1215.3724	2.7	4794
90	1294.9929	3.0	5343
91	1295.9930	2.6	4688
92	1296.9913	9.2	16273
93	1297.9930	7.6	13485
94	1298.9896	14.5	25771
95	1299.9911	10.6	18810
96	1300.9887	14.4	25592
97	1301.9894	9.6	17028
98	1302.9866	9.9	17678
99	1303.9873	6.2	10961
100	1304.9854	4.2	7549

Acquisition Parameter

**General**	Fore Vacuum	2.39e+000 mBar	High Vacuum	9.94e-008 mBar	Source Type	ESI
	Scan Begin	75 m/z	Scan End	2000 m/z	Ion Polarity	Positive
**Source**	Set Nebulizer	2.0 Bar	Set Capillary	4500 V	Set Dry Gas	8.0 l/min
	Set Dry Heater	200 °C	Set End Plate Offset	-500 V		
**Quadrupole**	Set Ion Energy (MS only)	4.0 eV				
**Coll. Cell**	Collision Energy	8.0 eV	Set Collision Cell RF	500.0 Vpp	100.0 Vpp	
**Ion Cooler**	Set Ion Cooler Transfer Time	75.0 µs	Set Ion Cooler Pre Pulse Storage Time	10.0 µs		

Figure S70: HR-MS ESI spectrum of **3-Zn-pyrd5**

High Resolution Mass Spectrometry Report

Sample Name **JK-606**
Comment

Instrument maXis 4G
Method ms_nocolumn_75-1000_pos.m

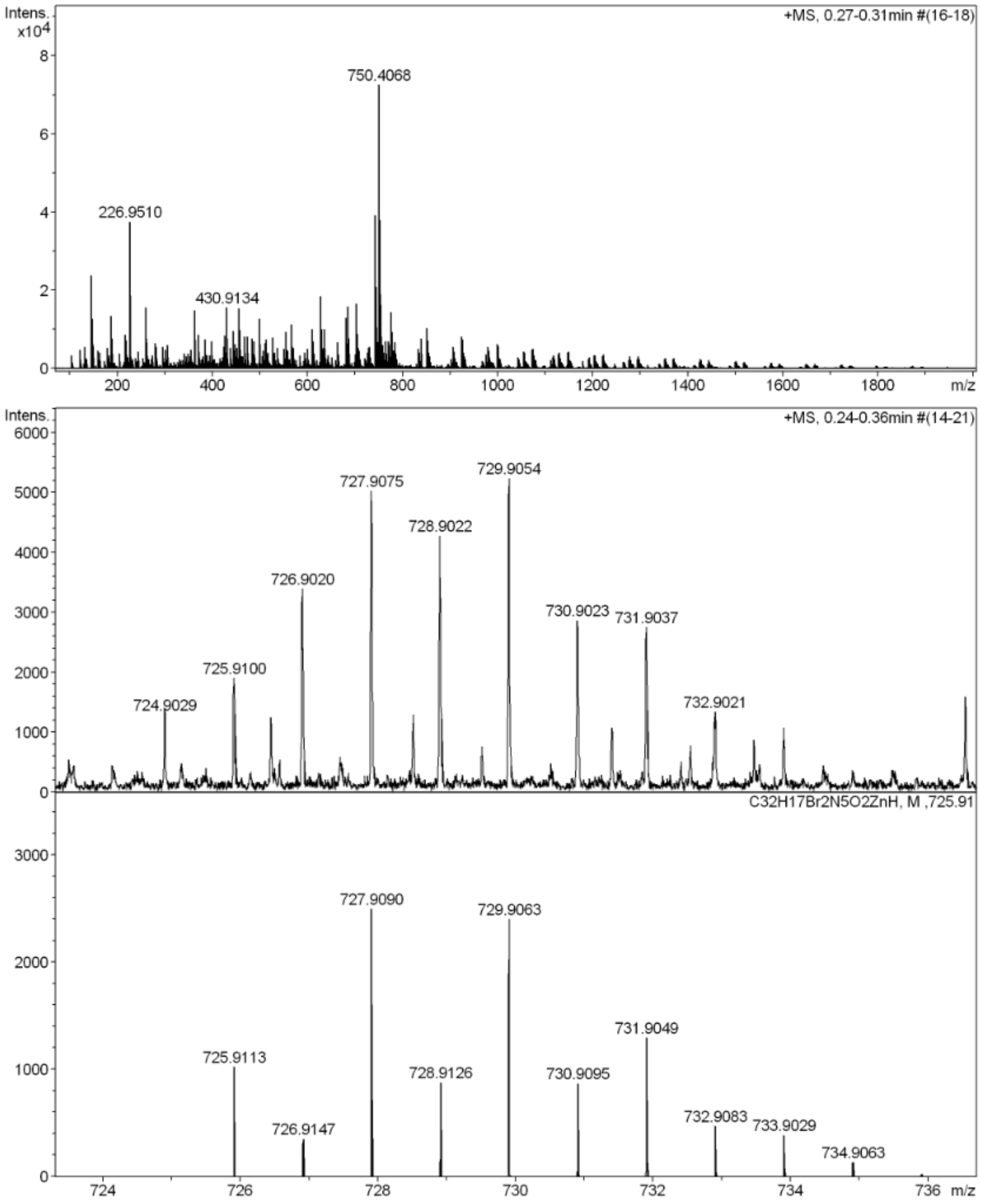

Bruker Compass DataAnalysis 4.0 Acquisition Date 27.02.2024 13:54:45 Page 1 of 3

High Resolution Mass Spectrometry Report

Measured m/z vs. theoretical m/z

Meas. m/z	#	Formula	Score	m/z	err [mDa]	err [ppm]	mSigma	rdb	e⁻ Conf	z
725.9100	1	C 32 H 18 Br 2 N 5 O 2 Zn	100.00	725.9113	1.4	1.9	216.8	25.5	even	1+

Mass list

#	m/z	I %	I
1	122.0961	7.3	4983
2	131.9618	8.7	5973
3	144.9819	35.9	24602
4	146.9800	19.7	13492
5	150.1273	8.4	5742
6	178.1585	8.4	5785
7	186.0082	20.2	13865
8	188.0065	11.1	7606
9	217.1043	12.2	8342
10	219.1867	11.0	7568
11	226.9511	54.8	37570
12	227.0349	10.0	6861
13	260.1064	22.0	15086
14	261.1301	9.4	6416
15	279.0929	9.9	6806
16	282.2785	7.7	5275
17	294.9385	7.8	5345
18	305.1567	7.8	5319
19	362.9259	21.1	14442
20	371.1006	12.9	8838
21	385.2918	10.7	7303
22	399.3072	9.8	6701
23	424.3625	8.5	5826
24	425.2142	12.0	8206
25	429.3181	17.0	11647
26	430.9134	23.0	15787
27	438.3785	8.0	5475
28	443.3340	13.2	9078
29	447.3438	7.9	5422
30	452.3211	7.7	5292
31	455.1882	22.6	15517
32	457.2768	13.5	9242
33	467.2457	8.5	5832
34	468.3887	12.3	8415
35	473.3445	11.3	7740
36	482.4041	11.4	7790
37	487.3599	9.9	6812
38	498.9011	18.1	12391
39	511.2719	9.2	6337
40	512.4152	11.3	7763
41	526.4306	11.8	8116
42	536.1648	9.0	6181
43	550.3428	7.8	5329
44	554.1753	14.4	9886
45	555.1759	7.4	5042
46	555.2984	9.6	6584
47	556.4416	7.4	5041
48	566.8886	17.1	11745
49	570.4569	8.2	5622
50	610.1836	15.4	10544
51	611.1848	10.6	7244
52	612.1826	7.3	4996
53	628.1945	28.5	19568
54	629.1948	15.3	10494
55	630.1930	12.0	8241
56	633.1494	7.4	5042
57	634.8761	14.5	9911
58	663.4539	10.1	6927
59	680.4797	18.8	12893
60	681.4830	8.3	5711
61	684.2021	8.3	5691
62	685.4349	21.5	14720

High Resolution Mass Spectrometry Report

#	m/z	I %	I
63	686.4381	10.3	7070
64	702.2133	25.6	17565
65	702.8636	13.2	9015
66	703.2138	17.1	11693
67	704.2117	12.9	8829
68	705.5821	15.1	10383
69	706.5854	7.3	5028
70	707.1687	7.8	5352
71	727.9075	7.3	5032
72	729.9054	7.6	5239
73	742.4956	54.5	37390
74	743.4988	28.3	19415
75	744.5021	8.3	5723
76	748.5386	8.7	5959
77	750.4067	100.0	68542
78	751.4100	48.4	33150
79	752.4066	51.7	35414
80	753.4087	22.5	15397
81	758.2208	8.5	5800
82	764.5737	10.2	7012
83	770.8513	10.5	7168
84	776.2319	21.4	14648
85	777.2325	14.4	9878
86	778.2305	12.7	8689
87	784.5424	9.4	6471
88	832.2403	7.7	5261
89	838.8384	11.3	7738
90	850.2509	15.5	10620
91	851.2517	13.4	9192
92	852.2491	10.9	7497
93	906.2590	7.6	5189
94	924.2698	12.2	8332
95	925.2696	10.9	7480
96	926.2682	9.7	6626
97	980.2771	7.4	5046
98	998.2880	8.9	6119
99	999.2888	8.8	6057
100	1000.2867	8.6	5868

Acquisition Parameter

**General**	Fore Vacuum<br>Scan Begin	2.48e+000 mBar<br>75 m/z	High Vacuum<br>Scan End	9.84e-008 mBar<br>2000 m/z	Source Type<br>Ion Polarity	ESI<br>Positive
**Source**	Set Nebulizer<br>Set Dry Heater	2.0 Bar<br>200 °C	Set Capillary<br>Set End Plate Offset	4500 V<br>-500 V	Set Dry Gas	8.0 l/min
**Quadrupole**	Set Ion Energy (MS only)	4.0 eV				
**Coll. Cell**	Collision Energy	8.0 eV	Set Collision Cell RF	500.0 Vpp	100.0 Vpp	
**Ion Cooler**	Set Ion Cooler Transfer Time	75.0 µs	Set Ion Cooler Pre Pulse Storage Time	10.0 µs		

Bruker Compass DataAnalysis 4.0 Acquisition Date 27.02.2024 13:54:45 Page 3 of 3

Figure S71: HR-MS ESI spectrum of **3b-Zn-pyr**

High Resolution Mass Spectrometry Report

Sample Name **JK-385**
Comment

Instrument maXis 4G
Method ms_nocolumn_high_pos_use_acn.m

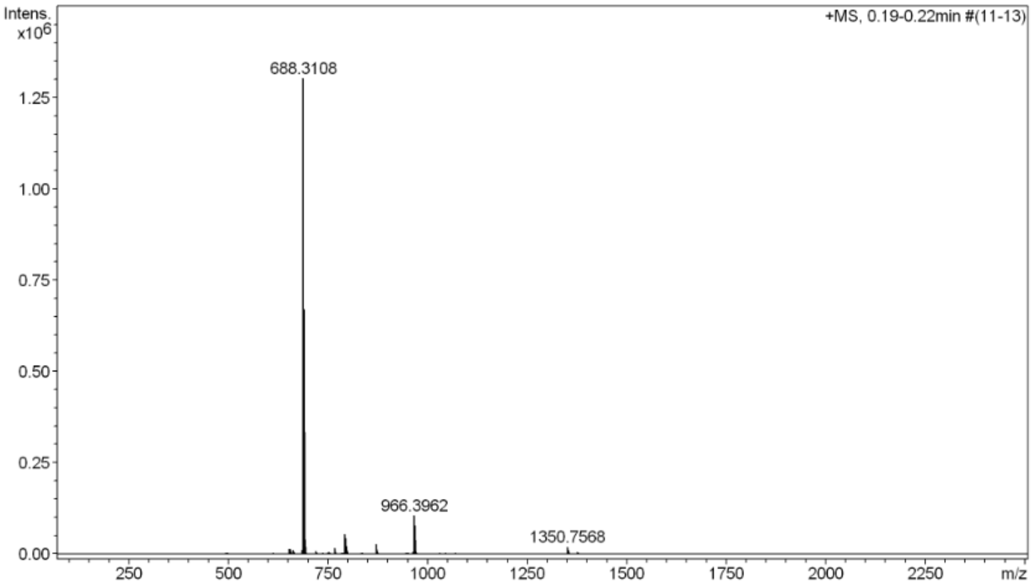

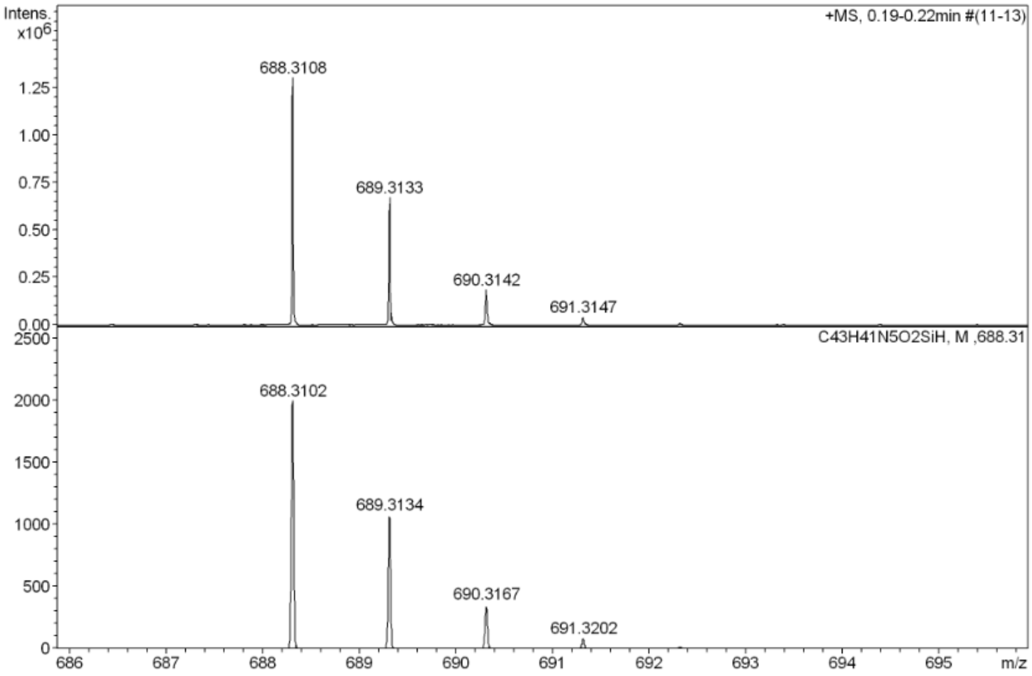

High Resolution Mass Spectrometry Report

Measured m/z vs. theoretical m/z

Meas. m/z	#	Formula	Score	m/z	err [mDa]	err [ppm]	mSigma	rdb	e⁻ Conf	z
688.3108	1	C 43 H 42 N 5 O 2 Si	100.00	688.3102	-0.6	-0.9	19.8	26.5	even	1+
1375.6125	1	C 86 H 83 N 10 O 4 Si 2	100.00	1375.6132	0.7	0.5	38.6	52.5	even	

Mass list

#	m/z	I %	I
1	492.9001	0.2	2361
2	493.9003	0.3	3837
3	494.8999	0.4	5097
4	496.8994	0.4	4972
5	498.9013	0.2	2587
6	579.1618	0.2	2201
7	611.3123	0.2	2761
8	612.2778	0.2	2770
9	613.3112	0.2	2474
10	651.3583	0.4	4982
11	652.3594	0.9	11810
12	653.3585	1.2	15557
13	654.3596	0.6	7562
14	655.3580	1.1	14142
15	656.3601	0.5	6281
16	657.3589	0.6	7196
17	658.3615	0.3	3336
18	663.4525	0.9	11991
19	664.4564	0.5	5981
20	685.4342	0.9	11985
21	686.4378	0.4	5628
22	687.3024	0.3	4234
23	688.3108	100.0	1302803
24	688.6023	0.2	2253
25	688.6648	0.2	2236
26	688.6971	0.2	2296
27	688.7413	0.2	2556
28	688.8039	0.2	2153
29	688.9675	0.2	2910
30	689.3133	51.6	672207
31	690.3142	14.3	186030
32	691.3147	3.2	41876
33	692.3163	0.6	7491
34	694.3854	0.2	3006
35	696.3837	0.2	2366
36	720.2991	0.6	7979
37	721.3019	0.3	4264
38	736.5415	0.2	3140
39	750.3465	0.2	2764
40	751.3468	0.3	4337
41	752.2886	0.6	7835
42	753.2920	0.3	4285
43	753.3462	0.3	3287
44	765.3366	0.2	2505
45	766.3409	1.3	17405
46	767.3426	0.8	10726
47	768.3446	0.3	3991
48	784.5412	0.3	4542
49	785.5446	0.2	2772
50	787.9625	0.2	2864
51	788.9622	0.3	4485
52	790.3727	1.2	16182
53	790.9616	0.3	3396
54	791.3741	3.2	42287
55	792.3741	4.3	55844
56	793.3764	1.8	23301
57	794.3735	3.5	45668
58	795.3758	1.5	20116
59	796.3749	1.7	22150
60	797.3769	0.7	9524
61	798.3306	0.4	5602

#	m/z	I %	I
62	798.3806	0.2	2491
63	798.5576	0.2	2516
64	799.3338	0.2	2754
65	833.4926	0.2	2784
66	834.4989	0.2	2198
67	835.4976	0.2	2977
68	837.4991	0.2	2407
69	870.4584	2.2	28391
70	871.4609	1.6	21310
71	872.4618	0.7	9067
72	873.4628	0.2	2812
73	946.4838	0.2	3169
74	947.4853	0.3	3881
75	948.4855	0.2	2984
76	949.4845	0.3	3350
77	950.4851	0.2	2535
78	951.4846	0.2	2364
79	963.5207	0.5	5871
80	964.5249	0.3	3990
81	966.3962	8.2	106982
82	967.3992	6.2	80311
83	968.4011	2.6	33753
84	969.4029	0.7	9707
85	970.4042	0.2	2460
86	1028.4349	0.2	2441
87	1029.4343	0.3	3364
88	1031.4344	0.2	2737
89	1044.4130	0.3	3318
90	1045.4141	0.3	3533
91	1070.4979	0.2	2901
92	1071.4997	0.2	2155
93	1148.5443	0.2	2152
94	1350.7568	1.6	21343
95	1351.7597	1.6	20541
96	1352.7615	0.9	11919
97	1353.7627	0.3	4266
98	1375.6125	0.5	6133
99	1376.6162	0.5	6623
100	1377.6173	0.3	4185

Acquisition Parameter

**General**	Fore Vacuum<br>Scan Begin	3.09e+000 mBar<br>75 m/z	High Vacuum<br>Scan End	1.12e-007 mBar<br>2500 m/z	Source Type<br>Ion Polarity	ESI<br>Positive
**Source**	Set Nebulizer<br>Set Dry Heater	2.0 Bar<br>200 °C	Set Capillary<br>Set End Plate Offset	4500 V<br>-500 V	Set Dry Gas	8.0 l/min
**Quadrupole**	Set Ion Energy (MS only)	4.0 eV				
**Coll. Cell**	Collision Energy	12.0 eV	Set Collision Cell RF	2000.0 Vpp	800.0 Vpp	
**Ion Cooler**	Set Ion Cooler Transfer Time	120.0 µs	Set Ion Cooler Pre Pulse Storage Time	18.0 µs		

Figure S72: HR-MS ESI spectrum of **4**

High Resolution Mass Spectrometry Report

Sample Name **JK-diTIPS-FB-Nirto**
Comment

Instrument maXis 4G
Method ms_nocolumn_75-1000_pos.m

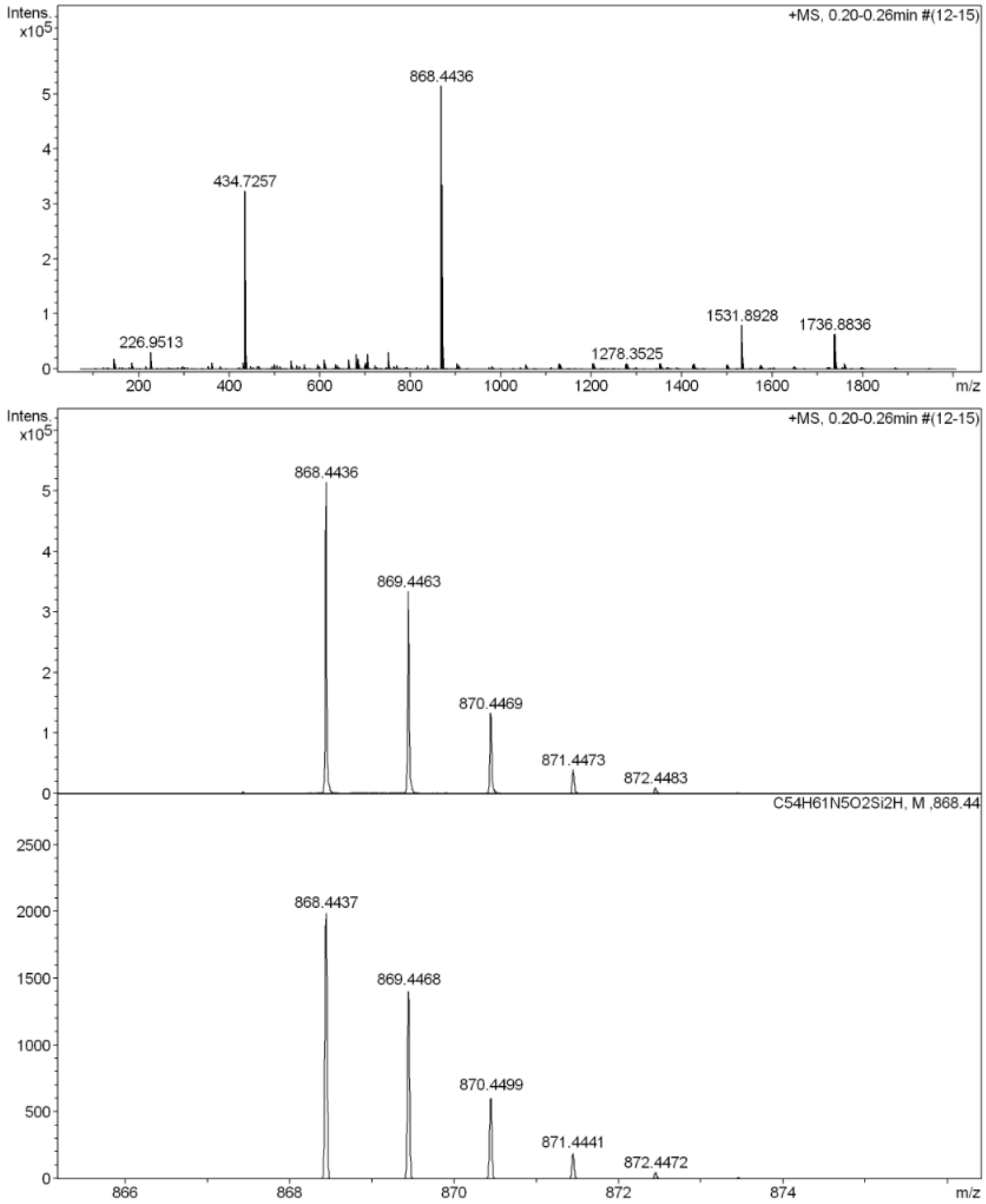

Bruker Compass DataAnalysis 4.0 Acquisition Date 28.02.2024 13:10:47 Page 1 of 3

High Resolution Mass Spectrometry Report

Measured m/z vs. theoretical m/z

Meas. m/z	#	Formula	Score	m/z	err [mDa]	err [ppm]	mSigma	rdb	e⁻ Conf	z
868.4436	1	C 54 H 62 N 5 O 2 Si 2	100.00	868.4437	0.0	0.0	39.0	28.5	even	1+
1735.8806	1	C 108 H 123 N 10 O 4 Si 4	100.00	1735.8800	-0.6	-0.3	10.7	56.5	even	
1757.8624	1	C 108 H 122 N 10 Na O 4 Si 4	100.00	1757.8620	-0.4	-0.3	37.2	56.5	even	

Mass list

#	m/z	I %	I
1	144.9823	3.7	18993
2	146.9804	2.0	10174
3	186.0086	2.3	11744
4	226.9513	6.1	31522
5	227.0350	1.8	9392
6	297.2341	1.2	6118
7	362.9259	2.3	11799
8	381.2968	1.2	6347
9	430.9132	2.5	13120
10	434.7257	63.0	324632
11	435.2269	39.3	202418
12	435.7271	15.9	82027
13	436.2273	5.0	25750
14	436.7280	1.4	7067
15	467.2453	1.2	6306
16	498.9008	1.8	9104
17	506.3161	1.5	7767
18	511.2718	1.2	6252
19	536.1646	3.0	15402
20	537.1653	1.4	7318
21	550.3430	1.5	7794
22	566.8878	1.8	9247
23	594.3686	1.7	8965
24	610.1831	3.3	17171
25	611.1836	2.0	10456
26	612.1818	1.5	7742
27	634.8756	1.7	8898
28	638.3945	1.4	7177
29	663.4523	3.3	16814
30	664.4563	1.5	7491
31	680.4791	5.4	27765
32	681.4836	2.8	14272
33	685.4347	3.8	19525
34	686.4379	1.8	9031
35	699.5943	1.3	6558
36	700.6257	2.5	12747
37	701.6295	1.3	6609
38	702.8629	1.7	8543
39	705.5816	5.3	27480
40	706.5847	2.7	13762
41	721.5731	1.3	6774
42	750.4058	6.1	31630
43	751.4091	3.4	17515
44	752.4054	3.3	17241
45	753.4084	1.5	7534
46	764.5725	1.2	6388
47	770.8501	1.5	7531
48	838.8376	1.5	7479
49	868.4436	100.0	515658
50	869.4463	65.0	335167
51	870.4469	26.0	133840
52	871.4473	7.9	40667
53	872.4483	2.0	10563
54	902.8151	2.1	10651
55	903.8186	1.3	6651
56	907.7707	1.4	7066
57	1054.2954	1.6	8307
58	1055.2967	1.5	7767
59	1056.2954	1.4	7233
60	1128.3139	1.8	9389

#	m/z	I %	I
61	1129.3156	2.0	10116
62	1130.3147	2.0	10253
63	1131.3139	1.5	7552
64	1202.3334	1.8	9320
65	1203.3343	2.2	11454
66	1204.3330	2.2	11358
67	1205.3329	1.6	8168
68	1276.3524	1.7	8973
69	1277.3529	2.2	11126
70	1278.3525	2.2	11511
71	1279.3505	1.7	8921
72	1280.3512	1.3	6488
73	1350.3702	1.6	8322
74	1351.3715	2.1	10666
75	1352.3710	2.1	10882
76	1353.3711	1.8	9196
77	1354.3685	1.2	6159
78	1424.3899	1.3	6940
79	1425.3908	1.9	9618
80	1426.3892	2.0	10493
81	1427.3895	1.6	8277
82	1428.3882	1.2	6078
83	1499.4093	1.5	7728
84	1500.4077	1.7	8992
85	1501.4088	1.5	7539
86	1530.8900	13.2	67896
87	1531.8928	15.6	80616
88	1532.8951	10.2	52528
89	1533.8961	4.5	23445
90	1534.8972	1.7	8855
91	1574.4271	1.4	7463
92	1575.4271	1.5	7590
93	1735.8806	9.1	46902
94	1736.8836	12.5	64409
95	1737.8854	10.0	51484
96	1738.8865	5.7	29384
97	1739.8873	2.6	13346
98	1757.8624	1.5	7778
99	1758.8651	2.1	10682
100	1759.8671	1.6	8054

Acquisition Parameter

**General**	Fore Vacuum	2.48e+000 mBar	High Vacuum	9.94e-008 mBar	Source Type	ESI
	Scan Begin	75 m/z	Scan End	2000 m/z	Ion Polarity	Positive
**Source**	Set Nebulizer	2.0 Bar	Set Capillary	4500 V	Set Dry Gas	8.0 l/min
	Set Dry Heater	200 °C	Set End Plate Offset	-500 V		
**Quadrupole**	Set Ion Energy (MS only)	4.0 eV				
**Coll. Cell**	Collision Energy	8.0 eV	Set Collision Cell RF	500.0 Vpp	100.0 Vpp	
**Ion Cooler**	Set Ion Cooler Transfer Time	75.0 µs	Set Ion Cooler Pre Pulse Storage Time	10.0 µs		

Figure S73: HR-MS ESI spectrum of **4b**

High Resolution Mass Spectrometry Report

Sample Name	**JK monoTIPS Zn Nitro**	Instrument	maXis 4G
Comment		Method	ms_nocolumn_75-1000_pos.m

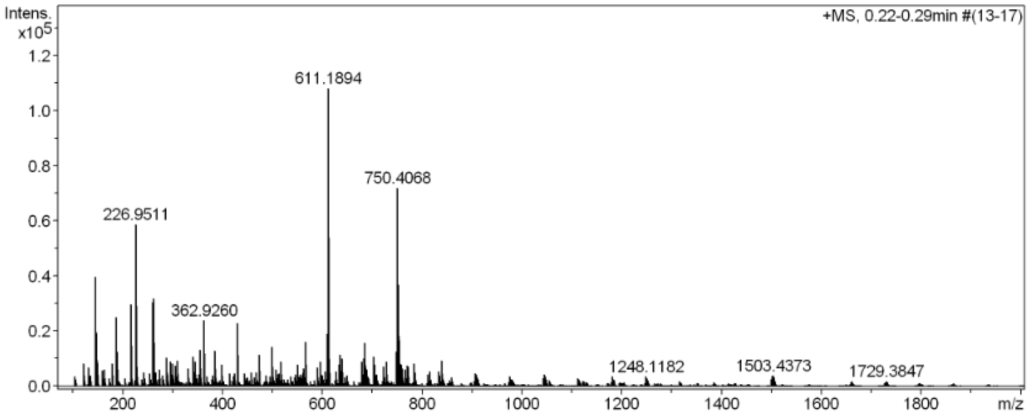

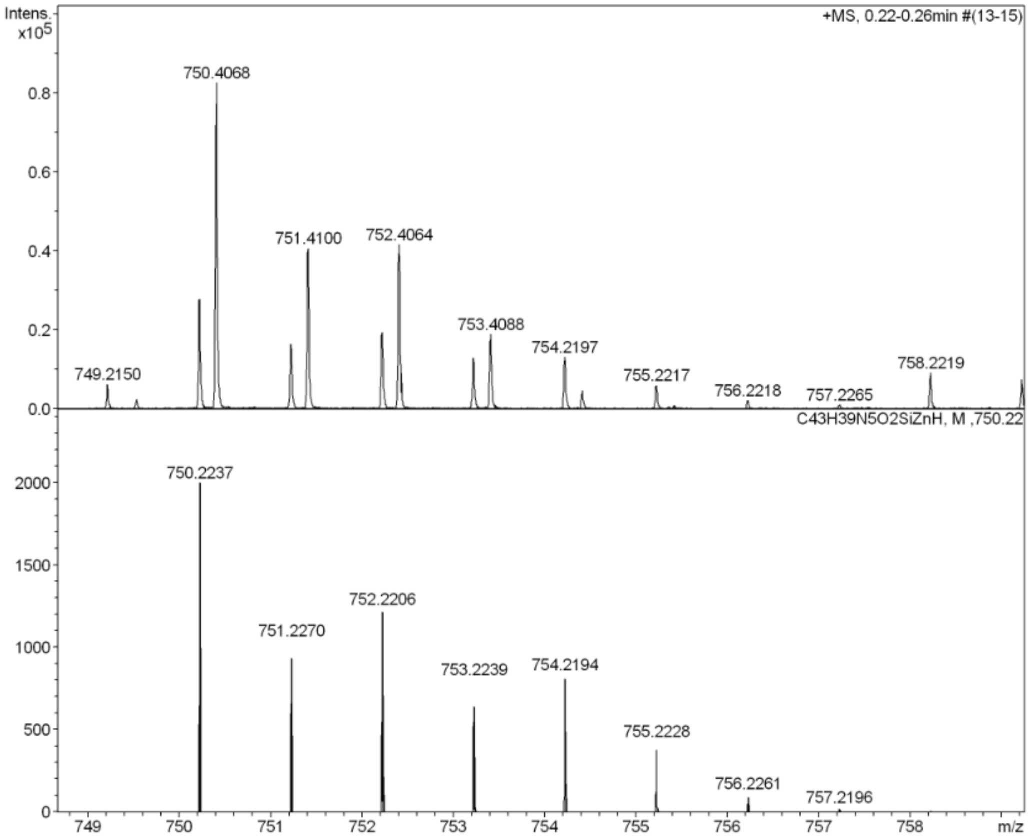

High Resolution Mass Spectrometry Report

Measured m/z vs. theoretical m/z

Meas. m/z	#	Formula	Score	m/z	err [mDa]	err [ppm]	mSigma	rdb	e⁻ Conf	z
750.2224	1	C 43 H 40 N 5 O 2 Si Zn	100.00	750.2237	1.3	1.7	40.0	27.5	even	1+
1499.4401	1	C 86 H 79 N 10 O 4 Si 2 Zn 2	100.00	1499.4402	0.1	0.0	86.1	54.5	even	

Mass list

#	m/z	I %	I
1	121.9660	4.0	5203
2	122.0962	6.9	8960
3	131.9616	5.0	6573
4	144.9818	29.5	38484
5	146.9799	14.1	18355
6	150.1272	6.5	8465
7	158.9636	4.4	5701
8	164.1428	4.7	6128
9	178.1584	6.5	8532
10	186.0082	18.3	23911
11	188.0065	9.5	12414
12	217.1042	26.2	34270
13	226.9511	40.8	53205
14	227.0348	11.3	14772
15	229.0327	5.3	6902
16	242.2839	4.8	6207
17	260.1065	28.5	37209
18	261.1097	6.3	8165
19	261.1304	28.1	36680
20	273.1665	5.6	7295
21	288.2526	9.4	12226
22	290.2683	4.0	5257
23	294.9386	6.1	7947
24	300.2524	7.7	10015
25	305.1564	7.0	9136
26	310.2345	8.3	10844
27	331.2086	5.8	7523
28	341.2660	9.8	12781
29	344.6583	8.2	10739
30	345.1599	4.2	5455
31	355.2813	12.0	15642
32	362.9259	15.9	20698
33	385.2918	11.8	15442
34	399.3075	6.7	8688
35	413.3227	4.1	5363
36	423.2194	4.1	5400
37	429.3179	11.8	15409
38	430.9134	14.7	19220
39	457.3492	4.2	5518
40	467.2458	4.5	5896
41	473.3441	10.2	13315
42	498.9009	9.0	11732
43	507.3285	5.7	7501
44	511.2722	4.1	5312
45	512.4149	4.2	5450
46	517.3702	8.1	10550
47	546.4005	4.2	5492
48	551.3545	7.5	9839
49	561.3967	6.0	7840
50	566.8882	10.3	13417
51	590.4259	6.3	8179
52	595.3811	8.4	10916
53	605.4220	5.0	6567
54	610.1836	16.2	21109
55	611.1895	100.0	130562
56	612.1919	43.3	56478
57	613.1939	9.3	12111
58	627.1669	5.3	6892
59	634.4522	7.3	9580
60	634.8756	6.8	8922
61	639.4067	9.5	12348

#	m/z	I %	I
62	640.4103	4.0	5182
63	678.4784	8.5	11114
64	683.4337	9.5	12454
65	684.2022	10.2	13300
66	685.2030	6.9	9040
67	685.4351	12.4	16213
68	686.2007	5.1	6625
69	686.4384	5.3	6964
70	688.3096	5.4	7103
71	689.0878	5.2	6799
72	702.8634	6.4	8336
73	705.5819	5.2	6796
74	722.5046	6.4	8399
75	727.4597	8.4	10998
76	747.5263	11.3	14767
77	748.5296	6.1	8027
78	749.2150	4.8	6306
79	750.2224	21.3	27795
80	750.4068	63.3	82654
81	751.2245	12.6	16486
82	751.4100	31.2	40795
83	752.2206	14.9	19436
84	752.4064	31.9	41715
85	753.2212	10.1	13159
86	753.4088	14.5	18991
87	754.2197	10.1	13173
88	755.2217	4.6	6061
89	758.2219	7.1	9295
90	759.2214	5.8	7594
91	760.2201	3.9	5088
92	766.5306	5.6	7315
93	770.8507	4.6	5972
94	771.4861	6.6	8574
95	784.5418	7.7	9992
96	785.5449	4.7	6128
97	810.5570	3.9	5089
98	815.5118	4.9	6388
99	832.2396	4.9	6417
100	838.8378	5.1	6641

Acquisition Parameter

**General**	Fore Vacuum	2.40e+000 mBar	High Vacuum	9.97e-008 mBar	Source Type	ESI
	Scan Begin	75 m/z	Scan End	2000 m/z	Ion Polarity	Positive
**Source**	Set Nebulizer	2.0 Bar	Set Capillary	4500 V	Set Dry Gas	8.0 l/min
	Set Dry Heater	200 °C	Set End Plate Offset	-500 V		
**Quadrupole**	Set Ion Energy (MS only)	4.0 eV				
**Coll. Cell**	Collision Energy	8.0 eV	Set Collision Cell RF	500.0 Vpp	100.0 Vpp	
**Ion Cooler**	Set Ion Cooler Transfer Time	75.0 µs	Set Ion Cooler Pre Pulse Storage Time	10.0 µs		

Figure S74: HR-MS ESI spectrum of **4-Zn**

High Resolution Mass Spectrometry Report

Sample Name **JK-605**
Comment

Instrument maXis 4G
Method ms_nocolumn_75-1000_pos.m

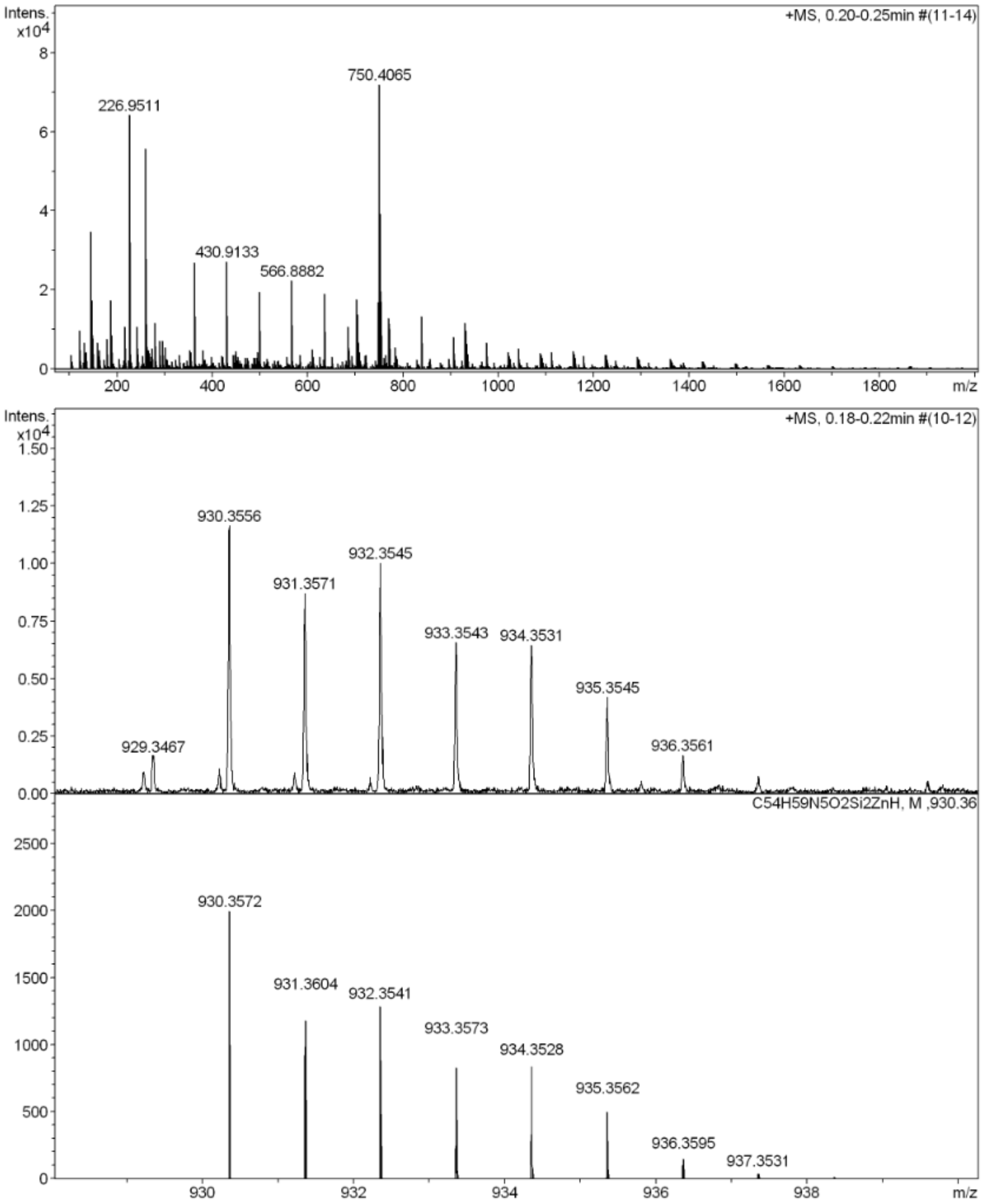

High Resolution Mass Spectrometry Report

Measured m/z vs. theoretical m/z

Meas. m/z	#	Formula	Score	m/z	err [mDa]	err [ppm]	mSigma	rdb	e⁻ Conf	z
930.3556	1	C 54 H 60 N 5 O 2 Si 2 Zn	100.00	930.3572	1.6	1.7	40.7	29.5	even	1+

Mass list

#	m/z	I %	I
1	103.9552	4.9	3500
2	121.9662	6.3	4491
3	122.0962	15.7	11251
4	131.9618	8.9	6418
5	133.9599	5.8	4182
6	136.1118	7.2	5166
7	144.9818	47.7	34212
8	146.9800	23.6	16934
9	150.1274	11.8	8496
10	158.9636	9.7	6980
11	164.1429	7.5	5407
12	178.1585	10.2	7355
13	186.0083	22.5	16132
14	188.0064	11.3	8143
15	216.9224	5.5	3973
16	217.1042	16.0	11507
17	226.9511	89.2	64069
18	227.0351	10.1	7276
19	229.0329	4.7	3378
20	242.2838	15.8	11373
21	243.9412	5.8	4175
22	254.0710	4.4	3153
23	260.1067	85.6	61434
24	261.1099	16.3	11678
25	261.1298	7.7	5541
26	265.8863	6.2	4460
27	265.9620	6.8	4871
28	266.0707	5.9	4212
29	267.8846	5.9	4232
30	270.9770	5.0	3585
31	273.1666	7.7	5557
32	279.0927	18.1	13026
33	290.2684	11.9	8571
34	294.9383	9.7	6982
35	301.0745	7.2	5196
36	331.2086	5.6	4020
37	353.2653	6.8	4917
38	355.2812	5.7	4095
39	362.9259	37.7	27048
40	381.2968	7.2	5189
41	399.3071	4.8	3431
42	420.8839	4.9	3534
43	429.3183	4.3	3116
44	430.9132	37.2	26669
45	443.3339	5.3	3824
46	447.3442	5.8	4142
47	447.9031	5.5	3980
48	449.2858	6.8	4901
49	453.7846	5.8	4131
50	493.3126	4.5	3237
51	494.8114	8.4	6059
52	498.9007	26.5	19034
53	566.8882	30.7	22043
54	583.8782	5.3	3771
55	610.1835	6.9	4983
56	611.1850	4.3	3073
57	624.8469	4.6	3278
58	634.8754	25.9	18608
59	651.8653	4.3	3099
60	685.4348	13.5	9709
61	686.4379	6.5	4678
62	692.8345	4.8	3446

High Resolution Mass Spectrometry Report

#	m/z	I %	I
63	702.8628	23.4	16764
64	705.5816	20.1	14421
65	706.5850	10.1	7264
66	709.3790	5.7	4080
67	719.8520	4.6	3320
68	721.5748	5.2	3718
69	747.5258	23.3	16698
70	748.5291	12.2	8768
71	750.4064	100.0	71787
72	751.4094	47.2	33899
73	752.4061	54.8	39365
74	753.4082	23.8	17063
75	754.4114	5.9	4255
76	764.5725	4.8	3442
77	770.8499	18.1	12982
78	784.5418	8.8	6338
79	785.5447	5.1	3694
80	838.8376	17.5	12563
81	906.8251	10.7	7713
82	930.3556	16.2	11655
83	931.3571	12.1	8718
84	932.3545	14.0	10040
85	933.3543	9.2	6573
86	934.3531	9.0	6480
87	935.3545	5.9	4207
88	974.8126	8.9	6391
89	1020.3254	5.5	3926
90	1022.3241	4.7	3396
91	1042.7995	7.2	5176
92	1088.3115	5.8	4174
93	1089.3145	4.6	3319
94	1090.3097	4.9	3546
95	1110.7868	5.6	4042
96	1156.3005	6.2	4448
97	1158.2975	4.9	3547
98	1178.7736	4.3	3064
99	1224.2872	4.8	3454
100	1226.2859	4.7	3383

Acquisition Parameter

**General**	Fore Vacuum	2.39e+000 mBar	High Vacuum	9.94e-008 mBar	Source Type	ESI
	Scan Begin	75 m/z	Scan End	2000 m/z	Ion Polarity	Positive
**Source**	Set Nebulizer	2.0 Bar	Set Capillary	4500 V	Set Dry Gas	8.0 l/min
	Set Dry Heater	200 °C	Set End Plate Offset	-500 V		
**Quadrupole**	Set Ion Energy (MS only)	4.0 eV				
**Coll. Cell**	Collision Energy	8.0 eV	Set Collision Cell RF	500.0 Vpp	100.0 Vpp	
**Ion Cooler**	Set Ion Cooler Transfer Time	75.0 µs	Set Ion Cooler Pre Pulse Storage Time	10.0 µs		

Bruker Compass DataAnalysis 4.0 Acquisition Date 27.02.2024 12:29:09 Page 3 of 3

Figure S75: HR-MS ESI spectrum of **4b-Zn**

High Resolution Mass Spectrometry Report

Sample Name **JK-398**
Comment

Instrument maXis 4G
Method ms_nocolumn_mid_pos.m

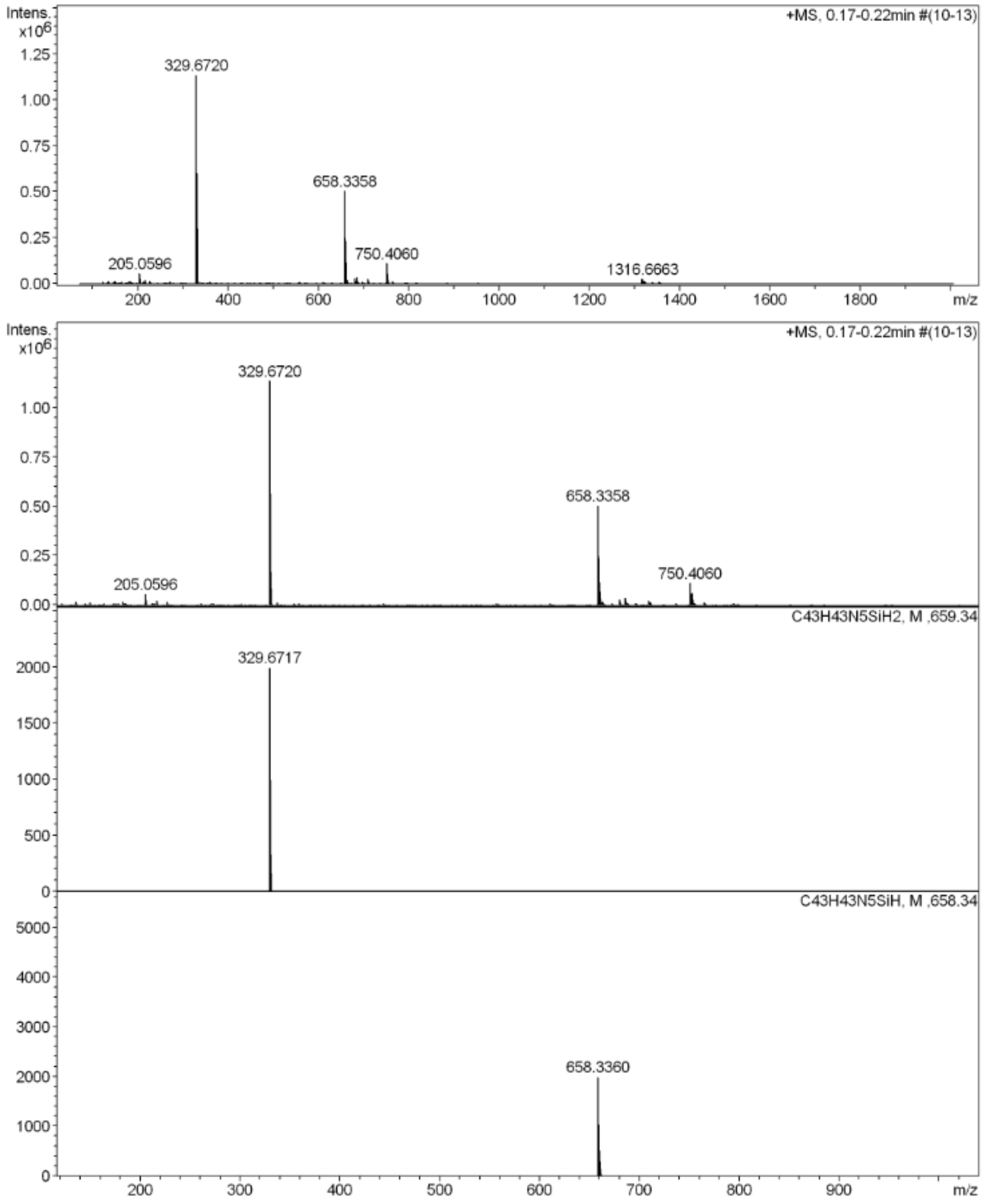

High Resolution Mass Spectrometry Report

Measured m/z vs. theoretical m/z

Meas. m/z	#	Formula	Score	m/z	err [mDa]	err [ppm]	mSigma	rdb	e⁻ Conf	z
329.6720	1	C 43 H 45 N 5 Si	100.00	329.6717	-0.3	-0.9	12.4	25.0	even	2+
658.3358	1	C 43 H 44 N 5 Si	100.00	658.3360	0.2	0.4	35.0	25.5	even	1+

Mass list

#	m/z	I %	I
1	122.0963	0.9	10110
2	131.9620	0.4	5049
3	136.1120	1.5	16982
4	144.9820	0.9	10474
5	146.9804	0.5	5199
6	147.0915	0.5	5213
7	150.1275	1.2	13326
8	164.1431	0.8	9341
9	173.0783	0.6	7239
10	175.1228	0.6	6602
11	178.1587	0.8	9656
12	183.0777	1.5	17252
13	185.1146	0.7	8145
14	186.0084	0.8	8694
15	188.0065	0.4	4905
16	205.0596	5.2	58734
17	212.1429	0.8	9360
18	213.1454	0.6	6887
19	215.1251	0.7	7902
20	217.1042	1.9	21970
21	226.1586	0.5	5673
22	226.9512	1.5	16952
23	227.0348	0.8	8862
24	229.1407	0.5	5417
25	242.9251	0.5	5199
26	261.1304	0.6	7185
27	265.9615	0.6	6328
28	271.1145	0.6	6868
29	271.1872	0.9	9846
30	273.1664	0.7	8419
31	279.0928	0.4	5003
32	301.0747	0.5	5690
33	329.6720	100.0	1136433
34	330.1730	53.2	604183
35	330.6741	15.0	170068
36	331.1753	3.2	36711
37	331.6764	0.6	7233
38	337.2339	1.1	12100
39	354.2842	0.6	6709
40	355.2823	0.4	4969
41	359.2394	0.9	10196
42	362.9251	0.5	5436
43	399.3070	0.5	6018
44	429.3175	0.5	5149
45	443.3331	0.6	6993
46	487.3592	0.4	5057
47	493.3127	0.5	5508
48	557.0936	0.7	7860
49	610.1829	0.9	10209
50	611.1839	0.5	5647
51	658.3358	44.4	504780
52	659.3384	20.9	237026
53	660.3403	6.2	70973
54	660.8936	0.8	9347
55	661.3429	1.5	16725
56	661.3949	0.8	9044
57	661.8970	0.5	5742
58	663.4522	1.7	19874
59	664.4557	0.8	9499
60	672.3142	0.6	6909
61	680.3165	2.6	29855

High Resolution Mass Spectrometry Report

#	m/z	I %	I
62	680.4791	0.7	7856
63	681.3192	1.3	15133
64	682.3233	0.4	4845
65	685.4343	3.2	36626
66	686.4375	1.5	16971
67	688.3083	0.6	6695
68	696.2906	1.0	11129
69	697.2931	0.6	6509
70	701.4083	0.5	5766
71	709.3794	2.1	23486
72	710.3821	1.0	11635
73	711.3784	1.1	12279
74	712.3807	0.5	5404
75	736.5407	0.7	7472
76	750.4060	9.9	112496
77	751.4089	4.8	54910
78	752.4057	5.3	60127
79	753.3547	0.6	6751
80	753.4079	2.4	26992
81	754.4104	0.6	6621
82	755.3550	0.6	7335
83	764.5728	1.2	13263
84	765.5757	0.6	6993
85	794.3777	0.6	6963
86	796.3800	0.5	5508
87	816.2914	0.5	5397
88	1315.6639	2.2	24881
89	1316.6663	2.3	26451
90	1317.6696	1.6	17692
91	1318.6729	0.8	8538
92	1320.7816	1.4	15571
93	1321.7843	1.4	15374
94	1322.7881	0.8	8566
95	1337.6457	0.9	10082
96	1338.6487	0.9	10712
97	1339.6503	0.6	6520
98	1353.6196	0.9	9924
99	1354.6226	0.9	10134
100	1355.6242	0.6	7140

Acquisition Parameter

**General**	Fore Vacuum<br>Scan Begin	3.17e+000 mBar<br>75 m/z	High Vacuum<br>Scan End	1.05e-007 mBar<br>2000 m/z	Source Type<br>Ion Polarity	ESI<br>Positive
**Source**	Set Nebulizer<br>Set Dry Heater	2.0 Bar<br>200 °C	Set Capillary<br>Set End Plate Offset	4500 V<br>-500 V	Set Dry Gas	8.0 l/min
**Quadrupole**	Set Ion Energy (MS only)	4.0 eV				
**Coll. Cell**	Collision Energy	8.0 eV	Set Collision Cell RF	600.0 Vpp	100.0 Vpp	
**Ion Cooler**	Set Ion Cooler Transfer Time	75.0 µs	Set Ion Cooler Pre Pulse Storage Time	10.0 µs		

Figure S76: HR-MS ESI spectrum of **5**

High Resolution Mass Spectrometry Report

Sample Name	**JK aniline Zn**	Instrument	maXis 4G
Comment		Method	ms_nocolumn_75-1000_pos.m

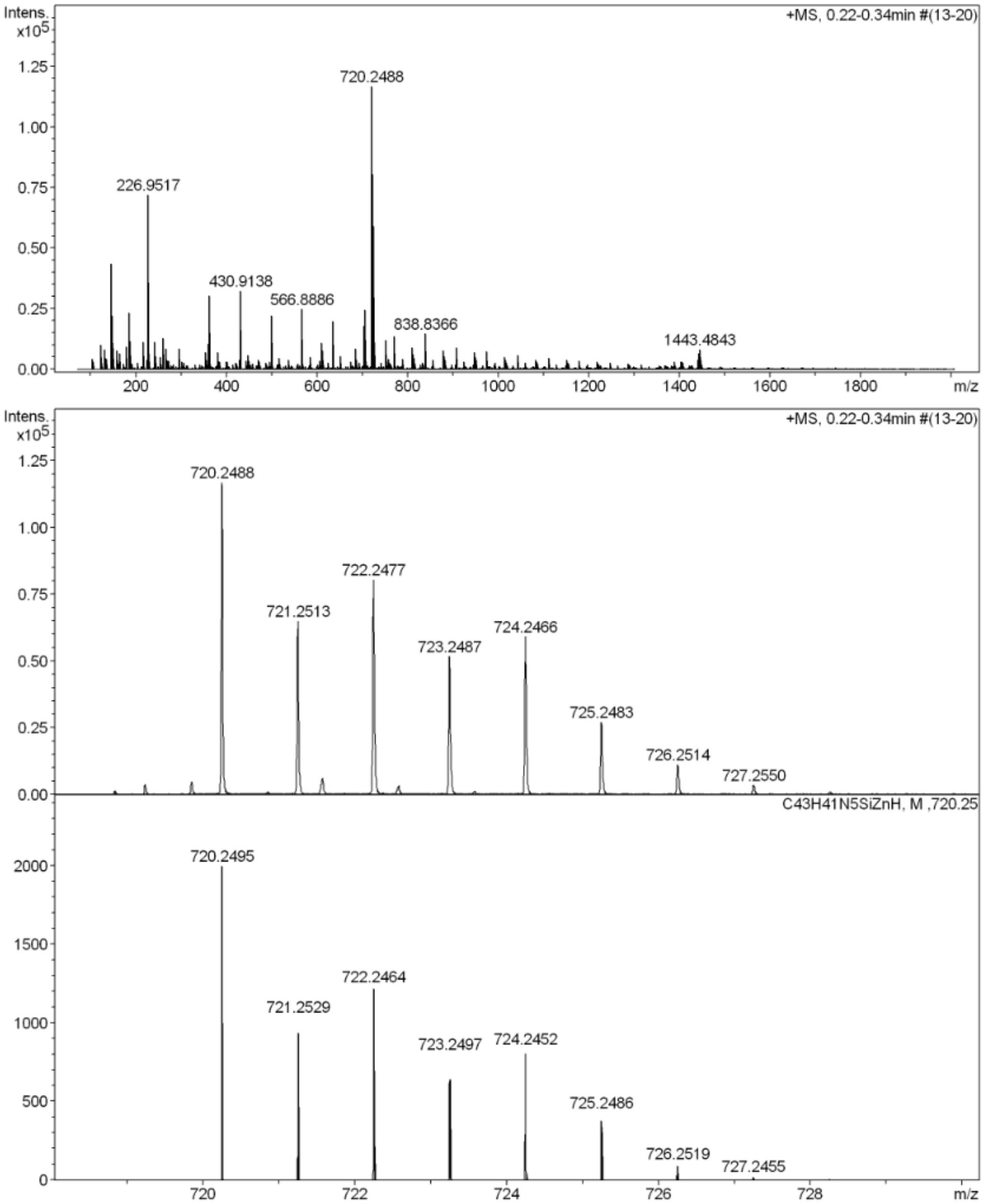

Bruker Compass DataAnalysis 4.0 Acquisition Date 27.02.2024 12:39:29 Page 1 of 3

High Resolution Mass Spectrometry Report

Measured m/z vs. theoretical m/z

Meas. m/z	#	Formula	Score	m/z	err [mDa]	err [ppm]	mSigma	rdb	e⁻ Conf	z
720.2488	1	C 43 H 42 N 5 Si Zn	100.00	720.2495	0.7	1.0	25.4	26.5	even	1+

Mass list

#	m/z	I %	I
1	103.9558	3.7	4305
2	121.9667	4.9	5690
3	122.0966	8.8	10291
4	131.9623	7.2	8447
5	133.9606	4.0	4637
6	136.1123	3.6	4268
7	144.9823	37.2	43524
8	146.9805	19.1	22290
9	150.1277	7.8	9083
10	158.9643	6.6	7752
11	164.1435	5.9	6883
12	178.1592	7.9	9285
13	186.0088	20.2	23627
14	188.0069	10.0	11674
15	217.1046	9.8	11439
16	226.9517	61.7	72233
17	227.0355	13.2	15490
18	229.0333	5.8	6729
19	242.2842	9.6	11204
20	243.9417	4.8	5585
21	254.0711	4.2	4956
22	260.1069	11.0	12913
23	265.9624	7.3	8559
24	266.0712	7.1	8339
25	294.9390	7.5	8803
26	353.2662	6.1	7090
27	355.2815	3.5	4073
28	360.6284	8.9	10444
29	361.1297	4.8	5617
30	361.6301	6.5	7579
31	362.1304	3.9	4508
32	362.6293	5.0	5841
33	362.9264	26.1	30522
34	381.2973	6.2	7255
35	430.9138	27.8	32473
36	447.9040	5.0	5897
37	469.9247	3.6	4168
38	498.9012	18.9	22075
39	515.8910	4.1	4804
40	537.9117	3.3	3915
41	566.8886	21.2	24839
42	583.8785	4.3	5034
43	610.1833	9.5	11146
44	611.1855	6.6	7758
45	612.1833	4.4	5126
46	634.8759	17.1	20023
47	651.8653	4.7	5457
48	684.2019	5.0	5811
49	685.2023	3.5	4072
50	685.4345	7.3	8519
51	686.4377	3.4	3949
52	702.8628	15.5	18178
53	705.5813	21.2	24746
54	706.5846	10.0	11666
55	719.2401	3.5	4092
56	719.8526	4.2	4872
57	720.2488	100.0	116986
58	721.2513	55.7	65112
59	721.5747	5.6	6568
60	722.2477	68.9	80644
61	723.2487	44.4	51911
62	724.2466	50.6	59215

High Resolution Mass Spectrometry Report

#	m/z	I %	I
63	725.2483	23.4	27421
64	726.2514	9.7	11398
65	750.4056	10.4	12117
66	751.4087	5.1	5933
67	752.4054	5.7	6686
68	758.2197	3.6	4253
69	770.8498	11.7	13708
70	787.8395	3.7	4271
71	810.2171	7.7	9037
72	811.2191	4.4	5113
73	812.2165	5.5	6453
74	813.2175	3.5	4097
75	814.2152	3.9	4608
76	838.8366	12.6	14710
77	855.8267	3.5	4044
78	878.2039	6.7	7867
79	879.2066	3.9	4510
80	880.2032	4.9	5696
81	882.2014	3.4	3963
82	906.8236	7.9	9196
83	946.1915	6.1	7149
84	947.1940	3.8	4484
85	948.1899	4.4	5180
86	950.1891	3.4	4003
87	974.8105	6.5	7588
88	1014.1778	4.4	5111
89	1016.1778	3.6	4158
90	1042.7981	5.2	6083
91	1082.1656	3.3	3901
92	1110.7840	4.0	4738
93	1150.1514	3.4	3926
94	1440.4854	3.5	4121
95	1441.4853	5.6	6603
96	1442.4854	5.5	6395
97	1443.4843	7.1	8305
98	1444.4860	5.5	6421
99	1445.4844	4.9	5775
100	1446.4871	3.3	3902

Acquisition Parameter

**General**	Fore Vacuum<br>Scan Begin	2.39e+000 mBar<br>75 m/z	High Vacuum<br>Scan End	9.94e-008 mBar<br>2000 m/z	Source Type<br>Ion Polarity	ESI<br>Positive
**Source**	Set Nebulizer<br>Set Dry Heater	2.0 Bar<br>200 °C	Set Capillary<br>Set End Plate Offset	4500 V<br>-500 V	Set Dry Gas	8.0 l/min
**Quadrupole**	Set Ion Energy (MS only)	4.0 eV				
**Coll. Cell**	Collision Energy	8.0 eV	Set Collision Cell RF	500.0 Vpp	100.0 Vpp	
**Ion Cooler**	Set Ion Cooler Transfer Time	75.0 µs	Set Ion Cooler Pre Pulse Storage Time	10.0 µs		

Bruker Compass DataAnalysis 4.0 Acquisition Date 27.02.2024 12:39:29 Page 3 of 3

Figure S77: HR-MS ESI spectrum of **5-Zn**

High Resolution Mass Spectrometry Report

Sample Name **JK-535**
Comment

Instrument maXis 4G
Method ms_nocolumn_high_pos_use_acn.m

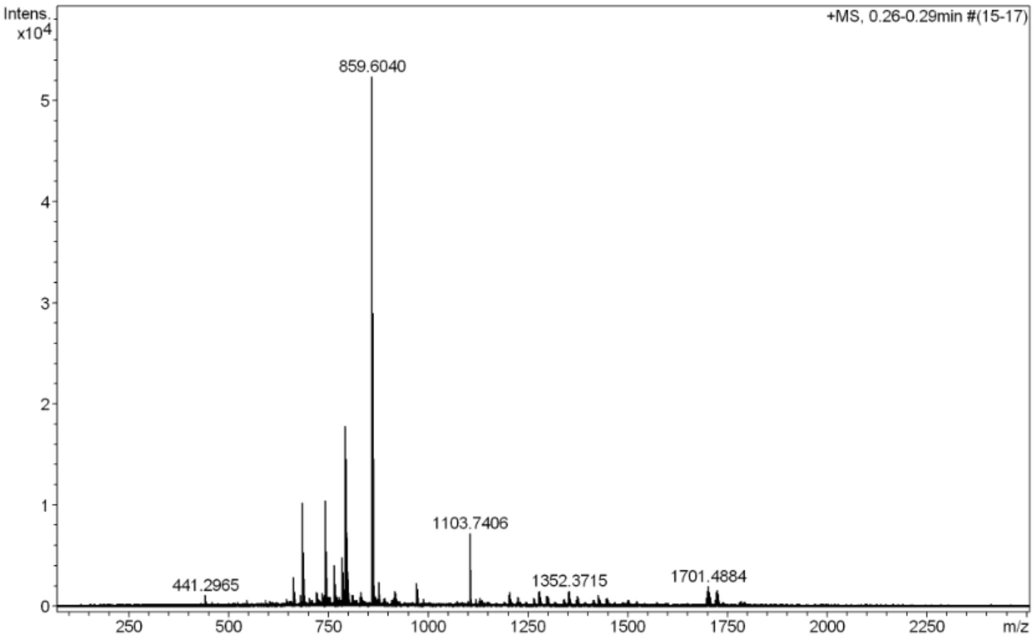

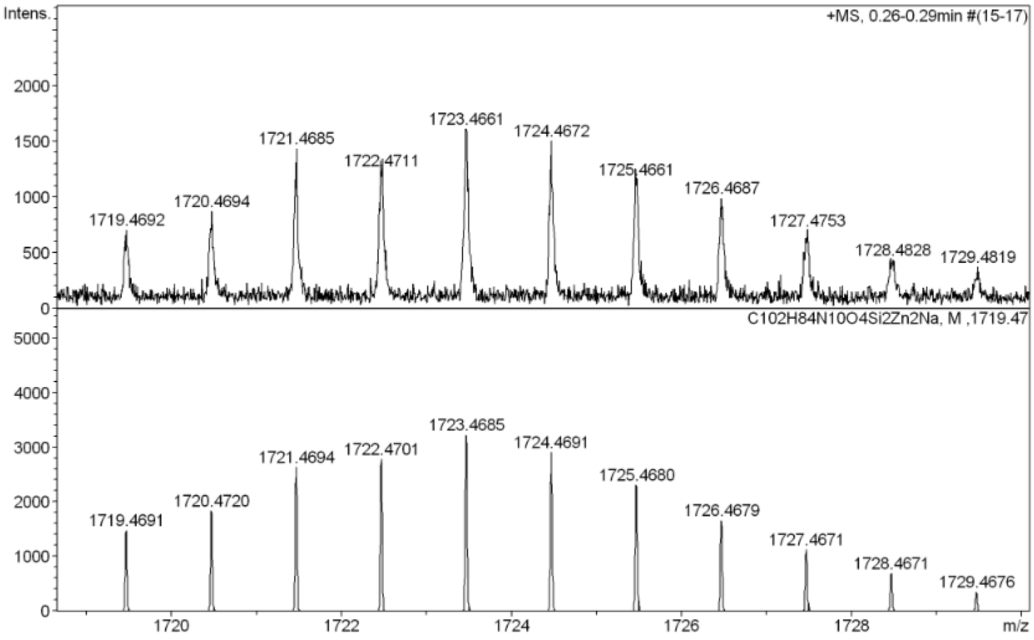

High Resolution Mass Spectrometry Report

Measured m/z vs. theoretical m/z

Meas. m/z	#	Formula	Score	m/z	err [mDa]	err [ppm]	mSigma	rdb	e⁻ Conf	z
1719.4692	1	C 102 H 84 N 10 Na O 4 Si 2 Zn 2	100.00	1719.4691	-0.1	-0.1	77.2	67.5	even	1+

Mass list

#	m/z	I %	I
1	441.2965	2.2	1166
2	663.4530	5.5	2899
3	664.4555	2.8	1462
4	680.4786	2.2	1161
5	685.4343	19.6	10261
6	686.4378	10.3	5388
7	687.4412	2.6	1345
8	689.4430	2.3	1199
9	720.2479	2.8	1469
10	721.5049	2.0	1026
11	722.5235	2.6	1348
12	733.4685	2.6	1368
13	738.5195	2.3	1212
14	742.4940	20.0	10475
15	743.4972	10.4	5442
16	744.5009	3.0	1578
17	750.3464	1.9	972
18	753.3475	1.9	988
19	764.5720	7.8	4068
20	765.5748	4.4	2286
21	770.5255	1.8	969
22	777.4951	1.8	943
23	784.5409	9.3	4870
24	785.5435	6.5	3390
25	786.5464	2.6	1384
26	790.3724	10.6	5534
27	790.5877	12.0	6273
28	791.3737	24.1	12651
29	791.5919	7.6	3989
30	792.3728	34.1	17884
31	792.6026	10.4	5447
32	793.3750	15.4	8090
33	793.6055	5.7	3009
34	794.3721	27.8	14595
35	794.6093	2.1	1102
36	795.3748	12.2	6379
37	796.3746	13.0	6829
38	797.3763	5.3	2796
39	798.3803	1.8	955
40	798.5557	5.2	2735
41	799.5580	3.3	1723
42	809.3063	2.3	1194
43	811.3052	2.1	1124
44	811.6043	1.8	966
45	829.3882	1.8	958
46	830.3890	2.9	1495
47	832.3889	2.4	1277
48	833.3961	1.9	981
49	859.6040	100.0	52428
50	860.6070	55.4	29058
51	861.6100	18.9	9926
52	862.6135	4.1	2162
53	868.3624	1.9	983
54	875.5783	4.6	2408
55	876.5837	3.1	1627
56	915.4691	1.8	966
57	916.4702	2.9	1537
58	917.4746	2.1	1094
59	918.4706	2.6	1374
60	971.7659	4.5	2346
61	972.7695	3.4	1800
62	1103.7406	13.9	7268

High Resolution Mass Spectrometry Report

#	m/z	I %	I
63	1104.7443	9.7	5075
64	1105.7482	4.1	2134
65	1202.3338	2.2	1167
66	1203.3314	2.4	1280
67	1204.3295	2.8	1450
68	1205.3299	2.1	1085
69	1276.3498	2.5	1305
70	1277.3510	2.7	1421
71	1278.3507	2.9	1533
72	1279.3487	2.5	1330
73	1280.3472	1.9	997
74	1297.2779	2.0	1040
75	1298.2823	2.1	1083
76	1299.2776	2.1	1115
77	1350.3695	2.1	1085
78	1351.3707	2.8	1449
79	1352.3715	3.1	1599
80	1353.3682	2.5	1305
81	1354.3686	1.9	1005
82	1372.2994	2.0	1030
83	1373.2969	2.0	1027
84	1374.2985	2.0	1044
85	1425.3892	2.0	1065
86	1426.3857	2.3	1205
87	1698.4826	2.2	1132
88	1699.4845	3.0	1594
89	1700.4854	2.9	1530
90	1701.4884	3.9	2026
91	1702.4859	3.2	1700
92	1703.4880	2.9	1505
93	1704.4853	2.3	1205
94	1705.4869	1.8	968
95	1721.4685	2.7	1441
96	1722.4711	2.4	1239
97	1723.4661	3.1	1613
98	1724.4672	2.9	1507
99	1725.4661	2.2	1160
100	1726.4687	1.9	995

Acquisition Parameter

**General**	Fore Vacuum<br>Scan Begin	3.15e+000 mBar<br>75 m/z	High Vacuum<br>Scan End	1.13e-007 mBar<br>2500 m/z	Source Type<br>Ion Polarity	ESI<br>Positive
**Source**	Set Nebulizer<br>Set Dry Heater	2.0 Bar<br>200 °C	Set Capillary<br>Set End Plate Offset	4500 V<br>-500 V	Set Dry Gas	8.0 l/min
**Quadrupole**	Set Ion Energy (MS only)	4.0 eV				
**Coll. Cell**	Collision Energy	12.0 eV	Set Collision Cell RF	2000.0 Vpp	800.0 Vpp	
**Ion Cooler**	Set Ion Cooler Transfer Time	120.0 µs	Set Ion Cooler Pre Pulse Storage Time	18.0 µs		

Figure S78: HR-MS ESI spectrum of **7-Zn**

High Resolution Mass Spectrometry Report

Sample Name	**DA 585**	Instrument	maXis 4G
Comment	manually infused (in MeOH+fa 0.1%)	Method	24 Direct_pos_high.m

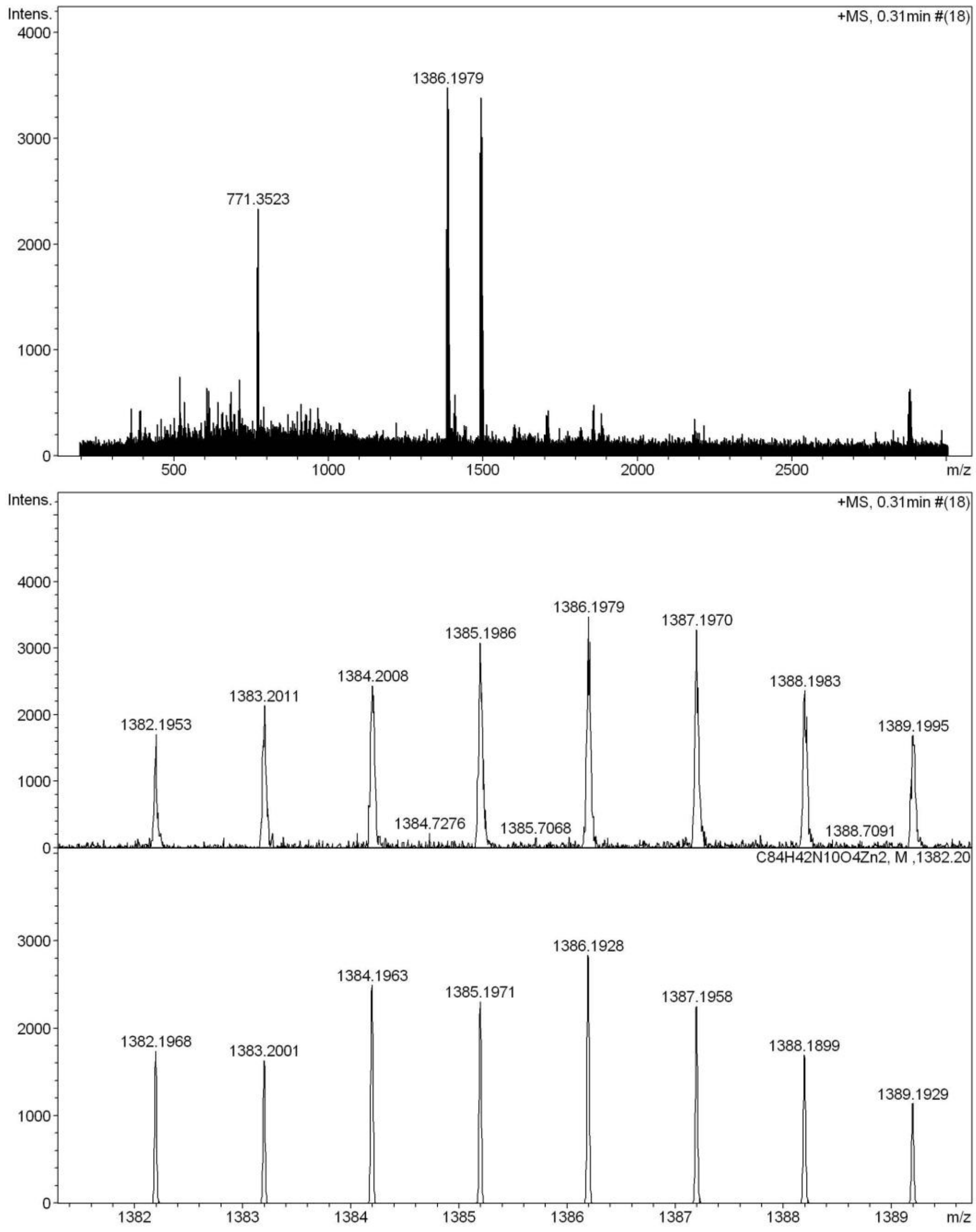

High Resolution Mass Spectrometry Report

Measured m/z vs. theoretical m/z

Meas. m/z	#	Formula	Score	m/z	err [mDa]	err [ppm]	mSigma	rdb	e⁻ Conf	z
1382.1953	1	C 84 H 42 N 10 O 4 Zn 2	100.00	1382.1968	1.5	1.1	96.1	69.0	odd	1+

Mass list

#	m/z	I %	I
1	363.1293	13.0	452
2	389.1583	12.2	424
3	391.1581	12.5	436
4	459.2191	10.1	353
5	499.0719	10.4	361
6	517.2944	20.1	701
7	519.2954	21.6	752
8	520.2989	10.5	364
9	533.2605	14.7	512
10	605.3107	12.0	418
11	607.5651	18.6	648
12	608.5660	11.7	407
13	613.4083	17.9	622
14	614.4253	13.3	462
15	641.4329	14.9	517
16	655.3742	11.0	384
17	657.3805	12.1	420
18	669.4596	10.5	366
19	673.1582	9.5	332
20	682.4024	14.1	492
21	683.4059	12.3	428
22	685.4309	14.6	508
23	693.4707	11.4	398
24	697.4955	11.4	398
25	709.4287	12.5	436
26	711.4357	20.7	722
27	721.5777	10.6	368
28	753.3489	9.7	336
29	769.3496	51.2	1782
30	770.3545	28.3	986
31	771.3523	67.2	2340
32	772.3515	21.7	756
33	781.5340	9.9	346
34	789.5018	13.6	472
35	815.7090	9.7	336
36	883.5943	9.6	334
37	899.6166	12.3	428
38	913.6228	9.5	332
39	923.6238	9.8	340
40	925.6320	11.6	402
41	927.6438	11.1	388
42	939.6458	11.6	402
43	941.6527	12.3	429
44	965.6601	13.2	461
45	967.6872	10.2	356
46	1382.1953	49.2	1713
47	1383.2011	61.6	2145
48	1384.2008	70.3	2445
49	1385.1986	88.8	3089
50	1386.1979	100.0	3480
51	1387.1970	94.3	3282
52	1388.1983	68.0	2366
53	1389.1995	48.8	1697
54	1390.1947	30.6	1064
55	1391.1958	26.9	936
56	1392.1985	11.9	414
57	1405.1839	11.6	405
58	1407.1852	10.4	362
59	1408.1876	16.8	586
60	1409.1854	12.2	426
61	1410.1855	14.3	496
62	1411.1915	10.9	381

High Resolution Mass Spectrometry Report

#	m/z	I %	I
63	1412.1893	9.7	337
64	1489.1021	36.8	1280
65	1490.1038	23.9	832
66	1491.0985	82.4	2868
67	1491.6085	17.9	624
68	1492.1014	78.9	2745
69	1492.6087	20.5	713
70	1493.1003	97.4	3390
71	1493.6012	32.5	1130
72	1494.1002	94.4	3284
73	1494.6074	18.0	628
74	1495.0982	86.7	3016
75	1495.5999	15.3	533
76	1496.1018	66.9	2327
77	1496.6101	9.9	345
78	1497.0988	49.4	1720
79	1498.1003	34.2	1190
80	1499.0981	13.2	460
81	1500.1027	9.7	338
82	1705.3828	11.1	386
83	1707.3830	10.9	378
84	1709.3929	11.5	400
85	1711.3878	12.5	436
86	1856.2351	10.0	347
87	1857.2332	11.7	406
88	1858.2361	13.3	464
89	1883.2704	11.6	405
90	2183.7070	10.2	356
91	2874.3066	10.6	369
92	2876.3022	11.5	400
93	2877.2982	16.1	560
94	2878.2954	17.4	606
95	2879.2922	15.7	545
96	2880.2965	18.4	639
97	2881.3068	15.1	526
98	2882.3013	13.9	483
99	2883.2998	15.0	522
100	2885.2930	10.9	380

Acquisition Parameter

**General**	Fore Vacuum	2.45e+000 mBar	High Vacuum	9.63e-008 mBar	Source Type	ESI
	Scan Begin	200 m/z	Scan End	3000 m/z	Ion Polarity	Positive
**Source**	Set Nebulizer	0.4 Bar	Set Capillary	3600 V	Set Dry Gas	4.0 l/min
	Set Dry Heater	180 °C	Set End Plate Offset	-500 V		
**Quadrupole**	Set Ion Energy (MS only)	4.0 eV				
**Coll. Cell**	Collision Energy	70.0 eV	Set Collision Cell RF	2000.0 Vpp	300.0 Vpp	
**Ion Cooler**	Set Ion Cooler Transfer Time	142.0 µs	Set Ion Cooler Pre Pulse Storage Time	22.0 µs		

Bruker Compass DataAnalysis 4.0 Acquisition Date 08.02.2024 11:19:54 Page 3 of 3

Figure S79: HR-MS ESI spectrum of **(rac)-PoGe-[Zn, Zn]**

High Resolution Mass Spectrometry Report

Sample Name	**JK-PoGe-FB**	Instrument	maXis 4G
Comment		Method	ms_nocolumn_600-3000_pos .m

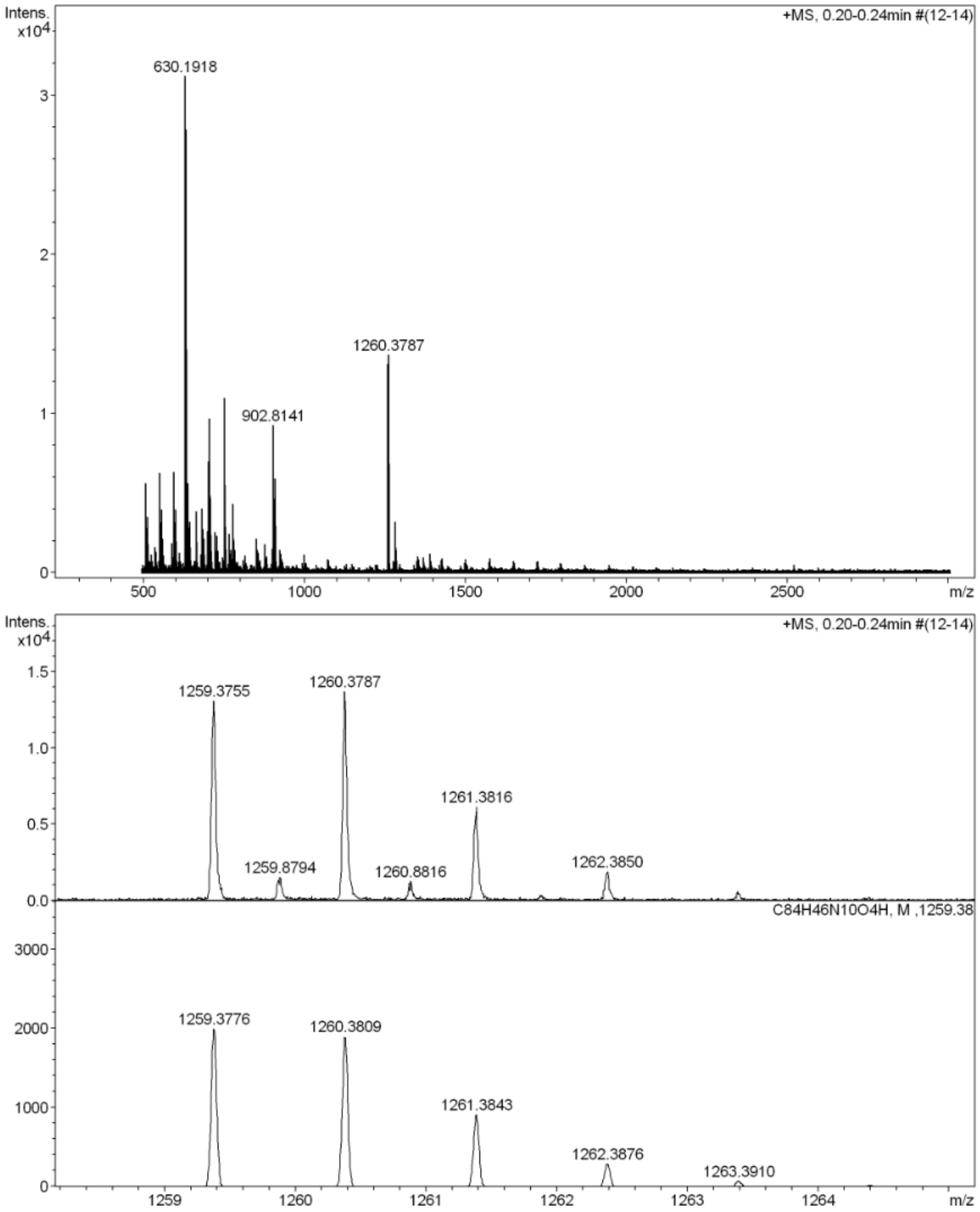

High Resolution Mass Spectrometry Report

Measured m/z vs. theoretical m/z

Meas. m/z	#	Formula	Score	m/z	err [mDa]	err [ppm]	mSigma	rdb	e⁻ Conf	z
1259.3755	1	C 84 H 47 N 10 O 4	100.00	1259.3776	2.1	1.7	30.6	66.5	even	1+

Mass list

#	m/z	I %	I
1	506.3159	18.2	5677
2	507.3202	4.3	1353
3	511.2721	11.3	3513
4	536.1645	5.3	1640
5	550.3423	20.2	6319
6	551.3467	6.5	2027
7	552.4090	6.0	1875
8	554.1744	9.3	2913
9	555.1755	4.7	1457
10	555.2976	12.8	3980
11	556.3006	5.0	1553
12	559.1304	5.8	1798
13	587.0976	5.6	1735
14	594.3683	20.3	6341
15	595.3726	6.2	1936
16	596.4361	6.6	2065
17	599.3226	12.8	3993
18	610.1831	4.1	1268
19	611.1806	4.0	1248
20	628.1935	23.1	7209
21	629.1943	14.1	4385
22	630.1918	100.0	31204
23	630.6933	89.3	27866
24	631.1949	40.8	12727
25	631.6961	13.8	4293
26	632.1956	5.4	1680
27	633.1493	8.6	2699
28	634.1504	4.7	1460
29	635.1473	4.2	1306
30	636.3782	4.0	1246
31	638.3943	18.1	5635
32	639.3977	6.1	1913
33	640.4618	6.6	2067
34	643.3497	10.4	3230
35	663.4521	12.5	3896
36	664.4557	6.4	2000
37	680.4056	4.5	1408
38	680.4786	9.0	2795
39	681.4864	5.1	1580
40	682.4201	13.0	4070
41	683.4246	4.7	1460
42	683.5985	4.7	1454
43	684.4877	6.9	2147
44	685.4333	8.8	2758
45	686.4369	4.2	1296
46	687.3758	6.9	2160
47	699.5931	8.6	2677
48	700.6251	21.2	6629
49	701.6277	10.9	3386
50	702.2122	22.5	7024
51	703.2131	12.6	3933
52	704.2111	10.9	3407
53	705.2102	5.9	1840
54	705.5811	31.1	9706
55	706.5840	15.1	4711
56	707.1683	8.6	2696
57	707.5844	5.3	1651
58	708.1686	5.8	1796
59	709.1649	4.5	1400
60	721.5729	8.2	2561
61	726.4463	7.5	2354
62	728.5130	4.8	1484

High Resolution Mass Spectrometry Report

#	m/z	I %	I
63	731.4018	4.6	1427
64	750.4052	35.3	11030
65	751.4082	19.2	5999
66	752.4057	19.4	6049
67	753.4088	9.5	2958
68	764.5717	7.9	2463
69	765.5757	4.1	1294
70	770.4714	4.6	1447
71	772.5394	4.5	1399
72	776.2306	12.3	3825
73	777.2318	9.1	2827
74	778.2286	7.8	2445
75	779.2295	4.7	1481
76	781.1863	6.6	2050
77	782.1875	4.7	1466
78	850.2488	7.0	2191
79	851.2500	6.8	2108
80	852.2475	4.8	1492
81	855.2070	4.1	1293
82	876.7981	5.9	1835
83	900.7983	4.9	1525
84	902.8141	29.8	9295
85	903.8175	21.6	6726
86	904.8242	8.2	2566
87	907.7698	19.1	5973
88	908.7723	11.0	3448
89	909.7804	5.9	1829
90	924.2685	4.7	1473
91	1259.3755	42.0	13120
92	1259.8794	5.0	1572
93	1260.3787	43.9	13714
94	1260.8816	4.1	1274
95	1261.3816	19.8	6178
96	1262.3850	6.2	1941
97	1281.3566	10.4	3249
98	1282.3603	9.5	2960
99	1283.3610	4.8	1497
100	1388.1743	3.9	1208

Acquisition Parameter

**General**	Fore Vacuum<br>Scan Begin	2.48e+000 mBar<br>500 m/z	High Vacuum<br>Scan End	9.70e-008 mBar<br>3000 m/z	Source Type<br>Ion Polarity	ESI<br>Positive
**Source**	Set Nebulizer<br>Set Dry Heater	0.4 Bar<br>180 °C	Set Capillary<br>Set End Plate Offset	4500 V<br>-500 V	Set Dry Gas	4.0 l/min
**Quadrupole**	Set Ion Energy (MS only)	4.0 eV				
**Coll. Cell**	Collision Energy	10.0 eV	Set Collision Cell RF	2000.0 Vpp	300.0 Vpp	
**Ion Cooler**	Set Ion Cooler Transfer Time	160.0 µs	Set Ion Cooler Pre Pulse Storage Time	15.0 µs		

Figure S80: HR-MS ESI spectrum of **(rac)-PoGe-[2H, 2H]**

High Resolution Mass Spectrometry Report

Sample Name	**JK-PoGe-Cu2**	Instrument	maXis 4G
Comment	direct infusion + MeOH+fa0.1%	Method	24 Direct_pos_high.m

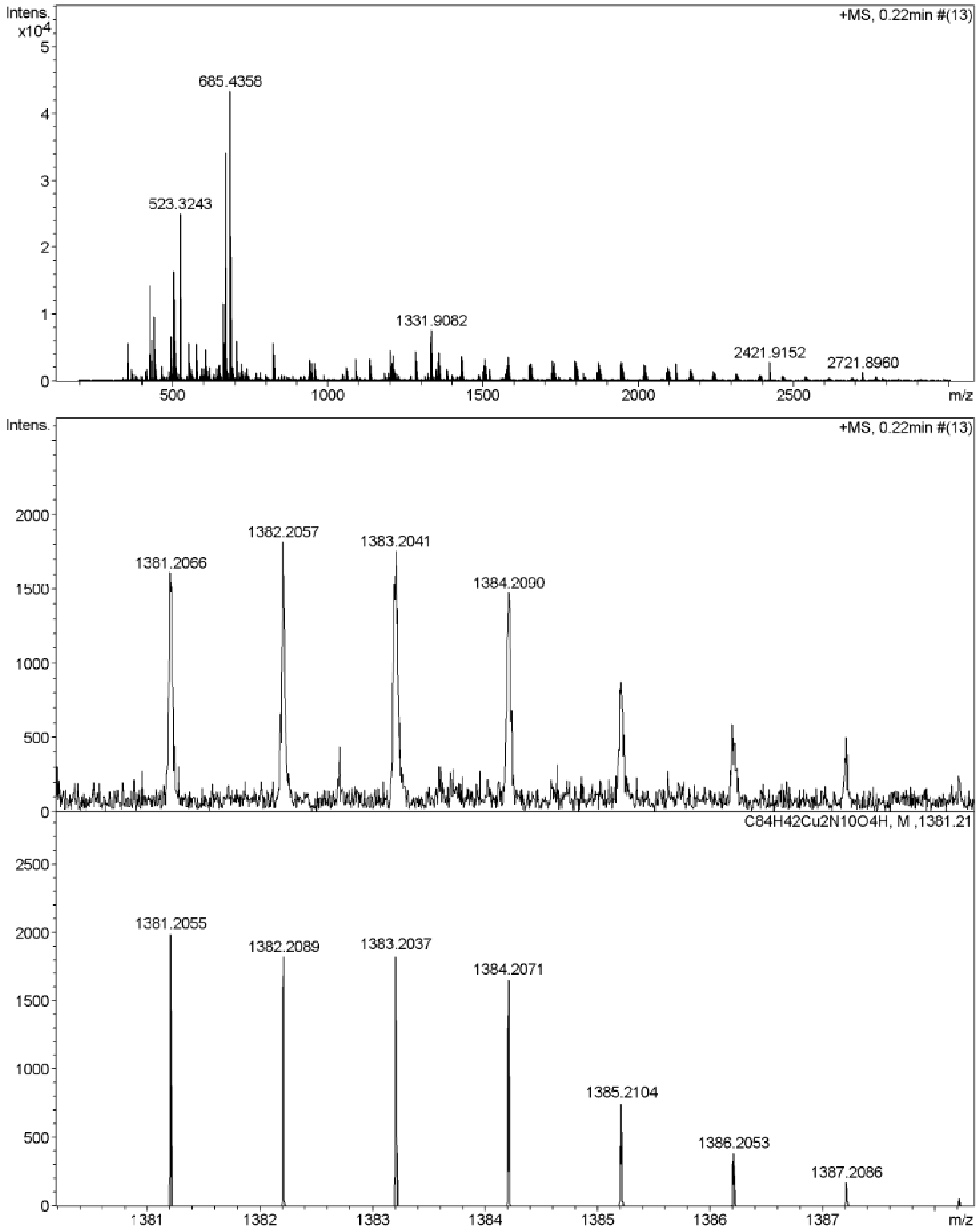

High Resolution Mass Spectrometry Report

Measured m/z vs. theoretical m/z

Meas. m/z	#	Formula	Score	m/z	err [mDa]	err [ppm]	mSigma	rdb	e⁻ Conf	z
1381.2066	1	C 84 H 43 Cu 2 N 10 O 4	100.00	1381.2055	-1.0	-0.7	212.4	67.5	even	1+

Mass list

#	m/z	I %	I
1	355.0719	13.2	5744
2	425.3238	9.5	4114
3	429.0891	32.9	14306
4	430.0901	14.7	6396
5	431.0874	9.7	4204
6	441.2978	22.2	9644
7	442.3014	6.3	2731
8	495.2661	15.6	6771
9	503.1078	37.8	16420
10	504.1082	17.6	7633
11	505.1060	12.0	5212
12	507.3507	28.3	12310
13	508.3543	10.0	4348
14	523.3243	57.8	25115
15	524.3275	15.0	6533
16	551.3285	13.2	5735
17	552.3327	5.4	2345
18	577.1264	13.0	5666
19	578.1271	7.4	3215
20	579.1260	6.4	2794
21	605.4242	5.4	2346
22	607.3914	10.9	4746
23	649.4502	5.6	2444
24	651.1455	6.0	2602
25	663.4537	26.9	11697
26	664.4571	14.4	6269
27	668.4605	6.3	2754
28	669.4622	78.7	34200
29	670.4654	36.8	15986
30	671.4693	8.1	3536
31	685.4358	100.0	43436
32	686.4390	42.9	18624
33	687.4445	11.7	5097
34	687.4692	11.2	4845
35	705.5835	14.0	6082
36	706.5868	5.9	2581
37	721.5775	6.3	2727
38	824.3018	12.6	5486
39	824.8034	12.1	5250
40	825.3048	9.3	4043
41	939.5972	7.4	3196
42	947.5474	6.4	2792
43	956.7071	6.4	2791
44	1087.7706	7.9	3410
45	1088.7740	6.1	2660
46	1133.2718	6.1	2630
47	1134.2723	7.9	3428
48	1135.2699	7.3	3152
49	1136.2703	5.7	2473
50	1199.7742	10.6	4584
51	1200.7776	8.7	3800
52	1207.2906	6.7	2895
53	1208.2906	9.0	3900
54	1209.2886	8.9	3886
55	1210.2900	6.6	2854
56	1281.3096	7.0	3057
57	1282.3099	9.2	4000
58	1283.3091	10.5	4578
59	1284.3086	7.0	3052
60	1285.3073	5.9	2581
61	1331.9082	17.7	7695
62	1332.9117	14.9	6463

High Resolution Mass Spectrometry Report

Sample Name	**DA 585**	Instrument	maXis 4G
Comment	manually infused (in MeOH+fa 0.1%)	Method	24 Direct_pos_high.m

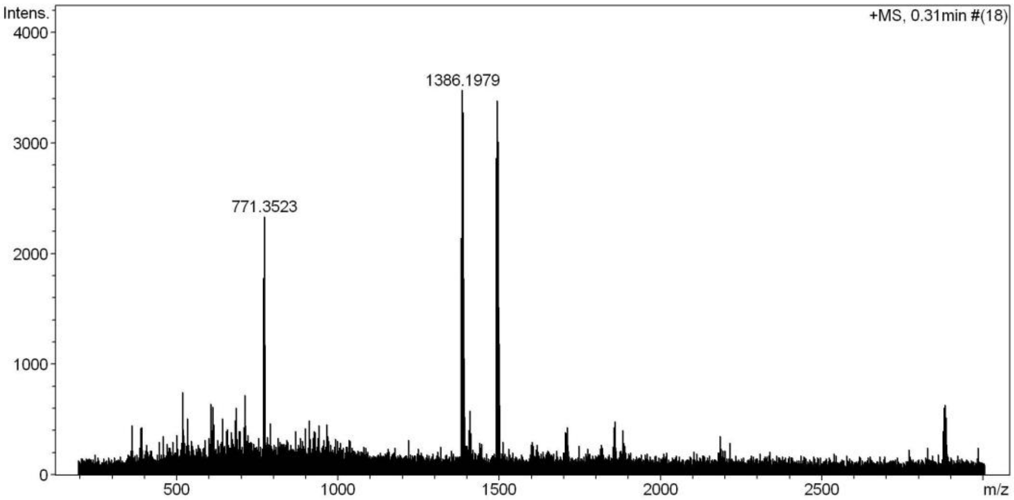

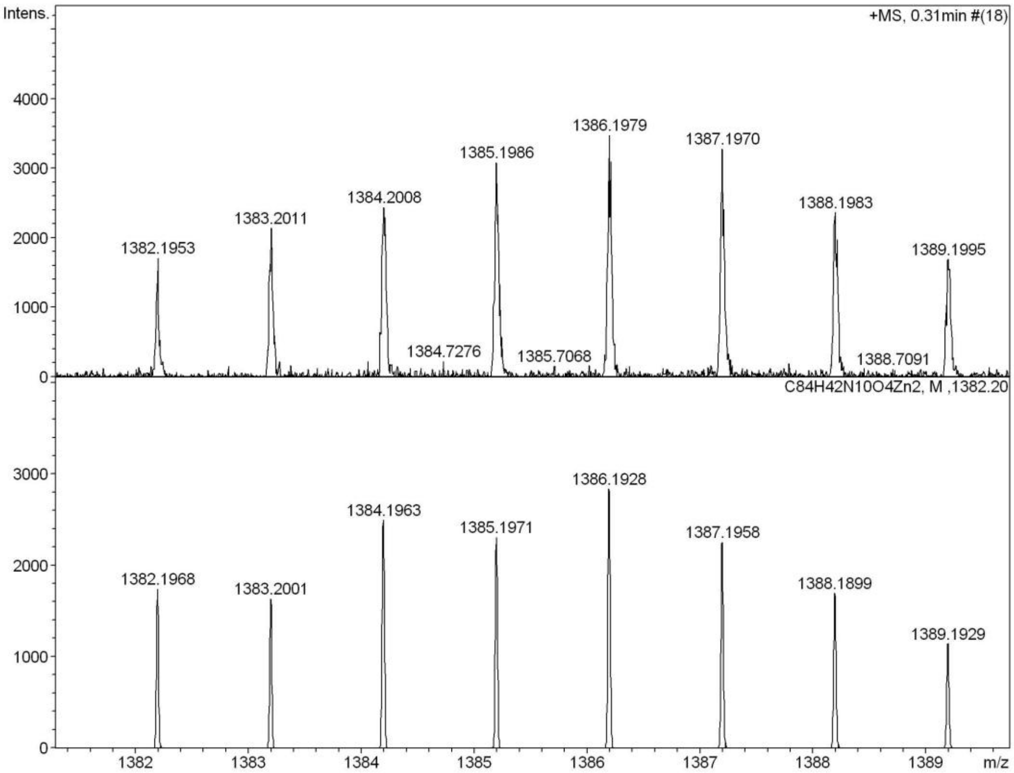

Bruker Compass DataAnalysis 4.0 Acquisition Date 08.02.2024 11:19:54 Page 1 of 3

High Resolution Mass Spectrometry Report

#	m/z	I %	I
63	1333.9143	6.1	2670
64	1355.3291	6.8	2953
65	1356.3282	7.8	3368
66	1357.3267	10.0	4326
67	1358.3286	7.9	3410
68	1359.3256	5.7	2467
69	1429.3472	6.7	2922
70	1430.3473	8.2	3582
71	1431.3447	8.7	3772
72	1432.3455	7.4	3214
73	1433.3450	6.0	2596
74	1503.3668	5.5	2410
75	1504.3664	7.1	3066
76	1505.3647	7.8	3397
77	1506.3645	6.6	2875
78	1578.3860	5.5	2403
79	1579.3837	8.4	3668
80	1580.3846	6.6	2861
81	1648.4452	5.7	2466
82	1649.4494	5.8	2504
83	1652.4089	5.6	2426
84	1653.4054	5.6	2449
85	1654.4043	5.7	2494
86	1722.4637	6.5	2830
87	1723.4652	7.1	3078
88	1727.4221	6.4	2780
89	1796.4835	7.0	3062
90	1797.4836	7.0	3024
91	1869.5020	5.6	2454
92	1870.5031	6.6	2872
93	1871.5044	7.0	3032
94	1872.5013	5.8	2541
95	1944.5234	5.9	2556
96	1945.5209	6.7	2925
97	2018.5383	6.0	2592
98	2020.5373	5.5	2368
99	2121.9345	6.4	2760
100	2421.9152	6.7	2909

Acquisition Parameter

**General**	Fore Vacuum<br>Scan Begin	2.48e+000 mBar<br>200 m/z	High Vacuum<br>Scan End	1.03e-007 mBar<br>3000 m/z	Source Type<br>Ion Polarity	ESI<br>Positive
**Source**	Set Nebulizer<br>Set Dry Heater	0.4 Bar<br>180 °C	Set Capillary<br>Set End Plate Offset	3600 V<br>-500 V	Set Dry Gas	4.0 l/min
**Quadrupole**	Set Ion Energy (MS only)	4.0 eV			300.0 Vpp	
**Coll. Cell**	Collision Energy	50.0 eV	Set Collision Cell RF	2000.0 Vpp		
**Ion Cooler**	Set Ion Cooler Transfer Time	142.0 µs	Set Ion Cooler Pre Pulse Storage Time	22.0 µs		

Figure S81: HR-MS ESI spectrum of **PoGe-[Cu, Cu]**

HPLC Chromatography

Np-HPLC purification

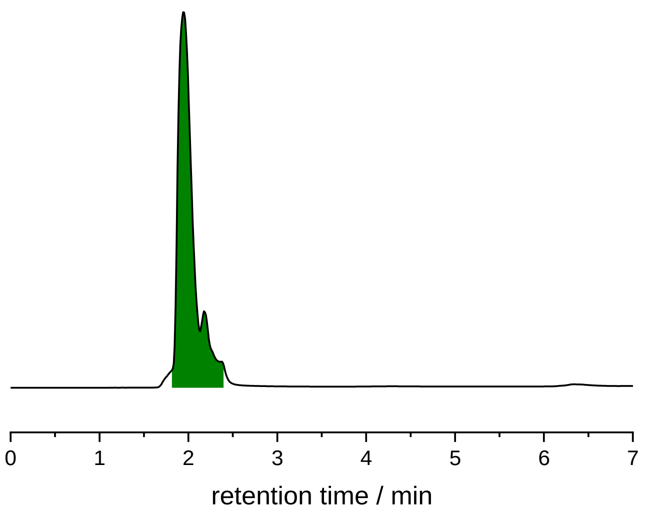

Figure S82: Purification of ***(rac)*-PoGe[Zn, Zn].** Conditions: 1% EtOH in heptane, ReproSil 20x250mm, 20 mL/min.

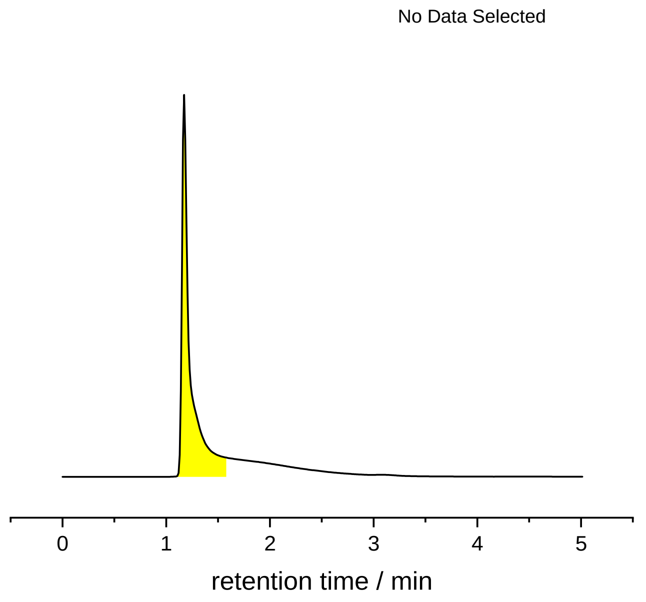

Figure S83: Purification of ***(rac)*-PoGe[Zn, Zn].** Conditions: 1% EtOH in heptane, ReproSil 4.6x250mm, 2 mL/min.

Chiral Resolution

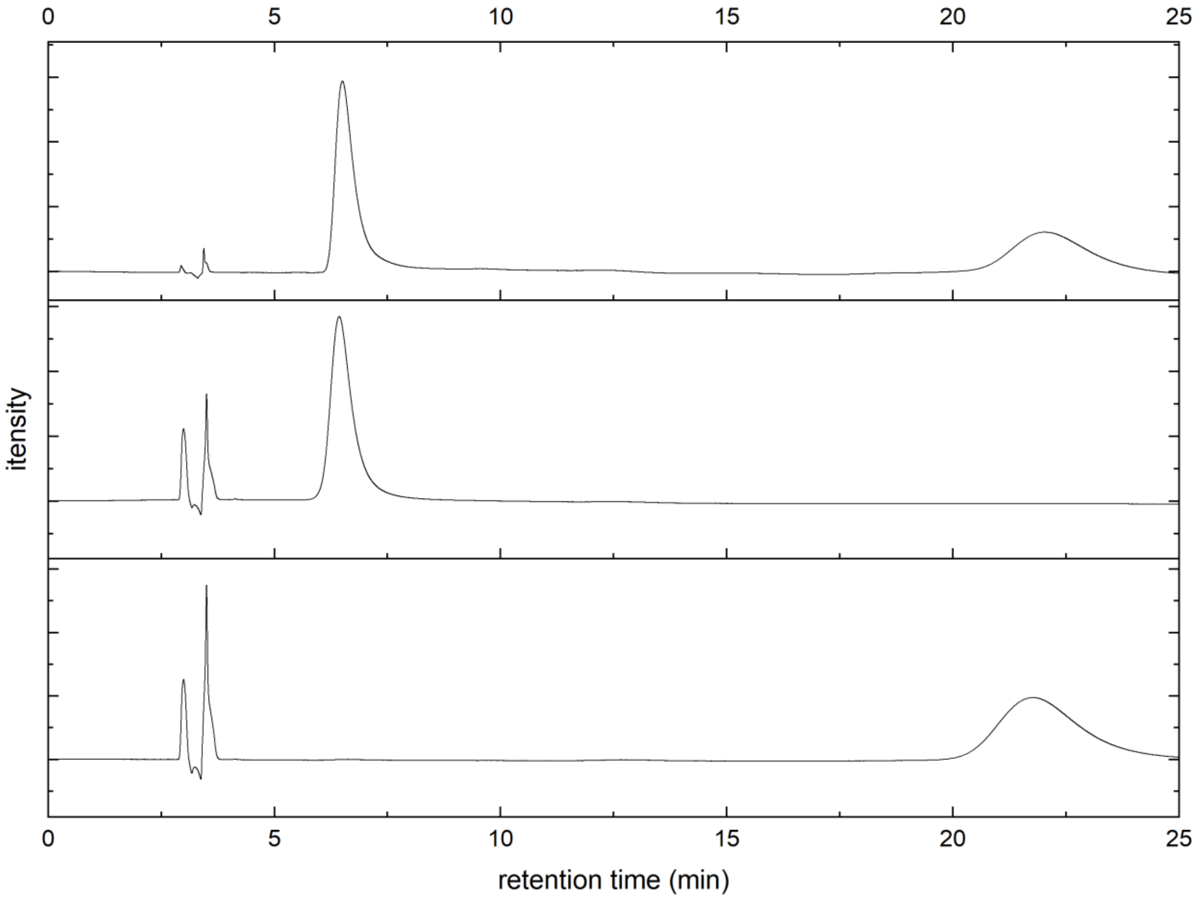

Figure S84: Chiral resolution of ***(rac)*-PoGe[Zn, Zn]** (top); injection of separated sample ***(P)*-PoGe[Zn, Zn]** (middle), injection of separated sample ***(M)*-PoGe[Zn, Zn]** (bottom). Conditions: 50% EtOAc in heptane, Chiralpak IG 4.6x250mm, 1.0 mL/min.

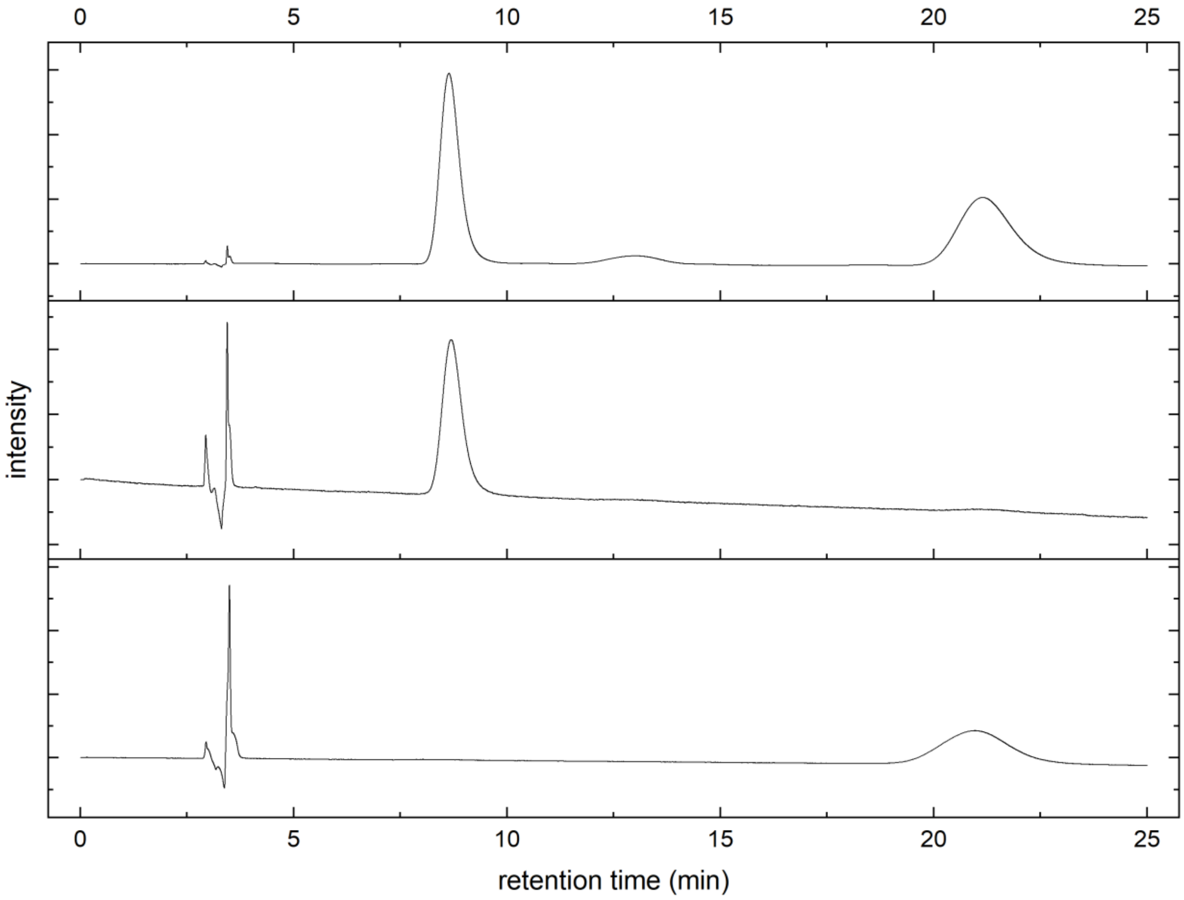

Figure S85: Chiral resolution of ***(rac)*-PoGe[H, H]** (top); injection of separated sample ***(P)*-PoGe[H, H]** (middle), injection of separated sample ***(M)*-PoGe[H, H]** (bottom). Conditions: 50% EtOAc in heptane, Chiralpak IG 4.6x250mm, 1.0 mL/min.

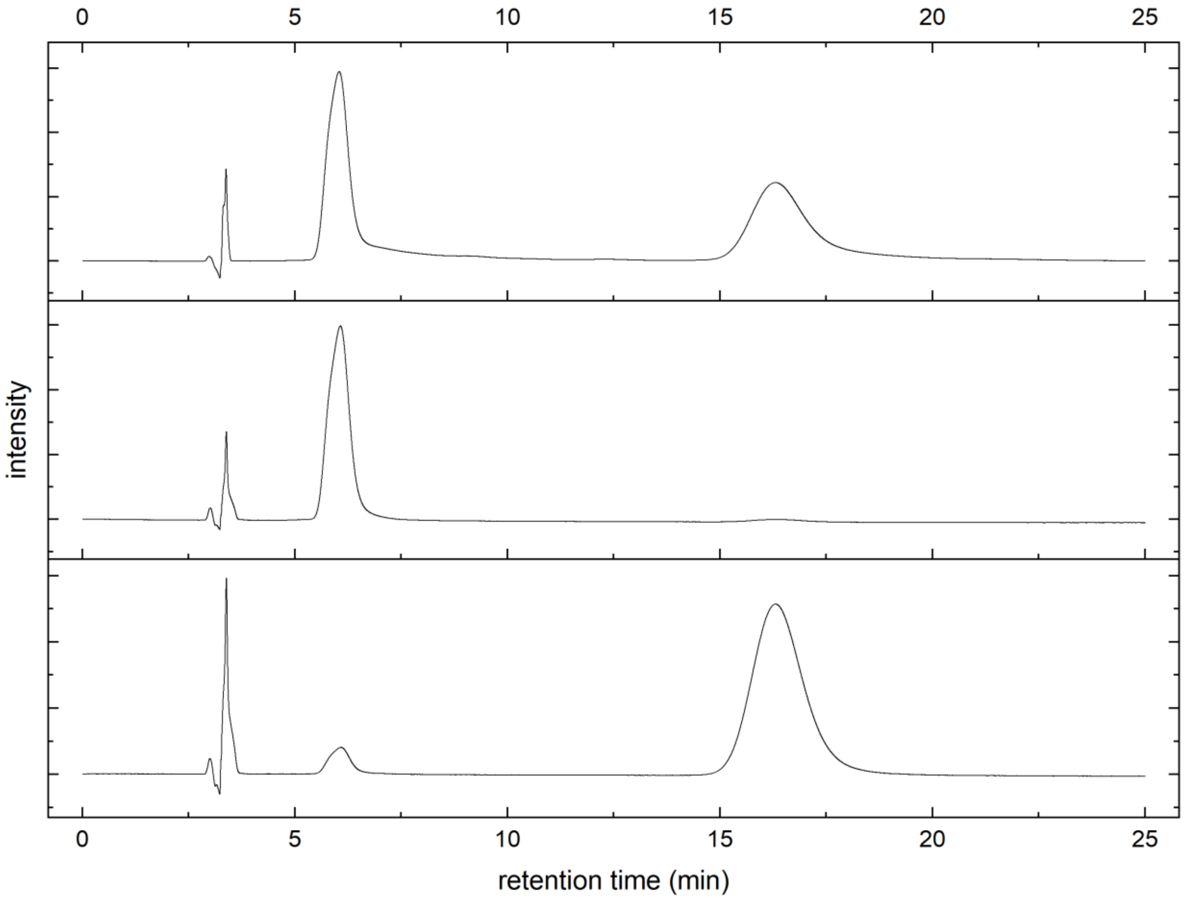

Figure S86: Chiral resolution of ***(rac)*-PoGe[Cu, Cu]** (top); injection of separated sample ***(P)*-PoGe[Cu, Cu]** (middle), injection of separated sample ***(M)*-PoGe[Cu, Cu]** (bottom).. Conditions: 70% EtOAc in heptane, Chiralpak IG 4.6x250mm, 1.0 mL/min.

ECD Spectroscopy

Dissymmetry Plot

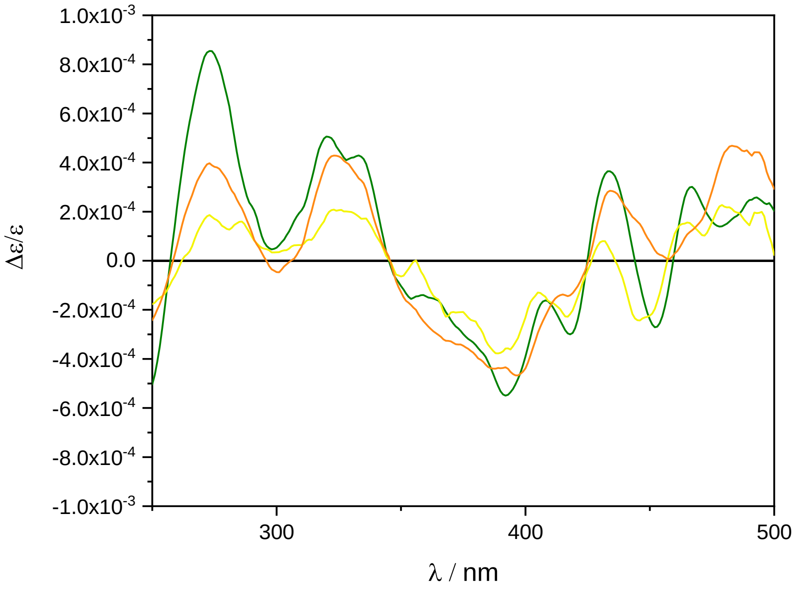

Figure S87: : g_{abs}-plot of ***(P)*-PoGe[Zn, Zn]** (green), ***(P)*-PoGe[Cu, Cu]** (yellow), and ***(P)*-PoGe[2H, 2H]** (orange), all in chloroform.

Computational Details and Geometric Analysis

Geometry Optimization

Optimization, frequency analysis and TD-DFT calculations were performed using Gaussian 09 (E.01),[4] the geometries and subsequent frequency analysis were calculated at the B3LYP/def2SV(p) level of theory. ***(P)*-PoGe[Cu, Cu]** could theoretically assume an open shell singlet or open shell triplet configuration, treating it as either resulted in similar computed energy and was treated, for convenience, to assume a singlet state during later calculations. The initial guess for the geometry of ***(P)*-PoGe[Zn, Zn]** was obtained from molecular mechanics modeling. The initial guesses of ***(P)*-PoGe[Cu, Cu]** and ***(P)*-PoGe[2H, 2H]** were derived from the optimized structure of ***(P)*-PoGe[Zn, Zn]**. Based on our experience with rigid and chiral architectures, the geometries obtained were used for TD-DFT calculations without further conformer analysis.

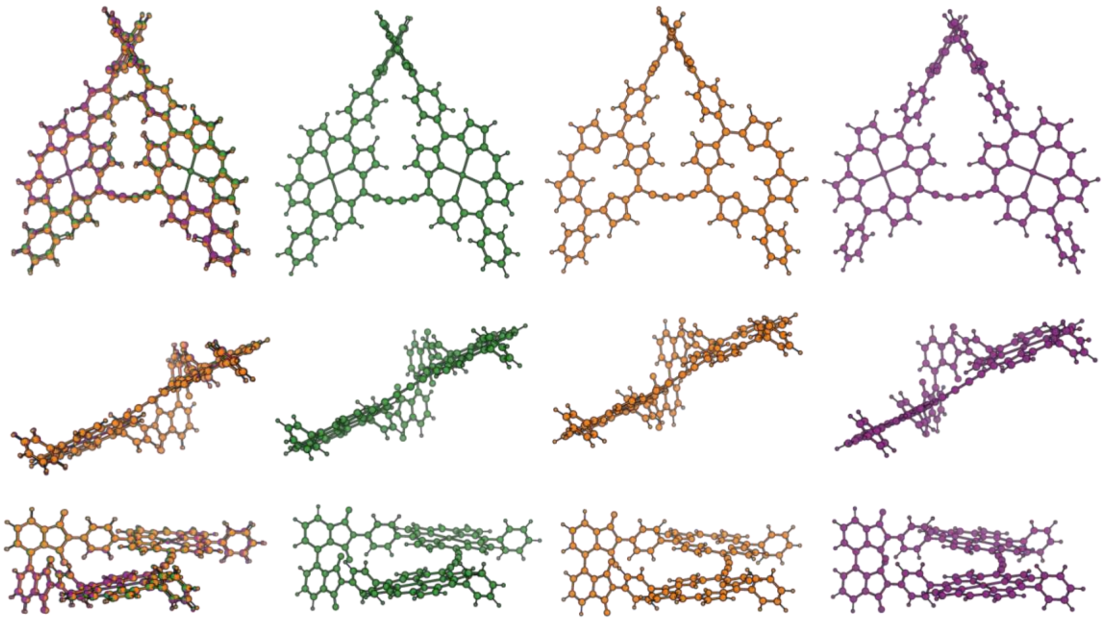

Figure S88: *Visualization of optimized structures of* ***(P)-PoGe[Zn, Zn]*** *(green),* ***(P)-PoGe[2H, 2H]*** *(orange), and* ***(P)-PoGe[Cu, Cu]*** *(purple) at B3LYP/def2SV(p) level of theory. In the first row all structures are illustrated overlapped. Top to bottom: Top, front and side view.*

TD-DFT

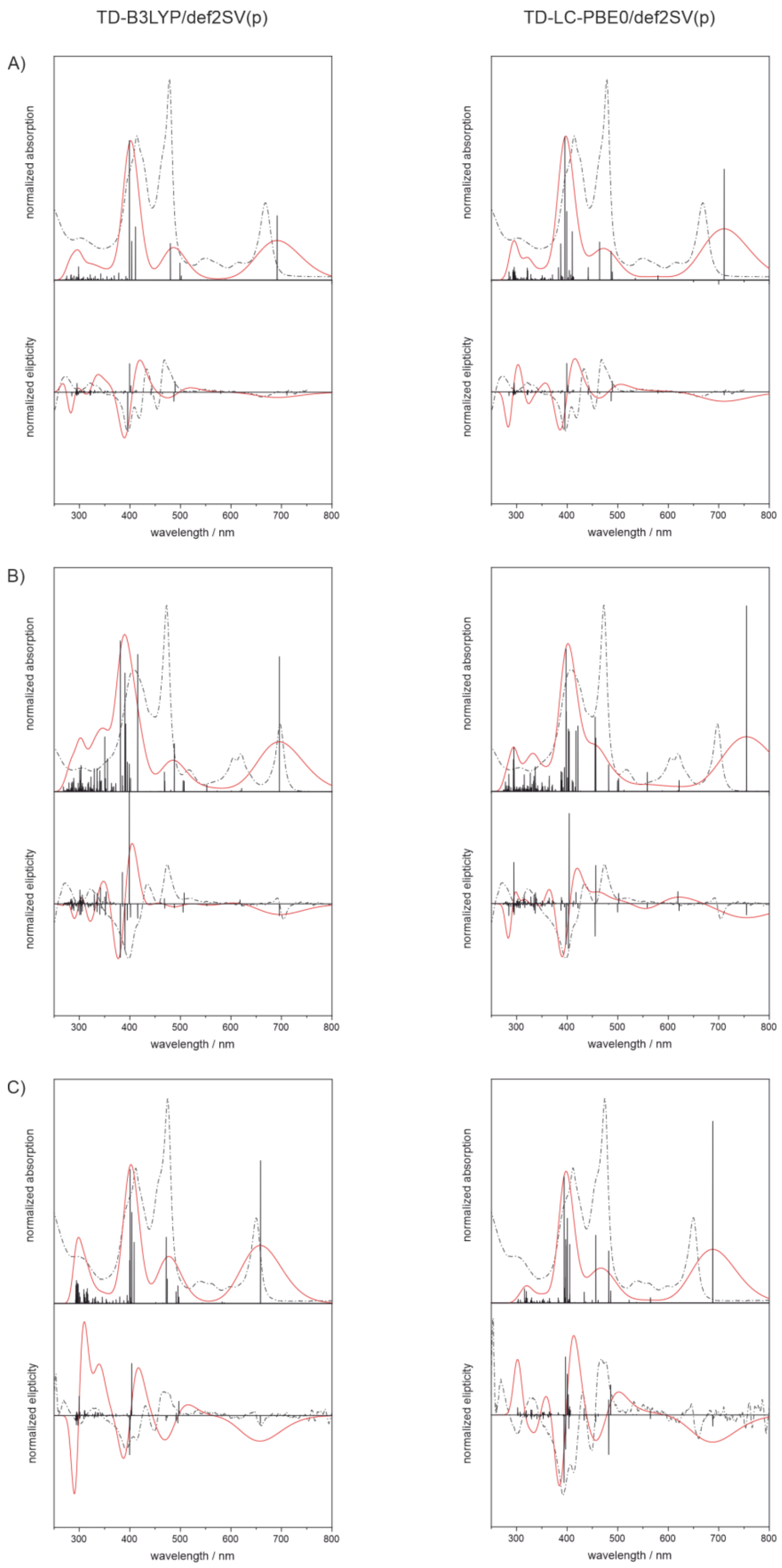

Figure S89: Simulated absorption and ECD spectra of A) ***(P)*-Poge[Zn, Zn],** B) ***(P)*-Poge[2H, 2H],** and C) ***(P)*-Poge[Cu, Cu]**. 200 transitions were computed at the TD-B3LYP/def2SV(p) (left) and TD-LC-PBE0/def2SV(p) (right) level of theory.

For the simulation of ECD and absorption spectra, the workflow of *Pescitelli* and *Bruhn* was followed.[5] All simulated spectra were obtained by calculation of 200 vertical transitions at the TD-B3LYP/def2SV(p) and TD-LC-PBE0[6,7]/def2SV(p) (obtained by a value of 0.30 as range separation parameter) level of theory with the PCM model for chloroform. SpecDis 1.71 was used to extract the transitions and fit the calculated data.[8] In no calculation, it was necessary to shift the obtained transitions. All spectra were fitted with gaussians and σ = 0.16 eV.

TDM Vector Analysis

NTOs and TDM vector decomposition were analyzed using Multiwfn 3.7[9]. Vectors were visualized using VMD 1.9.4.[10]. For all analyses, the computed transitions of ***(P)*-PoGe[Zn, Zn]** at TD-B3LYP/def2SV(p) level of theory were used. For discussion of the absorption spectra, natural transition orbitals (NTOs, *Figure S90*) and polarization of the first 50 computed transitions with oscillator strength f ≥ 0.1 were considered. The NTO analysis shows that the transitions are mostly located on the banister as concluded from the experimental data.

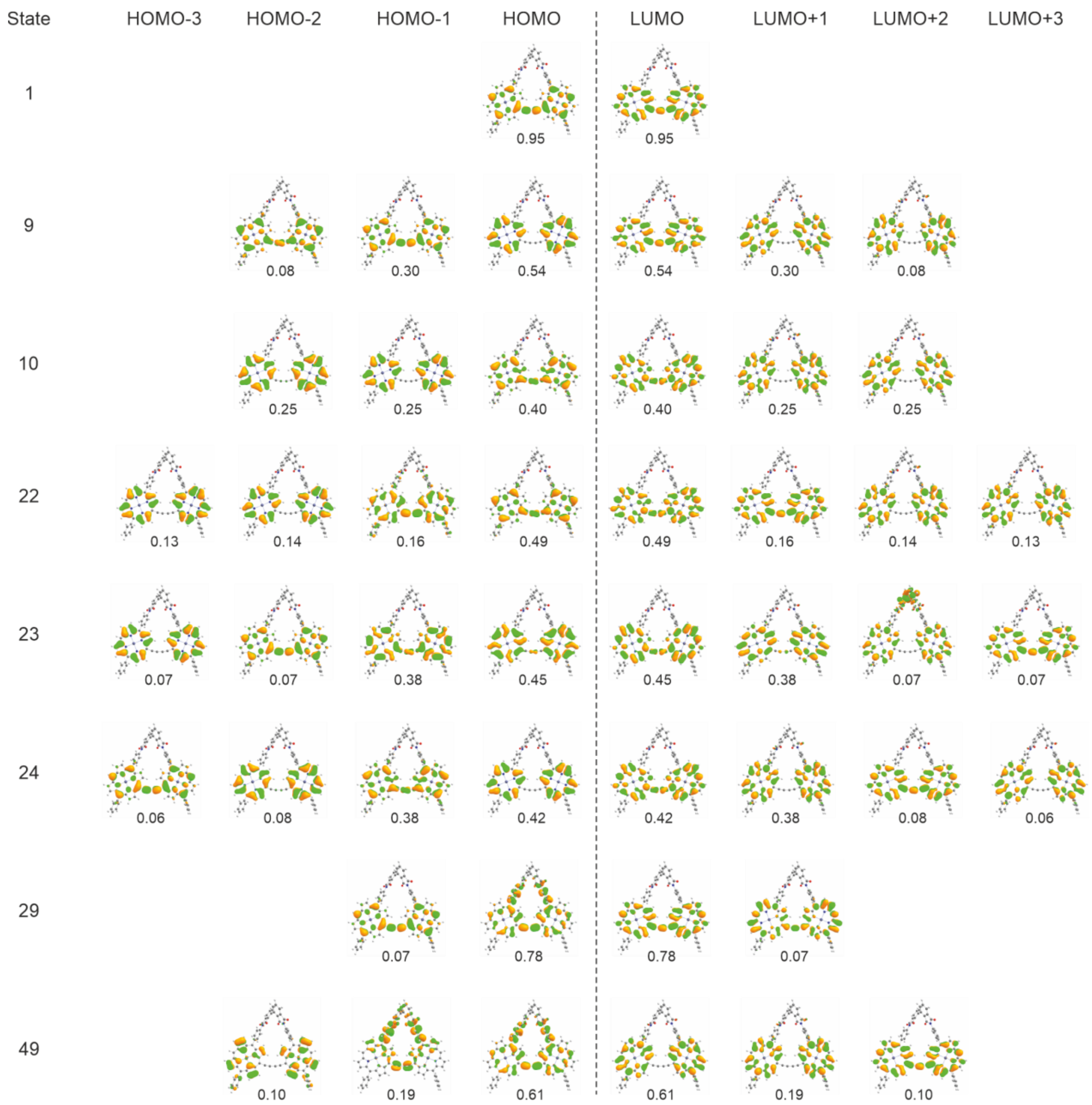

Figure S90: NTOs of selected transitions with oscillator strength above 0.1 and their occupancy. Only orbitals with occupancy above 0.05 are shown.

Table S1: States discussed for transition polarization analysis.

Transition/State	Energy / eV	Wavelength / nm	Oscillator Strength / a.u
1	1.7927	691.61	1.1969
9	2.4834	499.26	0.3156
10	2.5812	480.34	0.6812
22	3.0151	411.21	0.9894
23	3.0722	403.56	0.7221
24	3.1065	399.11	2.5985
29	3.2795	378.06	0.1328
49	3.6217	342.34	0.1184

Figure S91: Calculated wavelength vs. oscillator strength. Polarization along the banister are plotted as bars above the x-axis, polarizations along the rung below the axis.

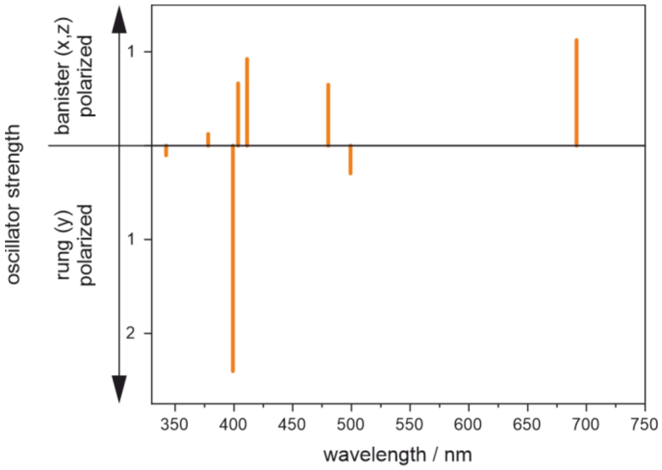

The polarization of the considered transitions is in solid agreement with a study by *Peeks et al., which* investigated the rotational barrier of butadiyne-linked porphyrins.[11] This allows us to conclude that the entire region has distinct areas influenced by TDMs along B_x and B_y.

In the simulated ECD spectrum of ***(P)*-PoGe[Zn, Zn]**, four transitions can be identified with comparably large rotary strength (*Table S2*), which dominate the prediction of the bisignate signals in the B-band. Their computed TDMs were decomposed to their contributions from every single atom. State 1 (within the Q-region) was also analyzed for completeness. ***(P)*-PoGe[Zn, Zn]** was divided into 9 parts: the porphyrin subunits, the phthalimide subunits, the rungs, the phenylenes in the periphery (each as two parts), and the butadiyne linker. The overall TDM vector for any discussed state and its vector decomposition by each described subunit was visualized. All transitions are dominated by TDM vectors located on the porphyrins. The other subunits only show little contribution. The pair of states 8 and 9 and the pair 23 and 24 closely represent the expected directions $B_{y,}$ as discussed in the main text of this paper.

Table S2: States discussed for vector analysis of relevant ECD transitions.

Transition/State	Energy / eV	Wavelength / nm	Oscillator Strength / a.u	Rotary Strength / a.u
1	1.7927	691.61	1.1969	-69.5274
8	2.4713	501.69	0.0770	322.1299
9	2.4834	499.26	0.3156	-263.4614
23	3.0722	403.56	0.7221	1506.6949
24	3.1065	399.11	2.5985	-1617.0478

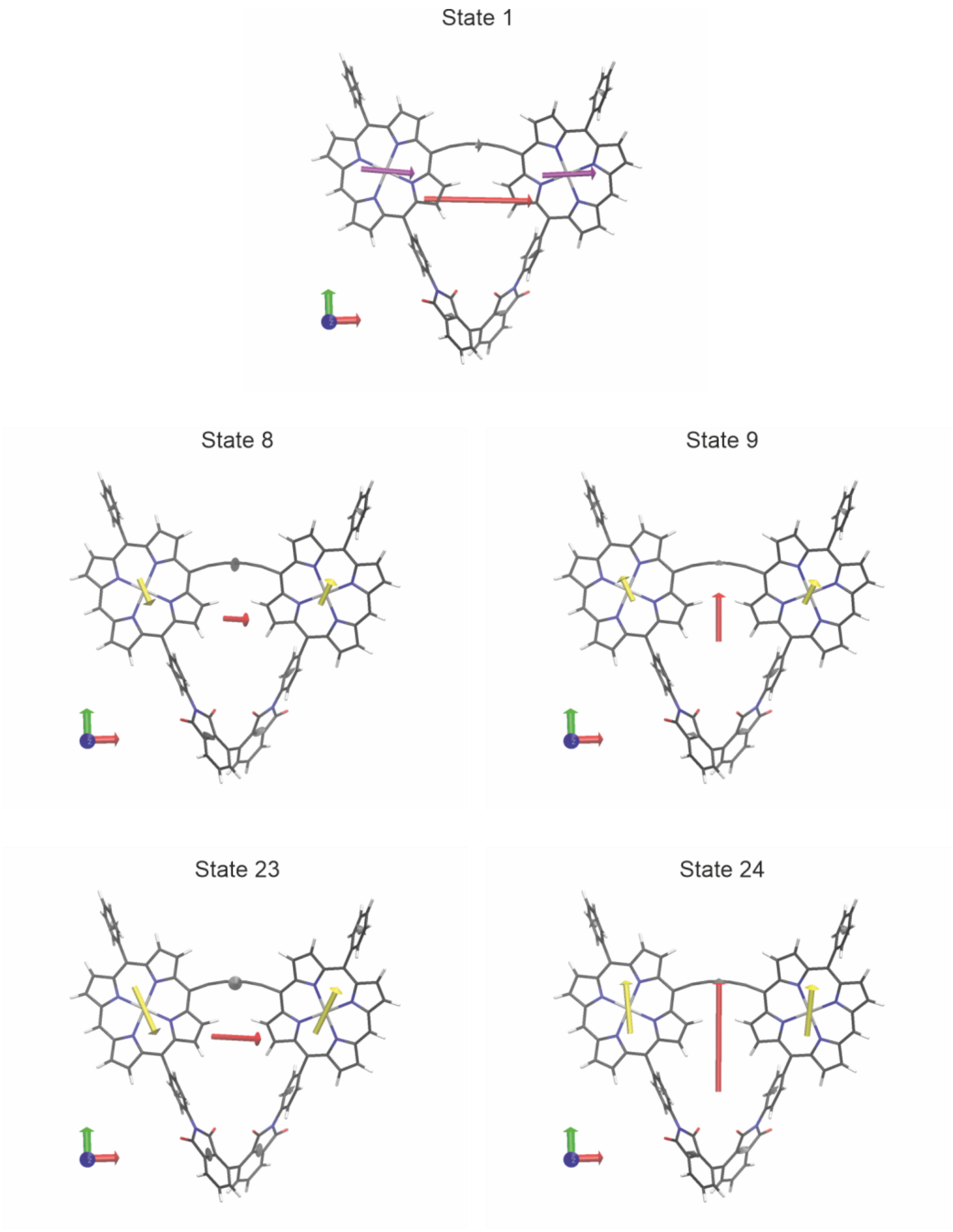

Figure S92: Vector analysis of the discussed transitions: the overall transition dipole moment (TDM) is visualized as a red arrow. Vectors on the porphyrin are either yellow (if polarized mostly along the y-axis) or purple (if mainly polarized along the x-axis). All other vectors are visualized in grey. Coordinate system: X (red), Y (green), Z (blue).

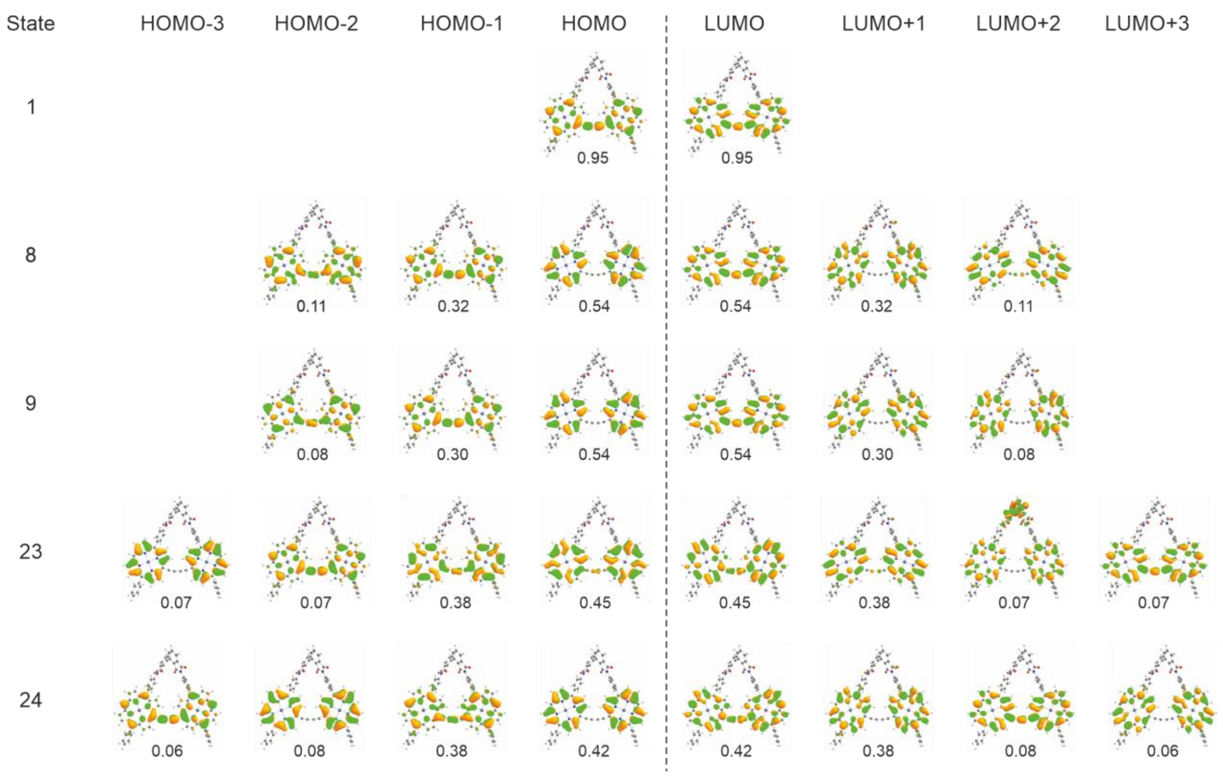

Figure S93: NTOs of selected transitions with intense rotary strength. Only orbitals with occupancy above 0.05 are shown.

Determination of Torsions

Mercury 2021.3.0 was used to measure the angles in the modeled structures of ***(P)*-PoGe[Zn, Zn]**, ***(P)*-PoGe[Cu, Cu]**, and ***(P)*-PoGe[2H, 2H]**. Four centroids (dummy atoms) were calculated within each porphyrin and phthalimide subunit acting as anchoring point of the plane they spanned. For ***(P)*-PoGe[Zn, Zn]** and ***(P)*-PoGe[Cu, Cu]** the centroids were congruent with the coordinated metals. The torsions were measured anchored by the centroids over the inner CC bond of the butadiyne linker or the CC bond connecting the phthalimides, respectively.

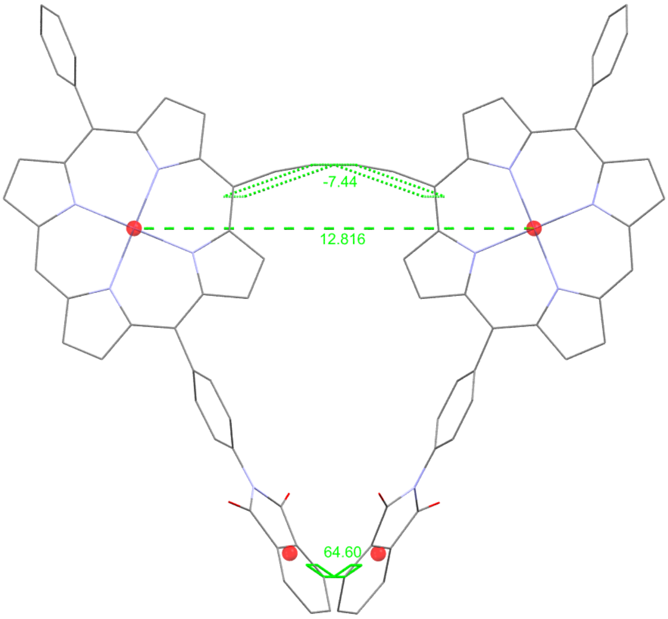

Figure S94: Wireframe model of ***(P)*-PoGe[Zn, Zn]** and the calculated centroids (red). Torsions around axis and banister and the inter porphyrin distance are indicated.

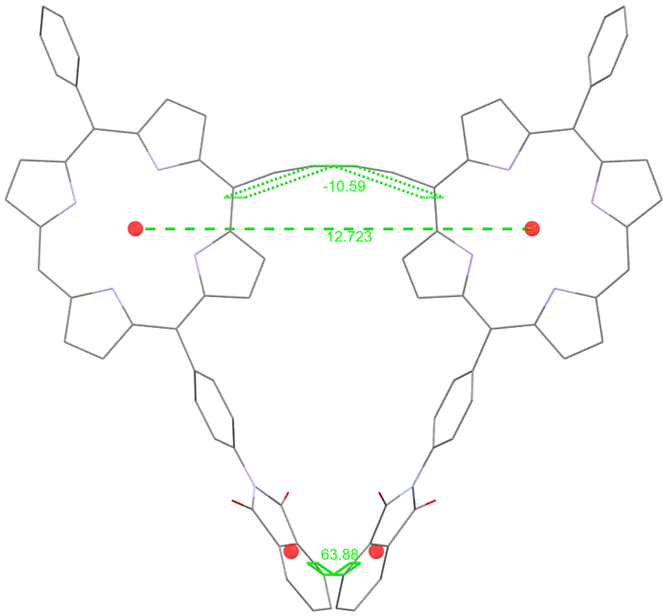

Figure S95: Wireframe model of ***(P)*-PoGe[2H, 2H]** and the calculated centroids (red). Torsions around axis and banister and the inter porphyrin distance are indicated.

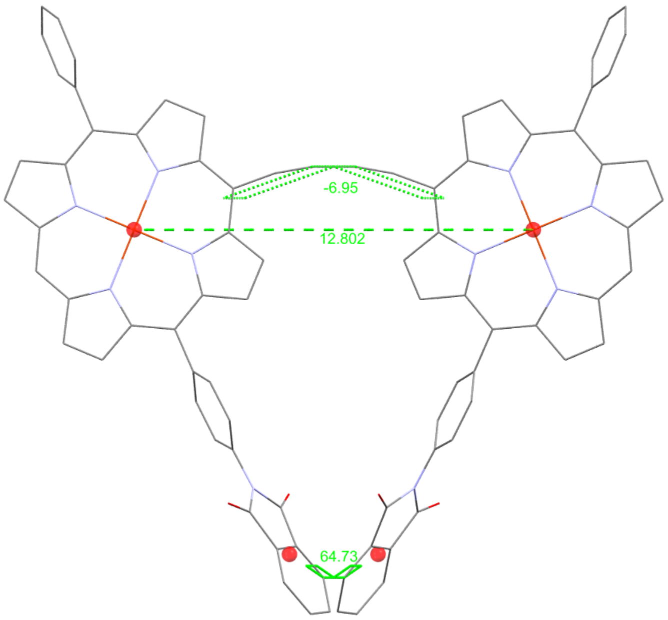

Figure S96: Wireframe model of ***(P)*-PoGe[Zn, Zn]** and the calculated centroids (red). Torsions around axis and banister and the inter porphyrin distance are indicated

Fluorescence Lifetime Measurements

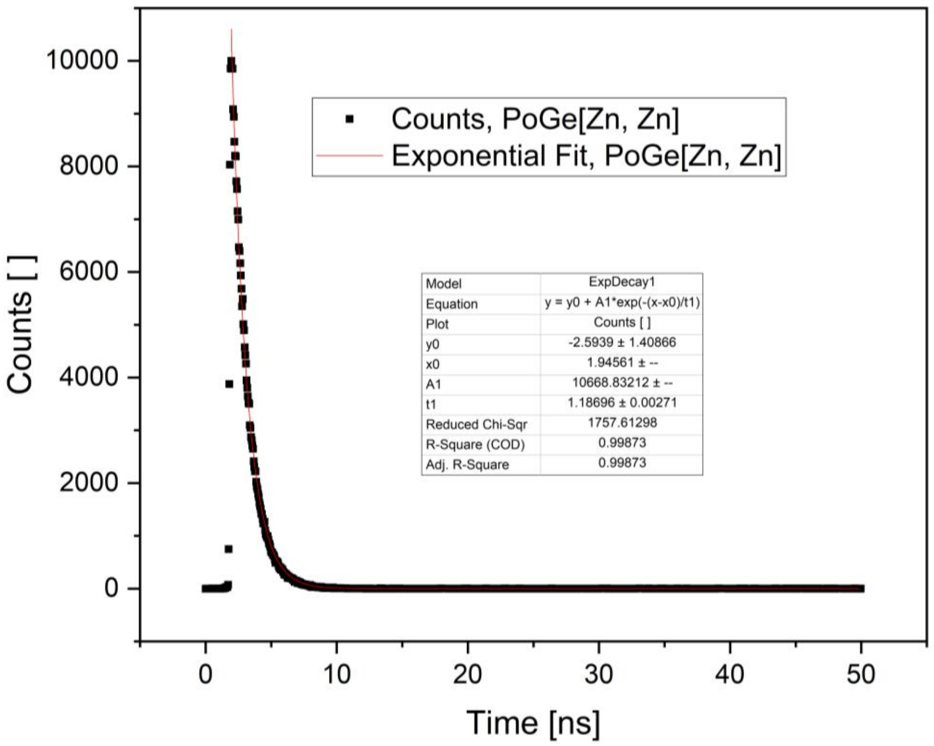

Figure S97: Experimental fluorescence lifetime data (black squares) and exponential decay fit (red line) for ***(rac)*-PoGe[Zn, Zn]** ($CHCl_3$, OD = 0.23, λ(ex) = 400 nm, λ(em) = 700 nm, 10^4 counts).

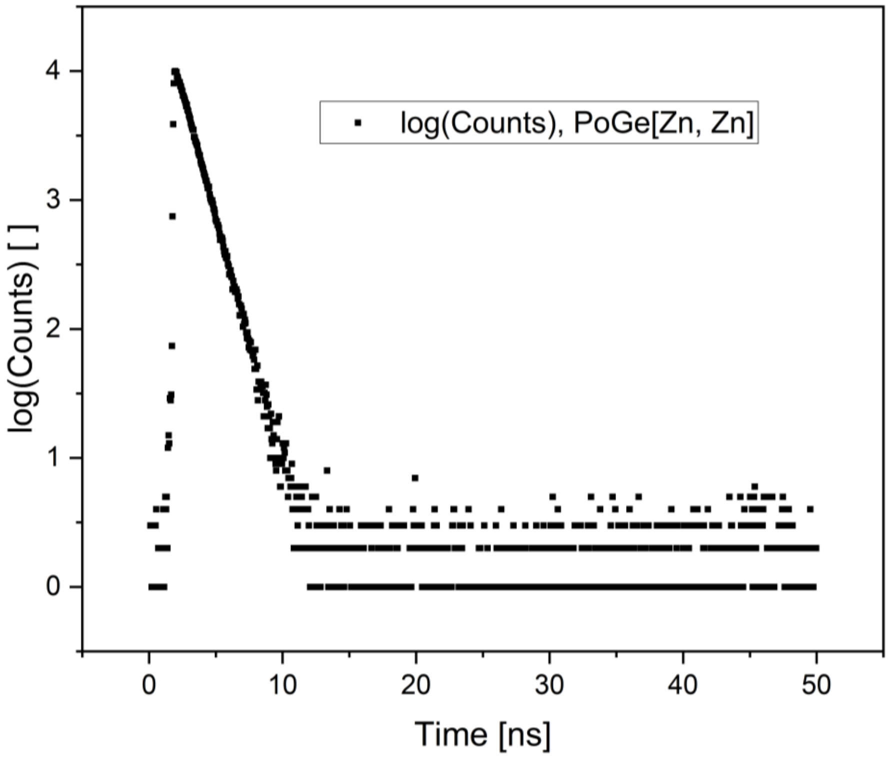

Figure S98: Logarithmic representation of experimental fluorescence lifetime data (black squares) for ***(rac)*-PoGe[Zn, Zn]** ($CHCl_3$, OD = 0.23, λ(ex) = 400 nm, λ(em) = 700 nm, 10^4 counts).

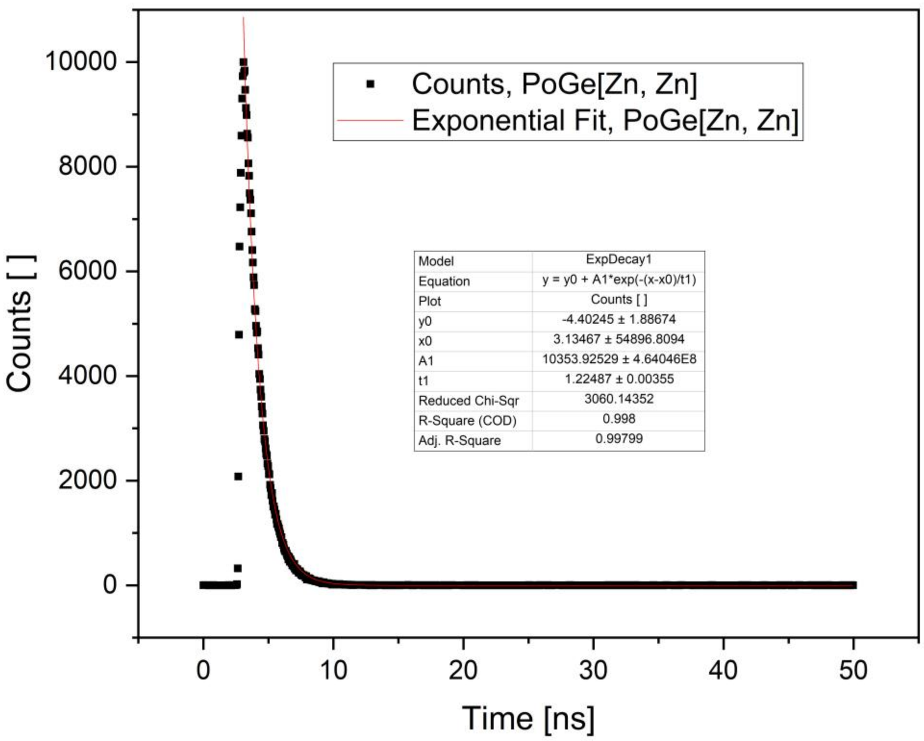

Figure S99: Experimental fluorescence lifetime data (black squares) and exponential decay fit (red line) for ***(rac)*-PoGe[Zn, Zn]** ($CHCl_3$, OD = 0.23, λ(ex) = 635 nm, λ(em) = 700 nm, 10^4 counts).

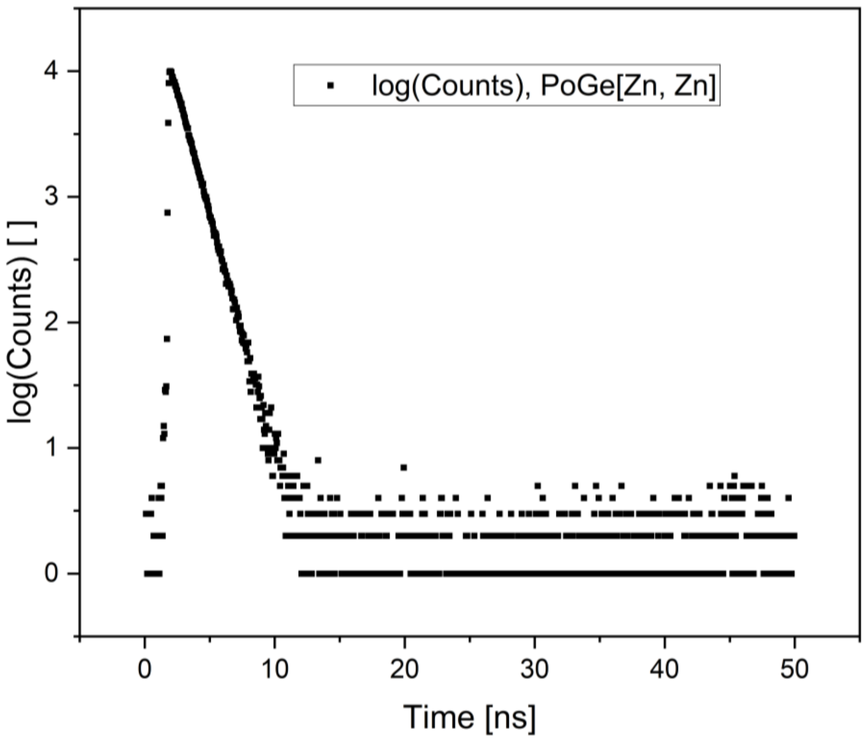

Figure S100: Logarithmic representation of experimental fluorescence lifetime data (black squares) for ***(rac)*-PoGe[Zn, Zn]** ($CHCl_3$, OD = 0.23, λ(ex) = 635 nm, λ(em) = 700 nm, 10^4 counts).

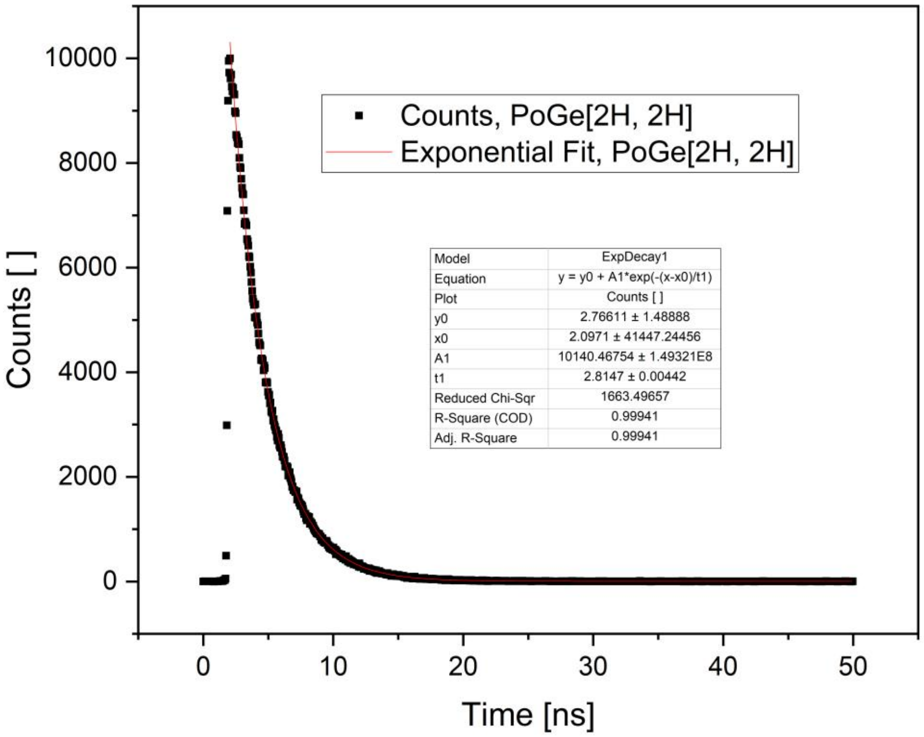

Figure S101: Experimental fluorescence lifetime data (black squares) and exponential decay fit (red line) for ***(rac)*-PoGe[2H, 2H]** ($CHCl_3$, OD = 0.15, λ(ex) = 400 nm, λ(em) = 700 nm, 10^4 counts).

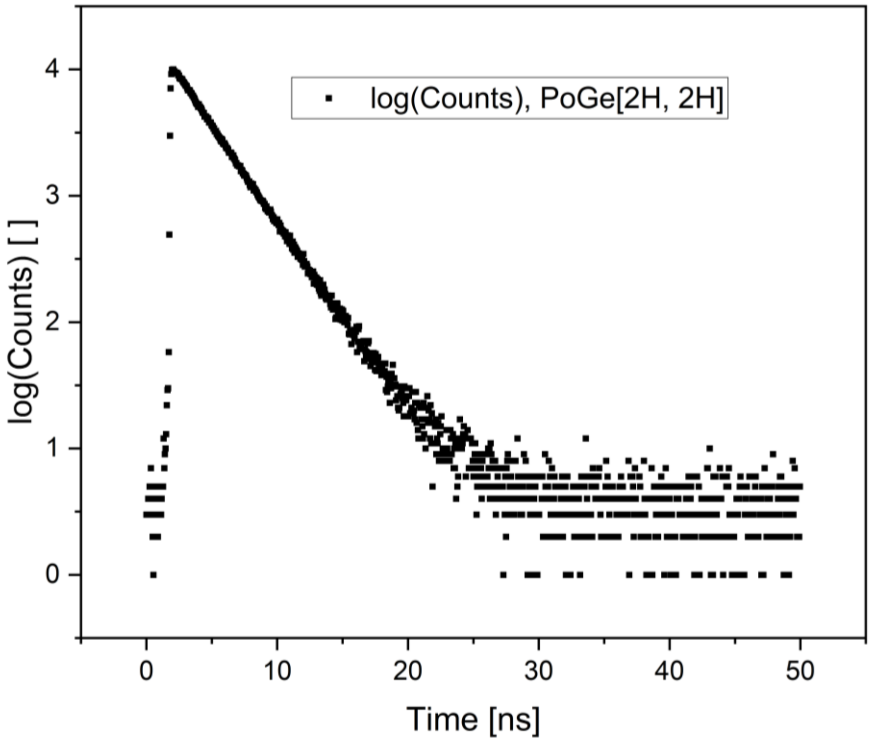

Figure S102: Logarithmic representation of experimental fluorescence lifetime data (black squares) for ***(rac)*-PoGe[2H, 2H]** ($CHCl_3$, OD = 0.15, λ(ex) = 400 nm, λ(em) = 700 nm, 10^4 counts).

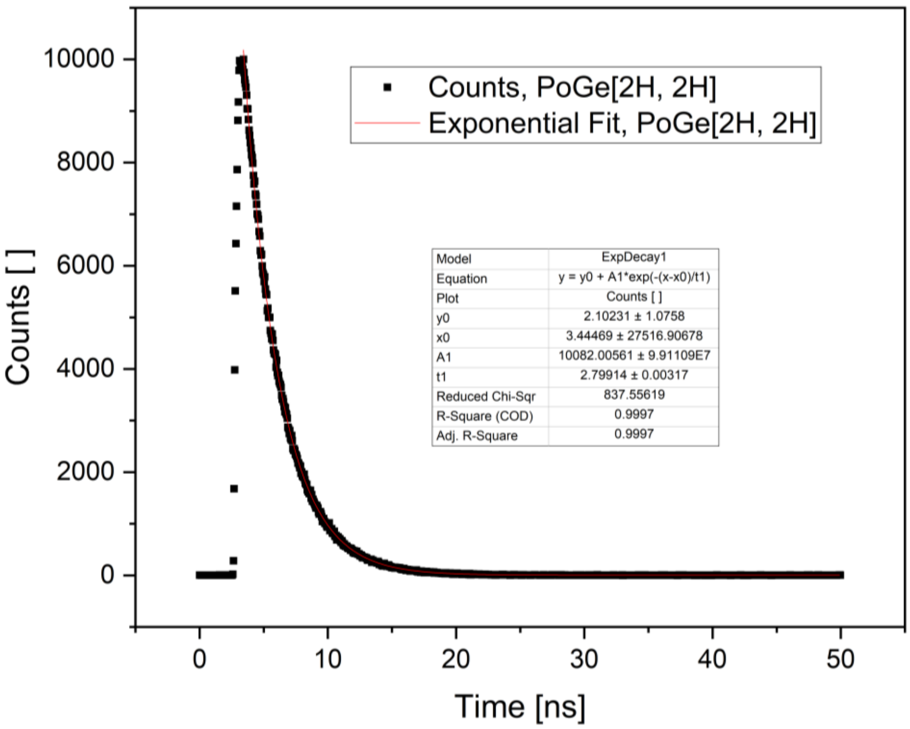

Figure S103: Experimental fluorescence lifetime data (black squares) and exponential decay fit (red line) for ***(rac)*-PoGe[2H, 2H]** ($CHCl_3$, OD = 0.15, λ(ex) = 635 nm, λ(em) = 700 nm, 10^4 counts).

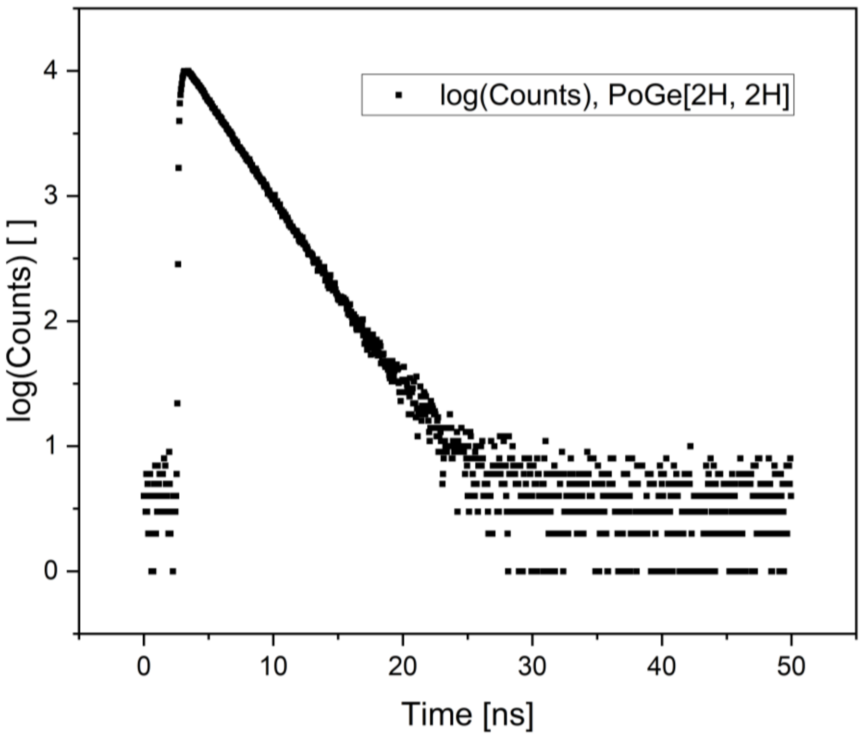

Figure S104: Logarithmic representation of experimental fluorescence lifetime data (black squares) for ***(rac)*-PoGe[2H, 2H]** ($CHCl_3$, OD = 0.15, λ(ex) = 635 nm, λ(em) = 700 nm, 10^4 counts).

Cyclic Voltammetry

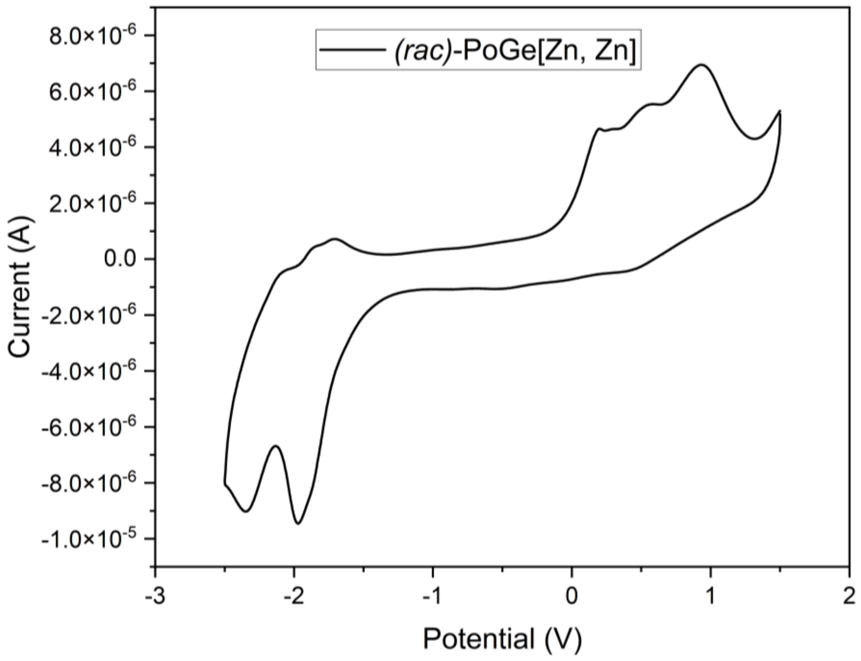

Figure S105: Cyclic voltammogram of ***(rac)*-PoGe[Zn, Zn]** in CH_2Cl_2 with 0.1 M $TBAPF_6$ electrolyte, measured in a one-compartment cell, a Pt-working electrode, a Pt auxiliary electrode and a silver wire dipped in the solution as a reference electrode, referenced against Fc/Fc+ couple measured at +0.49 V in a separate experiment.

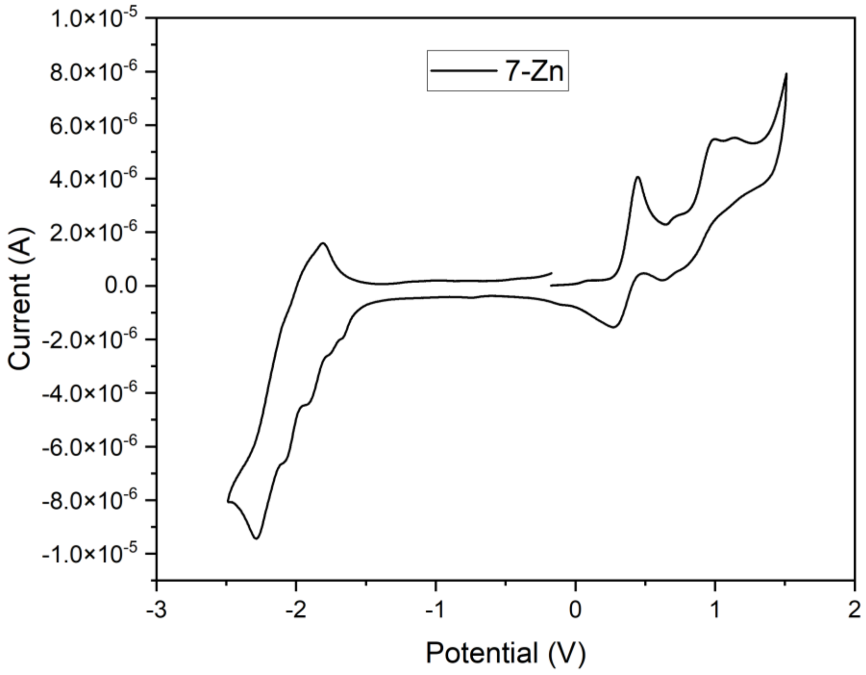

Figure S106: Cyclic voltammogram of **7-Zn** in CH_2Cl_2 with 0.1 M $TBAPF_6$ electrolyte, measured in a one-compartment cell, a Pt-working electrode, a Pt auxiliary electrode and a silver wire dipped in the solution as a reference electrode, referenced against Fc/Fc+ couple measured at +0.50 V in a separate experiment.

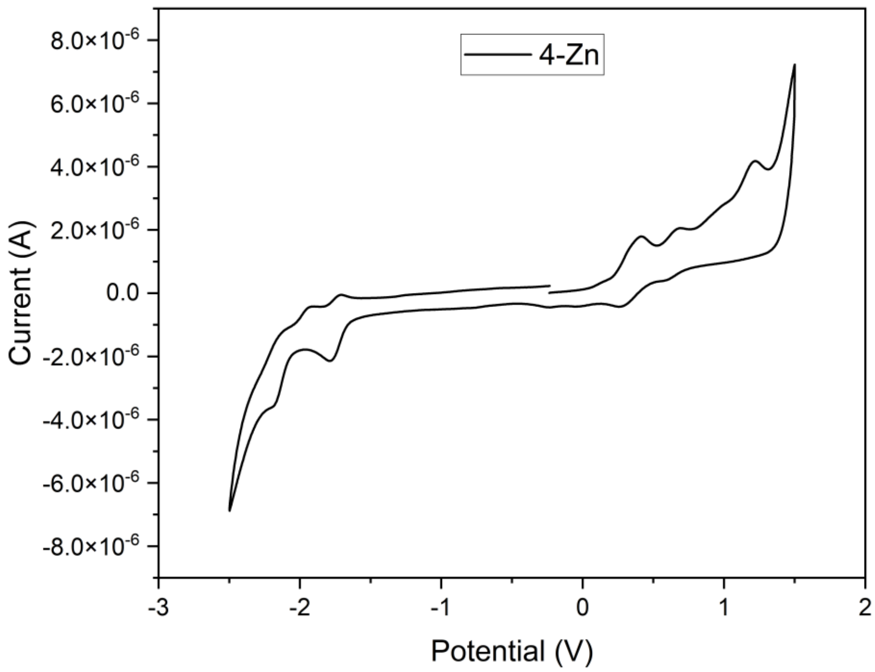

Figure S107: Cyclic voltammogram of **4-Zn** in CH_2Cl_2 with 0.1 M $TBAPF_6$ electrolyte, measured in a one-compartment cell, a Pt-working electrode, a Pt auxiliary electrode and a silver wire dipped in the solution as a reference electrode, referenced against Fc/Fc+ couple measured at +0.50 V in a separate experiment.

Table S3: Electrochemical data obtained by cyclic voltammetry and extracted from Figure S105 – S107 for **4-Zn**, **7-Zn** and ***(rac)*-PoGe[Zn, Zn]**. a) Reported is $E_{pc,ox}$ and $E_{pa,red}$ of irreversible redox processes at the peak maximum, b) reported is $E_{1/2,ox}$ and $E_{1/2,red}$, the half-wave potentials of reversible or quasi-reversible redox processes, c) ill-defined reduction wave. given is only the maximum of the reduction wave. All potentials are reported vs Fc/Fc^+. Measurements were performed in dry, deoxygenated CH_2Cl_2 under an argon atmosphere at 25 °C, using 0.10 M tetrabutylammonium hexafluorophosphate ($TBAPF_6$) as supporting electrolyte. Cyclic voltammograms were recorded at a scan rate of 100 mV s^{-1} using a single-compartment cell equipped with a platinum working electrode, a platinum counter electrode, and a silver wire reference electrode.

Compound	E Red, 2 [V]	E Red, 1 [V]	E Ox, 1 [V]	E Ox, 2 [V]	E Ox, 3 [V]	E Ox, 4 [V]
4-Zn	-2.19[a]	-1,75	+0.35[b]	+0.65[b]	+1.22[a]	-
7-Zn	-	-2.29[c] (max)	+0.34[b]	+0.72[a]	+0.99[a]	+1.14[a]
(rac)-PoGe[Zn, Zn]	-2.35[a]	-1.97[a]	+0.20[a]	+0.55[a]	+0.93[a]	-

.

Cartesians

Energies are reported in kcal/mol.

***(P)*-PoGe[Zn, Zn]**
142
Energy: -4788001.2767952

C	-5.09325	1.17182	-0.51557
C	-3.22296	-3.34818	-0.06209
C	-7.72907	-5.20237	0.45905
C	-9.55797	-0.68983	0.09242
C	-8.51116	0.21947	-0.09509
C	-8.67966	1.64333	-0.27872
C	-7.43027	2.16888	-0.45552
C	-6.49415	1.06439	-0.37098
N	-7.18071	-0.10215	-0.15294
C	-4.19452	0.08746	-0.45524
C	-2.75503	0.21300	-0.55898
C	-2.23550	-1.04194	-0.43679
C	-3.35166	-1.94117	-0.24968
N	-4.52234	-1.23827	-0.26708
C	-4.30051	-4.25385	0.11743
C	-4.14210	-5.67609	0.31474
C	-5.39726	-6.19433	0.45742
C	-6.32833	-5.08770	0.34529
N	-5.62456	-3.92138	0.14044
C	-8.63451	-4.11742	0.40616
C	-10.07842	-4.23346	0.49344
C	-10.58543	-2.96817	0.39743
C	-9.45855	-2.07721	0.24217
N	-8.29270	-2.79861	0.25003
C	-4.49887	2.52809	-0.75350
C	-4.42987	3.48869	0.26904
C	-3.80941	4.72339	0.06376
C	-3.23324	5.02011	-1.17990
C	-3.31006	4.07866	-2.21710
C	-3.94088	2.85371	-2.00142
N	-2.56712	6.26169	-1.39557
C	-1.89065	-3.83767	-0.03360
C	-0.68010	-4.05115	-0.01476
C	0.68010	-4.05115	0.01477
C	1.89065	-3.83767	0.03361
C	5.09325	1.17182	0.51557
C	9.55797	-0.68983	-0.09244
C	7.72907	-5.20237	-0.45905
C	3.22297	-3.34817	0.06210
C	3.35166	-1.94117	0.24968
C	2.23550	-1.04193	0.43678
C	2.75503	0.21301	0.55896
C	4.19452	0.08746	0.45523
N	4.52234	-1.23826	0.26707
C	6.49415	1.06440	0.37097
C	7.43027	2.16888	0.45553
C	8.67966	1.64333	0.27873
C	8.51116	0.21948	0.09508
N	7.18071	-0.10214	0.15294
C	9.45855	-2.07721	-0.24219
C	10.58543	-2.96816	-0.39746
C	10.07842	-4.23345	-0.49346
C	8.63451	-4.11741	-0.40617
N	8.29270	-2.79860	-0.25005
C	6.32834	-5.08770	-0.34527
C	5.39727	-6.19433	-0.45739
C	4.14211	-5.67609	-0.31472
C	4.30051	-4.25385	-0.11742
N	5.62456	-3.92138	-0.14044
C	4.49887	2.52809	0.75349
C	4.42986	3.48869	-0.26904
C	3.80940	4.72339	-0.06376
C	3.23324	5.02011	1.17991
C	3.31005	4.07866	2.21710
C	3.94088	2.85371	2.00142
N	2.56712	6.26169	1.39557
C	1.69525	6.88015	0.46323
C	1.19130	8.12365	1.13088
C	1.79317	8.21000	2.38711
C	2.66875	7.01954	2.58650
C	-2.66876	7.01954	-2.58649
C	-1.79318	8.21000	-2.38710
C	-1.19130	8.12365	-1.13087
C	-1.69526	6.88015	-0.46322
O	-1.44982	6.46684	0.64365
O	-3.33842	6.74396	-3.55145
C	-1.57213	9.27517	-3.25159
C	-0.71362	10.28822	-2.80641
C	-0.11221	10.21302	-1.54357
C	-0.32633	9.12523	-0.66998
C	0.32632	9.12523	0.66999
C	0.11220	10.21302	1.54358
C	0.71361	10.28822	2.80642
C	1.57212	9.27516	3.25160
O	3.33842	6.74395	3.55145
O	1.44982	6.46685	-0.64365
C	-8.30170	-6.57484	0.65397
C	-8.86683	-6.95117	1.88522
C	-9.40008	-8.23095	2.06703
C	-9.38009	-9.15666	1.01865
C	-8.82190	-8.79433	-0.21157
C	-8.28638	-7.51518	-0.39122
C	8.30171	-6.57483	-0.65396
C	8.28640	-7.51516	0.39123
C	8.82192	-8.79432	0.21159
C	9.38010	-9.15665	-1.01863
C	9.40008	-8.23095	-2.06702
C	8.86682	-6.95118	-1.88522
H	-10.56806	-0.27101	0.11140
H	-9.63608	2.16723	-0.28118
H	-7.16179	3.20817	-0.63587
H	-2.20935	1.14332	-0.69858
H	-1.18915	-1.33512	-0.47107
H	-3.18813	-6.20047	0.34552
H	-5.67132	-7.23222	0.63443
H	-10.62899	-5.16521	0.60559
H	-11.63105	-2.65932	0.42025
H	-4.85296	3.26017	1.25150
H	-3.75274	5.44557	0.87867
H	-2.87859	4.30293	-3.19282
H	-3.98751	2.12619	-2.81663
H	10.56806	-0.27100	-0.11142
H	1.18915	-1.33512	0.47106
H	2.20935	1.14332	0.69856
H	7.16179	3.20817	0.63587
H	9.63608	2.16723	0.28118
H	11.63105	-2.65931	-0.42028
H	10.62899	-5.16521	-0.60561
H	5.67133	-7.23222	-0.63439
H	3.18814	-6.20047	-0.34548
H	4.85296	3.26018	-1.25150
H	3.75274	5.44558	-0.87867
H	2.87859	4.30293	3.19282
H	3.98751	2.12619	2.81662
H	-2.05551	9.31067	-4.23123
H	-0.50405	11.14953	-3.44767

H	0.56114	11.01461	-1.22613
H	-0.56115	11.01461	1.22615
H	0.50404	11.14952	3.44768
H	2.05550	9.31066	4.23124
H	-8.87959	-6.23243	2.71000
H	-9.83024	-8.50668	3.03512
H	-9.79809	-10.15825	1.16023
H	-8.80472	-9.51050	-1.03923
H	-7.85475	-7.23471	-1.35662
H	7.85478	-7.23469	1.35664
H	8.80475	-9.51048	1.03926
H	9.79810	-10.15825	-1.16021
H	9.83022	-8.50669	-3.03511
H	8.87957	-6.23244	-2.71000
Zn	6.40819	-2.01441	0.00665
Zn	-6.40819	-2.01441	-0.00667

***(P)*-PoGe[2H, 2H]**

144

Energy: -2556458.4889625

C	-5.04983	1.03877	-0.56874
C	-3.22229	-3.53176	-0.09169
C	-7.68686	-5.44171	0.47138
C	-9.46198	-0.87471	0.12470
C	-8.41032	0.02927	-0.07939
C	-8.62880	1.46145	-0.26588
C	-7.40056	2.00914	-0.46667
C	-6.44699	0.90059	-0.39076
N	-7.08803	-0.27670	-0.15690
C	-4.14105	-0.03562	-0.52729
C	-2.71187	0.03662	-0.63969
C	-2.21270	-1.23498	-0.50888
C	-3.31638	-2.12852	-0.30411
C	-4.30806	-4.43221	0.10408
C	-4.10356	-5.86172	0.31881
C	-5.34130	-6.40057	0.46877
C	-6.28787	-5.29124	0.34402
N	-5.62326	-4.11351	0.12617
C	-8.60608	-4.37116	0.43437
C	-10.03998	-4.43264	0.51826
C	-10.52281	-3.14789	0.42979
C	-9.40407	-2.26239	0.27799
C	-4.46094	2.39473	-0.80809
C	-4.42991	3.36798	0.20508
C	-3.81207	4.60421	0.00373
C	-3.20098	4.89093	-1.22553
C	-3.24093	3.93806	-2.25410
C	-3.86822	2.71047	-2.04302
N	-2.53582	6.13387	-1.43375
C	-1.89077	-4.02406	-0.05173
C	-0.67980	-4.23489	-0.02203
C	0.67980	-4.23489	0.02199
C	1.89077	-4.02405	0.05169
C	5.04983	1.03878	0.56873
C	9.46199	-0.87470	-0.12462
C	7.68687	-5.44170	-0.47138
C	3.22229	-3.53176	0.09166
C	3.31637	-2.12851	0.30407
C	2.21269	-1.23497	0.50883
C	2.71186	0.03663	0.63964
C	4.14105	-0.03562	0.52727
C	6.44699	0.90060	0.39078
C	7.40056	2.00915	0.46672
C	8.62880	1.46145	0.26595
C	8.41032	0.02928	0.07945
N	7.08803	-0.27670	0.15693
C	9.40408	-2.26238	-0.27793
C	10.52282	-3.14788	-0.42972
C	10.03999	-4.43263	-0.51821
C	8.60609	-4.37115	-0.43434
C	6.28787	-5.29122	-0.34403
C	5.34131	-6.40055	-0.46880
C	4.10357	-5.86171	-0.31883
C	4.30806	-4.43220	-0.10410
N	5.62327	-4.11350	-0.12618
C	4.46093	2.39473	0.80808
C	4.42990	3.36798	-0.20509
C	3.81206	4.60421	-0.00374
C	3.20097	4.89093	1.22553
C	3.24092	3.93806	2.25409
C	3.86821	2.71047	2.04302
N	2.53581	6.13387	1.43374
C	1.68363	6.75734	0.48703
C	1.17046	7.99994	1.14902
C	1.74938	8.08141	2.41638
C	2.61616	6.88669	2.62959
C	-2.61617	6.88669	-2.62960
C	-1.74939	8.08141	-2.41639
C	-1.17047	7.99994	-1.14903
C	-1.68365	6.75734	-0.48704
O	-1.45843	6.34717	0.62533
O	-3.26448	6.60353	-3.60672
C	-1.51516	9.14540	-3.27892
C	-0.66793	10.16190	-2.82007
C	-0.08866	10.09078	-1.54661
C	-0.31549	9.00402	-0.67513
C	0.31548	9.00402	0.67512
C	0.08865	10.09078	1.54660
C	0.66791	10.16190	2.82006
C	1.51515	9.14540	3.27891
O	3.26447	6.60354	3.60671
O	1.45842	6.34717	-0.62533
C	-8.25155	-6.81637	0.65827
C	-8.87506	-7.18302	1.86456
C	-9.40296	-8.46625	2.03672
C	-9.31989	-9.40536	1.00355
C	-8.70385	-9.05295	-0.20178
C	-8.17382	-7.77057	-0.37219
C	8.25156	-6.81636	-0.65827
C	8.17384	-7.77056	0.37218
C	8.70387	-9.05293	0.20177
C	9.31991	-9.40534	-1.00357
C	9.40297	-8.46622	-2.03673
C	8.87507	-7.18299	-1.86457
H	-10.46870	-0.44794	0.15147
H	-9.59827	1.96154	-0.25355
H	-7.15240	3.05164	-0.65916
H	-2.14594	0.95244	-0.79122
H	-1.17105	-1.54174	-0.54901
H	-3.13765	-6.36391	0.35387
H	-5.59830	-7.44028	0.66134
H	-10.61449	-5.35008	0.62370
H	-11.56339	-2.82498	0.45384
H	-4.88036	3.14715	1.17695
H	-3.78464	5.33625	0.81144
H	-2.78397	4.15588	-3.21976
H	-3.88870	1.97524	-2.85235
H	10.46871	-0.44793	-0.15138
H	1.17104	-1.54173	0.54895
H	2.14594	0.95245	0.79116
H	7.15239	3.05165	0.65920
H	9.59827	1.96155	0.25364
H	11.56341	-2.82497	-0.45374
H	10.61450	-5.35007	-0.62365

H	5.59832	-7.44026	-0.66137
H	3.13766	-6.36390	-0.35391
H	4.88035	3.14715	-1.17695
H	3.78463	5.33625	-0.81144
H	2.78396	4.15588	3.21975
H	3.88870	1.97524	2.85234
H	-1.98010	9.17753	-4.26756
H	-0.44928	11.02252	-3.45921
H	0.57733	10.89447	-1.21911
H	-0.57735	10.89447	1.21910
H	0.44927	11.02252	3.45920
H	1.98009	9.17753	4.26755
H	-8.93379	-6.45561	2.67967
H	-9.87712	-8.73453	2.98614
H	-9.73337	-10.40982	1.13773
H	-8.63721	-9.77968	-1.01761
H	-7.69787	-7.49763	-1.31854
H	7.69789	-7.49762	1.31853
H	8.63723	-9.77967	1.01759
H	9.73339	-10.40979	-1.13775
H	9.87714	-8.73450	-2.98615
H	8.93380	-6.45557	-2.67968
N	-4.45296	-1.36520	-0.33229
H	-5.40782	-1.69913	-0.20557
N	-8.27720	-3.04581	0.29064
H	-7.31638	-2.71943	0.18877
N	4.45296	-1.36519	0.33227
H	5.40782	-1.69913	0.20557
N	8.27721	-3.04580	-0.29060
H	7.31639	-2.71941	-0.18873

***(P)*-PoGe[Cu, Cu] – open shell singlet**

142

Energy: -4613714.7334672

C	-5.09624	1.16331	-0.48903
C	-3.22295	-3.34405	-0.05144
C	-7.71770	-5.20635	0.43775
C	-9.54553	-0.70104	0.14255
C	-8.49536	0.19526	-0.04461
C	-8.66779	1.61862	-0.21120
C	-7.42246	2.14500	-0.39804
C	-6.48845	1.04008	-0.33400
N	-7.16681	-0.13359	-0.12037
C	-4.20983	0.07646	-0.43351
C	-2.77324	0.20596	-0.54394
C	-2.25226	-1.04624	-0.42310
C	-3.36628	-1.94432	-0.23203
N	-4.54239	-1.24863	-0.24305
C	-4.30382	-4.23768	0.11732
C	-4.14244	-5.66047	0.29265
C	-5.39544	-6.18183	0.42290
C	-6.32451	-5.07495	0.32691
N	-5.62756	-3.89976	0.14263
C	-8.60965	-4.11798	0.40051
C	-10.05059	-4.23775	0.49894
C	-10.55748	-2.97311	0.42599
C	-9.43184	-2.08333	0.27214
N	-8.26159	-2.79826	0.25799
C	-4.50522	2.52033	-0.72729
C	-4.41653	3.47306	0.30089
C	-3.79836	4.70855	0.09332
C	-3.24352	5.01330	-1.15820
C	-3.33972	4.07901	-2.20043
C	-3.96893	2.85362	-1.98250
N	-2.57957	6.25548	-1.37708
C	-1.89061	-3.83312	-0.02729
C	-0.68025	-4.04650	-0.01230
C	0.68026	-4.04649	0.01226
C	1.89062	-3.83312	0.02725
C	5.09623	1.16332	0.48902
C	9.54554	-0.70101	-0.14253
C	7.71772	-5.20633	-0.43775
C	3.22296	-3.34404	0.05141
C	3.36629	-1.94431	0.23200
C	2.25226	-1.04623	0.42307
C	2.77324	0.20597	0.54392
C	4.20983	0.07647	0.43350
N	4.54240	-1.24862	0.24304
C	6.48845	1.04010	0.33400
C	7.42245	2.14502	0.39805
C	8.66779	1.61864	0.21121
C	8.49536	0.19528	0.04462
N	7.16682	-0.13358	0.12037
C	9.43185	-2.08331	-0.27212
C	10.55749	-2.97309	-0.42596
C	10.05061	-4.23773	-0.49891
C	8.60967	-4.11795	-0.40049
N	8.26160	-2.79824	-0.25799
C	6.32453	-5.07493	-0.32692
C	5.39546	-6.18181	-0.42292
C	4.14246	-5.66046	-0.29267
C	4.30384	-4.23767	-0.11734
N	5.62757	-3.89974	-0.14264
C	4.50521	2.52034	0.72728
C	4.41652	3.47307	-0.30089
C	3.79834	4.70857	-0.09332
C	3.24351	5.01331	1.15820
C	3.33971	4.07901	2.20043
C	3.96891	2.85362	1.98250
N	2.57955	6.25549	1.37708
C	1.69893	6.87251	0.45170
C	1.20105	8.11715	1.12172
C	1.81444	8.20567	2.37221
C	2.69254	7.01622	2.56533
C	-2.69256	7.01622	-2.56532
C	-1.81447	8.20567	-2.37220
C	-1.20108	8.11715	-1.12172
C	-1.69895	6.87251	-0.45169
O	-1.44314	6.45749	0.65217
O	-3.37199	6.74352	-3.52426
C	-1.60120	9.27193	-3.23726
C	-0.73816	10.28399	-2.79873
C	-0.12546	10.20691	-1.54147
C	-0.33200	9.11813	-0.66721
C	0.33197	9.11813	0.66722
C	0.12543	10.20691	1.54148
C	0.73812	10.28399	2.79874
C	1.60117	9.27192	3.23727
O	3.37196	6.74352	3.52426
O	1.44312	6.45750	-0.65217
C	-8.28808	-6.58210	0.61193
C	-8.82400	-6.98804	1.84659
C	-9.35521	-8.27137	2.00867
C	-9.36163	-9.17027	0.93703
C	-8.83218	-8.77792	-0.29669
C	-8.29900	-7.49516	-0.45694
C	8.28810	-6.58208	-0.61192
C	8.29900	-7.49514	0.45695
C	8.83219	-8.77790	0.29671
C	9.36166	-9.17024	-0.93700
C	9.35525	-8.27135	-2.00864
C	8.82404	-6.98802	-1.84657
H	-10.55448	-0.28194	0.17740
H	-9.62677	2.13728	-0.19607

H	-7.15067	3.18446	-0.57121
H	-2.23412	1.13935	-0.68705
H	-1.20719	-1.34289	-0.46075
H	-3.18542	-6.17936	0.31586
H	-5.67408	-7.22141	0.58087
H	-10.59509	-5.17367	0.60439
H	-11.60093	-2.65887	0.46275
H	-4.82244	3.23798	1.28904
H	-3.72650	5.42473	0.91222
H	-2.92492	4.30913	-3.18189
H	-4.03077	2.13174	-2.80172
H	10.55448	-0.28191	-0.17738
H	1.20720	-1.34289	0.46071
H	2.23412	1.13935	0.68703
H	7.15066	3.18448	0.57122
H	9.62677	2.13730	0.19609
H	11.60094	-2.65884	-0.46271
H	10.59511	-5.17365	-0.60435
H	5.67411	-7.22139	-0.58088
H	3.18544	-6.17935	-0.31589
H	4.82243	3.23800	-1.28904
H	3.72649	5.42474	-0.91222
H	2.92490	4.30913	3.18189
H	4.03076	2.13175	2.80172
H	-2.09375	9.30891	-4.21227
H	-0.53406	11.14606	-3.44073
H	0.55087	11.00797	-1.22908
H	-0.55090	11.00796	1.22909
H	0.53402	11.14605	3.44075
H	2.09372	9.30890	4.21228
H	-8.81607	-6.28988	2.68895
H	-9.76303	-8.57092	2.97933
H	-9.77788	-10.17463	1.06328
H	-8.83559	-9.47327	-1.14207
H	-7.88930	-7.19082	-1.42468
H	7.88930	-7.19080	1.42469
H	8.83559	-9.47325	1.14209
H	9.77791	-10.17461	-1.06325
H	9.76308	-8.57089	-2.97929
H	8.81612	-6.28986	-2.68893
Cu	-6.40086	-2.01986	0.00973
Cu	6.40086	-2.01984	-0.00974

***(P)*-PoGe[Cu, Cu] – open shell triplet**

142

Energy: -4613714.7334044

C	-5.09624	1.16331	-0.48903
C	-3.22295	-3.34405	-0.05144
C	-7.71770	-5.20635	0.43775
C	-9.54553	-0.70104	0.14255
C	-8.49536	0.19526	-0.04461
C	-8.66779	1.61862	-0.21120
C	-7.42246	2.14500	-0.39804
C	-6.48845	1.04008	-0.33400
N	-7.16681	-0.13359	-0.12037
C	-4.20983	0.07646	-0.43351
C	-2.77324	0.20596	-0.54394
C	-2.25226	-1.04624	-0.42310
C	-3.36628	-1.94432	-0.23203
N	-4.54239	-1.24863	-0.24305
C	-4.30382	-4.23768	0.11732
C	-4.14244	-5.66047	0.29265
C	-5.39544	-6.18183	0.42290
C	-6.32451	-5.07495	0.32691
N	-5.62756	-3.89976	0.14263
C	-8.60965	-4.11798	0.40051
C	-10.05059	-4.23775	0.49894
C	-10.55748	-2.97311	0.42599
C	-9.43184	-2.08333	0.27214
N	-8.26159	-2.79826	0.25799
C	-4.50522	2.52033	-0.72729
C	-4.41653	3.47306	0.30089
C	-3.79836	4.70855	0.09332
C	-3.24352	5.01330	-1.15820
C	-3.33972	4.07901	-2.20043
C	-3.96893	2.85362	-1.98250
N	-2.57957	6.25548	-1.37708
C	-1.89061	-3.83312	-0.02729
C	-0.68025	-4.04650	-0.01230
C	0.68026	-4.04649	0.01226
C	1.89062	-3.83312	0.02725
C	5.09623	1.16332	0.48902
C	9.54554	-0.70101	-0.14253
C	7.71772	-5.20633	-0.43775
C	3.22296	-3.34404	0.05141
C	3.36629	-1.94431	0.23200
C	2.25226	-1.04623	0.42307
C	2.77324	0.20597	0.54392
C	4.20983	0.07647	0.43350
N	4.54240	-1.24862	0.24304
C	6.48845	1.04010	0.33400
C	7.42245	2.14502	0.39805
C	8.66779	1.61864	0.21121
C	8.49536	0.19528	0.04462
N	7.16682	-0.13358	0.12037
C	9.43185	-2.08331	-0.27212
C	10.55749	-2.97309	-0.42596
C	10.05061	-4.23773	-0.49891
C	8.60967	-4.11795	-0.40049
N	8.26160	-2.79824	-0.25799
C	6.32453	-5.07493	-0.32692
C	5.39546	-6.18181	-0.42292
C	4.14246	-5.66046	-0.29267
C	4.30384	-4.23767	-0.11734
N	5.62757	-3.89974	-0.14264
C	4.50521	2.52034	0.72728
C	4.41652	3.47307	-0.30089
C	3.79834	4.70857	-0.09332
C	3.24351	5.01331	1.15820
C	3.33971	4.07901	2.20043
C	3.96891	2.85362	1.98250
N	2.57955	6.25549	1.37708
C	1.69893	6.87251	0.45170
C	1.20105	8.11715	1.12172
C	1.81444	8.20567	2.37221
C	2.69254	7.01622	2.56533
C	-2.69256	7.01622	-2.56532
C	-1.81447	8.20567	-2.37220
C	-1.20108	8.11715	-1.12172
C	-1.69895	6.87251	-0.45169
O	-1.44314	6.45749	0.65217
O	-3.37199	6.74352	-3.52426
C	-1.60120	9.27193	-3.23726
C	-0.73816	10.28399	-2.79873
C	-0.12546	10.20691	-1.54147
C	-0.33200	9.11813	-0.66721
C	0.33197	9.11813	0.66722
C	0.12543	10.20691	1.54148
C	0.73812	10.28399	2.79874
C	1.60117	9.27192	3.23727
O	3.37196	6.74352	3.52426
O	1.44312	6.45750	-0.65217
C	-8.28808	-6.58210	0.61193
C	-8.82400	-6.98804	1.84659

C	-9.35521	-8.27137	2.00867
C	-9.36163	-9.17027	0.93703
C	-8.83218	-8.77792	-0.29669
C	-8.29900	-7.49516	-0.45694
C	8.28810	-6.58208	-0.61192
C	8.29900	-7.49514	0.45695
C	8.83219	-8.77790	0.29671
C	9.36166	-9.17024	-0.93700
C	9.35525	-8.27135	-2.00864
C	8.82404	-6.98802	-1.84657
H	-10.55448	-0.28194	0.17740
H	-9.62677	2.13728	-0.19607
H	-7.15067	3.18446	-0.57121
H	-2.23412	1.13935	-0.68705
H	-1.20719	-1.34289	-0.46075
H	-3.18542	-6.17936	0.31586
H	-5.67408	-7.22141	0.58087
H	-10.59509	-5.17367	0.60439
H	-11.60093	-2.65887	0.46275
H	-4.82244	3.23798	1.28904
H	-3.72650	5.42473	0.91222
H	-2.92492	4.30913	-3.18189
H	-4.03077	2.13174	-2.80172
H	10.55448	-0.28191	-0.17738
H	1.20720	-1.34289	0.46071
H	2.23412	1.13935	0.68703
H	7.15066	3.18448	0.57122
H	9.62677	2.13730	0.19609
H	11.60094	-2.65884	-0.46271
H	10.59511	-5.17365	-0.60435
H	5.67411	-7.22139	-0.58088
H	3.18544	-6.17935	-0.31589
H	4.82243	3.23800	-1.28904
H	3.72649	5.42474	-0.91222
H	2.92490	4.30913	3.18189
H	4.03076	2.13175	2.80172
H	-2.09375	9.30891	-4.21227
H	-0.53406	11.14606	-3.44073
H	0.55087	11.00797	-1.22908
H	-0.55090	11.00796	1.22909
H	0.53402	11.14605	3.44075
H	2.09372	9.30890	4.21228
H	-8.81607	-6.28988	2.68895
H	-9.76303	-8.57092	2.97933
H	-9.77788	-10.17463	1.06328
H	-8.83559	-9.47327	-1.14207
H	-7.88930	-7.19082	-1.42468
H	7.88930	-7.19080	1.42469
H	8.83559	-9.47325	1.14209
H	9.77791	-10.17461	-1.06325
H	9.76308	-8.57089	-2.97929
H	8.81612	-6.28986	-2.68893
Cu	-6.40086	-2.01986	0.00973
Cu	6.40086	-2.01984	-0.00974

References

(1) Laha, J. K.; Dhanalekshmi, S.; Taniguchi, M.; Ambroise, A.; Lindsey, J. S. A Scalable Synthesis of Meso-Substituted Dipyrromethanes. *Org. Process Res. Dev.* **2003**, *7* (6), 799–812. https://doi.org/10.1021/op034083q.

(2) Yang, Y.; Jin, P.; Ding, S.; Chu, Y.; Shen, Y. Organo-Solubility Carbazole-Containing Polyimides with Tunable Memory Characteristics Based on Different Dianhydride Moieties. *Macromol. Chem. Phys.* **2018**, *219* (17), 1800195. https://doi.org/10.1002/macp.201800195.

(3) Manka, J. S.; Lawrence, D. S. High Yield Synthesis of 5,15-Diarylporphyrins. *Tetrahedron Lett.* **1989**, *30* (50), 6989–6992. https://doi.org/10.1016/S0040-4039(01)93405-7.

(4) M. J. Frisch, G. W. Trucks, H. B. Schlegel, G. E. Scuseria, M. A. Robb, J. R. Cheeseman, G. Scalmani, V. Barone, G. A. Petersson, H. Nakatsuji, X. Li, M. Caricato, A. Marenich, J. Bloino, B. G. Janesko, R. Gomperts, B. Mennucci, H. P. Hratchian, J. V. Ortiz, A. F. Izmaylov, J. L. Sonnenberg, D. Williams-Young, F. Ding, F. Lipparini, F. Egidi, J. Goings, B. Peng, A. Petrone, T. Henderson, D. Ranasinghe, V. G. Zakrzewski, J. Gao, N. Rega, G. Zheng, W. Liang, M. Hada, M. Ehara, K. Toyota, R. Fukuda, J. Hasegawa, M. Ishida, T. Nakajima, Y. Honda, O. Kitao, H. Nakai, T. Vreven, K. Throssell, J. A. Montgomery, Jr., J. E. Peralta, F. Ogliaro, M. Bearpark, J. J. Heyd, E. Brothers, K. N. Kudin, V. N. Staroverov, T. Keith, R. Kobayashi, J. Normand, K. Raghavachari, A. Rendell, J. C. Burant, S. S. Iyengar, J. Tomasi, M. Cossi, J. M. Millam, M. Klene, C. Adamo, R. Cammi, J. W. Ochterski, R. L. Martin, K. Morokuma, O. Farkas, J. B. Foresman, and D. J. Fox,. Gaussian 09, Revision E.01.

(5) Pescitelli, G.; Bruhn, T. Good Computational Practice in the Assignment of Absolute Configurations by TDDFT Calculations of ECD Spectra. *Chirality* **2016**, *28* (6), 466–474. https://doi.org/10.1002/chir.22600.

(6) Dhbaibi, K.; Matozzo, P.; Abella, L.; Jean, M.; Vanthuyne, N.; Autschbach, J.; Favereau, L.; Crassous, J. Exciton Coupling Chirality in Helicene-Porphyrin Conjugates. *Chem. Commun.* **2021**, *57* (82), 10743–10746. https://doi.org/10.1039/D1CC03314J.

(7) Autschbach, J.; Srebro, M. Delocalization Error and “Functional Tuning” in Kohn–Sham Calculations of Molecular Properties. *Acc. Chem. Res.* **2014**, *47* (8), 2592–2602. https://doi.org/10.1021/ar500171t.

(8) Bruhn, T.; Schaumlöffel, A.; Hemberger, Y.; Bringmann, G. SpecDis: Quantifying the Comparison of Calculated and Experimental Electronic Circular Dichroism Spectra. *Chirality* **2013**, *25* (4), 243–249. https://doi.org/10.1002/chir.22138.

(9) Lu, T.; Chen, F. Multiwfn: A Multifunctional Wavefunction Analyzer. *J. Comput. Chem.* **2012**, *33* (5), 580–592. https://doi.org/10.1002/jcc.22885.

(10) Humphrey, W.; Dalke, A.; Schulten, K. VMD: Visual Molecular Dynamics. *J. Mol. Graph.* **1996**, *14* (1), 33–38. https://doi.org/10.1016/0263-7855(96)00018-5.

(11) Peeks, M. D.; Neuhaus, P.; Anderson, H. L. Experimental and Computational Evaluation of the Barrier to Torsional Rotation in a Butadiyne-Linked Porphyrin Dimer. *Phys. Chem. Chem. Phys.* **2016**, *18* (7), 5264–5274. https://doi.org/10.1039/C5CP06167A.
